# eHealth or e-Chaos: The use of Digital Health Interventions for Health Systems Strengthening in sub-Saharan Africa over the last 10 years: A scoping review

**DOI:** 10.7189/jogh.12.04090

**Published:** 2022-12-03

**Authors:** Humphrey C Karamagi, Derrick Muneene, Benson Droti, Violet Jepchumba, Joseph C Okeibunor, Juliet Nabyonga, James Avoka Asamani, Moussa Traore, Hillary Kipruto

**Affiliations:** 1 World Health Organization – Regional Office for Africa, Brazzaville, Republic of Congo; 2 World Health Organization, Geneva, Switzerland; 3 Elkay Solutions, Nairobi, Kenya; 4 World Health Organization, Harare, Zimbabwe; 5 North-West University, Potchefstroom, Mahikeng, Vanderbijlpark, South Africa; 6 World Health Organization, Ouagadougou, Burkina Faso

## Abstract

**Background:**

Digital health solutions are a potent and complementary intervention in health system strengthening to accelerate universal access to health services. Implementing scalable, sustainable, and integrated digital solutions in a coordinated manner is necessary to experience the benefits of digital interventions in health systems. We sought to establish the breadth and scope of available digital health interventions (DHIs) and their functions in sub-Saharan Africa.

**Methods:**

We conducted a scoping review according to the Joanne Briggs Institute’s reviewers manual and followed the Preferred Reporting Items for Systematic Reviews and Meta-Analyses - Extension for Scoping Reviews (PRISMA-ScR) checklist and explanation. We retrieved data from the WHO Digital Health Atlas (DHA), the WHO e-Health country profiles report of 2015, and electronic databases. The protocol has been deposited in an open-source platform – the Open Science Framework at https://osf.io/5kzq7.

**Results:**

The researchers retrieved 983 digital tools used to strengthen health systems in sub-Saharan Africa over the past 10 years. We included 738 DHIs in the analysis while 245 were excluded for not meeting the inclusion criteria. We observed a disproportionate distribution of DHIs towards service delivery (81.7%, n = 603), health care providers (91.8%, n = 678), and access and use of information (84.1%, n = 621). Fifty-three percent (53.4%, n = 394) of the solutions are established and 47.5% (n = 582) were aligned to 20% (n = 5) of the system categories.

**Conclusions:**

Sub-Saharan Africa is endowed with digital health solutions in both numbers and distinct functions. It is lacking in coordination, integration, scalability, sustainability, and equitable distribution of investments in digital health. Digital health policymakers in sub-Saharan Africa need to urgently institute coordination mechanisms to terminate unending duplication and disjointed vertical implementations and manage solutions for scale. Central to this would be to build digital health leadership in countries within SSA, adopt standards and interoperability frameworks; advocate for more investments into lagging components, and promote multi-purpose solutions to halt the seeming “e-chaos” and progress to sustainable e-health solutions.

eHealth is one of the most rapidly growing areas in health. The World Health Organization (WHO), defines eHealth as the use of information and communication technologies (ICT) for health [1]. Further, WHO defines digital health as the field of knowledge and practice associated with the development and use of digital technologies to improve health. Digital health expands the concept of eHealth to include digital consumers, with a wider range of smart devices and connected equipment, and encompasses other uses of digital technologies for health such as the internet of things, artificial intelligence, big data, and robotics[2]. In this paper, the terms eHealth and digital health are used interchangeably.

The WHO has been leading in the drive to adopt digital health as a means to improve health services and outcomes that leave no one behind. The global strategy on digital health, 2020-2025, considers digital health an accelerator to achieve health-related Sustainable Development Goals (SDGs) by improving accessibility and affordability of conventional health services[2]. The global strategy on digital health is underpinned by global resolutions related to digital health ([2], [3],[4],[5]), and a specific resolution on eHealth solutions in the African region [6]. Additionally, WHO has developed a guideline with 10-recommended digital health interventions for health system strengthening that is particularly applicable to resource-constrained settings. The proposed interventions cut across six health system strengthening building blocks: (i) service delivery, (ii) health workforce, (iii) health information systems, (iv) access to essential medicines, vaccines, and technology (v) financing, and (vi) leadership/governance. The WHO considered acceptability and feasibility in recommending digital health interventions that address the eight health system challenges (i) information, (ii) availability, (iii) quality, (iv) acceptability, (v) utilization, (vi) efficiency, (vii) cost, and (vii) accountability [7].

Sub-Saharan Africa embraced the call to utilize digital health to strengthen service delivery. Governments, donors, private entities, and universities responded to the clarion call to innovate scalable and sustainable digital interventions to strengthen health systems. Sub-Saharan Africa has arguably become a leader in the development of digital health interventions to support service delivery [8]. Digital health interventions are intended to be enablers of greater efficiency and transparency by interconnecting the different components of a functional health system [9]. The sustained push to integrate digital health into service delivery has yielded an immense number of digital interventions in the region. However, there is a paucity of systematic evidence on the number and functions of digital tools in the region.

We sought to review the digital health landscape in sub-Saharan Africa to document the extent of available digital tools in numbers, functions, users, and stages of development. This will inform policymakers on digital health successes, emerging challenges, and gaps in the region.

## METHODS

We undertook a scoping review of digital health interventions implemented in sub-Saharan Africa to strengthen health systems during the past 10 years. Based on the digital solution description, we mapped the digital interventions to the strengthened health systems building blocks, their respective target users, reported stage of development, addressed health system challenges, and aligned system categories. The scoping review was done according to the Joanna Briggs Institute’s reviewers manual [10] and followed the Preferred Reporting Items for Systematic Reviews and Meta-Analyses – extension for Scoping Reviews (PRISMA-ScR) checklist and explanation [11]. The review protocol has been deposited in an open-source platform, the Open Science Framework at https://osf.io/5kzq7 [12] and as a publication [13].

### Search Strategy

We retrieved data on sub-Saharan Africa from the global Digital Health Interventions (DHIs) repository – the WHO Digital Health Atlas (DHA) – and supplemented it with information from the WHO eHealth Observatory – eHealth country profiles report (2015). We searched for peer-reviewed publications and grey literature on DHIs in four [4] electronic databases: PubMed, HINARI-Reasearch4Life, Cochrane Library, and Google Scholar. The keywords used in the search were “Digital Health” OR “Digital Health Interventions”, OR “Digital Health Tools” OR “Digital Health Technology” AND “Africa” OR “sub-Saharan Africa”. The searches applied a filter to include documents and/or publications dated between 01/01/2011-31/12/2021. The references were screened for DHIs implemented in sub-Saharan Africa. In this study, Sub-Saharan Africa was defined as the geographical area of the continent of Africa that lies south of the Sahara and consists of lists 46 of Africa's 54 countries, excluding Algeria, Djibouti, Egypt, Libya, Morocco, Somalia, Sudan, and Tunisia.

The reviewers identified a total of 983 DHIs. These included: 466 DHIs from the DHA, 284 DHIs from the WHO eHealth Observatory – eHealth country profiles report (2015), and 233 DHIs from electronic databases. We included abstracts, complete articles, peer-reviewed publications, and articles with an English translation that described the implementation or evaluation of a DHI within sub-Saharan Africa over the study period.

### Selection for inclusion

**Figure 1:**
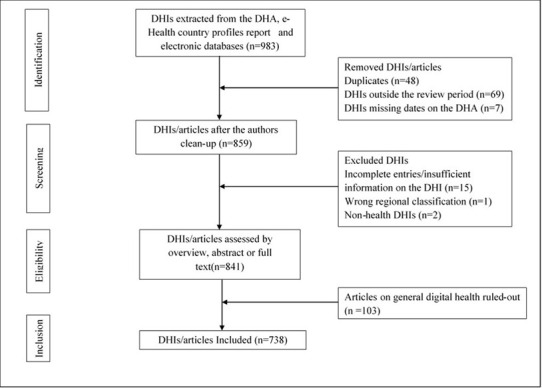
PRISMA flow diagram summarizing the selection and inclusion criteria of identified DHIs in sub-Saharan Africa.

We excluded 245 (24.9%) of the identified DHIs and articles after screening and applying the eligibility criteria. In the first step, 13% of the identified articles were excluded as these were either duplicates or outside the review period. In the second step, 2% were excluded due to insufficient information, entry errors, and wrong regional classification. In the third step, 10% were excluded as these focused on general digital health. Seventy-five percent of the originally identified articles were included in the review as shown in **Figure 1** – PRISMA flow diagram.

### Data analysis

The reviewers carried out a descriptive data analysis to determine frequencies and proportions that were tabulated based on the digital interventions’ distinct functions. We further did a spatial distribution analysis of the digital health interventions across sub-Saharan Africa. Based on the description of the DHI, the reviewers linked the DHI to one or more of the respective Health System Strengthening (HSS) building blocks – (i) service delivery, (ii) health workforce, (iii) health information systems, (iv) access to essential medicines, vaccines, and technology (v) financing and, (vi) leadership/governance - as defined by WHO health systems Building Blocks Framework [14]. The DHIs were classified as being either informal (use of ICT for health purposes in the absence of formal processes and policies), pilot (testing and evaluating a program), or established (an ongoing program that has been conducted for a minimum of 2 years and is planned to continue) according to the WHO eHealth survey 2015 [15]. Subsequently, the interventions were then mapped to either one or more of the eight [8] health system challenges namely, (i) information, (ii) availability, (iii) quality, (iv) acceptability, (v), utilization (vi), efficiency, (vii) cost, and (viii) accountability. Finally, the interventions were aligned to one or more appropriate system categories out of the twenty-five [16] categories listed in the WHO classification [17].

## RESULTS

### Digital interventions in sub-Saharan Africa

**Figure 2:**
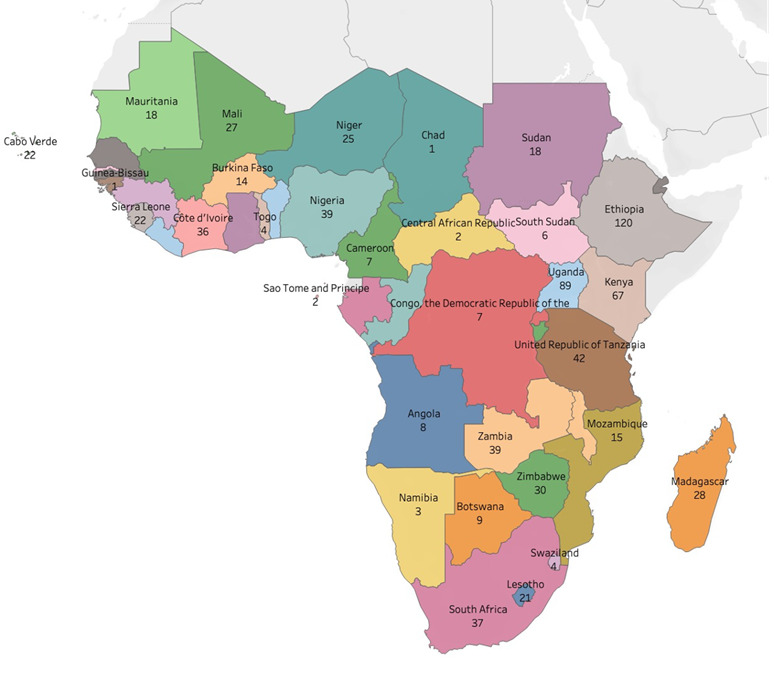
Spatial distribution of digital health interventions in sub-Saharan Africa.

A total of 738 digital health interventions (Table S1 in the **Online Supplementary Document**) were included in the final analysis. Four countries (8.5%) – Ethiopia, Uganda, Kenya, and Malawi – contributed 34% (254) of the DHIs implemented in sub-Saharan Africa over the period under review (**Figure 2**).

Out of the 738 digital health interventions included in the study, 82% (n = 603) had a component or module that aimed to improve service delivery system in the region compared to only 0.4% (n = 3) which addressed leadership and governance. Thirty-four percent (34%, n = 252) of the digital interventions strengthened more than one of the six HSS building blocks (**Table 1**).

**Table 1:** Distribution of digital health interventions as per the targeted Health System Strengthening (HSS) building block in sub-Saharan Africa

Targeted health system strengthening building block	Number of digital health interventions n (%)
Service delivery	603 (82)
Health workforce	75 (10)
Health information system	224(30)
Access to essential medicines, vaccines, and technology	56 (8)
Financing	29(4)
Leadership and governance	3(0.4)

**Figure 3:**
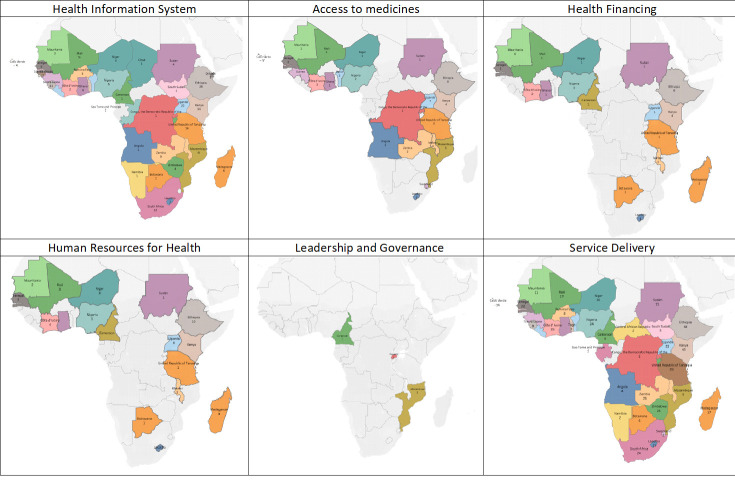
Spatial distribution of digital health interventions in sub-Saharan Africa according to their respective health systems strengthening buildings blocks (Table S3-S8 in the **Online Supplementary Document**).

Country distribution of digital health interventions in sub-Saharan Africa according to their respective health systems strengthening building blocks has been summarized in Table S2 in the **Online Supplementary Document**. The spatial distribution of digital interventions in sub-Saharan Africa revealed an uneven and sparse distribution of digital interventions in four of the six health systems strengthening building blocks with minimal investment in the use of digital health to strengthen health care financing and health leadership and governance (Figure 3, Table S3-S8 in the **Online Supplementary Document**).

### Target users of digital health interventions in sub-Sahara Africa

Ninety-two percent (92%, n = 678) of reviewed digital interventions had a domain for use by health care providers. On the other hand, data services received the least investment during the review period (**Table 2**).

**Table 2:** Distribution of digital health interventions (DHIs) as per the target user in sub-Saharan Africa

Health system strengthening building block	Target user n (%)			
**Clients**	**Providers**	**Managers**	**Data service**
Service delivery	144 (19.5)	461 (62.6)	130 (17.6)	2 (0.3)
Health workforce	3 (3.9)	68 (88.3)	6 (7.8)	0 (0)
Health information system	1 (0.4)	97 (39.8)	132 (54.1)	14 (5.7)
Access to essential medicines, vaccines, and technology	3 (3.6)	36 (42.9)	43 (51.2)	2 (2.4)
Financing	7 (23.3)	15 (50.0)	8 (26.7)	0 (0.0)
Leadership & governance	0 (0)	1 (33.3)	2 (66.7)	0 (0)
Total	158 (21)	678 (92)	321 (43)	18 (2)

### Stage of development of digital health interventions in sub-Sahara Africa

Half (53%, n = 394) of the digital interventions in sub-Saharan Africa are established. While most interventions aimed to strengthen service delivery, 36.5% (n = 221) were in pilot and 23.4% (n = 142) did not indicate the stage of development. (**Table 3**).

**Table 3:** Distribution of digital health interventions (DHIs) as per the stage of development in sub-Saharan Africa

Health system strengthening building block	Stage of development n (%)			
**Informal**	**Pilot**	**Established**	**Unknown**
Service delivery	36 (5.9)	221 (36.5)	207 (34.2)	142 (23.4)
Health workforce	7 (8.3)	22 (26.2)	37 (44.0)	18 (21.4)
Health information system	3 (1.3)	52 (22.8)	118 (51.8)	55 (24.1)
Access to essential medicines, vaccines, and technology	0 (0.0)	8 (14.8)	23 (42.6)	23 (42.6)
Financing	0 (0.0)	7 (23.3)	8 (26.7)	15 (50%)
Leadership & governance	0 (0.0)	2 (66.7)	1 (33.3)	0 (0.0)
Total	46 (6)	312 (42)	394 (53)	253 (34)

### Health system challenges addressed by digital health interventions in sub-Sahara Africa

Information (84%, n = 620), availability (53%, n = 394) and efficiency (45%, n = 330) are the most addressed health system challenges across all the six HSS-building blocks (**Table 4**).

**Table 4:** Distribution of digital health interventions (DHIs) as per the targeted health system challenge in sub-Saharan Africa

Health system strengthening building block	Health System Challenges n (%)							
**Information**	**Availability**	**Quality**	**Acceptability**	**Utilization**	**Efficiency**	**Cost**	**Accountability**
Service delivery	312 (28.0)	271 (24.3)	141 (12.7)	5 (0.4)	123 (11.0)	194 (17.4)	0 (0.0)	68 (6.1)
Health workforce	26 (17.3)	55 (36.7)	66 (44.0)	0 (0.0)	1 (0.7)	2 (1.3)	0 (0.0)	0 (0.0)
Health information system	223 (62.6)	16 (4.5)	16 (4.5)	0 (0.0)	18 (5.1)	75 (21.1)	2 (0.6)	6 (1.7)
Access to essential medicines, vaccines, and technology	51 (26.4)	51 (26.4)	2 (1.0)	0 (0.0)	2 (1.0)	45 (23.3)	0 (0.0)	42 (21.8)
Financing	5 (11.1)	1 (2.2)	0 (0.0)	0 (0.0)	0 (0.0)	13 (28.9)	13 (28.9)	14 (28.9)
Leadership & governance	3 (60.0)	0 (0.0)	0 (0.0)	0 (0.0)	0 (0.0)	1 (20.0)	0 (0.0)	1 (20.0)
Total	620 (84)	394 (53)	225 (30)	5 (0.6)	144 (20)	330 (45)	15 (2)	130 (18)

### Digital health interventions in sub-Sahara Africa and aligned system categories

Learning and training systems (18%, n = 135), electronic medical records (16%, n = 117), knowledge management systems (16%, n = 114) logistics management information systems (15%, n = 112) and telemedicine (14%, n = 104) were the most aligned system categories (**Table 5**).

**Table 5:** Distribution of digital health interventions (DHIs) according to aligned system categories in sub-Saharan Africa

System categories	Service delivery, n (%)	Health workforce, n (%)	Health information system, n (%)	Access to essential medicine, n(%)	Financing, n (%)	Leadership & governance, n (%)	Total
**A. Census, population information data warehouse**	0 (0.0)	0 (0.0)	1(100.0)	0 (0.0)	0 (0.0)	0 (0.0)	1
**B. Civil registration and vital statistics**	4 (33.3)	0 (0.0)	7 (58.3)	1 (8.3)	0 (0.0)	0 (0.0)	12
**C. Client applications**	55 (85.9)	0 (0.0)	1 (1.6)	3(4.7)	5(7.8)	0 (0.0)	64
**D. Client communication system**	74 (98.7)	0 (0.0)	1 (1.3)	0 (0.0)	0 (0.0)	0 (0.0)	75
**E. Clinical terminology and classifications**	1 (100.0)	0 (0.0)	0 (0.0)	0 (0.0)	0 (0.0)	0 (0.0)	1
**F. Community-based information system**	36 (46.2)	0 (0.0)	40 (51.3)	1 (1.3)	0 (0.0)	1 (1.3)	78
**G. Data interchange interoperability and accessibility**	1 (6.3)	0 (0.0)	14 (87.8)	1 (6.3)	0 (0.0)	0 (0.0)	16
**H. Electronic medical record**	65 (55.6)	1(0.9)	51(43.6)	0 (0.0)	0 (0.0)	0 (0.0)	117
**I. Emergency response system**	62 (91.2)	0 (0.0)	5 (7.4)	1(1.5)	0 (0.0)	0 (0.0)	68
**K. Facility management information system**	21 (84.0)	0 (0.0)	4 (16.0)	0 (0.0)	0 (0.0)	0 (0.0)	25
**L. Geographic information system**	5 (71.4)	0 (0.0)	2 (28.6)	0 (0.0)	0 (0.0)	0 (0.0)	7
**M. Health finance and insurance information system**	20 (41.7)	0 (0.0)	1 (2.1)	0 (0.0)	27 (56.3)	0 (0.0)	48
**N. Health management information system**	19 (35.2)	1 (1.9)	32 (59.3)	2 (3.7)	0 (0.0)	0 (0.0)	54
**O. Human resources information system**	3 (75.0)	0 (0.0)	1 (25.0)	0 (0.0)	0 (0.0)	0 (0.0)	4
**P. Identification registries and directories**	9 (75.0)	1 (8.3)	0 (0.0)	1(8.3)	0 (0.0)	1(8.3)	12
**Q. Knowledge management system**	88 (77.2)	7 (6.1)	19 (16.7)	0 (0.0)	0 (0.0)	0 (0.0)	114
**R. Laboratory a diagnostics information system**	32 (97.0)	0 (0.0)	0 (0.0)	0 (0.0)	1 (3.0)	0 (0.0)	33
**S. Learning and training system**	70 (51.9)	63 (46.7)	2 (1.5)	0 (0.0)	0 (0.0)	0 (0.0)	135
**T. Logistics management information system**	61 (54.5)	0 (0.0)	3 (2.7)	47 (42.0)	0 (0.0)	1 (0.9)	112
**U. Pharmacy information system**	11 (73.3)	0 (0.0)	0 (0.0)	4 (26.7)	0 (0.0)	0 (0.0)	15
**V. Public health and disease surveillance system**	12 (15.6)	0 (0.0)	65 (84.4)	0 (0.0)	0 (0.0)	0 (0.0)	77
**W. Research information system**	5 (13.9)	1(2.8)	29 (80.6)	0 (0.0)	0 (0.0)	1 (2.8)	36
**X. Shared health records and health information repositories**	11 (68.8)	0 (0.0)	5 (31.3)	0 (0.0)	0 (0.0)	0 (0.0)	16
**Y. Telemedicine**	100 (96.2)	2 (1.9)	2 (1.9)	0 (0.0)	0 (0.0)	0 (0.0)	104

## DISCUSSION

From our findings, we see documented 738 distinct digital health interventions at different levels of functioning in the SSA region over the past 10 years. These tend to be concentrated in a few countries (eg, Kenya, Uganda, Ethiopia, Malawi). One in five do not have a link to any health service outcomes. Only half of the DHIs can be classified as “established”. Two of every three are only focused on solutions in one building block, limiting integration. Most (92%) require health worker engagement for them to work. The largest proportion (84%) are focused on mining data, as opposed to improving provision of services. The SSA region is not lacking in numbers, distinct functions, variety, and complexity of digital interventions. The reviewers observed an unprecedented level of duplication within and between countries, a bias toward service delivery (81.7%) compared to the other five health systems strengthening building blocks, a preference for targeting health care providers (91.8%) to the detriment of the other three target users and a big challenge in scale-up of the interventions with only 53% reported as established. It is worrying that 84% of DHIs are focused on “data mining” of some sort, instead of improving service provision. Partiality was observed in system categories where 78.8% of the DHIs are aligned to 20% of designated system categories. The gaps in the use of digital health to strengthen health systems are obvious. The review has highlighted the need to re-strategize ideation, development, and the scale-up of digital health in the region. The World health organization adopted the health system strengthening approach to improve health and equity for all. The framework prides itself as a comprehensive holistic approach to improving health outcomes[14]. This framework is anticipated to give guidance in the alignment of interventions if the health system is to operate optimally. The fragmentation and partiality observed in the digital health landscape in sub-Saharan Africa call attention to the need to re-align digital health investments to reflect the all-inclusive ethos of health system strengthening.

The potential of digital health to supplement conventional health services is well documented in [9], [8], [18]. The eagerness to exploit this potential has resulted in an unstructured scramble to develop digital health interventions in sub-Saharan Africa. As a consequence, there is an unintentional and extreme duplication of digital tools which implies a lack of a coordinated approach and weak partnerships. We observed multiple similar tools within and between countries. The digital health landscape in sub-Saharan Africa highlights the need to develop multi-purpose tools that can be used across the health system building blocks, integrate complementary domains to strengthen health systems holistically, and scale-up implementation for maximum benefit. The few tools will then shift focus from the availability of tools to the utilization of a tool [19]. This approach provides an opportunity to evaluate the impact of digital health interventions, approaches, and outcomes on health systems. Assessing the benefits of digital health is not feasible in the current fragmented approach with high heterogeneity [20].

Only 23.7% of the tools identified in the study were articles in electronic databases and grey literature. The tools identified through electronic databases were missing in the global digital health repository. This implies a lack of knowledge sharing and dissemination in the adoption of digital health in the region. There are no reports of failures in the initial phase of pilot and experimentation, and this could be attributed to publication bias associated with failed interventions or due a to lack of interest by implementers to publish failed DHIs for fear of repercussions from funding entities or agencies. Sub-Saharan Africa lags in scholarly publishing and knowledge production [21]. This raises a pertinent question; how many digital health interventions have been developed in sub-Saharan Africa without a trace in literature?

The large-scale uncoordinated implementation of digital health tools in sub-Saharan Africa is one of the barriers to achieving the full benefits of digital health in the region. Pilots and trials are typically carried out in controlled environments. Transitioning such evidence-based interventions to large-scale complex health systems presents a new set of challenges including but not limited to government policies and the end of funding cycles from donors. If such interventions are not adopted by governments and funding sustained, they will close with the project cycle. The sustainability of funding in digital health has given government-led interventions an advantage over private interventions. Over and above the benefits to health systems, interventions developers and financiers need to take into consideration the return on investment, capital investments, operational costs, and how long they are willing to invest in the tool [22]. The Principles of Donor Alignment for Digital Health [23] which was launched on October 16, 2018, at the Berlin summit – were set against the backdrop of digital health fragmentation, duplication, and lack of interoperability that characterized many developing countries’ digital health systems and are consistent with the findings of this paper [23]. These principles urge the development partners to align investments with national digital health strategies. To do so, the development community needs to strengthen digital health collaboration within the context of national digital health strategies; prioritize investments that incorporate digital global goods; quantify and support sustainable costs for the long term; strengthen, track and measure progress in the digital health echo system; enhance donor skills to implement the principles for digital development establish and strengthen the maturity of digital health continuum; develop the country capacity to lead digital health interventions; support the update of digital global goods; encourage sharing and peer-learning to foster coordination and alignment of implementation activities. To alleviate the gaps observed in this review, countries in sub-Saharan Africa need to implement and leverage these principles across the spectrum of digital health interventions.

Proof of concept is not enough to propel digital tools into adoption for large-scale use. Successful implementation of digital health interventions requires a balance of a good digital tool, a receptive user, and an ideal context at the policy and implementation level. To achieve this balanced mix, the proposed tools must have an advantage over conventional service delivery approaches and be easy to use without the need for advanced training, adoption of new skills, or further resource investment [24]. The developers must be clear the on quality of the interventions in comparison to conventional services, and their value for users [25].

### Strengths and limitations

The mapping process relied on overviews and descriptions provided by the developers and authors of the digital interventions. The mapping process is not fool proof to a difference in understanding and interpretation. It should be noted that some digital health interventions were designed to address more than one health system building block, address more than one health system challenge, or target more than one user, hence they were not mutually exclusive, and interpretation of the findings should be in this context. Voluntary registration of tools and the minimal sharing of information on digital health tools in electronic databases and grey literature created an opportunity to miss tools during the review. The digital health interventions extracted from the digital health country profiles did not provide references hence a potential duplication during data extraction. The large number of tools identified and reviewed should conceal any biases that would be generated by missed tools.

## CONCLUSIONS

Our review suggests the current digital health landscape in SSA leans more towards echaos than ehealth. Multiple overlapping solutions with limited focus and scalability, coupled with a demand on health workers define the existing solutions. Instead of helping to expand access to quality services demanded by populations[16], many of the solutions are data mining operations of limited benefit to users. It is time for digital health policymakers in sub-Saharan Africa to establish measures and institutions to guide, coordinate and provide oversight for further adoption of digital health in the region. The aim is to stop further duplication, encourage interventions that holistically strengthen the health systems, and direct future investments towards lagging components of the health system. All stakeholders need to re-look the digital health focus and funding model to minimize the collapse of interventions after the initial pilot and trial phases. Potential and proof of concept do not translate to the adoption and strengthening of health systems. We have to incorporate implementation science approaches, models, and frameworks to increase the chances of successful scale-up. There is an urgent need for a platform to share successful and failed interventions to stop the continuous reinvention of the wheel. Digital health in Sub-Saharan Africa is endowed with numbers, distinct functions, variety, and complexity. It is however riddled with preferences and biases towards specific components of the health system, immense duplication, minimal learning, sharing, and dissemination, a profound lack of coordination, and a lack of consideration for business sense. The potential for digital health in sub-Saharan Africa is unquestionable. The realization of these benefits to strengthen health systems is possible. The big question is how to move from potential to reality while retaining quality, value, and order. To swing from echaos to ehealth, the region requires digital health interventions that are (i) applicable across multiple contexts, (ii) aimed at improving multiple health service outcomes, (iii) incorporate multiple health system building blocks, (iv) have a minimal additional demand on health workers time, and (v) are not just data mining operations.

## Additional material


Online Supplementary Document.

## References

[1] WHO. Global Observatory for eHealth [Internet]. 2022 [cited 2022 Jun 20]. Available from: https://www.who.int/observatories/global-observatory-for-ehealth

[2] World Health Organization. Global strategy on digital health 2020-2025. World Health Organization; 2021 10.2471/BLT.20.253955 PMC7133480 32284641

[3] World Health Organization. Resolution wha58. 28. ehealth. Fifty-eighth World health assembly, Geneva. 2005;16–25.

[4] World Health Organization eHealth standardization and interoperability. Sixty-Sixth World Health Assembly. 2013;WHA66:24.

[5] Organization WH. Digital health. Draft resolution proposed by Algeria, Australia, Brazil, Estonia, Ethiopia, Germany, India, Indonesia, Israel, Italy, Luxembourg, Mauritius, Morocco, Panama, Philippines, and South Africa. 71st WH Assembly agenda item 12.4 [Internet]. 2018;

[6] World Health Organization 2010. Resolution: eHealth solutions in the African Region: Current context and perspectives. AFR/RC/60/R3; 2010.

[7] World Health Organization. WHO guideline: recommendations on digital interventions for health system strengthening. World Health Organization; 2019. 31162915

[8] Holst C Sukums F Radovanovic D Ngowi B Noll J Winkler AS Sub-Saharan Africa—the new breeding ground for global digital health. The Lancet Digital Health. 2020;2:e160-2. 10.1016/S2589-7500(20)30027-3 33328076

[9] Neumark T Prince RJ Digital Health in East Africa: Innovation, Experimentation and the Market. Global Policy. Wiley Online Library. 2021;12:65-74.

[10] Peters M Godfrey C Khalil H Mcinerney P Soares C Parker D 2017 guidance for the conduct of JBI scoping reviews. Joana Briggs Inst Rev Man. 2017;13:141-6.

[11] Tricco AC Lillie E Zarin W O’Brien KK Colquhoun H Levac D PRISMA extension for scoping reviews (PRISMA-ScR): checklist and explanation. Annals of internal medicine. Ann Intern Med. 2018;169:467-73. 30178033 10.7326/M18-0850

[12] Foster ED Deardorff A Open science framework (OSF). Journal of the Medical Library Association: JMLA. Medical Library Association. 2017;105:203.

[13] Kipruto H, Muneene D, Droti B, Jepchumba V, Okeibunor CJ, Nabyonga-Orem J, et al. Use of Digital Health Interventions in Sub-Saharan Africa for Health Systems Strengthening Over the Last 10 Years: A Scoping Review Protocol. Frontiers in digital health. Frontiers Media SA; 2022;4. 10.3389/fdgth.2022.874251 PMC9120370 35601887

[14] World Health Organization. Everybody’s business–strengthening health systems to improve health outcomes: WHO’s framework for action. World Health Organization; 2007

[15] WHO. Atlas of EHealth Country Profiles: Based on the Findings of the Third Global Survery on EHealth 2015. World Health Organization; 2016.

[16] Karamagi HC Tumusiime P Titi-Ofei R Droti B Kipruto H Nabyonga-Orem J Towards universal health coverage in the WHO African Region: assessing health system functionality, incorporating lessons from COVID-19. BMJ global health. BMJ Glob Health. 2021;6:e004618. 10.1136/bmjgh-2020-004618 33789869 PMC8015798

[17] Organization WH. Classification of digital health interventions v1. 0: a shared language to describe the uses of digital technology for health. World Health Organization; 2018.

[18] Tambo E Madjou G Mbous Y Olalubi OA Yah C Adedeji AA Digital health implications in health systems in Africa. Eur J Pharm Med Res. 2016;3:91-3.

[19] Torous J, Vaidyam A. Multiple uses of app instead of using multiple apps–a case for rethinking the digital health technology toolbox. Epidemiology and psychiatric sciences. Cambridge University Press; 2020;29. 10.1017/S2045796020000013 PMC7216034 32000876

[20] Howarth A Quesada J Silva J Judycki S Mills PR The impact of digital health interventions on health-related outcomes in the workplace: A systematic review. Digital health. Digit Health. 2018;4:2055207618770861. 10.1177/2055207618770861 29942631 PMC6016571

[21] Ondari-Okemwa E Scholarly publishing in sub-Saharan Africa in the twenty-first century: Challenges and opportunities. First Monday. 2007. 10.5210/fm.v12i10.1966

[22] Marwaha JS Landman AB Brat GA Dunn T Gordon WJ Deploying digital health tools within large, complex health systems: key considerations for adoption and implementation. npj Digital Medicine. Nature Publishing Group. 2022;5:1-7. 10.1038/s41746-022-00557-1 PMC8795422 35087160

[23] The Principles of Donor Alignment for Digital Health [Internet]. Available: https://digitalinvestmentprinciples.org, Accessed: 17 September 2022/. Available from: https://digitalinvestmentprinciples.org/

[24] Connolly SL, Hogan TP, Shimada SL, Miller CJ. Leveraging implementation science to understand factors influencing sustained use of mental health apps: a narrative review. Journal of technology in behavioral science. Springer; 2021;6:184–96. 10.1007/s41347-020-00165-4 PMC7476675 32923580

[25] Mathews SC McShea MJ Hanley CL Ravitz A Labrique AB Cohen AB Digital health: a path to validation. NPJ digital medicine. Nature Publishing Group. 2019;2:1-9. 10.1038/s41746-019-0111-3 PMC6550273 31304384

