# Supplementary material for: eHealth or e-Chaos: The use of Digital Health Interventions for Health Systems Strengthening in sub-Saharan Africa over the last 10 years: A scoping review
Source: J Glob Health. 2022 Dec 3;12:04090. doi: 10.7189/jogh.12.04090 (PMC9718445; doi:10.7189/jogh.12.04090)
Supplement: Online Supplementary Document. [file jogh-12-04090-s001.pdf]

Table of Contents

1.0 Online Supplementary Document 2

Table S1: List of Digital Health Interventions in sub-Saharan Africa included in the scoping review analysis 2

Table S2: Supplementary material: Country Distribution of digital health interventions according to the health systems strengthening building blocks 173

Table S3: Supplementary material: Digital Health Interventions used to strengthen service delivery in sub-Saharan Africa 175

Table S4: Supplementary material: Digital Health Interventions used to strengthen health workforce in sub-Saharan Africa 228

Table S5: Supplementary material: Digital Health Interventions used to strengthen Health Information System in sub-Saharan Africa 234

Table S6: Supplementary material: Digital Health Interventions used to strengthen Access to Essential Medicines, Vaccines, and Technology in sub-Saharan Africa 257

Table S7: Supplementary material: Digital Health Interventions used to strengthen Healthcare Finance in sub-Saharan Africa 263

Table S8: Supplementary material: Digital Health Interventions used to Leadership and Governance strengthen in sub-Saharan Africa 266

1.0 Online Supplementary Document

Table S1: List of Digital Health Interventions in sub-Saharan Africa included in the scoping review analysis

Name/Reference/Point of contact	Country	Start Date	Overview of digital health implementation	HSS building block
Projecto Exemplar Elon Musk, elon@tesla.com	Angola	01/01/2018	Reminders to patients for missed appointments for malaria diagnosis follow-up	Service delivery
IRS - Cunene Sergio Lopes, sergio@mentor-initiative.net	Angola	01/10/2018	Kobo platform for a digital collection of IRS data using tablets was trialed.	Health information system, Service delivery
OpenLMIS Angola (SIGLOFA) Rebecca Alban, info@openlmis.org	Angola	01/03/2019	This is an OpenLMIS system being used in provincial warehouses and hospitals. It includes requisitions, stock management, and a new reporting stack. It includes over 750 commodities. Preparatory activities are currently underway to support the national-level rollout of SIGLOFA.	Service delivery, Access to medicines, vaccines, and technology
Angola immunization supply chain Neeraj Thakare, neeraj@logistimo.com	Angola	24/07/2019	In partnership with Gavi, Logistimo's SCM platform has been deployed in 6 provinces, across ~500 health facilities to improve the visibility, tracking, and availability of vaccines from the central level to the last level. The deployment was localized keeping in mind the native context including the Portuguese language.	Service delivery, Access to medicines, vaccines, and technology
Kassai Anya Fedorova, Anya.Fedorova@psiangola.org	Angola	10/11/2019	Kassai is an e-learning solution for Health professionals in Angola. It is the fastest and easiest way for health professionals to have access to training. The platform contains several multimedia courses concerning Malaria and family planning. At this stage, the platform has over 1500 health workers enrolled in 5 important provinces of Angola. It will go to national scale in the near future.	Human resources for health

The Safe Delivery App in Benin Lauren Bellhouse, lauren@maternity.dk	Benin	01/10/2018	Following the successful pilot of the Safe Delivery App (SDA) French Global version as a job aid with 15 health champion trainers and 100 health care workers, the SDA is now introduced as a pre-service training tool with midwifery trainers and students in the two national midwifery schools. The job aid pilot was concluded in January 2018 with a significant increase in knowledge and confidence of the included health care workers. In collaboration with the Directorate for Maternal and Child Health, Plan International Benin, and Plan International Denmark, Maternity Foundation is in the process of creating a Benin-specific version of the App adhering to national guidelines and directives.	Service delivery, Human resources for health
Apprendre a Vivre Benin Uju Ofomata, uju.ofomata@oneworld.org	Benin	01/10/2016	The project has three components. First, the mobile application, Ma Vie Mon Choix provides games, videos, maps of service providers, a confidential question and answer services for adolescents related to SRHR. Second, an eLearning application provides SRHR-related videos, lesson plans, activities, and discussion questions to teachers for use in class. Third, the Tro Tro Ga Ho! website provides information on gender-based violence laws and resources in Benin for service providers.	Service delivery
NTD LF TAS and Coverage Surveys Clara Burgert, cburgert@rti.org	Benin	01/03/2019	Use of Secure Data Kit platform to collect disease prevalence survey information for LF pre-TAS, TAS surveys, and coverage evaluation surveys.	Health information system

OpenLMIS Benin (SIIL) Rebecca Alban, info@openlmis.org	Benin	01/01/2015	The SIIL system was developed in collaboration with AMP and the Beninese government and partners in order to facilitate electronic data collection through the informed push system. SIIL is a locally customized variant of the OpenLMIS free software developed by a partnership of organizations working internationally to address logistical challenges. An assessment of the outcomes of the demonstration project indicated a marked improvement in performance indicators for the immunization logistics system on the Comé site. As a result, the Benin Ministry of Health took the decision to deploy the strategy, and the SIIL software, across the country. Further expansions of the system took place in 2016 and 2017, resulting in the expansion of the system to 10 additional health zones.	Service delivery, Health information system, Access to medicine, vaccines and technology
DHIS2 for COVID-19 Surveillance: Botswana Rebecca Potter, covid@dhis2.org	Botswana	08/05/2020	The DHIS2 digital data package for COVID-19 is designed to accelerate case detection, situation reporting, active surveillance, and response in countries. The COVID-19 digital data package includes standard metadata aligned with the WHO's technical guidance on COVID-19 surveillance and has been adapted local country context and language in this implementation.	Health information system
Integrated e-Diagnostic Approach Thierry Agagliate, tag@tdh.ch	Burkina Faso	01/01/2014	IeDA provides a complete solution for delivering quality care to under-fives, supporting healthcare workers through a digital clinical job aid (on a tablet device), providing performance data to facility and district managers for management purposes, supporting coaching & continuous training of healthcare workers. IeDA has been deployed in 600+ facilities (1000 by the end of 2019) and guided 3000+ healthcare workers, delivering 120,000+ consultations/month for 1 million children per year .	Service delivery, Health Information System

suivi des diabétiques Sanou Mohamed, sanou45@gmail.com	Burkina Faso	23/05/2019	suivi des malades à partir de leur téléphone portable	Service delivery
Suivi des malades diabitiques au Burkina Sanou Abdoulaye, sanou45@gmail.com	Burkina Faso	29/06/2019	Ce projet utilise les téléphones mobiles pour suivre à distance certains malades en les fournissant des informations et conseils liés à leur hygiène aliementaire	Service delivery
Projjet Télé cardiologie Ouedraogo, sanou60@gmail.com	Burkina Faso	24/05/2019	Ce projet permet à certains structures sanitaires de l'intérieur du pays ne disposant pas de cardiologue d'utiliser ce réseau pour receuillir des avis et expertises des cardiologues de Ouagadougou	Service delivery
One Health Platform Romain Tohouri, romain_tohouri@jsi.com	Burkina Faso	01/10/2018	The OH system in Burkina Faso is composed of three electronic disease surveillance platforms customized using DHIS2 to collect, transfer, and analyze all data needed by each ministry involved in the OH approach -- the MOH, MRAH, and MEEVCC. Each ministry has full control over its own database and can track as many diseases as it wishes, although a core set of agreed-upon diseases is common to all three databases. To ensure the functionality of this decentralized system, a fourth database was set up to receive real time aggregate data from the three ministry databases. An interoperability layer ensures that reported cases, suspected cases, and confirmed cases are automatically populated into the fourth database and that email and text message alerts are sent when an outbreak is detected. The electronic platform is now used as the Burkina Faso national COVID-19 data collection platform across the country. The data is collected using mobile tablets.	Health information system
DHIS2 for COVID-19 Surveillance: Burkina Faso Boukary, ouedbouks@gmail.com	Burkina Faso	08/05/2020	Health authorities and local implementing partners configured and deployed DHIS2 for Covid-19 surveillance.	Health information system

Bio-fortified Value Chains for Improved Maternal and Child Nutrition (B4MCN) Bertin Nduwayo, bertin_nduwayo@wvi.org	Burundi	01/08/2017	Community health workers (CHWs) in Burundi use weight and mid-upper arm circumference (MUAC) to monitor a child's level of malnutrition. Because the standards for rehabilitation are given by weight for age, CHWs found it difficult to identify children who were wasting because they were underweight for their height, and many underweight children who did not meet those thresholds were overlooked.<br><br>Taking a multi-stakeholder approach that included input from multiple government ministries and UNICEF, B4MCN introduced the CommCare application in 2019 and trained CHWs to use the tool effectively. The approach has added great value, as it combines both weight and MUAC information readily to clearly indicate the child's nutritional status. Now, all children in the project area who suffer from malnutrition due to wasting or being underweight receive appropriate support. Encouraged by the B4MCN project's success, donors in Burundi are looking to use this solution in other programs.	Service delivery, Health information system
DHIS2 for COVID-19 Surveillance: Cabo Verde Rebecca Potter, covid@dhis2.org	Cabo Verde	08/05/2020	The DHIS2 digital data package for COVID-19 is designed to accelerate case detection, situation reporting, active surveillance and response in countries. The COVID-19 digital data package includes standard metadata aligned with the WHO's technical guidance on COVID-19 surveillance and has been adapted to local country context and language in this implementation.	Health information system

Building a national health map in a week Yaw Anokwa, yanokwa@nafundi.com	Cameroon	15/12/2015	To build on the country's micro-planning success, the Ministry of Health chose to create a detailed and up-to-date map with important points of interest like health facilities, settlements, markets, churches/mosques, schools and health area boundaries. These locations are critical for planning immunization campaigns and the easiest way to build this national health map was with mobile data collection. The project used 400 data collectors to submit more than 80,000 forms in a week.	Leadership and governance
Implementation of a Performance-Based Financing Data System for Cameroon - using the open source tool Hesabu Mfouapon N Henock, National Data Manager of PBF, henockmfouapon@yahoo.fr	Cameroon	01/02/2017	Cameroon - Data System to Manage the performance based financing program including offline mobile data collection of quality of care and client satisfaction, the computation of program indicators, the calculation of incentive bonuses, the management of the PBF primary and subcontractor relationships and distribution of incentives. In addition, data is displayed publicly through open data dashboards.	Service delivery, Healthcare finance
Bafia District Hospital Armand Mpassy-Nzoumba, armandmpassy@yahoo.com	Cameroon	01/01/2018	GNU Health was successfully implemented at the Bafia District Hospital as part of WHO's the strategy in Cameroon to strengthen the national health system in Cameroon. The Bafia hospital serves as a pilot project for the implementation of the strategy. The project has made it possible to:<br>-improving the quality of patient care<br>-improving hospital management<br>-improving health prevention<br>-improving health statistics<br>- improving the capacity and productivity of the hospital's health care staff<br>We have deployed the system in the following hospital units:<br>Management, Cashier, Outpatient unit, Mother-Child Consultation, Inpatient unit, Medical Analysis Laboratory, Maternity, Neonatology, HIV/AIDS care unit.	Service delivery

Bikop Health Center Armand MPASSY, GNU Solidario, armandmpassy@yahoo.com	Cameroon	09/03/2017	As part of its strategy to support the health system in Cameroon, the Recover Foundation has initiated a program to support the development of non-profit hospitals. Thus, it initiated the computerization project of the Bikop hospital in cooperation with GNU Solidario. The project aims to :<br>-improving the quality of care<br>-improvement of hospital management<br>-the improvement of health prevention<br><br>This project will serve as a pilot for the implementation of hospital management systems in other hospitals supported by the Recover Foundation.<br>The project was developed on the basis of GNU Health, a health information and hospital management system.	Service delivery
DHIS2 for COVID-19 Surveillance: Cameroun (in development) Rebecca Potter, covid@dhis2.org	Cameroon	08/05/2020	The DHIS2 digital data package for COVID-19 is designed to accelerate case detection, situation reporting, active surveillance and response in countries. The COVID-19 digital data package includes standard metadata aligned with the WHO's technical guidance on COVID-19 surveillance and has been adapted to local country context and language in this implementation.	Health information system
Klarah Ginyu Innocentia Kwalar, hello@klarah.com	Cameroon	19/10/2021	We use mobile applications and dashboards to match nurses to patients based on patient need, carer experience and their proximity to each other.	Service delivery
Suivi de de la vaccination des enfants de moisn d'un an Sessouma Abdoulaye, sesabdnaz@yahoo.fr	Chad	01/06/2019	Le projet consistera à colecter les données de vaccination sur les enfan,ts sur une periode d'un an	Health information system

mHealth with ESPEN Collect REBOLLO POLO Maria, rebollopolom@who.int	Congo	01/01/2015	ESPEN has developed a mobile data collection tool called ESPEN Collect. ESPEN Collect is a tool for the national programs in WHO's AFRO region.<br>The application is compatible with Android devices and maintained. It is using a secured server to store the data (PostgreSQL). It uses survey forms designed using the XLSform or XML form syntax. Those forms are compatible with ODK ecosystem (Tropical data, Kobo Collect, ESPEN Collect, etc.)<br>For any disease-specific survey, ESPEN and partners will provide scientific review of protocols, standardized training, real-time data management, and support to field supervisors, and the results of the survey will immediately be available on the ESPEN portal and incorporated into automated processes to produce the EPIRF.	Health information system
The Prevention Pack Program: ensuring availability of post-rape medicines in rural clinics Jean Armas, jean.armas@globalstrategies.org	Congo, the Democratic Republic of the	01/03/2013	The Prevention Pack program improves access to HIV prophylaxis, emergency contraception and antibiotics for rape survivors. The program combines community sensitization about post-rape medical care with the provision of a pre-bundled post-rape medical kit (the Prevention Pack) and the implementation of a cloud-based, GPS-enabled inventory management system called Logistimo. The project was originally implemented in 2013 at 12 rural clinics in the South Kivu Province and has now expanded to 45 sites in partnership with IMA World Health. The data manager calls all sites daily to determine demand for post-rape care and Prevention Pack consumption. Inventory data is entered into Logistimo and project personnel use the consumption rate, trends and geolocation of sites to guide Prevention Pack restocking strategy. To date, there have been zero medication stock outs. To learn more, read our article in BMJ Global Health:	Service delivery, Access to medicine, vaccine and technology

			http://bit.ly/2v0HZaQ or visit us on the web at: http://bit.ly/2v3Bkg5	
Connecting persons seeking information about HIV/AIDS to HIV medical and social service organizations Jean Armas, jean.armas@globalstrategies.org	Congo, the Democratic Republic of the	01/09/2014	Foundation Femme Plus (FFP), a DRC-based non-profit, partnered with us to create a digital resource map and toll-free information hotline to help connect community members seeking information about HIV/AIDS to HIV medical and social services in Bukavu, DRC. FFP visited medical and social service organizations serving persons living with HIV/AIDS (PLWHA) throughout Bukavu and recorded contact information, service availability and GPS coordinates. The data was collected offline with tablets using a mobile data collection tool. The online resource map was created using a geographic information system software called CartoDB. The map is continuously validated and updated by FFP and their government partner to ensure the map is always kept current. In early 2017, the toll-free hotline was activated. Based on the content of the call, FFP operators then provided callers with the physical addresses and phone numbers of local community resources, such as confidential HIV testing facilities.	Service delivery
Meilleur santé accessible à tous EPENGE DJONGA EMMANUEL, Falbalardc@gmail.com	Congo, the Democratic Republic of the	12/04/2019	Congo medika est une application médicale pour smartphone qui facilite la mise en relation entre un patient et un médecin selon le problème de santé identifié: elle permet donc une consultation à domicile ou à l'hôpital . Elle permet en outre la livraison à domicile des médicaments et la publication d'informations de santé accessible à tous.	Service delivery
DHIS2 for COVID-19 Surveillance: Congo DRC (in development) Rebecca Potter, covid@dhis2.org	Congo, the Democratic Republic of the	08/05/2020	The DHIS2 digital data package for COVID-19 is designed to accelerate case detection, situation reporting, active surveillance and response in countries. The COVID-19 digital data package includes standard metadata aligned with the WHO's technical guidance on COVID-19 surveillance and has been adapted to local	Health information system

			country context and language in this implementation.	
OpenELIS Global in Cote d'Ivoire Casey Iiams-Hauser, caseyi@uw.edu	Côte d'Ivoire	01/01/2011	OpenELIS is used to improve the quality of lab results and make producing high quality work easier for the users. Users are primarily medical lab personnel. Lab Technicians, Reception, Biologists and Management. The data links to a national dashboard which allows lab data to be used in decision making by a wide variety of stakeholders.	Service delivery
OpenLMIS Cote d'Ivoire (eSIGL) Rebecca Alban, info@openlmis.org	Côte d'Ivoire	01/07/2013	In late 2014, a series of workshops and meetings were held with stakeholders and subject matter experts to develop and validate system and functional requirements to guide the configuration of the electronic LMIS. The objective of eSIGL is to enable the direct customers (health districts and hospitals) of the Central Medical Store of Côte d'Ivoire (NPSP-CI) to place orders and to provide reports on the management of drugs.	Access to medicines, vaccines and technology, Service delivery
COVID-19 Vaccine Allocation App Anubhuti Mishra, anubhuti.mishra@thepalladiumgroup.com	Côte d'Ivoire	18/10/2021	COVID-19 vaccination poses an unprecedented challenge to country governments. Countries need to rapidly vaccinate their susceptible populations across geographic regions under complex infrastructure, supply, and demand constraints. This formidable task requires detailed planning and complex allocation of limited resources, with flexibility and adaptation as difficulties arise. Thus, our product is targeted at ministries of health in low- and middle-income countries (LMICs) to support their decision-making. We have a deep understanding of the needs and challenges of healthcare systems in LMIC, which gives us a unique advantage in building a customized product that is affordable and easy to use.	Service delivery, Health information system

DHIS2 for COVID-19 Surveillance: Djibouti Rebecca Potter, covid@dhis2.org	Djibouti	08/05/2020	The DHIS2 digital data package for COVID-19 is designed to accelerate case detection, situation reporting, active surveillance and response in countries. The COVID-19 digital data package includes standard metadata aligned with the WHO's technical guidance on COVID-19 surveillance and has been adapted to local country context and language in this implementation.	Health information system
Hiwot: Addressing Maternal & Neonatal Health Stefanie Kot, skot@d-tree.org	Ethiopia	01/10/2017	A child and newborn digital health system for use by Health Extension Workers as they assess, refer and follow up with sick children. The system includes iCCM and newborn visits, immunization reminders and documentation, e-learning tools, on-app monthly reports, referral and follow-up reminders, and a supervisory system. As part of the project, a dashboard has been created to provide patient level data to health workers and their supervisors, as well as government reports.	Service delivery, Health information system
OppiaMobile Alex Little, consult@alexlittle.net	Ethiopia	01/08/2013	Delivering a digital version of the FMoH national curriculum for the upgrade training of 160 Health Extension Workers, enhanced with additional video and quiz content, and a common/open platform (OppiaMobile). Few of the HEWs had access to the hardcopy versions of the curriculum and so limited opportunity to review and practice. The HEWs were followed up for a year after the training was completed, to evaluate how the skills learned were being put into practice.	Service delivery, Human resources for health
Medical Incident Reporting System Innovation (MIRSI) Fiseha Tesfaw, fiseha.tesfaw@jhpiego.org	Ethiopia	01/03/2017	Project aims to provide a medical incident reporting system. The current system is not anonymous, is punitive, or not in place at all, an easier, technology supported voluntary medical incidents reporting system was necessary. Project has been tasted in six tertiary level hospitals and proved the technology driven approach has given the number of incidents reported a huge boost.	Service delivery

Blood Safety Strengthening Programme: Blood Safety Information System (BSIS) Rhonwyn Cornell, rhonwyn.cornell@jembi.org	Ethiopia	01/12/2016	Safe blood is used to treat postpartum haemorrhage, childhood malaria, severe anemia, trauma and surgery. The BSSP develops and implements BSIS, an open source system designed to manage donor and donation information from the point of donation to transfusion. BSIS is for resource-limited blood services and supports AfSBT accreditation processes. Our implementations follows international good practice, building local capacity within the blood services to use, manage and maintain BSIS.	Service delivery
VERS Federal Ministry of Health, dha@moh.gov.et	Ethiopia	01/05/2017	VERS stands for Vital Events Registration System. It is used to register 4 major life events (birth, death, marriage, and divorce), and generate various reports. It is also used to check if events registered are current (events registered within the standard period).	Health information system
Tenaye Federal Ministry of Health, dha@moh.gov.et	Ethiopia	01/01/2018	Educational platform for sexual and reproductive health, mental health adolescent	Service delivery
Tena PIMS Federal Ministry of Health, dha@moh.gov.et	Ethiopia	01/08/2017	Patient information management system designed to manage all services in the hospital fully avoid paper work activities	Service delivery, Health information system
SuperHIM (Super HIMS health information management system) Federal Ministry of Health, dha@moh.gov.et	Ethiopia	01/10/2017	SuperHIMS is an integrated health management system developed by bet tech institute of technology. The system incorporates card room, triage, different specialized opd, laboratory, imaging and generate many types of reports.	Service delivery
SmartEMS (Samrt Electronic Medical System) Federal Ministry of Health, dha@moh.gov.et	Ethiopia	01/01/2017	Smart EMS is software system design to automate work, record management and reporting of small to large clinics and hospitals.	Service delivery, Health information system
RI (routine immunization dashboard) Federal Ministry of Health, dha@moh.gov.et	Ethiopia	01/09/2016	Vaccine Performance measurement tool	Health information system
Quntimed and Quentitb, Federal Ministry of Health, dha@moh.gov.et	Ethiopia	01/07/2018	Forecasting of medicine and supplies	Service delivery, Access to medicine, vaccine and Technology

PRS (Patient Registration System) Federal Ministry of Health, dha@moh.gov.et	Ethiopia	01/01/2016	PRS is an easy and simple system designed and developed by Jimma university ICT staff and only used to register 2 things: patient data like name and address; and bio data of a patient like a disease encountered. And generally it was designed after the failure of smart care system.	Health information system, Service delivery
Providers System Federal Ministry of Health, dha@moh.gov.et	Ethiopia	01/01/2018	Information about who used the insurance on the providers	Healthcare Financing
PrimeCare electronic health record Federal Ministry of Health, dha@moh.gov.et	Ethiopia	01/05/2014	The PrimeCare electronic health record is a desktop application used to record and manage patient information. Currently, the app is used only for patient card registration and retrieval.	Health information system, Service delivery
Pipeline Federal Ministry of Health, dha@moh.gov.et	Ethiopia	01/07/2018	To monitor the procurement and shipment status of medicines and supplies	Access to medicine, vaccine and technology, Service delivery
PaxscanL06 Federal Ministry of Health, dha@moh.gov.et	Ethiopia	01/10/2017	Reading radiology images	Service delivery
Patient Registration Software Federal Ministry of Health, dha@moh.gov.et	Ethiopia	01/07/2018	To register patient medical record	Service delivery, Health information system
PACS (Picture Archiving and Communication System) Federal Ministry of Health, dha@moh.gov.et	Ethiopia	01/02/2018	Collect and share an image of radiography	Service delivery
OTRS (Online Ticket Registration System) Federal Ministry of Health, dha@moh.gov.et	Ethiopia	01/01/2018	To provide an interface for internal maintenance and support. Using this application, users request hardware and software maintenance and troubleshooting by filling a form.	Service delivery
Orbit Health Federal Ministry of Health, dha@moh.gov.et	Ethiopia	01/09/2015	Enables to capture information about kidney donor and recipients and manage all processes through kidney operations	Service delivery
OR management system Federal Ministry of Health, dha@moh.gov.et	Ethiopia	01/12/2016	OR Schedule, progress, reports	Service delivery
Open Clinic Federal Ministry of Health, dha@moh.gov.et	Ethiopia	01/12/2016	A general hospital management system including financial transaction, admission, opd, in patient service, private, public, credit, debits and extra services. Generally wonderful system for EMR	Service delivery, Health information system

OPAC (Koha software open public access cataloge) Federal Ministry of Health, dha@moh.gov.et	Ethiopia	01/02/2017	To give detailed status of electronic health books and number of books in the library	Human resources for health
NHDD Federal Ministry of Health, dha@moh.gov.et	Ethiopia	01/04/2017	Captures the definition of data elements, indicators and the relationship between those elements to improve the MOH ability to manage data recording and reporting guidelines.	Health information system
National residency matching Federal Ministry of Health, dha@moh.gov.et	Ethiopia	01/05/2017	Online entrance examinations for general practitioners (GP)	Human resources for health
MRIS Federal Ministry of Health, dha@moh.gov.et	Ethiopia	01/07/2018	Multi sectoral report on HIV AIDS	Health information system
mHealth Federal Ministry of Health, dha@moh.gov.et	Ethiopia	01/01/2017	To create a two way communication among health workers like sharing documents, video, audio and educational messages, and questions and answers	Service delivery, Human resources for health
MFR Federal Ministry of Health, dha@moh.gov.et	Ethiopia	01/01/2017	Collects national health facility list, location, services, infrastructure and number of health professionals.	Service delivery
MEMS (Medical equipment management system) Federal Ministry of Health, dha@moh.gov.et	Ethiopia	01/03/2018	The objective of the system is to automate medical equipment management system of the country from procurement up to disposal.	Service delivery
Members system Federal Ministry of Health, dha@moh.gov.et	Ethiopia	01/12/2017	Detail information about insurance members	Healthcare Financing
Medicine Registration System Federal Ministry of Health, dha@moh.gov.et	Ethiopia	01/07/2017	Medicine registration, suspension and cancellation processes	Service delivery
KPI Survey Database Federal Ministry of Health, dha@moh.gov.et	Ethiopia	01/07/2012	The application is a data analysis tool that is used to measure staff and patient satisfaction.	Service delivery
IVR (Interactive voice response) Federal Ministry of Health, dha@moh.gov.et	Ethiopia	01/07/2014	To collect MNCH, PHEM, IRD, Stockout & death data from health post by health extension worker	Service delivery, Access to medicine, vaccine and Technology
Intellicare Federal Ministry of Health, dha@moh.gov.et	Ethiopia	01/05/2016	To capture the patient interaction in the facility, human resource and financial transactions	Service delivery

IFMIS - Integrated Financial Management Information System Federal Ministry of Health, dha@moh.gov.et	Ethiopia	01/09/2011	Used to manage the budgeting and expenditure of the ministries finance	Healthcare Financing
IBEX (Integrated Budget Expenditure System IBEX) Federal Ministry of Health, dha@moh.gov.et	Ethiopia	01/07/2018	Financial record and reports	Healthcare Financing
HRIS Licensing Federal Ministry of Health, dha@moh.gov.et	Ethiopia	01/10/2011	HRIS-License is a tool to collect, store and manage health professionals licenses.	Human resources for health
HMIS Brisk Federal Ministry of Health, dha@moh.gov.et	Ethiopia	01/07/2015	Patient registration, patient data collection, triage patient and department assignment, clinical data collection, specimens collection, payment and billing transaction and patient management, in short serve as full EMR	Service delivery, Health information system
Gx alert Federal Ministry of Health, dha@moh.gov.et	Ethiopia	01/10/2017	To do tasks related with viral load laboratory	Service delivery
Green Stone Digital library Federal Ministry of Health, dha@moh.gov.et	Ethiopia	01/01/2017	Enables to read and download different medical health electronic books subscribe journals	Service delivery, Human resources for health
Fleet management system Federal Ministry of Health, dha@moh.gov.et	Ethiopia	01/12/2016	To gather, store, process, monitor and report the movement of vehicles owned by the ministry	Service delivery
EPS pharmaceutical information systems (ARSMBDHS) Federal Ministry of Health, dha@moh.gov.et	Ethiopia	01/06/2017	Drug, medical equipment and laboratory reagents stock management system.	Access to medicines, vaccines and technology, Service delivery
eMRIS (Electronic Multi Sectoral Response Information System) Federal Ministry of Health, dha@moh.gov.et	Ethiopia	01/08/2016	Data and reporting for HIV related data	Health information system
EMR - ART Module Federal Ministry of Health, dha@moh.gov.et	Ethiopia	01/07/2018	For ART data registry and analysis.	Service delivery
eHMIS - Regions except SNNP Region Federal Ministry of Health, dha@moh.gov.et	Ethiopia	01/06/2012	For entry, analysis and reporting routine health data.	Health information system
EGBLS Federal Ministry of Health, dha@moh.gov.et	Ethiopia	01/07/2015	Equipment or property management	Service delivery

ECHMIS (Electronic Communication HMIS) Federal Ministry of Health, dha@moh.gov.et	Ethiopia	01/05/2017	Used to register inventory and maintain bin cards and stoke cards	Service delivery, Access to medicine, vaccine and technology
eCHIS - Old Federal Ministry of Health, dha@moh.gov.et	Ethiopia	01/01/2015	Rural community health information registration systems	Health information system,
eCHIS - New Federal Ministry of Health, dha@moh.gov.et	Ethiopia	01/07/2018	eCHIS is designed to facilitate the daily activities of the health extension worker. It serves as a job aid, point of service data collection, patient identification, and communication tool at the community and health post level.	Service delivery, Health information system
E&RIS (Emergency and referral information system) Federal Ministry of Health, dha@moh.gov.et	Ethiopia	01/06/2016	Service provided in facilities (government), referral networking and bed management	Service delivery
DSS (demographic survellance system) Federal Ministry of Health, dha@moh.gov.et	Ethiopia	01/03/2010	To support universities in generating longitudinal data on major demographic events and identify cause of death.	Health information system
DSS - Self Assement Tool Federal Ministry of Health, dha@moh.gov.et	Ethiopia	01/04/2013	Collect data for self-assessment	Human resources for health
DHMS (Digital Hospital Management System) Federal Ministry of Health, dha@moh.gov.et	Ethiopia	01/05/2016	Hospital activities card, lab, bed management, pharmacy, etc. it's like full EMR	Service delivery, Health information system
District Health information system (DHIS2) Federal Ministry of Health, dha@moh.gov.et	Ethiopia	01/05/2016	DHIS2 is a tool for collection, validation, analysis, presentation of aggregate and patient-based statistical data, tailored (but not limited) to integrated health information management activities. DHIS2 provides functions such as data entry tools, provides different kinds of validation tools, easy to use - one click reports, flexible and dynamic data analysis, easy to use metadata management, messaging interfaces for communications.	Health information system
DATIM (Data for Accountability Transparency & Impact Monitoring) Federal Ministry of Health, dha@moh.gov.et	Ethiopia	01/06/2015	Used to report HMIS data from ART Sites that are supported by CDC	Health information system

Dagu Federal Ministry of Health, dha@moh.gov.et	Ethiopia	01/01/2010	It is a Health Commodity Management Information System used to record new drugs (stock), calculate consumption rate, issue drugs to Units (departments) and manages inventory.	Access to medicine, vaccine and technology, Service delivery
Cnet Federal Ministry of Health, dha@moh.gov.et	Ethiopia	01/08/2012	Finance & store Management.	Service delivery
CMMS (Computerized Maintenance Management System) Federal Ministry of Health, dha@moh.gov.et	Ethiopia	01/12/2017	It is an electronic maintenance support system for medical equipment & office electronic equipment. The system registers all available medical equipment & manages inventory.	Service delivery
CCEIT Federal Ministry of Health, dha@moh.gov.et	Ethiopia	01/07/2017	Cold chain equipment inventory	Service delivery, Access to medicine, vaccine and technology
CBHI (community based health insurance system) Federal Ministry of Health, dha@moh.gov.et	Ethiopia	01/03/2016	Manage hospital's patient health care financing system & reporting to respective woredas that are included in the cbhi scheme.	Service delivery, Healthcare finance
APTS (auditable pharmaceuticals transactions system) Federal Ministry of Health, dha@moh.gov.et	Ethiopia	01/08/2016	Facilitating pharmacy payment, stock management, staff performance	Service delivery
Antroplus Federal Ministry of Health, dha@moh.gov.et	Ethiopia	01/07/2018	RNA, different nutrition analysis	Service delivery
AGHMC EMR Federal Ministry of Health, dha@moh.gov.et	Ethiopia	01/07/2018	It is an electronic Medical Records System (EMR) that records individual patients information from card (registration) to assigning to physicians for treatment and records and retrieves patients information where needed.	Service delivery, Health information system
ADR (report online) Federal Ministry of Health, dha@moh.gov.et	Ethiopia	01/07/2015	Used to report adverse drug event	Service delivery
Admission Referral Service Federal Ministry of Health, dha@moh.gov.et	Ethiopia	01/01/2017	admission, discharge, referral service in & out, elective surgery appointment, normal outpatient appointment	Service delivery

Ethiopia Health Data Analytics Platform (EHDAP) Dawit Kassa, dawit@zenysis.com	Ethiopia	01/01/2016	The Zenysis software integrates data from fragmented systems through an interoperability layer that uses data science techniques to harmonize the differences between integrated systems without requiring any modification to the systems themselves.<br><br>In Ethiopia, the Zenysis platform successfully integrated data from more than 15 fragmented systems for the first time. This includes data from three routine Health information systems as well as surveillance data, surveys, supply chain data, financial data, climatological data and more.<br><br>More than 600 million data points from these systems are now accessible for analysis through a single, easy-to-use platform. The platform has enabled a significant shift from low-resolution (regional-level) analytics to high-resolution (facility-level) analysis and decision-making. Decision-makers can now access continuously updated performance statistics for all 860+ districts on-demand.	Health information system
DHIS2 for COVID-19 Surveillance: Ethiopia Rebecca Potter, covid@dhis2.org	Ethiopia	08/05/2020	The DHIS2 digital data package for COVID-19 is designed to accelerate case detection, situation reporting, active surveillance and response in countries. The COVID-19 digital data package includes standard metadata aligned with the WHO's technical guidance on COVID-19 surveillance and has been adapted to local country context and language in this implementation.	Health information system

Life-saving Mobile Health Kits (LMHK) for Obstetric Care in Remote Areas Belay Haffa, belay_haffa@wvi.org	Ethiopia	01/07/2019	Supported by a grant from Born on Time, a public-private partnership that is implementing several interventions to support the prevention of preterm birth, World Vision Ethiopia engaged with technology partner SLK TECHLABS to deploy mobile health screening kits with an internet-driven telemedicine capability in the Amhara region. These mobile health kits include a mobile phone app and diagnostic equipment, including ultrasound, that captures key vitals and assesses pregnant women for risk factors of preterm birth and other obstetric complications. The kits are used by trained outreach health care providers who are also linked with enhanced telemedicine at referral hospitals. This enables timely management of any areas of concern and encourages families to seek skilled attendants for potential preterm births. The kits can also be moved easily from village to village, which maximizes access to these essential services for more women living in remote areas.	Service delivery, Health information system

Implementation of a LIMS based on GNU Health at the International Center for Medical Research of Franceville Armand Mpassy-Nzoumba, armandmpassy@yahoo.com	Gabon	01/10/2015	The system was implemented at the Medical Laboratory department. The purpose was to improve the overall efficiency of the department by: streamlining lab and billing processes, providing accuracy and timely delivery of lab services, improving management of patient data, improving revenues, reducing costs, improving reporting and statistics. It is operated by about 10 users. The following functions are fully integrated: Patient Management, Laboratory sample and test management, Reporting and statistics, Patient Billing and Financial Accounting, Health Insurance management, Patient Appointment management, Data exchange with the general accounting system of the whole organization. The system has been installed using the following platforms and technologies: OpenSuse Linux (server), Microsoft Windows 7/10 (clients), PostgreSQL (database), Tryton-ERP.	Service delivery
Suivi des malades diabétiques Mr Dupont, dupont58@gmail.com	Gabon	01/07/2019	Ce projet utilise les téléphones mobiles pour suivre et informer les malades diabétiques rattachés à un centre de santé	Service delivery

Real-time COVID-19 case monitoring for the MoH Gambia Aleksa Krolls, aleksa@openfn.org	Gambia	03/04/2020	Open Function is currently configuring a CommCare-to-DHIS2 integration on the OpenFn platform to enable real-time monitoring and analysis of COVID-19 cases for The Gambian Ministry of Health. The MoH Gambia is implementing Dimagi's template CommCare COVID-19 applications. Using OpenFn, CommCare data will be automatically cleaned and uploaded to DHIS2 for visualization and monitoring of COVID-19 cases nationwide. OpenFn is used by dozens of global health organizations worldwide, and has been used to facilitate disease monitoring and analysis at scale across the world. The OpenFn provides a secure and scalable infrastructure on which organizations can connect any technology and automate key workflows, including data cleaning, data validation, and duplicate-checking.	Health information system
Hello Nurse Darlene Irby, Darlene.Irby@jhpiego.org	Ghana	01/01/2017	Hello Nurse is a two chapter interactive story covering malaria prevention, diagnosis and case management for pre-service nurses. The MCSP Malaria Interactive Story App ('Hello Nurse') is part of a series of e-learning tools being used by USAID’s flagship Maternal and Child Survival Program (MCSP) to strengthen malaria health services, focused on the components around strengthening and building the capacity of pre-service institutions.	Service delivery, Human resources for health
Blood Safety Strengthening Programme: Blood Safety Information System (BSIS) Implementation Rhonwyn Cornell, rhonwyn.cornell@jembi.org	Ghana	03/10/2016	Safe blood is used to treat postpartum hemorrhage, childhood malaria, severe anemia, trauma and surgery. The BSSP develops and implements BSIS, an open source system designed to manage donor and donation information from the point of donation to transfusion. BSIS is for resource-limited blood services and supports AfSBT accreditation processes. Our implementations follows international good practice, building local	Service delivery

			capacity within the blood services to use, manage and maintain BSIS.	
The Safe Delivery App in Ghana Lauren Bellhouse, lauren@maternity.dk	Ghana	01/12/2017	The Safe Delivery App (SDA) for Ghana was formally launched in December 2017 in partnership with the Danish Embassy in Ghana and UNFPA Ghana, with the support of the Ghana Health Service and the Ministry of Health. Prior to the launch, partners worked to adapt the SDA to Ghanaian national standards and protocols. Targeted training enabling skilled birth attendants to use the SDA as an on-the-job aid for handling normal and complicated births is ongoing. The App has been integrated into trainings carried out by UNFPA, Ghana Health Service, CHAG, NORSAAC, and most recently a virtually training by the MOH.	Service delivery, Human resources for health
MNCH mobile content with nutrition focus, available in Twi, Ewe, Hausa and English in SMS and voice transcript format Kim Viljoen, kviljoen@gsma.com	Ghana	01/09/2013	Nutrition and MNCH mobile ready content for Ghana available in English, Twi, Ewe and Hausa. All content was produced by in-country specialists and is based on recommended MNCH and local nutrition practices. The Ministry of Health played a vital role in the development and content validation. The Content is available in the form of: topic-specific factsheets, SMSs and voice message transcripts.	Service delivery
DHIS2 for COVID-19 Surveillance: Gambia Rebecca Potter, covid@dhis2.org	Ghana	08/05/2020	The DHIS2 digital data package for COVID-19 is designed to accelerate case detection, situation reporting, active surveillance and response in countries. The COVID-19 digital data package includes standard metadata aligned with the WHO’s technical guidance on COVID-19 surveillance and has been adapted to local country context and language in this implementation.	Health information system

LBG Covid-19 Response Tang Simon, simon@literacybridge.org	Ghana	09/07/2020	With financial support from Amplio Network-USA, LBG partnered Ghana Health Service in April 2020 to launch the COVID-19 awareness campaign in eight (8) vulnerable districts in the Upper West region. In this region, there is an increased risk of the virus due to cross-border activities with Burkina Faso, as well as from migration within the country. Most big organizations shut down in March 2020 following the outbreak of the disease in Ghana. Consequently, our COVID-19 response is the main intervention reaching vulnerable communities. We are leveraging our relationships with key stakeholders, including district health directors, district assembly members, traditional authorities, religious leaders, and others to coordinate. Under this response project, LBG has conducted training and deployed Talking Books with COVID-19 content in four local languages across 207 Community-based Health Planning and Services zones/compounds and 78 community health volunteers.	Service delivery
MEDTRACK Omar Seidu Farouk, omar@medtrack.com.gh	Ghana	06/06/2017	MedTrack is an electronic health records platform that connects physicians to community pharmacies and diagnostics centres. The system integrates medical records into national digital identification numbers of citizens, making them portable and universally accessible by authorized providers. We are Africa’s first EHR management platform. Our mission is to bring electronic health records to 1 billion Africans using their national digital identities. MedTrack’s health records management eliminates fragmentation across different health facilities and serves as the building blocks for emerging healthcare innovations in telemedicine, Healthtech AI and Population Health Management tools.	Service delivery, Health information system

Carely Digital Health Platform Kelvin Ashie, healthdirectglobal@gmail.com	Ghana	04/05/2020	Our Technology Solution - Health Direct Global provides a multi-sided platform that allows patients and doctors to track medical records efficiently, schedule and manage appointments and have access to alternative healthcare financing for their healthcare needs through a diverse range of healthcare plans. Our multi-sided platform allows users to have on-demand access to healthcare through a network of health service providers and also a financially inclusive platform that allows users to save and spend towards their healthcare needs. Health Direct positions itself as a data-driven health-tech company that focuses on building the data engine and payment rails that will power the health industry in Africa for impact, and scale and provide a pathway for reverse innovation for shared value creation in the healthcare industry.	Service delivery

Global Health Security Agenda Pia MacDonald, pmacdonald@rti.org	Guinea	01/02/2015	Through the U.S. Centers for Disease Control and Prevention-funded Global Health Security Agenda (GHSA) project in Guinea (2015–2019), RTI International provides technical assistance to the Ministry of Health and Hygiene (MOH) to strengthen the disease surveillance system to better prevent, detect, and respond to outbreaks. RTI is providing assistance to the MOH to implement DHIS 2 for epidemiological disease surveillance nationwide including aggregate weekly reporting and case notifications.	Health information system
Suivi de la vaccination de routine des enfants de moins 5 ans Dr Souma, souma58@gmail.com	Guinea	27/07/2019	ce projet utilise le mobile pour suivre les enfants à vacciner en envoyer des messages de RV et de rappels aux mamans	Service delivery
Global Open Facility Registry (GOFR) Eileen Reynolds, ereynolds@rti.org	Guinea	01/07/2018	RTI International and JSI implemented the Global Open Facility Registry (GOFR) project in Guinea. GOFR's objective is to enable integrated and interoperable Master Facility Registries (MFR) by:<br>• Developing software facility reconciliation tools that strengthen interoperable MFRs.<br>• Supporting Ministries of Health in MFR practices.<br>Our accomplishments were:<br>• Contribution to the requirements for Guinea for the facility reconciliation tool and testing of the tool.<br>• Development of a facility registry management manual.<br>• Development of a DHIS2 account request form and process to strengthen management of user accounts on the national Health information system.<br>• Production of a report which analyzed data collected on health facilities using the national DHIS2.<br>• Training of the MoH ICT and SNIS teams on how to use the GOFR reconciliation tool to enhance interoperability	Health information system

			• Strengthening of DHIS 2 as the current repository of the facility registry by adding sub-districts to the organizational hierarchy.	
OpenLMIS Guinea Rebecca Alban, info@openlmis.org	Guinea	01/08/2018	The GHSC-PSM project provided the assistance needed to implement a new e-LMIS, including the development of a roll-out plan for the automation process detailing roles, responsibilities, and commitments of each stakeholder. The roll-out team entered all LMIS data, developed training materials, created a pool of trainers, and organized training workshops in regions. Meanwhile, the Guinea MoH set up a governance body for the OpenLMIS, including a steering committee and a multidisciplinary technical team with representatives of DNPM, Bureau de Stratégie et de Dévelopement (BSD), the Information and Communication Technology (ICT) unit and Pharmacie Centrale de Guinee (PCG), USAID, GHSC-PSM, Catholic Relief Services (CRS), and the World Health Organization (WHO).	Access to medicine, vaccine and technology, Service delivery
Guinea EVD contact Tracing Anne Lui, Aboubacar DIALLO, Mamady Camara, adiallo84x@gmail.com	Guinea	15/10/2014	An informatics system consisting of a mobile health application and business intelligence software was used for collecting and analyzing Ebola contact tracing data. This system offered potential to improve data access and quality to support evidence-based decision making for the Ebola response in Guinea. Implementation challenges included software limitations, technical literacy of users, coordination among partners, government capacity for data utilization, and data privacy concerns.	Health information system
DHIS2 for COVID-19 Surveillance: Guinea Rebecca Potter, covid@dhis2.org	Guinea	08/05/2020	The DHIS2 digital data package for COVID-19 is designed to accelerate case detection, situation reporting, active surveillance, and response in countries. The COVID-19 digital data package includes standard metadata aligned with the WHO's technical guidance on COVID-19 surveillance and has been adapted to local	Health information system

			country context and language in this implementation.	
DHIS2 for COVID-19 Surveillance: Guinea Bissau Rebecca Potter, covid@dhis2.org	Guinea-Bissau	08/05/2020	The DHIS2 digital data package for COVID-19 is designed to accelerate case detection, situation reporting, active surveillance, and response in countries. The COVID-19 digital data package includes standard metadata aligned with the WHO's technical guidance on COVID-19 surveillance and has been adapted to local country context and language in this implementation.	Health information system
Hoji Mobile Data Collection and Analysis Platform Gitahi Ng'ang'a, gitahi@hoji.co.ke	Kenya	01/03/2016	Hoji is an easy to use mobile data collection and analysis platform for surveys, M&E and DQAs. It enables health professionals to easily develop and deploy digital data collection and analysis solutions at scale. Field workers collect and submit data to an online web server in real-time. They may also collect data while offline. The server then runs detailed real-time analysis, enabling managers to make evidence-based decisions, optimize interventions and maximize impact.	Health information system
cStock/DHIS2 Zoya Mohammed, zoya_mohamed@insupplyhealth.com	Kenya	01/02/2017	The purpose of this project is to scale up the cStock approach (first implemented in Malawi) in Kenya using DHIS2 as the technology platform. In Malawi cStock was successful and continues to be an integral part of the community health program however the technology is not easily transferable to new contexts. The pilot in Kenya will test the applicability of this approach in another context and develop a tool and supply chain model that can be used by community health supply chains globally.	Service delivery, Access to medicine, vaccine and Technology
personal women's health advice via mobile chat MacGregor, Mac@lily.health	Kenya	31/05/2017	The technology aims to provide women: 1. Expert reproductive health advice 2. Easily accessible on all mobile phones 2.Private messaging format that is comfortable to use	Service delivery

Scaling of BLIS(Basic Laboratory Information System) 3.0 Implementation Across Africa Emmanuel Kweyu, ekweyu@strathmore.edu	Kenya	01/01/2014	The (BLIS) project, is an open source mobile and Web-based system that can be installed in a local, district, or national laboratory. It standardizes data collection, track the specimen/test workflow and improve the ability to generate useful reports and can both give a realistic picture of laboratory services and assist with staff and budget planning. BLIS has incrementally improved based on the user feedback and now is moving to the new release of Ver. 3.0.	Service delivery
Medic App in Siaya County in partnership with Ministry of Health Michael Korir, korir@medicmobile.org	Kenya	03/08/2020	In 2017, County Government of Siaya and Medic Mobile partnered to implement the CHT to support Siaya's Community Health Worker (CHW) program. Since then the program has grown to support more than 2,217 CHWs providing integrated care for pregnant women and children including immunization,Integrated Community Case Management (iCCM) and malnutrition support, family planning, eye care, community based disease surveillance and HIV testing and referrals.	Service delivery
M-TIBA mobile health wallet Maarten Ras, m.ras@carepay.co.ke	Kenya	01/09/2016	M-TIBA is a digital health payment platform that directly connects patients, payers and healthcare providers and exchanges money and data between them. M-TIBA is built on Safaricom's revolutionary M-PESA mobile payment platform, with a front-end mobile health wallet that can empower everyone with a mobile phone to make informed decisions about their healthcare. The wallet facilitates savings, remittances, low-cost insurance and donations for the health of individuals and families.	Healthcare Financing

Connected Diagnostics for malaria Tobias Rinke de Wit, t.rinkedewit@pharmaccess.org	Kenya	01/10/2017	Connected Diagnostics (ConnDx) is an innovative, cost-efficient way to revolutionize the diagnosis and financing of malaria treatment. If treatment is targeted only at patients who have tested positive, resources can be spent more effectively to save lives and unnecessary prescriptions avoided. Capitalizing on the mobile revolution, ConnDx makes payments for treatment conditional on positive tests, using low-cost diagnostics, cloud-based technology, and the M-TIBA mobile health wallet.	Healthcare FInancing
Digital Cash Advance for the health sector Tom Bouma, t.bouma@medicalcreditfund.org	Kenya	01/01/2017	Medical Credit Fund's Digital Cash Advance is expanding mobile lending into the Kenyan health sector. The Digital Cash Advance is a short-term loan facility that uses the digital revenues of health SMEs to secure and repay uncollateralized loans. In a sector where it can be difficult to secure a regular bank loan, it provides a fast digital financing solution. As of December 2018, over 900 loans totaling $2.6 million have been disbursed to 200 borrowers, with a 97% repayment rate.	Healthcare financing
Regional Action Through Data (RAD) Laurie Markle, LMarkle@brhc.com	Kenya	26/09/2018	Ensure continuity of care in immunization for children within the population along the selected cross-border areas, utilizing an electronic Health information system based on individual data. Main users are clients (children accompanied by mother/fathers and guardians) and providers. the solution is currently being implemented along the borders of Uganda and Kenya, with another pair of countries to be selected for the next phase by the IGAD Member Countries.	Service delivery, Health information system
Malaria Test Tim Nichols, tim.nichols@vitalwave.com	Kenya	06/03/2019	Testing the DSME space	Service delivery

SmartHealth App in Kenya Nii Amon Dsane, ndsane@livinggoods.org	Kenya	01/09/2016	Living Goods and Medic Mobile have collaborated to create a powerful set of mobile and web tools to support the Community Health Workers (CHW's) assisted by Living Goods.Built on Community Health Toolkit Framework core, the open-source Smart Health app is used by CHWs to support delivery of high quality and integrated primary health care services, including common childhood diseases such as malaria, diarrhea and pneumonia; maternal and neonatal care,family planning and immunization referral services. Although Living Goods independently configured its immunization and COVID-19 workflows, it still actively collaborates with Medic Mobile for ongoing technical support, including needs assessments, technology strategy and upgrades. This application also has a supervisor dashboard that enables the real-time remote management of CHW's, also helps manage effective stocking of medical commodities, and is being used to provide the government with critical data to better plan and budget for community-level interventions	Service delivery
MNCH mobile content with nutrition focus, available in Swahili and English in SMS and voice transcript format Kim Viljoen, kviljoen@gsma.com	Kenya	01/09/2013	The GSMA, together with its global content consortium lead by CABI, developed open-source nutrition and MNCH mobile ready content for Kenya available in Swahili and English. All content was produced by in-country specialists and is based on recommended MNCH and local nutrition practices. The Ministry of Health played a vital role in the development and content validation. The Content is available in the form of: topic-specific factsheets, SMSs and voice message transcripts.	Service delivery

Keheala - Digital adherence technology that uses behavioral 'nudges' and basic feature phones to iA139:J139mprove health outcomes. Jon Rathauser, jonr@keheala.com	Kenya	01/04/2018	Keheala is a digital health platform that delivers behavioral interventions across basic feature phones and smartphones in order to improve healthcare access and treatment outcomes for patients in the developing world. Keheala addresses the non-medical drivers of disease - stigma, a lack of information, motivation and support - with demonstrated behavioral strategies from the social sciences (behavioral economics and psychology).<br>In partnership with the U.S. Agency for International Development (USAID), the Kenyan Ministry of Health and academic partners at MIT, Keheala implemented a 1200 tuberculosis patient randomized controlled trial in Kenya. Patients using the Keheala intervention demonstrated a 68% reduction in the unsuccessful treatment outcomes - death, failed treatment and loss to follow up - compared to the standard of care control group. These results were recently published by the New England Journal of Medicine.	Service delivery
Medic App in Elgeyo Marakwet Susan Maigua, susan@medicmobile.org	Kenya	06/01/2017	The project seeks to improve access to critical health services and overall health for underserved, women, and children in Elgeyo Marakwet County through education, community training, and the provision of tools and other services to the Ministry of Health and staff. Leveraging mobile tools and other tools, we will increase ANC and immunization coverage, increase the Service delivery points (SDP) for basic emergency obstetric and newborn care (BeMONC) and family planning (FP) commodities, improve FP uptake, and strengthen health Service delivery on ANC, immunization, delivery, PNC, and service reporting.	Service delivery

Digital African Health Library Dr Bruce Dahlman, director@digitalhealthlibrary.net	Kenya	01/04/2016	It is a smartphone based digital library membership service that brings to clinicians the benefit of a one-stop clinical decision support application with 50+ evidence-based resources within a single search to answer the clinical care questions that are prompted by patients at the 'point of care'. The Digital African Health Library is powered by Indextra, the leading mobile library platform for both smartphone or tablet; on Android and Apple's iOS. Within each collaborating country, the Digital Library aims to include Ministry of Health guidelines and newsletters, local medical journals as well as the core handbooks from Oxford, Cambridge, Wiley and British National Forumulary Publishers.	Service delivery
Improving Healthcare Capacity and Access Using THINKMD's Clinical Decision Support Tool Used by Private Healthcare Providers Meg McLauhlin, mmclaughlin@thinkmd.org	Kenya	01/05/2019	THINKMD's platform will be implemented by private health care providers working at local pharmacy kiosks and private healthcare clinics within the Kibera slums of Nairobi, Kenya. In a one-year implementation, private healthcare providers will utilize THINKMD's platform to triage and treat patients. Pre/post analysis will be completed to better understand the impact of using THINKMD's platform and will consider: quality of care; healthcare capacity and skills; adherence to protocols; monitoring of drug administration; monitoring of medicine inventory; appropriate treatment; patient satisfaction; health seeking behavior; knowledge of danger signs; as well as other measures helping to identifiy both health and economic impact. Population health data will be captured throughout the implementation and be used to help monitor public health and the potential for outbreaks.	Service delivery

Safe Abortion App Sarah Shannon, Executive Director, SJHOLCOMBE@gmail.COM	Kenya	01/01/2018	This app provides comprehensive information on safe abortion that can be used alone or shared by women, healthcare workers and women's health advocates. Once downloaded, the app operates offline, without a data plan or internet access. By meeting women's needs for reliable, understandable and actionable information, this app can help prevent the harm caused by unsafe abortion and restrictions on women's access to reproductive health. The app provides a pregnancy calculator; a comparison of methods; explicit instructions and a description of what to expect; information on danger signs; and an extensive set of FAQ.	Service delivery
Empower Health Molly Guy, molly.guy@medtronic.com	Kenya	01/02/2018	The goal of Empower Health is to improve disease awareness, reduce the burden of disease, and improve the efficiency of managing hypertension and diabetes for both patients and clinicians. The Empower Health model consists of a mobile tablet, an automated blood pressure (BP) machine, a glucometer, and a novel proprietary software application—combined in a unique platform for efficient screening and longitudinal management of a patient cohort. Leveraging the model, physicians provide patients with a tailored hypertension and/or diabetes management plan. Patients can access regular BP and blood glucose checks at community-partner locations where they receive real-time feedback on their measurement. On the mobile application, clinicians can view patient data, provide direct patient feedback on their condition via SMS, and write electronic prescriptions—accessible through participating pharmacies.	Service delivery

M-Sawazisha: Integrating Mobile Technology in MNCH in Afya Jijini Michael Korir, korir@medicmobile.org	Kenya	12/03/2018	M-Sawazisha will help community health volunteers register new pregnancies, track ANC visits, flag danger signs, report on deliveries, report on post-partum visits, report on postpartum family planning and ask questions about pregnancy and family planning at the household level. The project will be implemented in the Nairobi slums (Mathare area) and a list of project facilities will be confirmed and provided by HealthRight International. Twenty-five CHVs will be selected from key catchment areas for each of the possible four health facilities in the project area. The 6-month project will train a total of 100 CHVs to offer services in these areas, and the general project will also use CHVs to deliver Nutrition and WASH messages at the community level.	Health information system, Service delivery
PROMPTS - Promoting Mothers during Pregnancy through SMS Rachel M. Jones, Rjones@jacarandahealth.org	Kenya	01/05/2018	PROMPTS is a text messaging platform that empowers mothers to seek care at the right time and place. PROMPTS is a free program that provides pregnant women and new mothers in public health systems with a carefully designed sequence of behavioral nudge messages , and an AI-supported helpdesk service that triages responses and questions to flag for clinical urgency.<br>Key program results:<br>• 150,000+ mothers have enrolled in platform in less than a year from over 250 public hospitals<br>• 32,000+ questions have been answered within 24 hours.<br>• Facilities with PROMPTS have a 20% increase in mothers completing 4 ANC visits compared to equivalent non-participating facilities.<br>• A randomized controlled trial (RCT) conducted with 900 women seeking care at public facilities demonstrated when pregnant women or new mothers receive our SMS messages, they are 22% more likely to seek	Service delivery

			advice for their negative health symptoms (p=0.07; 90% CI), and they are 1.6x more likely to take up family planning.	

Medkit.Network Dr. Louis Somoni Machogu, dr.somoni@gmail.com	Kenya	26/04/2020	Aggregator of Pharmacy professionals and their pharmacies, linking them with patients, their clinicians and tracking availability & quality assurance for Pharmaceutical services, Health Products and technologies. Vision is to have all pharmacies communicating on the free medkit.network HMIS/POS/ERP/Inventory Management infrastructure offering sustainability JIT on supply chain; and patient data portability to aid telemedicine & epharmacy services. Currently servicing the needs of Kenyans under quarantine, Covid 19 stigma & home based care with their e - prescription, needs as well delivering medicines to parents and loved ones to Kenyans living in diaspora as well as those in country. We have served clients paying cash, via insurance or even seeking medicines through public facilities. This ensures compliance to reduced movement and risk of contact with the corinavirus by both the patients living with a chronic ailment and the healthcare workers working in the pharmacies.	Service delivery, Access to medicine, vaccine and Technology

SASAdoctor Telemedicine Android App Francis Osiemo, francis.osiemo@sasadoctor.com	Kenya	05/02/2018	SASAdoctor Virtual Medical Care is an exciting new medical service which provides accessible and affordable high-quality medical care to Kenyans. SASAdoctor allows patients to consult with Kenyan trained and supervised medical doctors and clinical officers on an Android App via live video conferencing. This live face to face medical consultation will result in an assessment, diagnosis and if necessary, a signed prescription; a requisition for investigation; a signed sick note or a referral to a specialist. All these documents will be delivered right into the App on your phone. Every Kenyan will now be able to carry their entire personal medical record, maintained by a medical professional, in their SASAdoctor App on their device. Key features of SASAdoctor Virtual Medical Care includes: • Affordability: Consultation as low as $5 and free for those with insurance. •Kenya Wide Coverage •Personal Medical Record •Good Medicine • Available 24/7 •Medication Delivery • mLab services	Service delivery
Kenya Quality Model For Health e-tool Steven Wanyee, swanyee@intellisoftkenya.com	Kenya	01/01/2018	The Kenya Quality Model for Health is defined as a conceptual framework for guiding and supporting coordination in an integrated manner, efforts, and investments aimed at improving the quality of healthcare in Kenya. The eKQMH tool aims at providing health facilities with IT-based tools to support continuous quality improvement initiatives in line with the KQMH standard.	Service delivery
Bethany Kids Kijabe Hospital Steven Wanyee, swanyee@intellisoftkenya.com	Kenya	01/06/2015	This is an OpenMRS based system. The purpose of the of the system was upgrade data managemnet elements which include efficiency in data management in terms of secure data storage, improved data capture methods, and provision of proper tools for data analysis to support provision of care.	Health information system, Service delivery

Kenya Health & Empowerment Wade Munday, wmunday@addisclinic.org	Kenya	01/05/2020	The project uses asynchronous telemedicine to drive quality improvement in rural health settings and create equal access to digital health innovations for frontline health workers in Kenya. Local providers are given free access to a telemedicine platform and a global network of volunteer physicians who assist in the care of patients remotely and in less than 12 hours. The Addis Clinic staff in Kenya coordinate quality improvement through remote physician support, technology training and technical assistance, and continuing medical education.	Service delivery
e-Hospital Steven Wanyee, swanyee@intellisoftkenya.com	Kenya	01/08/2017	eHospital is an end-to-end hospital information system that integrates medical records system, lab system, and ERP in a distribution that addresses the workflows, forms, and reports of the East African market. In addition to being an end-to-end facility system which captures fundamental patient touchpoints, eHospital is a distribution of Bahmni. This essentially means that eHospital leverages the software excellence and technical architecture of a globally proven system backed by a strong community committed to the continuous development of the product.	Service delivery

COVID-19 SMS Data Collaboration Initiative Boris Maguire, boris@echomobile.org	Kenya	07/05/2020	COVID is having huge social and economic effects on Kenya's poor, and ground-level info is needed for data-driven solutions. We have partnered to collect this data by directly engaging 1M low-income Kenyans via interactive SMS. Social orgs across Kenya use Echo's platform to engage over 2M people monthly. These trusted connections present a unique opportunity to understand the impacts of COVID on the poor; in real time and at unparalleled scale. We propose to realize this opportunity by leveraging these connections. We are rallying Echo users to continuously engage their audiences with a standardized SMS data collection tool. To inform data collection content and ensure results feed practical action, Echo has partnered with the Population Council, a partner to Kenya's COVID Task Force and CDC Kenya.Together, we can provide unparalleled longitudinal insights on Kenya's most vulnerable communities, making targeted, data-driven action possible. But we need and are seeking funding to achieve this.	Health information system
DHIS2 for COVID-19 Surveillance: Kenya Rebecca Potter, covid@dhis2.org	Kenya	08/05/2020	The DHIS2 digital data package for COVID-19 is designed to accelerate case detection, situation reporting, active surveillance and response in countries. The COVID-19 digital data package includes standard metadata aligned with the WHO's technical guidance on COVID-19 surveillance and has been adapted to local country context and language in this implementation.	Health information system

Mobile Solutions for Women and Children's Health (Mobile WACh) Keshet Ronen, keshet@uw.edu	Kenya	22/05/2020	Mobile WACh is an open-source human-computer hybrid SMS messaging platform. Mobile WACh sends pre-composed messages to clients at scheduled times. Clients may send messages at any time and a live healthcare worker (HCW) answers incoming messages, leading to personalized dialogue. The internet-based HCW interface contains a dashboard designed to facilitate HCW management of tasks such as message response, patient tracking, and message translation into English. The system back-end collects SMS metadata such as time stamps and delivery status, as well as data on seminal events such as childbirth. To date, Mobile WACh has been implemented in 8 studies (including 4 randomized controlled trials, RCTs) targeting a range of reproductive, maternal, neonatal and child health outcomes. Scheduled intervention messages were developed for each study based on formative research with the target population and behavioral theory selected based on the target outcome.	Service delivery

DawaSure francis munyeki, munyekif@cadcreations.co.ke	Kenya	05/06/2020	Clinical Managing long-term illness and chronic conditions requires a continuous monthly supply of medicines, for example hypertension; diabetes, for a vast majority of the aged population without medical insurance these drugs depend on sponsor relatives (sons, daughters) to purchase. Due to this additional financial burden, the sponsor has to make difficult decisions each month spending on ongoing financial pressures they themselves are having. Therefore, they tend to shop around for medicines depending on the price. This often results in purchases of variants, equivalents of the prescribed medicine supplied ranging from different manufacturer, dosage, active components, derivatives and sometimes outright fakes, at the time of purchase. Dawasure works to the benefit of the patient by ensuring a regular and continuous supply of the right drugs each month. Dawasure works by creating a direct link between the patient and the manufacturer through the regular supply chain.	Service delivery, Access to medicine, vaccine and Technology
Living Goods- Community Health Innovation Network (CHIN) Fransicah Nzanga, fnzanga@livinggoods.org	Kenya	29/06/2020	CHIN's goal is to promote creative technology solutions that enable community health care to more seamlessly integrate with health facilities, while extending high-quality diagnostic technologies that reach clients at the community level. Initial innovations were: 1) a Closed Loop Referral system to digitally verify all CHW patient referrals where facility-based care took place, 2) Improved tech protocols for distributing HIV self-tests via CHWs, and 3) Predictive Analytics to identify and predict which households are most in need of medical attention. Current focus is Client Initiated Health Assessment (CIHA); a home-based self-triage assessment platform to guide patients and caregivers through an initial health screening and care-seeking journey directly linking them digitally to their local CHW. Implemented by	Service delivery

			Living Goods, Medic Mobile and THINKMD, CIHA seeks to investigate community level uptake of an integrated mHealth system that enables clients to more effectively request care and support.	
Lwala Community Alliance x OpenFn: Building a real-time decision support tool to strengthen healthcare in western Kenya Aleksa Krolls, aleksa@openfn.org	Kenya	01/01/2017	OpenFn powers Lwala's connected Health information system, integrating its CommCare case management CHW app with a central Salesforce-based patient database and automating secure, two-way information exchange for real-time monitoring and CHW decision support.	Service delivery
COVID-19 Service delivery Tracker Liz McNeil, lmcneil@jacarandahealth.org	Kenya	17/07/2020	Jacaranda Health partners with County governments to deploy low-cost, innovative solutions to the public health system in Kenya, to improve maternal and newborn health outcomes. In this project, we use our program tools to collate information from mothers about their experiences in facilities, and from providers about their challenges and performance. We have created a simple dashboard that is shared with county and facility managers to make more informed decisions. In the wake of the COVID-19 response, we have added the Service delivery challenges tracker where providers can update challenges in real-time to alert health system administrators. It is a simple map tool where users can see the status of individual facilities and the details of the reported challenges. Facilities are labeled as Red for significant challenges or Service delivery disruptions, Yellow for some challenges or Service delivery disruptions, and Green for no challenges or Service delivery disruptions.	Service delivery

KenyaEMR Pascal Mwele, Pascal.Mwele@thepalladiumgroup.com	Kenya	15/07/2020	KenyaEMR is a tailored distribution of OpenMRS which was developed by I-TECH (2012) but is currently being supported by The Palladium Group since 2016 under KeHMIS II Project. KenyaEMR is key towards aiding monitoring UNAIDS goals through; •Registrations & Enrolments: HTS, ART, MCH, TB/HIV, PrEP, KP, OVC, Adolescents & Young adults. •Triage: Vital signs, nutrition, and obstetric history •HIV Prevention: Key populations, PrEP, eHTS and PMTCT •HIV Treatment: Clinical workflow management; TB/HIV management; Patient management planning; and Differentiated care management and 0utcome documentation •MCH Services: ANC, L&D, PNC, HEI, FP and immunization services •Strategic Information: Line lists and reports (for Quality, Indicators, Surveillance, outcome, program and Ad hoc needs); Registers; Dashboards and Charts. Interoperability: with but not limited to; ADT (pharmacy management), T4A & Ushauri (appointment management), mLab & EID/VL (lab management), DHIS (automated indicator reporting) and 3PM (Key populations)	Health information system, Service delivery

National Data Warehouse (NDWH) Pascal Mwele, pascal.mwele@thepalladiumgroup.com	Kenya	16/07/2020	The NDWH is both an analytics platform and a repository. As a national repository, NDWH hosts de-identified data to facilitate programmatic monitoring of Health information systems (HIS) implementations and progress on clinical care and outcomes for HIV infected and exposed persons. The primary source of data is the EMR deployed at facility level. As an analytics platform, the NDW supports the visualization of the progress towards UNAIDs 90:90:90 goals, case based surveillance, the tracking of metrics relating to Human resources for health (HRH), the Monitoring of performance for different program indicators in the HIV scope and supports ad hoc data analysis for verified users to support further data analysis needs required. Currently the NDW hosts data for over 2.2 million HIV positive persons and over 1.1M testing records for tested individuals from 8 EMRs in approximately 1200 active facilities.	Health information system
Interoperability Layer Pascal Mwele, pascal.mwele@thepalladiumgroup.com	Kenya	26/08/2020	Interoperability Layer (IL) is a digital solution developed to orchestrate and broker data exchange across disparate systems at the facility level for care coordination and to higher levels for reporting and decision support. IL supports and enforces different standards through a channel configuration module. Currently, IL has a total of 7 participating systems in the country namely, Text for adherence (T4A)- which uses SMS/USSD for monitoring Adherence to ARVs and reporting problems, Ushauri - an automated appointment diary with a defaulter tracing module that ensures patients are seen and followed up in the shortest time possible, Web Antiretroviral Dispensing Tool (Web ADT), mobile Laboratory (mLab) which uses SMS for sending lab results from the national laboratories to a dedicated facility phone, Kenya's DHIS2, and IQCare and KenyaEMR.	Health information system

DWAPI Pascal Mwele, pascal.mwele@thepalladiumgroup.com	Kenya	26/08/2020	Also known as data warehouse upload tool, DWAPI is a multiplatform solution that is used to extract data from EMR databases in a defined extract format and transmit the encrypted extract to an API at national data warehouse staging area for further processing.	Health information system
Afya Mobile Pascal Mwele, pascal.mwele@thepalladiumgroup.com	Kenya	26/08/2020	An android based mobile application is used to capture HIV related data and specifically HIV Testing Services data. The application enables providers in health facilities and outreach services in the community to capture client demographics and fill HTS defined forms. The app is key in achieving community solutions towards reduction and finally elimination of the HIV pandemic	Service delivery, Health information system
ENDCORONAVIRUS.CO.KE Katherine Piets, kat@every1mobile.com	Kenya	09/03/2020	Every1Mobile self funded our END CORONAVIRUS KENYA project, a mobile-friendly, low data website in Kiswahili that provides critical handwashing guidance and easy access to accredited information on the COVID-19 disease from the World Health Organisation, local helplines and daily statistics. It contains the most important information about the virus and how users can protect themselves and their families, offering a series of interactive features to improve knowledge, attitudes and behaviours.<br><br>The site is deployed using Every1Mobile's own existing social platform software, which is accessible through the most basic web-enabled feature phones, and is designed to consume as little data as possible. This means that we are able to reach anyone with minimal data coverage and access to any basic handset. Content is regularly updated using authoritative sources and presented in colloquial language, to ensure users have the most recent facts and guidance, and to reinforce attitudes and behaviours.	Service delivery

m-Jali Caroline Mbindyo, caroline.mbindyo@mref.org	Kenya	21/09/2020	A data collection tool used in the following intervention areas CBHMIS/Diarrhoea Triage tool/Socio Economic Mapping,Tool based on World Bank, Data Visualisation, Household Mapping, Geo-Tagging, National Safety,Net Program, Malaria Net Distribution, Malaria Pill,Distribution, Covid-19 Community Surveillance Module, NCD,triage tool , Referral tool	Health information system
Case Management Mobile App for Children with Disabilities Jessica Charles, jessica@kupenda.org	Kenya	22/12/2020	In partnership with Kuhenza, MIT, Boston University, Dimagi and Sabrams Consulting, Kupenda for the Children (www.kupenda.org) developed mobile app to support community-based care and Covid-19 prevention for children with disabilities. This app enables NGOs to track the physical, social, academic and emotional health of children with disabilities, based on Measure Evaluation's Child Status Index. The app also includes a reporting form that local leader child advocates use to document their child outreach efforts after completing a disability training program. Through the app leaders also access resources for family counseling (i.e., Service Referral Guide, Home Visit Guide, Disability Guidebook, Disability Law Guide). Based on the leaders' reports, NGO staff provide support to children who show signs of malnutrition, illness, Covid-19, or abuse. We are also integrating the app with GIS mapping software that shows where the children live and helps connect them to services.	Service delivery, Health information system
Ohospital Daniel Kimani, suhadetechkenya@gmail.com	Kenya	10/03/2020	Ohospital is a digital telehealth platform connecting patients to health providers. We use Software as a service, mobile app, and USSD to deliver the services. We are in our early stages and growing with about 33 doctors working with us.	Service delivery

PaperEMR Pratap Kumar, pratap@health-e-net.org	Kenya	09/11/2021	PaperEMR is a solution for digital data to be captured directly from paper, by simply taking a picture!<br>Healthcare documentation is commonly done on paper. Going "paper-less" is expensive and complex for most organisations. PaperEMR enables organisations, with or without other digital systems, to use paper-based tools and workflows to generate high-quality digital data.<br>Existing paper-based tools are slightly modified for digital data capture. Information entered on paper is captured by taking a picture on any device with a browser and camera, including feature phones. Data is captured directly on the device, reviewed by the user, and shared to the cloud either over the internet or by SMS. PaperEMR unlocks a variety of possibilities, including simplified M&E, operational improvements (faster availability and use of data), and delivery of novel services, including telemedicine and referral management.	Service delivery, Health information system
DHIS2 MoH Data Warehouse Tsele Moloeli, moloelit@yahoo.com	Lesotho	16/06/2020	DHIS2 aims at capturing of weekly, monthly and quarterly aggregate reports and event based data capturing from all health facilities and hospitals for the benefit of reporting to Ministry of Health so that analysis of data may be done from a warehouse perspective that eliminates data silos. The implementation is fully fleshed, more additions however of data sets and indicators are constantly being added on request and modifications	Health information system
Lesotho eRegister Monaheng Maoeng, monaheng.maoeng@gov.ls	Lesotho	05/01/2018	eRegister aims at digitizing the HIV ART card and TB cab in order to produce a digital TB and ART register and to report to DHIS2 Presumptive TB Case quarterly report and the monthly HIV Care and treatment report. This eRegister when fully functional and rolled out country wide with a shared health records layer	Service delivery, Health information system

			across facilities, it will then develop into a fully fledged Electronic Medical Records system.	
HIV and Health Situation Rooms Masebeo Koto, kotomasebeo@gmail.com	Lesotho	06/01/2018	The HIV and Health Situation Room seeks to address the analysis of HIV situation using cutting edge analytics technology with rich displays and elaborate multiple chat analysis to advice high Government officials on action plans. currently it focuses on HIV, PMTCT and it will grow into a full Health situation room. It was launched officially on the 8th May 2018.	Health information system
Blood Safety Strengthening Programme: Blood Safety Information System (BSIS) Implementation Rhonwyn Cornell, rhonwyn.cornell@jembi.org	Lesotho	01/12/2015	Safe blood is used to treat postpartum haemorrhage, childhood malaria, severe anemia, trauma and surgery. The BSSP develops and implements BSIS, an open source system designed to manage donor and donation information from the point of donation to transfusion. BSIS is for resource-limited blood services and supports AfSBT accreditation processes. Our implementations follows international good practice, building local capacity within the blood services to use, manage and maintain BSIS.	Service delivery
Lab Information System Malebanye Lerotholi, 4malbe@gmail.com	Lesotho	01/01/2014	This is a national lab information system for capturing lab results and giving back feedback.	Service delivery
SMS Notification System Tsele Moloeli, moloet@yahoo.com	Lesotho	01/11/2017	Sms notifications for urgent lab tests results like EID, Viral load etc. Clients are notified to report to their respective health facility to pick the results which shall be viewed online by the clinician upon arrival or before.	Service delivery
Electronic Logistics Management and Inventory Management System Monaheng Maoeng, monaheng.maoeng@gov.ls	Lesotho	10/11/2020	Manages the pharmacy store rooms logistics and inventory	Service delivery, Access to medicine, vaccine and technology

CITIZEN APP Pule Phafane, pule@smartmycompany.com	Lesotho	15/06/2020	CITIZEN APP is a complete Outbreak Management System for COVID-19 that can assist various spheres Government in their efforts to stop the virus spread. The main features are:<br>1. Virus Tracking & Quarantine Enforcement - Tracks movement of citizens and patients<br>2. Contact Tracing – Real-time outbreak tracking by Country, District, Village/Town and Street and building.<br>3. Social Distancing Infection Risk Alert – Alerts users when they are or have been in an area with infection risk levels.<br>4. Queue Management - allows testing stations, NGO's, Government and business to manage appointments, scheduling through in-app digital queening.<br>5. Surveys - Conduct and capture real time surveys and additional statistical information to help Government make informed decisions.<br>6. Market Place – An in-app e-commerce platform that extents economic trading on medical supplies and other products and services.	Service delivery, Health information system
DHIS2-Covid -19 surveillance -Lesotho Monaheng Maoeng, monaheng.maoeng@gov.ls	Lesotho	15/06/2020	This project aims at managing covid19 patients and to provide statistics required for reporting on covid19. Tracker capture app is mainly used to track covid19 suspects and contacts. Stages involved are 1. screening 2. contact monitoring 3. case investigation 4. case monitoring 5. contact tracing. Alerts are also captured using data entry app per facility nationally.	Health information system

Bophelo-Ka-Mosebeletsi (Village Healthcare Workers) Application Teboho Khoali, teboho.khoali@undp.org	Lesotho	16/06/2020	Village Healthcare Workers (VHW) is a Lesotho's primary and a community-based healthcare unit comprising of community members who volunteered to support and provide linkages to the Ministry of Health healthcare system by undertaking community health related tasks. The unit has been more mandatory during the COVID-19 pandemic through execution of awareness campaigns in communities, monitoring individuals in home quarantine and reporting suspects of COVID-19 who have international travel history or symptoms to healthcare facilities and authorities. Since VHW unit used paper-based system from its origin, inefficiency and handling of bulk data challenges were being realised, and reports would be submitted after a week or a month time to the facilities. To eradicate this challenges, UNDP Lesotho Accelerator Lab developed a system which collects COVID-19 related data in communities and sends reports to healthcare centers and ministry's management in real-time for quick turnarounds in strategic decision making.	Health information system
EADEL - EGPAF Analytics Data Engine Lesotho Sele Mthimkhulu, smthimkhulu@pedaids.org	Lesotho	24/06/2020	Data capturing and reporting for EGPAF Lesotho, used by Records Assistants, SI&E Officers and Advisors, better reporting.	Health information system
Client Tracking Application Sele Mthimkhulu, smthimmkhulu@pedaids.org	Lesotho	24/06/2020	Track all the clients and sent SMS reminder for appointments, 208 users	Service delivery
mHero Liberia IntraHealth International, mHero@intrahealth.org	Liberia	01/01/2014	mHero is a two-way, mobile phone-based communication system that uses basic text messaging, or SMS, to connect ministries of health and health workers. mHero operates on simple talk-and-text mobile devices—no smartphone or tablet required. IntraHealth International and UNICEF created mHero in August 2014 to support health-sector communication during the Ebola outbreak in Liberia.	Service delivery

mHero in Liberia Intrahealth International, digitalhealth@intrahealth.org	Liberia	01/12/2013	mHero was developed to link health worker data in iHRIS to RapidPro so Mininstry of Health staff could communicate with frontline health workers during the Ebola crisis	Health information system
Liberia Health Analytics Platform (LHAP) Claire Cravero, claire@zenysis.com	Liberia	01/01/2018	The Zenysis software integrates data from fragmented systems through a generalized interoperability layer that uses data science techniques to harmonize the differences between integrated systems without requiring any modification to the systems themselves.<br><br>In 2018, Liberia's National Public Health Institute (NPHIL) and the Ministry of Health (MOH) partnered with Zenysis to improve infectious disease surveillance, data quality, supervision and capacity for data-driven decision-making. In Liberia, Zenysis integrates more than five fragmented data sources for the first time, including lab data, weekly disease surveillance data and routine Health information system data from DHIS 2.<br><br>This partnership with Zenysis allowed the MOH and NPHIL generate analyses that they used to influence health sector policy planning, resource allocation and intervention design. The rapid integration of data systems helped Liberia target activities to strengthen health systems to improve public health outcomes.	Health information system

mTOMADY Jennifer Bencivenga, jb@mtomady.com	Madagascar	01/05/2017	mTOMADY is a digital end-to-end platform for financial inclusion in healthcare. It improves access to essential health services, strengthens resilience against medical impoverishment, and supports efforts to achieve Universal Health Coverage in low-resource settings. The platform connects beneficiaries, healthcare providers, and payers over the existing mobile money infrastructure, and enables the secure exchange of healthcare payments and structured data. mTOMADY is used by:<br>- Beneficiaries to access health information and financing;<br>- Healthcare providers to send data and receive delay-free payments;<br>- Payers, such as governments, health insurance providers, or donors to manage beneficiaries, claims, and payouts. Automated results based-payments can also be used to incentivize community health workers or agents. mTOMADY circumvents restrictions on smartphone ownership and internet access, enabling it to function in the most remote, low-resource, and infrastructure poor areas.	Healthcare finance, Service delivery
USAID ACCESS m-health initiative Stephanie Ranaivo, sranaivo@usaidaccess.org	Madagascar	01/10/2018	The ACCESS m-health project aims to improve the quality of both services and data collection, reporting and use. A mobile application which uses CommCare platform (www.dimagi.com) has been built. It integrates Madagascar Ministry of Public Health algorithms, validation checks, job aids, and digital reporting forms. The app has two versions: One version for the community health volunteers (CHV) has modules on family planning, maternal health, child health, growth monitoring, promotion of healthy behaviors, stock information, referrals, community-based surveillance, and monthly activity reporting. The health centers version has modules on CHV supervision (consultation of CHV reports, calculation of CHV performance score,	Service delivery, Health information system

			inventory of available registers on site) and monitoring of referrals made by CHVs (development of e-learning modules is ongoing). Over 350 users have been trained so far, and are using the app. Over 5,000 users are planned by 2023.	
Covid-19 DHIS 2 Rebecca Potter, covid@dhis2.org	Madagascar	08/05/2020	The DHIS2 digital data package for COVID-19 is designed to accelerate case detection, situation reporting, active surveillance and response in countries. The COVID-19 digital data package includes standard metadata aligned with the WHO's technical guidance on COVID-19 surveillance and has been adapted to local country context and language in this implementation.	Health information system
The Malawi Digital Village Clinic (DVC) Christopher Kulanga, chriskulanga@gmail.com	Malawi	01/06/2012	The Digital Village Clinic is community based decision support mobile application running on android phones, integrating health service packages offered by trained community health workers called Health Surveillance Assistants (HSAs). The DVC has integrated: Community Integrated Management of Child Illnesses (CIMCI), Community Based Maternal and Neonatal Health (CBMNH), Community Based Family Planning (CBFP) and cStock. The DVC allows to switch ON/OFF any of the above based on the HSAs profile.	Service delivery
cStock - EPI Erick Mwale, Erick.Mwale@savethechildren.org	Malawi	09/01/2018	Adaptation of the existing cStock system to allow for health facilities to report on EPI commodities via SMS.	Service delivery, Access to medicine, vaccine and Technology
UBALE (MIRA STUDY) Dane Fredenburg, dane.fredenburg@crs.org	Malawi	01/07/2016	The MIRA Study App measures the resilience by the people affected by the droughts in 2015 and how they are responding to shocks caused by the droughts. A network of field agents have been trained to administer a survey to selected households in the targeted areas, monthly.The data collected from a series of sentinel sites in southern Malawi is used to map out, with high precision, where the most food insecure	Health information system

			households are, informing targeting decisions to improve food security and nutrition.	
Medic at St Gabriel's Hospital Alex Ngalande, unknown@yahoo.com	Malawi	26/01/2018	A DIY version of the Medic Mobile platform, allowing the St Gabriel's team to use their health volunteers to register pregnant mothers and adapt it for palliative care.	Health information system, Service delivery
ONSE - Mobile Village Toolkit Rudi Thetard - ONSE Chief of Party, rthetard@msh.org	Malawi	15/11/2016	Health systems strengthening: Quality, accessible, high-impact interventions in IMCI, MNCH, FP/RH, malaria, WASH, and nutrition at the facility and community levels depends on reliable platforms for community engagement and feedback, in this case CommCare mHealth platform.	Service delivery
ONSE - Integrated Supportive Supervision Toolkit Rudi Thetard - ONSE Chief of Party, rthetard@msh.org	Malawi	15/11/2016	The tool supports programme supervision for health facilities at the various levels of care	Service delivery, Health information system
ARGUS Joseph Wu, wcsg@lukeinternational.no	Malawi	01/08/2017	This is a pilot project supported by UNICEF and WHO to implement a mobile health (mHealth solution) developed by WHO to strengthen Malawi's integrated diseases surveillance and response (IDSR) system and aiming to deploy in 14 districts of Malawi to generate weekly IDSR reports onward. The solution is to implement SMS connection between health facilities and Epidemiology units to submit weekly IDSR report. Management dashboard will be deployed at national and district level for surveillance use.	Health information system
Supporting LIFE Joseph WU, wcsg@lukeinternational.no	Malawi	01/05/2013	The Supporting Low-cost Intervention for disease control (Supporting LIFE) project is designed to support HSAs at the patient point-of-care as well as larger epidemiological bodies to manage and control diseases through the utilization of cost-effective technologies through large scale community trial. The overarching objective of the Supporting LIFE project is to assist HSAs in delivering an intervention to improve and manage disease control, targeting children aged ≥2 months up to 5 years.	Service delivery

Malawi electronic integrated disease surveillance and response system (Malawi eIDSR) Joseph Wu, wcsg@lukeinternational.no	Malawi	01/03/2016	Thee project is to extend the existing infrastructure of EMRs and implement an eIDSR system to facilitate real-time detection and reporting of notifiable disease cases. Activities include: Enhance and development of border posts health surveillance system, optimize the existing national EMRs for surveillance purposes, dvelop and deploy eIDSR system management dashboard, deploy the integrated eIDSR system and functions to selected health facilities, capacity building and ongoing supports.	Health information system, Service delivery
One Community Steve Choko, stevec@jhuccpmw.org	Malawi	15/12/2015	One Community digital health system is a BI-DIRECTIONAL referral system. This means that referrals may be issued by CRPs to facilities and/or community services; referrals may also be issued by RLFs or community service facilitators into the One Community program. CHWs are given tablets for data collection, entry and other program-related activities. After data collection, data is entered on CommCare Android app and synchronized with the server where all data is stored.	Service delivery, Health information system
Malawi EID ART Initiation Tracking Sean Blaschke, sblaschke@unicef.org	Malawi	01/02/2017	The project uses a system developed using RapidSMS framework to track EID ART Initiations. HCWs register samples when they are collected using numbers printed on Pink cards. When sample are picked from Health centers, they are also crosschecked at the district lab to ensure the meet all standard before they proceed to the reference lab. Lab personnel confirm receipt of sample through sms.	Service delivery
eIDSR Case Based Surveillance App Malawi Soyapi Mumba, soyapim@gmail.com	Malawi	01/02/2017	The Epidemiology department of Ministry of Health is responsible for surveillance of key epidemic-prone diseases such as cholera, Ebola and ensuring timely response on reported cases.	Health information system
Chipatala Cha Pa Foni (CCPF) Health Center by Phone Malawi Carla Blauvelt, carla.blauvelt@villagereach.org	Malawi	01/09/2015	CCPF connects rural communities to mobile-based, personalized Ministry of Health-verified health information and referrals to extend the	Service delivery

			reach of health services and reduce burden on health facilities.	
Barr Foundation iCCM mHealth project Erica Layer, elayer@d-tree.org	Malawi	03/02/2016	The ultimate goal is to provide a holistic, high quality care in iCCM. The HSA supervisor component has the iCCM supervisory checklists to guide the SHSAs during routing program supervision. Online iCCM dashboards displayed usage statistics and program performance information	Health information system, Service delivery
CMAM Stock Monitoring System Malawi Sylvester Kathuma, kathumbasylvester@gmail.com	Malawi	01/12/2017	It is SMS based system to monitor and track availability of key nutrition commodities at health facility level	Access to medicines, vaccines and technology, Service delivery
Medic Mobile DIY Malawi Jacqueline Edwards, jacqueline@medicmobile.org	Malawi	01/01/2018	Using Medic Mobile textforms and DIY platform to register patients for ANC and palliative care follow up and referrals	Service delivery, Health information system
MNCH mobile content with nutrition focus, available in Chichewa, Tumbuka and English in SMS and voice transcript format Kim Viljoen, kviljoen@gsma.com	Malawi	01/09/2013	The GSMA, together with its global content consortium lead by CABI, developed open-source nutrition and MNCH mobile ready content for Malawi available in Chichewa, Tumbuka and English. All content was produced by in-country specialists and is based on recommended MNCH and local nutrition practices. The Ministry of Health played a vital role in the development and content validation. The Content is available in the form of: topic-specific factsheets, SMSs and voice message transcripts.	Service delivery
OpenLMIS Malawi Rebecca Alban, info@openlmis.org	Malawi	01/12/2017	OpenLMIS is a state-of-the-art, web-enabled, enterprise class electronic LMIS solution that facilitates the requisition and resupply process in low-resource settings for health commodities. The result is more complete, accurate and timely data for decision-making— essential to reducing stockouts, managing waste and losses, and operating an effective and efficient supply chain. Users range from facility-level staff/nurses/pharmacists, to national level managers who approve and ship products.	Access to medicine, vaccine and technology, Service delivery

Fetal Monitoring device and mobile decision support Chris Kulanga, Ckulanga@D-tree.org	Malawi	18/09/2018	Development and use of low-cost fetal monitoring device linked to a mobile application. Used in the facility to guide midwives through assessing fetal monitoring and follow protocols based on the uterine contractions and fetal heart rate and providing decision support based on the pregnant women's data.	Service delivery
Malawi German Health Programme (MGHP) Simon Ndira, simon.ndira@giz.de	Malawi	01/01/2017	We are strengthening health systems with a focus on reproductive health. In this context, we are implementing digital solutions to generate reliable data for decision support across all levels of care. Digital interventions include: roll out of e-Register platform in 10 health facilities, roll out of infrastructure to enable capturing data from health centers directly into DHIS2 in the 40 MGHP health facilities, introduction of the Quality of Care and Performance Index of MoHP, extension of the Government Wide Area Network (GWAN) to select health facilities, promotion of data use for decision support through the quality improvement and work improvement support teams in health facilities; strengthening digital governance and digital leadership at national level, including contribution to the drafting of the digital health strategy, definition of national health indicators, drafting of standard operating procedures for HIS among other activities.	Health information system
DHIS2 for COVID-19 Surveillance: Malawi Rebecca Potter, covid@dhis2.org	Malawi	08/05/2020	The DHIS2 digital data package for COVID-19 is designed to accelerate case detection, situation reporting, active surveillance and response in countries. The COVID-19 digital data package includes standard metadata aligned with the WHO's technical guidance on COVID-19 surveillance and has been adapted to local country context and language in this implementation.	Health information system

Post disaster IDSR Strengthening in 14 targeted districts of Malawi Joseph Wu, wcsg@lukeinternational.no	Malawi	06/08/2019	In March 2019, Cyclone Idai affected 15 districts in Malawi. After the initial response to the emergency, the government noticed the surveillance systems in the districts was not fully equipped and recommended enhancement. One Health Surveillance Platform with coordinated Integrated Disease Surveillance and Response system (IDSR) was desired by the government. A UNICEF funded Argus pilot in 2017 implemented by Luke International (LIN) was able to improve the country's weekly IDSR reporting rate from 0.57% (early 2017) to 31.7% (2018). LIN has been working with the MoH to strengthen the IDSR system through developments of various digital solutions, including DHIS2. Hence, UNICEF commissioned LIN for this project with MoH approval and support. The goal of this project is to improve IDSR reporting timeliness and completeness in 14 flood-prone targeted districts through a customized DHIS2 mobile application integrated with the national DHIS2 instance with One Health Surveillance approach.	Health information system

Electronic Participant Locator (ePAL) Luke Banda, lsbanda@mlw.mw	Malawi	01/04/2022	Malawi, like many low-income settings, has no municipal address system. The rapid proliferation of urban high-density housing and slums in Africa and Asia is highly conducive to the spread of disease and makes it extremely difficult for public health officials and researchers to identify hotspots of disease transmission. ePAL is a simple, low-cost geolocation digital health innovation that allows real-time capture of GPS coordinates (e-locate) from important sites of disease transmission at high resolution (within 2 metre) by health workers and researchers working remotely from patient households (e.g. GPS coordinates of a patient's household can be captured while the patient is physically in the clinic, without the need for a home visit). To ensure accuracy and ease follow-ups, ePAL has a function (e-tag) that allows health workers and researchers to capture pictures of landmarks and other relevant sites and upload them to a server. ePAL also presents opportunities to public and private organisations (e.g. banks) that currently still use paper-based maps to identify locations of clients. Paper-based maps are often prone to error and inaccuracy, leading to difficulties in tracking.	Health information system, Service delivery
Advanced mobile toolkit alongside Muso's ProCCM model in Mali Fatou Fall, fatou@medicmobile.org	Mali	16/01/2017	An advanced smartphone application for CHWs to support, coordinate, and track the work of CHWs coupling with tools for supervisors and dashboards for health managers at multiple levels of the health system (health centers, districts, regional and national). Currently, we are implementing mobile health tools to improve malaria Rapid Diagnostic Test (mRDT) quality, coverage, and equity as part of integrated primary health care delivery.	Service delivery

BIOWINPHARMA Mamadou Dakouo, contact@biowinpharma.com	Mali	30/06/2019	BIOWIN EMR makes Electronic Medical Records (EMR) easy, accessible anytime and anywhere to healthcare professionals and patients in 16 West African countries. BIOWIN EMR is West Africa’s open-source Electronic Medical Records (EMR) solution. We start with an open-source EMR software originally developed under GNU general public license and we create BIOWIN EMR Mobile Application and online platform. We deployed a tested product in June 2020 and the number of users is growing daily. We are making EMR easy and accessible anywhere and anytime to healthcare professionals and in 16 West African countries. Its functionality includes Electronic Medical Records, Electronic Prescription, Practice Management, Practice Integration, Medical Billing, Patient Engagement and Third Party Integration. BIOWIN EMR is West Africa’s premier open-source Electronic Medical Records (EMR) solution provider. We start with an open-source EMR software originally developed under GNU general public license and we create BIOWIN EMR Mobile Application and online platform.	Health information system, Service delivery

DHIS2 for COVID-19 Surveillance: Mali Rebecca Potter, covid@dhis2.org	Mali	08/05/2020	The DHIS2 digital data package for COVID-19 is designed to accelerate case detection, situation reporting, active surveillance and response in countries. The COVID-19 digital data package includes standard metadata aligned with the WHO’s technical guidance on COVID-19 surveillance and has been adapted to local country context and language in this implementation.	Health information system

Access to Infant and Maternal (AIM) Health Plus Mauritania Zeine Abidine Ba, zeine_abidine@wvi.org	Mauritania	01/01/2017	With the support of Irish Aid, the AIM Health Plus project addresses the leading causes of maternal and neonatal mortality and improves young child survival and nutritional status across four countries in Africa. Promoting behaviour change at the household level has been the emphasis. In Mauritania, the AIM Health Plus project has included a digital health component since 2019.<br><br>The project provides community health workers (CHWs) with smartphones equipped with a tailored version of Dimagi's CommCare software to use during their home visits. The application supports CHWs who are using the Timed and Targeted Counselling (ttC) approach to promote positive health and nutrition behaviour change among pregnant women and mothers or caregivers of children under 2. The application provides reminders to help CHWs to visit homes at the ideal time during pregnancy, infancy and childhood. It also supports CHWs as they conduct counselling sessions, including enabling them to submit community health data in near real time. This data is then used for managing CHW efforts, planning and decision-making. To boost the acceptability of this digital health tool, local language audio clips are incorporated into the application.	Service delivery, Health information system

upSCALE Joaquim Rebelo, j.rebelo@malariaconsortium.org	Mozambique	01/02/2016	The upSCALE platform consists of an interactive smart phone application that guides community health workers (CHWs) through patient registration, routine health checks, diagnosis, treatment, referral, and follow-up. The app automatically collates data for the CHWs' monthly reports, which they can submit to their supervisors from their phones. A complementary tablet-based application for supervisors enables them to improve CHWs' performance and feedback. Nationwide scale-up anticipated by 2020.	Service delivery, Health information system
MNCH mobile content with nutrition focus, available in 4 languages in SMS and voice transcript format Kim Viljoen, kviljoen@gsma.com	Mozambique	01/09/2013	The GSMA, together with its global content consortium lead by CABI, developed open-source nutrition and MNCH mobile ready content for Mozambique available in Portuguese, Changana, Makwa and English. All content was produced by in-country specialists and is based on recommended MNCH and local nutrition practices. The Ministry of Health played a vital role in the development and content validation. The Content is available in the form of: topic-specific factsheets, SMSs and voice message transcripts.	Service delivery

mAlert Safa Naraghi, safa@zenysis.com	Mozambique	11/03/2019	In March and April 2019, Cyclone Idai and Cyclone Kenneth hit Mozambique. Cyclone Idai was the largest natural disaster to hit Africa in more than 25 years, and both cyclones caused significant loss of life and property. To manage and analyze the voluminous and diverse data generated by the response, the National Institute of Health appointed Zenysis as its lead technology partner for the response.<br><br>The platform is used to produce Daily and Weekly Epidemiological Bulletins, National Situational Reports, detailed epidemiological presentations and analytics that helped inform critical daily discussions and decisions in emergency health cluster meetings and governmental discussions. The Zenysis platform was also used to generate analytics that helped secure cholera vaccines and determine the location of cholera vaccination sites. The platform's outbreak investigation tool continues to be used to identify, respond to, and record data about individual cases that threaten public health.	Health information system
eSIP-Saude Devan Manharlal, devan.manharlal@jhpiego.org	Mozambique	05/01/2015	eSIP-Saúde: Mozambique's novel approach for a sustainable Human resources for health information system. Over the past decade, governments and international partners have responded to calls for health workforce data with ambitious investments in human resources information systems (HRIS). However, documentation of country experiences in the use of HRIS to improve strategic planning and management has been lacking. The purpose of this case presentation is to document for the first time Mozambique's novel approach to HRIS, sharing key success factors and contributing to the scant global knowledge base on HRIS.	Human resources for health

SIFIn Júlio Tualufo, julio.tualufo@jhpiego.org	Mozambique	01/02/2011	Mozambique faces a chronic shortage of trained health personnel. One strategy used to increase the output of training institutions(HTI) has been the direct management of training by the MOH. The MOH has established a network of 18 HTI. These institutions provide middle-level (equivalent to 12 years of schooling) specialty training in the areas of general and maternal and child health nursing, pharmacy, clinical laboratory, ophthalmology, orthopedics and physiotherapy, nutrition, general medicine, Psychiatry, instrumentation, preventive medicine and others. Over the years, a need was felt concerning access and quality of information on training outputs and efficiency. A decision was made by the MOH to develop and implement and information system to serve two purposes: as an academic registrar, to guide and organize student and faculty management at the local level of each training institution. The information system was called as SIFIn (Standing for Sistema de Informação para Formação Inicial).	Human resources for health
OpenLMIS Mozambique (SELV) Rebecca Alban, Info@openlmis.org	Mozambique	01/01/2014	In Mozambique, the customized implementation of OpenLMIS for vaccine management is called Sistema Electrónico de Logística de Vacinas (SELV). By the end of 2014, SELV had been deployed to four provinces on desktop, laptop, and mobile devices, serving over 400 health centers and their combined 10 million patients. In 2016, an extended deployment to make SELV available to all regions began with a completion date in early 2017. Next phases of the SELV program include full integration with its medical commodity supply chain counterpart, SIGLUS.	Access to medicines, vaccines and technology, Service delivery

OpenLMIS Mozambique (SIGLUS) Rebecca Alban, Info@openlmis.org	Mozambique	01/01/2015	Through an agile development approach, this project expanded on the open-source OpenLMIS platform to include an Android application that captures key workflows at the health facility level including stock management, automated requisition orders, and alert algorithms for stock levels and expiries. The iterative development process engaged MoH stakeholders to ensure the system was built upon the correct business logic used in the Mozambican supply chain. Stakeholder use cases also informed the development of a web portal where real-time visibility over facility-level data is available for reporting, visualization, and analysis. A multi-center field pilot provided an opportunity to refine and improve the product based on user acceptance testing. Over two years of development, piloting, and evaluation, the mobile application and web portal were approved for national scale-up in Mozambique. Next phases of the SIGLUS program include full integration with its immunization supply chain counterpart, SELV.	Access to medicines, vaccines and technology, Service delivery
PharmaDexMZ Kim Hoppenworth, khoppenworth@mtapsprogram.org	Mozambique	01/01/2016	Pharmadex is supporting Medicines Registration workflows to issue marketing authorizations for pharmaceutical products.	Access to medicines, vaccines and technology

PENSA Valter Cumbi, vcumbi@sourcecode.solutions	Mozambique	30/11/2017	Our solution consists of a multi-channel and bi-directional mHealth platform available t all operators by dialing *660# and online at www.pensa.org.mz. It is free fo use and was designed to avail pertinent health information to all in Mozambique, particularly those in the rural areas and with low-tech phones. It provides access to:<br>- information on symptoms, causes, and preventive measures for infectious and non-infectious diseases;<br>- contact details for all the public health facilities throughout the country;<br>- maternal & child health information (such as the national vaccination schedule, what to expect during pregnancy, how the baby should develop in the first year, etc);<br>- a means of submitting suggestions/questions to the Ministry of Health(MOH) and more;<br><br>We also updated our platform with relevant information about Covid-19, such as causes, preventive measures, quarantine, masks, social distancing, and important contact information.	Service delivery
DHIS2 for COVID-19 Surveillance: Mozambique Rebecca Potter, covid@dhis2.org	Mozambique	08/05/2020	The DHIS2 digital data package for COVID-19 is designed to accelerate case detection, situation reporting, active surveillance and response in countries. The COVID-19 digital data package includes standard metadata aligned with the WHO's technical guidance on COVID-19 surveillance and has been adapted to local country context and language in this implementation.	Health information system

Community Scorecard App_Mozambique Robert Worthington, rob@kwantu.net	Mozambique	08/03/2022	Community Scorecards are a widely used tool to build a trusted and constructive relationship between communities and health facility staff. The Digital Community Scorecard App helps staff or volunteers running community scorecards to digitize and analyze the data generated from this process. It is comprised of three related tools. A simplified data entry app that is designed to work offline. A programme management app that includes real-time analysis tools and a data hub, that is able to aggregate and visualize data from multiple organizations on a private or public data portal.	Leadership and governance, Health information system
Namibia Master Facility List Maria Helao, Maria.Helao@mhss.gov.na	Namibia	01/01/2017	The Namibia Master Facility List was developed to serve as a central pillar of the Ministry of Health and Social Services Health Information Architecture, standing as a central resource providing information on all public and private health facilities. The system provide geo-coordinates as well as service availability, staffing and infrastructure levels and has a full API to connect with other relevant systems in country.	Service delivery
Namibia Planned Parenthood Association (NAPPA) Erin Sullivan, erin.sullivan@jhpiego.org	Namibia	01/01/2018	Digital tool used at Namibia Planned Parenthood Association clinics supporting registration and workflow of clients.	Service delivery, Health information system
DHIS2 for COVID-19 Surveillance: Niger (in development) Rebecca Potter, covid@dhis2.org	Niger	08/05/2020	The DHIS2 digital data package for COVID-19 is designed to accelerate case detection, situation reporting, active surveillance and response in countries. The COVID-19 digital data package includes standard metadata aligned with the WHO's technical guidance on COVID-19 surveillance and has been adapted to local country context and language in this implementation.	Heealth information system

Act to Save (A2S) Ezéchiel Mahamane, ezechiel_mahamane@wvi.org	Niger	01/01/2021	Preventable infectious diseases such as malaria, diarrhoea and pneumonia are the leading contributors to deaths in children under 5. About a third of those deaths also occur during the perinatal and infancy periods due to conditions such as neonatal sepsis, birth asphyxia and congenital anomalies. The Act to Save (A2S) project aims to address these leading causes of mortality of children under 5 in six communes in the Dosso and Maradi regions of Niger.<br><br>As only two out of every five births take place in local health facilities, A2S will effect change both in facilities and in communities by: promoting healthy behaviours in households through the uptake of essential family practices; bringing quality curative care to families through effective health screenings and referrals offered by community health workers (CHWs) making home visits and providing efficient and effective medical care in environments that are welcoming, sanitary and free of infection to children and families referred to health care facilities.<br><br>This approach aligns with Niger's national public health strategy and will be achieved using the CommCare data collection platform. This approach will also leverage the comprehensive management information system properties of a bundled suite of technology tools built on the Salesforce platform, informally referred to as Sinai.<br><br>A2S is supported by World Vision United States' Every Last One Campaign and is being implemented using a multi-stakeholder framework with government, non-governmental organisations and UN agency partners.	Service delivery, Health information system

HelloMAMA Steve Ollis, steve.ollis@mcsprogram.org	Nigeria	01/10/2015	MAMA delivers vital health messages to pregnant women and new mothers through age- and stage-based mobile messaging that validates and complements the efforts of frontline health workers. The program is locally-led, planned for scale, and complements the national health care system by aligning with health priorities, policies, and systems from the outset. MAMA aims to increase consistent antenatal care visits and facility deliveries, thereby reducing maternal and neonatal mortality rates.	Service delivery
USAID funded - CaTSS OpenMRS Electronic Medical Record System for 19 ART Clinics Benjamin Akinmoyeje, bakinmoyeje@msh.org	Nigeria	10/08/2015	OpenMRS is an emr system that allows hospitals and health facilities to manage all patient data. In addition, it can aggregate patient data and facility management data for monitoring and decision-making. The application is installed on Linux OS • MySQL—relational database management system (RDBMS); • Apache Tomcat—servlet application; • Mozilla Firefox—web browser application, and; • Hibernate—object to relational mapping and persistence application.	Health information system, Service delivery
WeMUNIZE Steve Ollis, steve.ollis@mcsprogram.org	Nigeria	22/03/2017	The WeMUNIZE system is developed to increase demand for immunization services using the voices of local influencers and SMS to remind caretakers and gatekeepers of the importance of childhood vaccinations and of upcoming appointments. The system is complemented by a mobile registration system on Android phones and a tracking system using RFID and barcodes to track children as they receive services. Caretakers are also provided a photo of their child during each visit to the health facility.	Service delivery

CarePay mobile health wallet Steve Maina, s.maina@carepay.co.ke	Nigeria	18/12/2018	CarePay is a digital health payment platform that directly connects patients, payers and healthcare providers and exchanges money and data between them. In Nigeria, the platform is being utilized as a mobile solution for administration and enrollment on the Lagos State Health Scheme. It will be used to improve decision-making and transparency, reduce transaction costs, and increase access especially for poor households.	Healthcare financing
MNCH mobile content with nutrition focus, available in 5 local languages in SMS and voice transcript format Kim Viljoen, kviljoen@gsma.com	Nigeria	01/09/2013	The GSMA, together with its global content consortium lead by CABI, developed open-source nutrition and MNCH mobile ready content for Nigeria available in Hausa, Igbo, Pidgin, Yoruba and English. All content was produced by in-country specialists and is based on recommended MNCH and local nutrition practices. The Ministry of Health played a vital role in the development and content validation. The Content is available in the form of: topic-specific factsheets, SMSs and voice message transcripts.	Service delivery
NaijaCare Dr Saidat Akanbi, saidat@every1mobile.com	Nigeria	01/10/2017	NaijaCare is a digital platform strengthening linkages between informal medicine vendors, community pharmacists and health facilities for improved primary health care in underserved communities in Nigeria.	Service delivery
Shugabanci Agbons Oaiya, bonkhi@gmail.com	Nigeria	30/06/2020	Support Kaduna and Niger state to establish a coordinating and governance structure for HRH domiciled at Primary Health Care, in accordance with the Primary Health Care Under One Roof (PHCOUR) policy. During this process, which included transferring HRH governance and management as well as payroll, an HRH-IS was developed.	Human resources for health

NaijaCare Project Moshood Abdullateef, moshood@every1mobile.com	Nigeria	01/05/2018	NaijaCare is a digital peer community that provides a suite of digital services for medicine vendors (PPMVs) in Nigeria. The digital tools such as e-learning, peer support, referral management, and online ordering are aimed at improving the livelihoods of PPMVs and their customers as these tools allow PPMVs to provide better Service delivery while growing their businesses. NaijaCare is implemented by Every1Mobile with support from Bill and Melinda Gates Foundation and Unilever. Through our pilot which started in May 2018, we have demonstrated that e-learning can help upskill medicine vendors in areas of record keeping, administering of rapid diagnostic tests for malaria, and taking a rights-based approach in offering family planning services to customers.	Service delivery, Human resources for health
Complete Health Nneka Mobisson, nneka.mobisson@mymdoc.com	Nigeria	01/01/2018	CompleteHealth is a digital health platform to support people living with chronic health needs.	Service delivery
DHIS2 for COVID-19 Surveillance: Nigeria (in development) Rebecca Potter, covid@dhis2.org	Nigeria	08/05/2020	The DHIS2 digital data package for COVID-19 is designed to accelerate case detection, situation reporting, active surveillance and response in countries. The COVID-19 digital data package includes standard metadata aligned with the WHO's technical guidance on COVID-19 surveillance and has been adapted to local country context and language in this implementation.	Health information system

Wellvis Wale Adeosun, wale@wellvis.org	Nigeria	23/06/2020	Wellvis is a comprehensive telehealth platform; we provide access on-demand health advice, consultations and care support in real-time offered by verified health professionals. Wellvis is an on-demand health information and service platform. We provide a technology platform that connects individuals with verified and licensed health practitioners in real-time for the purpose of offering health care advisory services as well as appointment bookings and other allied services. We offer a premium option for telemedicine services (direct one-on-one online consultation with verified health workers), appointment booking (plus reminders) for physical consultation with specialists and/or for lab investigations and a portal to access curated health services and products at a discount. We also offer online health support groups + disease management tools for individuals (and/or their caregivers) with chronic illnesses on a subscription model.	Service delivery
SwissTPH x OpenFn: Automated, real-time data integration & disease monitoring Aleksa Krolls, aleksa@openfn.org	Nigeria	01/07/2019	SwissTPH had no way to monitor the diagnoses and treatments of sick children in Nigeria in real-time. To analyze disease patterns across health facilities, SwissTPH researchers would have to wait days until staff members exported, cleaned, and uploaded the diagnoses data collected to DHIS2—a time-consuming data process, prone to human error. Through data integration, OpenFn eliminates time lags, saving SwissTPH money and time and sending critical patient information where and when it is needed, faster. By integrating a CommCare mobile data collection app with a national DHIS2 reporting system for the ALMANACH health initiative, OpenFn has provided SwissTPH with an automated, real-time connection that enables live disease monitoring.	Health information system, Service delivery

Audio Job Aids for Family Planning Providers Emily Mangone, emily_mangone@abtassoc.com	Nigeria	01/01/2019	Through user-centered problem-specification and solution design workshops with family planning (FP) providers in Nigeria, the USAID-funded Sustaining Health Outcomes through the Private Sector (SHOPS) Plus project developed on-demand audio job aids that FP providers could call at any time to select and listen to key technical messages on FP topics of interest. This approach was selected because it met the task-shifting needs of community health extension workers (CHEWs) who could access this information using basic phones— since many did not have smartphones or wifi. Further, the on-demand approach was selected because it was sustainable beyond the duration of the SHOPS Plus project. To implement this intervention, SHOPS Plus partnered with Viamo to leverage the existing 3-2-1 platform with the Airtel network operator. Viamo is a social enterprise that specializes in using IVR, short message service (SMS), Random Digit Dialing, and unstructured supplementary services data (USSD) to support partners with behavior change communications and evidence-gathering via simple mobile phones. The 3-2-1 platform is accessible for free to anyone in Nigeria with an Airtel SIM card and it already offered on-demand voice recordings on health topics, including general FP, for beneficiaries. All informational content on the platform is developed in partnership with local experts, development organizations and appropriate government agencies. In Nigeria, on average, the health messages are accessed 35,000 times each month. SHOPS Plus developed and validated FP curriculum-based content with FP providers and posted 38 key messages, which providers can listen to for free by navigating through an interactive menu. The menu topics for FP providers include: counseling, general FP	Service delivery

			information, safety and documentation, short-acting methods, long-acting methods, and pelvic exam. Only approved clinical providers who were given an access code could access this content.	

Lafya HealthShare Nanlir Kapnan, kapnan@lafya.co	Nigeria	02/11/2021	We are building a healthcare wallet that enables friends, families and companies to save money and split the cost of their medical expenses with each other.<br>Our product connects the unbanked population in the rural areas to the banked population in the urban areas through their relationships and enables them to save money in a mobile wallet and access healthcare financing when they have a medical emergency.	Healthcare finance

HelpMum	Dr Abiodun Adereni, abbey2real@gmail.com	Nigeria	01/01/2017	HelpMum's Mission is to reduce maternal and infant mortality in Nigeria. Our overall strategy and service to underserved communities has now been fully tested and adapted to the Covid pandemic and we are combining practical and digital approaches: HelpMum is addressing immediate & critical needs "on site" by providing basic, inexpensive HelpMum birth kits containing essential supplies required at childbirth to ensure a safe and hygienic delivery, fully involving the traditional birth attendants who playing a key role in these remote communities. As a note of context, 2/3 births in Nigeria still occur at home, with only 1/3 are attended by doctors or midwives. HelpMum also addresses the underlying lack of access to information & education to traditional birth attendants, via digital tutorials available on the HelpMum E-learning Platform providing TBAs short video tutorial on general clean and safe delivery practices in their own indigenous language. We recorded WHO midwifery tutorials in Nigerian indigenous languages to help disseminate best practices whether or not Wi-Fi is available. We fully leverage mobile & digital technologies to enhance our capacity to service more women in remote communities. We developed a proprietary IT telecom application, the HelpMum Vaccination Tracker which can be uploaded on any telecom	Service delivery, Human resources for health

			device, to help increase immunization awareness and immunization rates in remote rural area in Nigeria. We are currently in improving our vaccination tracking system with new Ai innovation in partnership with Google and Vanderbilt University.	

Real-Time Monitoring of Moderna Vaccines in collaboration with National Primary Health Care Development Agency. Oghenetega Iortim, tega@gricd.com	Nigeria	16/08/2021	Nigeria, Africa's most populous country, requires a more technical approach, structure, and infrastructure to support the effective distribution of vaccines to millions of Nigerians in both rural and urban regions in a short amount of time. In August 2021, Nigeria received a donation of 4.2 million doses of Moderna vaccines from the US Government. However, unlike Astrazeneca vaccines that need to be transported at 2-8 degrees, the Moderna vaccines are stored at a much lower temperature. Although upon arrival, the vaccines were frozen within the appropriate temperature range of -50°C and -15°C, the Moderna vaccines are to be stored at -15°C to -25°C degrees.<br><br>To mitigate waste and ensure optimal potency of the vaccines, the National Primary Health Care Development Agency, a parastatal of Nigeria's Federal Ministry of Health, engaged the services of Gricd Integrated Services by using our MOTE device, a Multi-Purpose Real-time Data Logger, and enterprise software for monitoring the temperatures of the vaccines while in storage and transit.<br><br>With 36 states and a landmass of 923,768km² to cover, NPHCDA, the Nigerian government arm tasked with the responsibility of getting these vaccines to the last mile, has her work cut out. This includes ensuring the vaccines get to each state in the best condition and being able to attest to the potency of the vaccines, both of which Gricd device and software guarantee.<br><br>Gricd MOTE is placed in all containers transporting the Moderna to generate information such as the temperature and location of the vaccine remotely. If the vaccines deviate from their intended route or exceed the	Service delivery, Access to medicine, vaccine and Technology

			temperature range, alerts are sent to the appropriate parties which helps them to ensure the vaccines maintain their potency and prevent vaccine wastage. Temperature excursions for all 4.2 million doses of Moderna Vaccines can be easily checked because the temperature of these vaccines are provided on the Gricd's Enterprise Software.<br><br>The National Primary Health Care Development Agency on the 13th of September, 2021 disclosed that a total of 1,407,736 people have received their first dose of the Moderna Vaccine and hitherto, no cases of ineffectiveness of the Vaccines has been recorded, this means that the agency has been able to properly monitor the vaccines while in storage and transit using the Gricd Mote and Enterprise software to ensure the effectiveness of the vaccines.<br><br>The goal is to save lives, reduce waste, and increase access to vaccines.	
ulerawa luke shors, lshors@icloud.com	Nigeria	31/08/2021	Currently a pilot project of a new claims system in primary health facilities in the Ekiti state.	Service delivery, Healthcare finance
Quick Health Chris Agape Ajah, hello@getquickhealth.com	Nigeria	01/07/2021	Quick health helps aggregate various health service providers on one platform for easier health access	Service delivery

PHARMARUN IBITAYO TAIWO, tayotaiwo83@yahoo.com	Nigeria	21/11/2021	PHARMARUN is an open source, mobile and web-based application that will allow users order for non-prescription drugs, request for vaccines, make payments and get delivery with minimum time where applicable. PHARMARUN also intends to track drug usage and compliance, vaccine distribution and usage using Artificial Intelligence algorithms. PHARMARUN will also be scaled further to respond to emergencies. Users will also have a wallet funded through multiple channels – Banks, crypto, USSD etc – where instant purchases can be made. Users will also earn based on referrals and active usage. DESIGN PRINCIPLES PHARMARUN when launched, will be a new paradigm from the conventional method of gaining access to drugs. We predict in a few years, PHARMARUN will become a seamless channel for dispensing drugs. Our design principle will address;<br>1. Open-Source Technology usage<br>2. Integrate with Third-party applications and databases e.g., Payment gateways, delivery services, GPS Services.<br>3. Multi factor authentication to secure users from attacks or compromise<br>4. Age verification and prevention of multiple user profile<br>5. Provisioning service for Rural areas with little access to internet using USSD services.<br>6. Scalability in terms of needs and population demography<br>7. All deployed technology is of global standards<br>8. Sustainable and affordable business model to keep the business growing while providing affordable service<br>9. Support multiple repository systems<br>10. Ensure recruitment of skilled Human	Service delivery, Access to medicine, vaccine and Technology

			Resources to mitigate any downtime<br>SOLUTION<br>PHARMARUN main objectives is to provide an electronic platform to order for non-prescription drugs, request for vaccines and track usage. In order to achieve this, PHARMARUN will need to cover the following modules;<br>1. Identity - Each user must create a profile, or link with google account or Facebook. This will help prevent one user having more than one account<br>2. Search inventory - select services required, either drugs or vaccine, then specify type<br>3. Determine dosage by probing a few questions – input few parameters such as age, history etc<br>4. Locate nearest Pharmacy or vaccine center - GPS service will locate a pharmacy nearest to user and dispatch request immediately<br>5. Fund wallets and make instant payment - Fund wallets through multiple channels so orders can be charged directly from users' wallet. This makes processing faster.<br>6. Fast Delivery system – dispatch rider is immediately deployed to make delivery within the shortest possible time, users can monitor dispatch through GPS tracking services.<br>7. Usage Monitor - Upon delivery, system monitors drug usage through AI algorithms to ensure drugs are taken as at when due and right dosage is taken.<br>8. Supplementary USSD service for Rural areas with poor internet access and smart devices.	

EmHealth-Paediatric Digital Health Initiative Dr Agbarakwe Chukwuemeka, brammy@emhealthglobal.org	Nigeria	01/01/2021		Health information system, Service delivery

OneCare Hafeez Ayanda, hayanda@innovastra.com	Nigeria	01/01/2020	OneCare is an eHealth solution that offers an interconnected healthcare system. OneCare is a cloud-based SaaS Electronic Health Records (EHR) fully integrated with Practice Management, Telemedicine, Mobile App, Healthcare Analytics, Pharmacy Information System, Laboratory & Radiology Information System, Document Management, Maternity Care solution, and Billing.<br><br>OneCare enables healthcare providers deliver better, more consistent, coordinated and efficient healthcare. It improves access to health services in remote and under-served communities and empower individuals to manage their own health more proactively and effectively. It offers Health Information Exchange which provide seamless exchange of patient information among healthcare providers.<br><br>OneCare offer patients the opportunity to have their health records move with them at different point of care. Patients are able to access their own health record in the way they choose, and also decide who is able to access it. Clinicians and other caregivers – including doctors, nurses, carers, and social services workers – will be able to access all relevant patient information when they need to, when they see the patient, with the patient's consent. It offers improved data collection providing public healthcare the opportunity to proactively monitor health indicators such as infectious disease prevalence, maternal and child outcomes.	Service delivery

E-Heza Data Solutions Wendy Leonard, MD, AAHIVS, wendy@theihanganeproject.com	Rwanda	01/01/2018	E-Heza is Rwanda's first point-of-care data solution was created alongside front-line health care workers and the Rwanda Ministry of Health. E-Heza will dramatically improve maternal and child health outcomes by giving nurses the tools they need to adopt evidence-based clinical care protocols, provide high quality care and utilize real-time data trends to tailor health education to individual family needs while simultaneously satisfying Rwanda Ministry of Health data reporting requirements. In response to Covid-19, E-Heza is developing an Acute Illness Assessment & Management module for CHWs and nurses at health centers. This module allows CHWs to enter responses to guided screening questions. If Covid-19 is suspected, an alert immediately advises the CHW to isolate the community member and contact the health center. Care management can continue in the same patient record at the health center. If Covid-19 is not suspected, the CHW can continue with assessment and management of the relevant condition. A facility-level Covid-19 module has recently been developed and deployed that allows frontline health workers to provide symptom review and physical exam, rapid testing if indicated, and decision matrix for high and low risk confirmed cases. A contact collection tool in incorporated into the visit so health workers can document contacts. The documented contacts populate a Contacts Management module that allows health workers to follow up on all contacts.	Service delivery

Rwanda Health Analytics Platform (RHAP) Eliachim Ishimwe, eliachim@zenysis.com	Rwanda	13/08/2018	Rwanda Health Analytics Platform (RHAP) is a rapid data integration and advanced analytics platform developed with the Rwanda Biomedical Center (RBC). RHAP enables data triangulation, processing, and visualization of fragmented data. The platform resolves critical differences in datasets by harmonizing location naming, data collection cadence, indicators, names, etc). RHAP is a web application that can be accessed with web connection.<br><br>The RHAP integrates data from the facility level Health information system, the community Health information system, the DHS, the Integrated Supervision Survey and data from an electronic LMIS to support and improve data-driven decision making. Currently the project is looking at expanding the scope by integrating additional datasets such as HIV, TB, etc.	Health information system
DHIS2 for COVID-19 Surveillance: Rwanda Rebecca Potter, covid@dhis2.org	Rwanda	08/05/2020	The DHIS2 digital data package for COVID-19 is designed to accelerate case detection, situation reporting, active surveillance and response in countries. The COVID-19 digital data package includes standard metadata aligned with the WHO's technical guidance on COVID-19 surveillance and has been adapted to local country context and language in this implementation.	Health information system

Rwandan digital ANC module Mr Felix Sayinzoga, felix.sayinzoga@rbc.gov.rw	Rwanda	24/08/2020	Following the adaptation of the national ANC policy and guideline, Rwanda is now embarking on efforts to test innovative ANC Service delivery approaches and mechanisms to improve the quality of antenatal care in line with the 2016 WHO recommendations. Rwanda will be adopting a person-centered digital tracking and decision support system to potentially accelerate the adoption of the new ANC recommendations and policies. This digital health system is a software global good, the WHO digital ANC module, which has been created on the OpenSRP platform. Rwandan Ministry of health will adapt and update the WHO digital ANC module to better reflect the new national ANC policies and Rwandan contexts. Implementation of these modules will take place in selected districts (Nyagatare & Nyanza Districts) that will allow larger scale-up if the initiative is successful.	Service delivery

Rwanda Health Information Exchange System (RHIES) Julie Vanvolsem, julie@savics.org	Rwanda	01/06/2019	The Rwanda Health Information moving disparate systems across to a consolidating electronic system.<br><br>Health data is currently managed on various platforms in Rwanda; paper forms, the EMR, the RHMIS, the electronic Laboratory Information System (LIS) and other databases. The non-integration between these platforms at the facility or national levels affects data integrity and quality negatively. There was therefore a need to develop an integrated Health information system that synchronizes data entry and reporting across various health databases to reduce transcription errors, data capture errors, and reduce reporting time.<br>The “Rwanda Health Information Exchange system” is a set of applications that work together in the Open Health Information Exchange (OpenHIE) architecture to serve point-of-service systems, like EMRs, DHIS2, National ID database and laboratory information system.<br>The main feature of this project was the deployment and configuration of an interoperability layer designed to ease	Health information system
JoCare Jerome Nshimiyimana, njerome05@gmail.com	Rwanda	21/11/2021	Through our Web app Platform, We help youth and parents to know or understand and discuss about sexual reproductive health information through our educative online innovative tools(girls calculator , games, blog, YouTube videos) and We have developed API (Application programming interface..) that can help other website to embed our tools like girls calculators and Web-games to their site , And Also we developed Health related WordPress plugins.	Service delivery

Automation and AI for Health Campaign Planning Sangeeta Jobanputra, sraja@connecti3.com	Rwanda	21/11/2021	We provide an AI-powered, end-to-end automated health campaign micro-planning platform. Our platform is transparent, allowing multiple health campaign planners to work on it at the same time and design entire health campaigns with a few mouse clicks. Our built-in algorithms and predictive analytics take care of the rest: identifying the target population, determining which communities are most in need of services, forecasting staff and medical supply requirements, tracking financial pledges and the flow of funds to health sites, tracking campaign results and performance, and assisting with report preparation. Depending on the complexity of the health campaign, our laboratory test of the platform shows a time savings of two to five months of planning. Our pre-test of the platform's use on two previous Rwandan national health campaigns in 2018 and 2019 revealed that it only reached 26% and 21% of the poorest households, respectively. According to our current predictive analytics, Rwanda's Ministry of Health could have reached an additional 2.1 million people by adding 180 mobile sites in strategic locations. Adding a complimentary service like family planning to a child-focused health campaign increased the relative cost of the overall campaign by only 4%, reaching an additional 50,000 women, according to data analytics conducted with our solution. The system can also provide optimized resource deployment options thanks to the automation of the planning process. For example, we discovered that most people used the health campaign services within the first three days of its implementation, but that staffing was not adjusted to meet demand, resulting in long lines and supply shortages.	Service delivery, Leadership and governance

Suivi nutritionnel des enfants de moins de 5 ans dans la région de Lobata Eduardo, ferreirae@who.int	Sao Tome and Principe	01/06/2019	le projet utilise les téléphones mobiles pour suivre à distance la pesée des enfants enfin de determiner leur état nutritionnel	Service delivery
DHIS2 for COVID-19 Surveillance: Sao Tome and Principe (in development) Rebecca Potter, covid@dhis2.org	Sao Tome and Principe	08/05/2020	The DHIS2 digital data package for COVID-19 is designed to accelerate case detection, situation reporting, active surveillance and response in countries. The COVID-19 digital data package includes standard metadata aligned with the WHO's technical guidance on COVID-19 surveillance and has been adapted to local country context and language in this implementation.	Health information system
Implementation of a Performance-Based Financing Data System for Senegal - using the open source tool Hesabu Dr Malick Cissé Ndiaye, illeyya@yahoo.fr	Senegal	04/06/2012	Data System to Manage the performance based financing program including offline mobile data collection of quality of care and client satisfaction, the computation of program indicators, the calculation of incentive bonuses, the management of the PBF primary and subcontractor relationships and distribution of incentives. In addition, data is displayed publicly through open data dashboards.	Service delivery
Suivi des grossesses dans la région de Kaolack Dr Dia, nourougaloya@gmail.com	Senegal	14/05/2019	suivi des grossesses des femmes à risques pour s'assurer des CPN	Service delivery
NTD SCH-STH prevalance survey Clara Burgert, cburgert@rti.org	Senegal	01/01/2018	Use of Secure Data Kit platform to collect disease prevalence survey information for SCH and STH surveys	Health information system
Informed Push Model - Yeksi-Naa Melanie Joiner, mjoiner@intrahealth.org	Senegal	01/07/2013	To inform a push model of supply chain management, a tablet-based logistics management information system tracks real time facility-level data for viewing at the district, regional, and national levels allowing for improved forecasting.	Access to medicines, vaccines and technology, Service delivery
Senegal immunization supply chain Neeraj Thakare, neeraj@logistimo.com	Senegal	05/01/2020	In partnership with Gavi, Logistimo's SCM platform has been deployed in 2 regions, across ~100 health facilities to improve visibility, tracking and availability of vaccines from the central level to the last-mile. The deployment	Access to medicines, vaccines and technology, Service delivery

			was localized keeping in mind the native context, including French language.	
DHIS2 for COVID-19 Surveillance: Senegal Rebecca Potter, covid@dhis2.org	Senegal	08/05/2020	The DHIS2 digital data package for COVID-19 is designed to accelerate case detection, situation reporting, active surveillance and response in countries. The COVID-19 digital data package includes standard metadata aligned with the WHO's technical guidance on COVID-19 surveillance and has been adapted to local country context and language in this implementation.	Health information system
Aarleen mHealth System Gorgui Diallo, gdiallo@africare.org	Senegal	03/08/2020	This 6-year MCH project funded by Saving Lives at Birth (SLAB) in Senegal aims to reduce maternal and neonatal mortality rates, through improving the timeliness of care, access to health services and strengthening community-provided services. Led by Africare, the project has 2 major interventions - maternal care groups and an mHealth system powered by CommCare. The maternal care groups are run by community health workers (Matrones), and these meetings are used to inform and teach pregnant women about their pregnancies and also to help identify pregnant women as early as possible during their pregnancies. The matrones at the cas de sante level and another for the nurse at the poste de sante level use CommCare to track pregnancy danger signs, track referrals and provide counselling.	Service delivery
electronic Integrated Disease Surveillance and Response (eIDSR) Nelson Clemens, nelson.clemens@sl.ehealthafrica.org	Sierra Leone	01/02/2016	The eIDSR project aims to improve the quality and timeliness of IDSR reporting. This is being achieved through implementation of DHIS2 at district level and the through provision of a tablet and a custom DHIS2 data entry app at facility level. The project also provides training and support to data entry clerks and health facility workers on use of the new technology.	Health information system

CHAMPS DAYO SPENCER-WALTERS, dayo.spencer-walters@sl.ehealthafrica.org	Sierra Leone	01/03/2017	We aim to ensure that all deaths are reported to the 117 system, both from the hospital and communities. This way, mortality surveillance will be improved and causes of deaths in under fives identified	Health information system
VaxTrac Dayo Spencer-Walters, dayo.spencer-walters@sl.ehealthafrica.org	Sierra Leone	01/06/2016	Electronic Vaccination Record and Tracking System	Health information system, Service delivery
AVADAR: Auto-Visual AFP Detection and Reporting Faye Simmonds, faye.simmonds@sl.ehealthafrica.org	Sierra Leone	20/03/2017	Auto Visual AFP Detection and Reporting (AVADAR) is an sms-based mobile technology innovation to improve completeness, timeliness, and availability of acute flaccid paralysis (AFP) reporting data. AVADAR addresses the above problem by "widening the net" of disease reporters, making data available in near-real time, sending automatic case alerts to disease surveillance officers (DSOs), and automatically aggregating and visualizing case alerts and investigations on a dashboard.	Halth informaiton system
117 Alerts System Dayo Spencer-Walters, dayo.spencer-walters@sl.ehealthafrica.org	Sierra Leone	01/09/2014	24 hour hotline built as a syndromic and mortality surveillance tool for early detection of disease outbreaks	Health information system
Electronic Vaccination Record and Tracking System Implementation Project (VaxTrac) Musa Bernard Komeh, musa.komeh@sl.ehealthafrica.org	Sierra Leone	01/06/2016	VaxTrac is a clinic based vaccine registry tool to record vaccine data at the time and place of vaccine administration.<br>The backbone of the system is a mobile, clinic-based immunization registry. The mobile app includes:<br>Clinical decision support to improve the quality of services delivered, Methods for defaulter tracing, Automated reporting that can reduce the administrative burden on front line health workers, and improve the quality of data.	Service delivery, Health information system
Saving Lives Mohammad B. Jalloh, mbjalloh@focus1000.org	Sierra Leone	02/01/2017	We aim to enhance the quality and timely generation of HF data through the digitization of HF forms, deployment of android-based tablets at data entry points - mostly at CHC level - and possible deployment across all CHPs in a bid to promote the timely and complete generation of data from facilities to the DHIS2.	Health information system, Service delivery

RapidPro Kazutaka Sekine, ksekine@unicef.org	Sierra Leone	01/09/2016	UNICEF has been supporting MoHS to deploy RapidPro use cases for informed decision makings.	Health information system, Service delivery
Mobile Training and Support (MOTS) Frederick F. Kamara (World Vision-SL), Fredrick_kamara@wvi.org	Sierra Leone	01/02/2017	The objective of the MOTS is to strengthen the Community Health Worker (CHW) program in support of preparedness of outbreak prevention measures, including vaccination. The development of MOTS will support building a foundation of knowledge, evidence and partnerships to be better equipped for Ebola vaccination and better prepared should a future Ebola outbreak occur.	Human resources for health, Service delivery
The Safe Delivery App in Sierra Leone Lauren Bellhouse, lauren@maternity.dk	Sierra Leone	17/09/2018	Building on successful implementation of the Safe Delivery App in Ghana, UNFPA Sierra Leone chose to roll out the English version of the SDA to a pilot group of midwives. Over an 8-month period, UNFPA Sierra Leone conducted a pilot project of the Safe Delivery App. In partnership with Maternity Foundation and the Ministry of Health and Sanitation, the project was implemented in four districts of Sierra Leone (Western Area, Bombali, Bo, and Kenema) and consisted of baseline data collection (Sept 2018), an introductory training, distribution of the Safe Delivery App, and endline data collection (May 2019). Pre- and post- use knowledge and skills were monitored via a survey (for knowledge) and an objective structured clinical exam (for skills). Results showed a significant difference between knowledge scores at baseline and endline, and a statistically significant increase in skills for post-partum hemorrhage. Phase II is planned (pre-service introduction to the App) once the Covid-19 situation allows.	Human resources for health, Service delivery

Access to Infant and Maternal (AIM) Health Plus Sierra Leone Joseph Musa, joseph_musa@wvi.org	Sierra Leone	01/01/2017	With the support of Irish Aid, the AIM Health Plus project addresses the leading causes of maternal and neonatal mortality and improves young child survival and nutritional status across four countries in Africa. Promoting behaviour change at the household level has been the emphasis. In Sierra Leone, the AIM Health Plus project has included a digital health component since its inception in 2017. The geographic focus is Imperi district and Sherbro Island.<br><br>The AIM Health Plus project provides community health workers (CHWs) with smartphones equipped with a tailored version of Dimagi's CommCare software to use during their home visits. The application supports CHWs who are using the Timed and Targeted Counselling (ttC) approach to promote positive health and nutrition behaviour change among pregnant women and mothers or caregivers of children under 2. The application provides reminders to help CHWs to visit homes at the ideal time during pregnancy, infancy and childhood. It also supports CHWs as they conduct counselling sessions, including enabling them to submit community health data in near real time. This data is then used for planning and decision-making. To boost acceptability of this digital health tool, local language audio clips are incorporated into the application. This digital health intervention is expected to improve the effectiveness of behaviour change communication delivered to women and caregivers of children under 2 as well as strengthen the health system through better use of community-level data.	Service delivery, Health information system

Global Open Facility Registry (GOFR) Reconciliation Tool Telli Koroma, tellikoroma@gmail.com	Sierra Leone	28/02/2018	The Facility Reconciliation Tool is an open source and open standards-based product to help anyone match lists of facilities from different data sources. The tools supports uploading data from CSV, FHIR servers and DHIS2 instances.	Health informartion system
MOTS COVID-19 Monica Amponsah, mamponsah@grameenfoundation.org	Sierra Leone	13/08/2020	MOTS is a mobile service that provides refresher training via voice on vaccination and outbreak response to Community Health Workers (CHWs) with a feature phone as the only requirement. MOTS allows CHWs to receive training in the language of their choice at their own convenience.	Service delivery, Human resources for health
DHIS2 for COVID-19 Surveillance: Sierra Leone Rebecca Potter, covid@dhis2.org	Sierra Leone	08/05/2020	Sierra Leone customized their DHIS2 system for Covid-19 surveillance	Health information system
Instant OpenHIE Daniel Futerman, daniel.futerman@jembi.org	South Africa	01/12/2019	The Instant OpenHIE project aims to address the primary needs of (i) allowing implementers to engage with a preconfigured health information exchange solution and running tools (based on the architecture) and test their applicability and functionality with a real health context problem; and (ii) having a packaged reference version of the OpenHIE architecture that is comprised of a set of reference technologies and other appropriate tools that form the building blocks of the health information exchange that can be configured and extended to support particular use cases.	Health information system
OpenHIM (Open Health Information Mediator) Daniel Futerman, daniel.futerman@jembi.org	South Africa	01/01/2015	The Open Health Information Mediator (OpenHIM) is a middleware component designed to ease interoperability between disparate information systems. It provides secure communications and data governance, as well as support for routing, orchestrating and translating requests as they flow between systems.<br>The OpenHIM is intended as an extensible tool to support interoperability workflows across a wide range of information systems, metadata	Health information system

			services and HIE components, and data standards.	
Public Health Incentives Gugulethu Nyathi, gugu@ribbonblockchain.com	South Africa	19/04/2018	Our technology ushers in the Uberization of Public Health, where the App enables value creation for Community Health Workers, Patients and Practitioners in the public health system. The app enables CHWs to enrol as Care Partners to assist Patients to earn incentives for testing, initiating treatment, adherence to medicine, disease suppression, and living healthier lifestyles. It also enables Patients to rate the Patient Experience of Care (PEC) thereby enabling Practitioners to earn an incentive based on the Quality of care. delivery. This collaboration system creates a network effect resulting in continuous cycle of improved disease prevention, population health outcomes, quality of care delivery and employment for Community Health Workers. Health data generated from this system is beneficiated and monetized via data analytics and the proceeds shared equitably across the participants and the platform. The reason for deploying the system is to automate public health systems for efficiency & UHC implementation	Service delivery
NACOSA Health Analytics Platform (NHAP) Safa Naraghi, safa@zenysis.com	South Africa	13/11/2017	NACOSA is one of the largest implementers of HIV prevention programs in South Africa. The NHAP integrates programmatic, financial and individual-level data from NACOSA's largest sub-recipients in order to better understand Service delivery performance, client retention, cost effectiveness and spending efficiency for each of its sub-recipients among other technical improvements.	Service delivery, Health information system

BroadReach COVID-19 Pandemic Management Fiona de Korte, fdekorte@brhc.com	South Africa	06/04/2020	BroadReach Consulting (BRC) with Vantage Technologies have developed an application and platform-based solution package technologically enabling both the demand and supply side of pandemic management. The demand or patient management side enables population-based screening and testing, contact tracing, and epidemiological surveillance, and the supply or facility management side enables leaders and managers to assess and address facility readiness, stock management and workforce wellness. The users of the solution are front line healthcare workers and decision-makers responsible for operating and managing networks of healthcare clinics and hospitals (e.g., ministries of health, private and NGO hospital/clinics). This solution allows stakeholders to prevent, identify and treat patients of COVID-19 and assess the readiness of facilities to respond to the virus. The solution is currently deployed in South Africa. Further phases of deployment include scaling up the solution in Sub-Saharan Africa and globally.	Service delivery, Health information system
DHIS2 for COVID-19 Surveillance: South Africa Rebecca Potter, covid@dhis2.org	South Africa	08/05/2020	South Africa customized their DHIS2 system for Covid-19 surveillance with assistance from local partners	Health information system

HIV Solution powered by Vantage Todd Malone, Todd.Malone@brhc.com	South Africa	01/09/2019	Vantage is currently an integral part of BroadReach Consulting’s support within USAID’s Accelerating Program Achievements to Control the Epidemic (APACE) program in South Africa. BroadReach, with Vantage, assists governments and implementing partner organizations to improve individual and population health outcomes at scale. Vantage is a 4th Industrial Revolution technology platform which leverages cloud computing, Artificial Intelligence and advanced data analytics to help governments and organizations use their next dollar and the next hour more effectively. Vantage provides:<br>• Powerful on-demand insights (decision support) for all stakeholders<br>• Vastly improved management, governance, transparency and accountability<br>• The ability to manage large workforces more effectively and at scale<br>• A means to rapidly scale up good and best practices while assuring quality<br>• Radical reduction in the time to develop, disseminate and digest reports, giving managers more time to focus on priorities.	Health information system
COVID 19 - Safe Entry Management Kent Perils, kent@bcpi.co.za	South Africa	03/10/2020	We offer a COVID -19 Safe Entry Management digital solution for the workplace, office, factory, venue, or any place of gathering. Our mobile application includes: Self Screening through health diagnosis including comorbidity diagnosis, Safe Entry Management - This allows a user safe entry into a venue displaying no signs or symptoms of the COVID-19, Case Management - This is a dashboard for Business Managers, HR Departments, RISK and SHE Departments to manage all employee related positive cases for Self Isolation, and Quarantine .	Health informaiton system

Vula Mobile William Mapham, william@vulamobile.com	South Africa	24/11/2021	Vula Mobile connects frontline health workers with specialists. We provide a safe and secure platform to refer patients and share advice	Service delivery
eLQAS: Collecting real-time polio vaccination data Yaw Anokwa, yanokwa@nafundi.com	South Sudan	01/11/2014	eLQAS is an electronic version of LQAS that uses phones and tablets to improve rapid monitoring of polio vaccination. Implemented as a free and open-source form in Open Data Kit, eLQAS enables real time results, increased data quality, improved accountability, and easier implementation.	Health information system, Service delivery
e-Hospital - South Sudan Steven Wanyee, swanyee@intellisoftkenya.com	South Sudan	01/06/2019	eHospital is an end-to-end hospital information system that integrates medical records system, lab system, and ERP in a distribution that addresses the workflows, forms, and reports of the East African market. In addition to being an end-to-end facility system which captures fundamental patient touchpoints, eHospital is a distribution of Bahmni. This essentially means that eHospital leverages the software excellence and technical architecture of a globally proven system backed by a strong community committed to the continuous development of the product.	Service delivery

Integrated Humanitarian Data Package for public health data needs Jonathan Hanson, jhanson@mapaction.org	South Sudan	01/04/2021	The underlying issue we address is the time, cost and expertise needed to select and prepare geographic datasets for vaccine planning and delivery. Most of the geographic tools, analyses and services being developed for Covid vaccine planning and delivery are underpinned geographic datasets, particularly relating to target population, health facilities, and logistics. But sourcing and preparing suitable data remains a significant bottleneck. One excellent project – analysing accessibility to health facilities in South East Asia – reported to the Covax GIS GIS Working Group that "it still takes 70-80% of an entire project's time to address data-related issues to conduct this type of analysis". This figure of around 75% is widely reported in projects reliant on data for mapping, analysis and services. With many partners typically involved in each country's National Deployment and Vaccination Plan, this effort will be duplicated many times, resulting in additional cost, time delays, capacity barriers, and inconsistencies. In contexts where datasets are typically out of date, incomplete, or unreliable, the problem becomes especially pronounced.<br><br>We also see the integrated data package model as having wider application in public health and humanitarian contexts, being scalable to different geographies and adaptable to different use cases.	Service delivery

Improving Healthcare Capacity and Access Using THINKMD's Clinical Decision Support Tool in a Humanitarian Setting Meg McLaughlin, mmclaughlin@thinkmd.org	Sudan	01/05/2019	The MEDSINC platform is currently being implemented by Medical Assistants working in community clinics within East and West Darfur. Medical Assistants are able to triage and treat incoming patients. Through the use of THINKMD's platform, Medical Assistants are able to automatically record all patient interaction; automatically generate triage, treatment and follow-up recommendations; monitor their workforce; monitor each facilities' patient flow; track population health; and report to the Ministry of Health.	Service delivery, Health information system
NEMO Andrew Nute, andrew.nute@cartercenter.org	Sudan	26/06/2018	NEMO is a server software that provides a user-friendly portal accessible as a website through a web browser. When logged into the server, the user can design survey forms using a simple and intuitive process that does not require an understanding of XML code (interpretteded by Open Data Kit Collect). The user then can publish the forms as XML to make them accessible to Android tablets that connect to the NEMO server using the administratively assigned secure credentials of the tablet user. After collection and submission of completed survey responses, the NEMO portal also allows for aggregating response data to build and export data sets and generate reports that can be saved in the user portal like a dashboard.	Health information system
DHIS2 for COVID-19 Surveillance: Sudan (in development) Rebecca Potter, covid@dhis2.org	Sudan	08/05/2020	The DHIS2 digital data package for COVID-19 is designed to accelerate case detection, situation reporting, active surveillance and response in countries. The COVID-19 digital data package includes standard metadata aligned with the WHO's technical guidance on COVID-19 surveillance and has been adapted to local country context and language in this implementation.	Health informaiton system

eLMIS Facility Edition (FE) Ashraf Islam, ashraf_islam@jsi.com	Swaziland		eLMIS FE has recently been upgraded from a Java Swing based client server application to a web-based two tier application with a PostgreSQL database back-end. At each health facility implementation, it works on their local area network (LAN) to enable multiple user access to the same application. It supports off-line access to it from within the LAN, without having to use internet, since it is a local installation within the facility. It can produce a monthly report and requisition (R&R).	Service delivery, access to medicine, vaccines and technology
The Safe Delivery App in Togo (part of DI 2018 Project) Lauren Bellhouse, lauren@maternity.dk	Togo	01/08/2018	As part of the larger DI 2018 Project of PlanBornefonden, the French Language Safe Delivery App was rolled out as a job aid for skilled birth attendants in Haho prefecture. A TOT took place in Haho for 6 trainers and district officials in August, 2018; baseline knowledge and confidence surveys were conducted Sept 2018. As of Feb 2020, user data confirms a solid uptake of the App in in-service settings (over 350 downloads nationally), with a heavy concentration in the Plan International project district (Haho) as well as use in Lomé and Kara, where partner organizations who have participated in Maternity Foundation/ Plan International trainings are based. For the pilot project of 47 midwives in Haho, at end-line the results indicated that the SDA has had a positive effect on clinical knowledge and confidence from baseline.	Service delivery

Digital Tools for CHWs with Integrate Health and Medic Mobile Fatou Fall, fatou@medicmobile.org	Togo	01/02/2017	Integrate Health (IH) and Medic Mobile co-designed a mobile application to support CHWs to deliver equitable and quality doorstep care. Building on IH's model that aim to save lives in neglected communities the application supports 27 CHWs in decision making (referral, diagnosis, treatment), targeted follow ups for closed loop care, and offline data collection. The technology links care provided at doorstep and health facility levels through a unique patient ID system, tracks longitudinally care provided, enhances data transmission and analysis for evidence based decision making. At the facility level, the health workers are supported to log services provided to the patients for ease of follow up at CHW level. The technology will evolve to be fit the model iterations. Data will be used to inform iterations at functionality, workflow and features levels to support fit-for-purpose.	Service delivery
Togo Health Analytics Platform (THAP) Quentin Perrot, quentin@zenysis.com	Togo	01/03/2018	THAP was a platform developed by Zenysis Technologies and led by the Togo Ministry of Health that integrates data from a number of siloed systems. By joining data on routine health, inventory and health campaign, THAP helped the Togolese Ministry of Health and Social Protection's National Malaria Program (PNLP) better understand malaria transmission patterns, target interventions to reduce malaria disease burden and identify supply chain inefficiencies.	Health information system

Télédermatologie TOGO Palokinam PITCHE, vincent.pitche@gmail.com	Togo	01/06/2018	In our project we use provider to provider telemedecine to support primary health workers who have previously been trained to manage infectious skin conditions like impetigo, scabies fungus infections. Then if they can solve a kind of dermatosis they use Bogou Platform to send this case. Bogou is the telexpertise platform lead by the "Reseau en Afrique Francophone pour la Télémédecine" RAFT. Our project intend to address the needs of dermatological care in rural areas of 4/6 health regions in the whole country where there is no dermatologist. The aims are quality and accessibility of dermatological care to individuals living in remote areas of Togo. we started this project since june 2018 and launched the use of Bogou on may 2019 in 20 pilot health care facilities. If the first assessment is positive we will generalized in 30 others	Service delivery
mTrac Sean Blaschke, sblaschke@unicef.org	Uganda	01/10/2011	mTrac is part of the MoH's national eHMIS system, integrated into DHIS2 and iHRIS. It allows submission of Weekly HMIS Surveillance Forms by SMS, alerts to predefined user groups based on notifiable events or data outliers, communications / surveys / polls initiated by government staff at the National and District level to all Health Workers, and health care consumers to report on their experiences as well as any Service delivery issues (stock-outs of commodities, extortion, etc).	Health information system
UgandaEMR (OpenMRS) Jonathan Mpango, jmpango@musph.ac.ug	Uganda	01/12/2015	Health facility patient level electronic medical records system	Health information system, Service delivery

Improving health worker adherence to IPTp provision guidelines through sending text messages Christian Rassi, c.rassi@malariaconsortium.org	Uganda	01/01/2015	This pilot study explored the feasibility and acceptability of an mHealth intervention which involved complementing classroom training with sending educational text messages to health workers. Training and text messages were designed to improve health workers' knowledge of and adherence to national guidelines for the provision of intermittent preventive treatment of malaria in pregnancy (IPTp). Evaluation of the pilot is complete and we are currently exploring options for scale-up.	Human resources for health
: Improving outcomes in HIV patients usingmobile phone based interactive software support Rosalind Parkes Ratanshi, rp549@medschl.cam.ac.uk	Uganda	01/12/2016	Ugandan Academy for Health Innovation & Impact is implementing an open source software-based tool to send messages and interactive voice response to people living with HIV, using the MoTeCh Software developed by Janssen & Janssen; previously used in India " Connect for Life" as a mobile technology for community health . The technology offers:Clinic appointment reminders, Supports weekly self-reported adherence, Weekly health info-tips,+/- Symptom reporting.	Service delivery
ART ACCESS Dr Rosalind Parkes-Ratanshi, rp549@medschl.cam.ac.uk	Uganda	01/01/2018	We are developing a pilot mHealth tool (ART Access App) to link patient information on ART and viral load to an algorithm which guides a community pharmacy on ART delivery without the need for the stable patients to go to the health facilities for drug refills. The application will capture the patients' demographics, unique identifier, current ART regimen, current viral load, treatment adherence score, clinical complaints, follow-up outcomes and reasons for missing appointments.	Service delivery

Connect for Life - Infectious Disease Institute (IDI) Theresa Pattery, tpattery@its.jnj.com	Uganda	01/07/2015	Attendance at clinic appointments, patient-support tools (daily pill reminder and health tip on topics covering adherence, nutrition, healthy lifestyle with disease-related information), supportive management of medication side-effects along with HIV knowledge using CfL is an effective predictor of virological outcomes positively impacting quality of life. This is demonstrated with CfL Randomized Controlled Trial & CfL Lite study (weekly support for ART-stable, undetectable viral load patients).	Service delivery
Duty Roster and Attendance Tracking Mobile Application Dennis Kibiye, kibsden@gmail.com	Uganda	01/07/2018	The absenteeism rate of public health workers was too high in Uganda, about 47% by end of FY 2015, it was agreed that attendance to duty should start being tracked so as to reduce this rate, because it had led to poor Service delivery and low outputs, however after development of the attendance module in a Human resource Management system, it was noted that the duty roster to schedule staff for duty had to be implemented as well but many facilities lacked computers to implement this system, so there was need to develop a mobile application to help lower level facilities access the duty roster and attendance tracking system Intrahealth International in conjunction with the Ministry of health Uganda designed a mobile application that would help facility in charges and district leadership to address the issue of tracking and reporting on absenteeism on a monthly basis for each public health worker. The results of the initiative have been positive in a way that currently the absenteeism rate has reduced to 11%	Human resources for health

STRE@MLINE Mr Samuel Mugisha, CEO Innovation Streams Ltd, samuel@innovationstreams.tech	Uganda	01/01/2015	Stre@mline is an exciting, unique integrated patient-centred software program incorporating key patient safety features e.g. WHO Emergency Triage, Assessment & Treatment (ETAT) tool, clinical prompts and immediate access to specific WHO and national guidelines. Stre@mline includes patient records, prescribing, audit, stock control, billing, HMIS reports and follows the full patient journey. Stre@mline incorporates health insurance membership demographics allowing real time analysis of usage of the scheme. See www.streamlinehealth.org	Health informaiton system, Service delivery
REFERENCE LABORATORY INFORMATION SYSTEMS (EID/VL LIMS) PROSCOVIA NAMBUYA MBABAZI, pronam2000@gmail.com	Uganda	03/03/2014	This system is used for data capture, processing and generation of Lab Test results for Viral Load and EID programs at UNHLS- CPHL.	Service delivery

Access to Infant and Maternal (AIM) Health Plus Uganda Racheal Auma, racheal_auma@wvi.org	Uganda	01/01/2017	Supported by Irish Aid, the AIM Health Plus project addresses the leading causes of maternal and neonatal mortality and improves young child survival and nutritional status across four countries in Africa with an emphasis on promoting behaviour change at the household level. The Ugandan deployment of the AIM Health Plus project, centred in Busia district, has included a digital health component since its inception in 2017.<br><br>The AIM Health Plus project provides village health teams (VHTs) with smartphones equipped with Dimagi's CommCare application to use during their home visits. The application supports VHTs who use the Timed and Targeted Counselling (ttC) approach to promote health and nutrition behaviour change among pregnant women and among mothers or caregivers of children under 2 years of age. The application reminds VHTs to visit homes during the pregnancy, infancy and childhood stages and also supports VHTs as they conduct counselling sessions, enabling them to submit community health data in near real time. This digital health intervention is expected to improve the effectiveness of the communication delivered to women and caregivers as well as strengthen the health system through better use of community-level data.	Service delivery

SmartHealth App in Uganda Nii Amon Dsane, ndsane@livinggoods.org	Uganda	01/05/2016	Living Goods and Medic Mobile collaborated to create a powerful set of mobile and web tools to support the Community Health Workers (CHWs) assisted by Living Goods. Built on Community Health Toolkit Framework core, the open-source Smart Health app is used by CHWs to support delivery of high quality and integrated primary health care services, including common childhood diseases such as malaria, diarrhea and pneumonia; maternal and neonatal care, family planning and immunization referral services. Although Living Goods independently configured its immunization and COVID-19 workflows, it still actively collaborates with Medic Mobile for ongoing technical support, including needs assessments, technology strategy and upgrades. The mobile application also has a supervisor dashboard that enables the real-time remote management of CHW's, also helps manage effective stocking of medical commodities, and is being used to provide government with critical data to better plan and budget for community-level interventions	Service delivery, Health information system
MNCH mobile content with nutrition focus, available in 5 languages in SMS and voice transcript format Kim Viljoen, kviljoen@gsma.com	Uganda	01/09/2013	The GSMA, together with its global content consortium lead by CABI, developed open-source nutrition and MNCH mobile ready content for Uganda available in Luganda, Rukiga, Atesot, Langi and English. All content was produced by in-country specialists and is based on recommended MNCH and local nutrition practices. The Ministry of Health played a vital role in the development and content validation. The Content is available in the form of: topic-specific factsheets, SMSs and voice message transcripts.	Service delivery

NoviGuide: Tablet-based clinical decision support bringing neonatal expertise to the bedside while uncovering barriers to care Joshua Bress, josh.bress@globalstrategies.org	Uganda	01/03/2017	NoviGuide is a tablet-based companion for clinicians who care for newborns. Quick, visually engaging, and fully-featured, NoviGuide's dynamic decision trees support providers through a care encounter, expanding lines of questioning in response to danger signs. Complex neonatal care protocols become second nature, with error-prone manual calculations a thing of the past. Babies get rapid, precise care. Care delivery then combines with data collection. Each encounter helps build a picture of the care landscape, data syncing later if connectivity is limited. An Insights Dashboard provides lively visualizations and identifies problem areas, and can connect to a DHIS2 instance. NoviGuide was developed through a co-design process involving neonatal specialists, neonatal pharmacists, software developers, and frontline bedside clinicians providing care in low-resource settings. Following extensive studies in KwaZulu-Natal, South Africa, and Tororo, Uganda, it is ready to serve new clinics in new geographies.	Service delivery
The Zone Raquel Palomino Gonzalez, lund@unfpa.org	Uganda	01/04/2017	An ICT platform providing integrated information on life skills as well as SRH, HIV and STI prevention and other health issues. The platform currently comprises of a website, and work is ongoing to make the information accessible through mobile phones. In addition, Global Mobile will be installed on 300 digital drums (heavy duty, durable community computers), which will be placed across the county in areas with low access to technology and internet. Strong evidence suggests that a significant link exists between income generation and SRH outcomes of young people, therefore an aspect of savings and business development has been incorporated. The Zone offers as well a platform to track quality of services delivered to young people, and easy	Service delivery

			referrals to service providers at healthcare facilities, as well as to counsellors through SMS and hotline linkages.	
Bringing Back Mothers and Babies Kisaakye Linda, lkisaakye@gmail.com	Uganda	02/09/2019	Aim is to improve retention in the PMTCT program, Users; Midwives, Records Persons; PMTCT program stakeholders, System was deployed to monitor monthly rentention of HIV mothers and babies.	Health information system, Service delivery
DREAMS/OVC Juliet Cheptoris, julietcheptoris@gmail.com	Uganda	16/06/2020	Track services provided to AGYWs and OVCs , Traget users: AGYWs, CSOs, IPs,DCDOs, MOH, Development Partners.	Health information system, Service delivery
Early Infant Diagnosis(EID) Dashboard Dr. Victor Bigira, vbigira@musph.ac.ug	Uganda	16/06/2020	openmrs	Health information system, Service delivery
HiMap Mobile Application Jonathan Mpango, jmpango@musph.ac.ug	Uganda	16/06/2020	Capture GIS coordinates of health facilities and district health offices	Service delivery
RxSolution Stephanie Xueref, smxueref@msh.org	Uganda	09/01/2014	RxSolution is an integrated computerized pharmaceutical management system used to support logistics and stock management of medicines and health supplies in public and private not for profit health facilities in Uganda. The system is used by facility stores personnel to manage medicines & health supplies inventory and is currently implemented in 250 higher level health facilities throughout the country. Plans are underway to link the system to the new national medical stores ERP to facilitate electronic ordering of commodities between facilities and NMS.	Access to medicines, vaccines and technology, Service delivery

Pharmaceutical Information Portal Stephanie Xueref, smxueref@msh.org	Uganda	09/01/2014	The Pharmaceutical Information Portal (PIP) is a data warehouse and business intelligence system developed to provide strategic insights into the performance of the public sector medicines supply chain in Uganda. The system is designed to inform evidence based decision making and collects data from multiple sources such as RxSolution health facility inventory data, district medicines supervision, performance assessment and recognition strategy (SPARS) data, pharmaceutical financial management data and data on key commodities from central medical warehouses. Users of the system are supply chain managers at national and sub-national levels. A national emergency logistics management information system is currently being integrated within the PIP to inform pre-positioning of supplies for epidemic disease outbreaks.	Access to medicines, vaccines and technology, Service delivery
Option B+ Kisaakye Linda, lkisaakye@gmail.com	Uganda	09/12/2012	Aim is to monitor progress on the PMTCT program, Users; Midwives, Records Persons; PMTCT program stakeholders, System was to deployed weekly monitor the rollout and implementation of the Option B+ program.	Health information system, Service delivery

integrated Human Resource Information System (iHRIS) Ismail Wadembere, iwadembere@intrahealth.org	Uganda	16/06/2020	A strong human resources information system (HRIS) enables leaders to quickly answer the key policy and management questions affecting Service delivery. The integrated Human Resource Information System (iHRIS) suite, a free Open Source HRIS is management tool to supply leaders with the information they need to assess HR problems, plan effective interventions and evaluate those interventions. It is a computerized Human Resources management tool consisting of electronic databases for storing, reporting, and analyzing data from the time professionals enter pre-service training to when they leave the workforce to ensure timely availability of accurate information for policy, planning and management. The iHRIS suite consist of iHRIS Plan designed to support the key HRH functions of planning; iHRIS Train to support pre and in-service training; iHRIS Qualify for regulation of registration and licensure; iHRIS Manage for management and iHRIS Retain for retention of the health workforce.	Human resources for health
Smart Hospital Information System (SmartHIS) Dr Simon Ndira, simon.ndira@compelling.works	Uganda	16/06/2020	SmartHIS is an integrated hospital information system, a revival of the eHMIS project that was tested in Tororo District Hospital as part of a research project in 2004. The aim is to come up with a free light-weight hospital information system covering all processes in a hospital	Service delivery, Health Informaiton system

Performance Tracking App for CHW Supervisors Simon Mutama, smutama@livinggoods.org	Uganda	18/09/2015	The LG Performance Tracker App for CHW Supervisors is an Android application with functionality for monitoring the performance of CHWs enrolled with LG and actively using the LG Smart-Health App. Supervisors have access to the performance metrics of the CHWs garnered through the LG Smart-Health Application and are able to record their own observations of CHW behaviour, activity and aptitude to paint a complete picture of performance of any given CHW. Within the Performance Tracking app, a supervisor has access to and can update information on CHW Stock status, Aptitude on conducting Sick Child accessments, Pregnancy Registration and Follow-ups, Newborn health checks and House Hold Registrations and interactions. The app also access the CHWs competence with conducting public health education	Service delivery, Health information system
mTrac Uganda Carol Kyozira, ckyozira@gmail.com	Uganda	11/05/2020	Medicines Mobile Tracking	Service delviery, Access to medicines, vaccines and technology
Strategic Information Mobile Apllication Jonathan Mpango, vbigira@musph.ac.ug	Uganda	10/01/2013	Track and monitor the implementation of the national Option B+ program	Health information system
Viral Load Dashboard Dr. Victor Bigira, vbigira@musph.ac.ug	Uganda	08/04/2014	To track the 90-90-90 at National, District, Hub & facility. Also tracking of an individual patient is on	Health information system

FamilyConnect Dr. Jesca Nsungwa Sabiiti, jnsabiiti@gmail.com	Uganda	16/06/2020	FamilyConnect is an SMS and USSD tool which is being rolled out to support the implementation of the Key Family Care Practices and the new WHO ANC model, improving health, sanitation, nutrition and other practices, as well as health seeking behaviour, among pregnant and lactating mothers. The tool enrolls VHTs to register pregnant women and women of reproductive age (15-49). Midwives and nurses can also be enrolled to register women. MoH through the Community Health department is also designing FamilyConnect to support Catchment Area Mapping, Planning and Action (CAPA) with VHT SMS reporting on HMIS097. Under FamilyConnect-EMTCT module, pregnant HIV positive mothers are registered by health workers to get PMTCT messages and appointment reminders. The FamilyConnect-NCD module targets patients with heart failure. The patients are registered by health workers to be able to input their heart failure symptoms for tailored advice in form of SMS as well as feedback on when to seek care.	Health information system, Service delivery
NTD LF pre-TAS and TAS survey Clara Burgert, cburgert@rti.org	Uganda	01/06/2017	Use of Secure Data Kit platform to collect disease prevalence survey information for LF pre-TAS and TAS surveys.	Health information system
Stre@mline Samuel Mugisha, samuel@streamlinehealth.org	Uganda	10/04/2014	Stre@mline is an e-health platform that enables clinicians in resource poor settings to deliver health care efficiently by providing key patient safety prompts across the entire patient journey. Stre@mline follows the patient journey incorporating demographics, triage, consultation, patient safety, investigation ordering and results, prescriptions, stock control of medicines, insurance, finances and with the ability to generate statistical data needed for monthly government Health Management Information System reports (HMIS), graphical trend data, etc.	Service delivery, Health information system

Open Client Registry Wayan Vota, wvota@intrahealth.org	Uganda	20/08/2019	IntraHealth developed OpenCR, a prototypical open source client registry with USAID funding through MEASURE Evaluation. Client information is distributed across multiple Health information systems (facilities, pharmacies, lab systems, etc.). OpenCR was designed to uniquely identify individuals who have records in multiple information systems to help countries track patients through the continuum of care. Development was informed by stakeholders in Uganda, including the Ministry of Health (MOH) and the Central Public Health Laboratory (CPHL), as well as technical teams at CDC and USAID. OpenCR was built to support epidemic control by facilitating the deduplication of patients' lab test results for tracking outcomes over time and identifying those lost to follow up.	Health information system, Service delivery
DHIS2 for COVID-19 Surveillance: Uganda Rebecca Potter, covid@dhis2.org	Uganda	08/05/2020	The DHIS2 digital data package for COVID-19 is designed to accelerate case detection, situation reporting, active surveillance and response in countries. The COVID-19 digital data package includes standard metadata aligned with the WHO's technical guidance on COVID-19 surveillance and has been adapted to local country context and language in this implementation.	Health information system
USAID RHITES-N, Lango Med Makumbi, med_makumbi@ug.jsi.com	Uganda	04/11/2020	Digital platforms used for targeted client communication including reminders for health facility appointments for ANC, eMTCT, VMMC & PLHIV. Health information dissemination via mobile SMS on program thematic areas and Toll free hotline and WhatsApp platforms for remote consultations.	Service delivery

Pfizer Project Bugiri Elisha Nangosha, elisha_nangosha@wvi.org	Uganda	01/04/2020	By increasing uptake of curative and preventive interventions, Pfizer Project Bugiri's goal is to reduce mortality among children under 5 from common childhood illnesses in Uganda's Bugiri district. To accomplish this, the project is focused on achieving three outcomes: improved demand for and uptake of community-based child health services; strengthened systems and structures that support child health interventions, including the supply chain and strengthened child health information. Trained village health teams (VHTs) address care-seeking behaviours of local child caretakers to increase demand for community health services. VHTs also influence social norms through faith and community leaders and by employing a socially accountable community engagement strategy. The project focuses on supportive supervision and on-site video distance learning using open-source content furnished by ORB. VHTs and health workers gain skills that they need to successfully implement and support Integrated Community Case Management, including at level II health facilities where Integrated Management of Newborn and Childhood Illness is provided. These service-quality enhancements reinforce child healthcare systems. Additionally, the project focuses on improved use of health information and supply chain management systems by enabling the tracking of medications and materials/equipment used. VHTs use CommCare to implement, track and store information about treatment outreach and supplies. The data supports and informs healthcare decision-making at all levels by also integrating with the government's existing District Health information system (DHIS2).	Service delivery

Buikwe Maternal Newborn and Child Health (B-MNCH) Project Angella Akolla, angella_akolla@wvi.org	Uganda	01/04/2019	B-MNCH works to improve maternal, newborn and child health in Uganda's Buikwe district by 2021. To achieve this, it is improving and integrating household access and utilisation of MNCH services at both the community and facility levels. B-MNCH uses behaviour change communication, capacity building, empowerment of local health committees and social accountability to encourage and facilitate change among boys and girls ages 0–5 and men and women ages 15–49. District-wide community health workers' (CHW) use of CommCare helps them to effectively follow up and deliver counselling messages to households with pregnant and lactating mothers. Data utilisation is increased even further by health facilities using both CommCare and DHIS2. Supported by World Vision Hong Kong and collaborating with multiple stakeholders at the local and national levels, B-MNCH is in the process of scaling up so that it can help influence other communities to enact these same improvements.	Service delivery, Health information system
Kampala Emergency Digital Emergency System Douglas Akii Bua, dbua@psiug.org	Uganda	22/03/2021	To test innovative approaches to address the demand and supply side barriers affecting care seeking, effective referral and provision of quality care equitably for better maternal and newborn health in slum settings in Kampala	Service delivery
Electronic Inventory Management Services System (e-IMSS) Cliton Chikwata, clinton.chikwata@tmcg.co.ug	Uganda	01/08/2020	An electronic management inventory services system that is accessed online by a facility and allows for<br>a. Inventory management for the health facility<br>b. Facilitates warehouse-to-site supply distribution between health facilities<br>c. Ability to manage individualized purchases/orders<br>d. Caters for API integration with other electronic management records systems and online eShop platforms<br>e. Ability to generate sales reports	Access to medicines, vaccines and technology, Service delivery

Public Health Open Community Mapping Kiggudde Deogratius, deogratias@mapuganda.org	Uganda	11/12/2019	OpenStreetMap Uganda (OSM Uganda) in collaboration with Humanitarian OpenStreetMap Team (HOT) in an effort to improve patient tracing in rural Uganda decided to develop community landmark maps and gazetteers to assist community health workers easily navigating to patients as well as facility-based health workers to trace origins of their patients. OSM Uganda deployed the community system based on learning from previous public health outbreaks of cholera where some of the facility-based health workers had difficulty in tracing the origin of their patients and in turn made it difficult to identify the source of the outbreak.Using the OpenStreetMap remote editing tools, OSM Uganda in collaboration with HOT remote mapped all the buildings, roads, and place names in Kisoro and Zombo districts. Community health workers were then trained on how to use maps.me and OSMand to map and share locations all community landmark features that assist one to navigate to a community. Using QGIS, community landmark maps and gazetteers were developed and shared to all community health workers to use to navigate between communities and for facility-based workers to trace the origins of thier patients.. In the future we would like to intergrate the information collected from the field with the DHIS2 platform with the department of health information at the Ministry of Health. Using OSM overpass turbo API the team would build live link with the DHIS2 plaform that will allow district and national stakeholders overlay community data collected uploaded to OpenStreetMap with health data to get a much deeper insight to how to effectively plan and execute public health interventions.	Service delivery, Health information system

OpenONCIMS Dr Wasswa William, williamwasswa036@gmail.com	Uganda	01/11/2019	OpenONCIMS is an Integrated Oncology Information Management system that leverages Data Science and AI for the improvement of cancer diagnosis and management in resource-constrained areas.	Service delivery
Vaccine Information Management System (VIMS) Ssanyu Nyinondi, snyinondi@tz.jsi.com	United Republic of Tanzania	09/01/2018	Tanzania's Vaccine Information Management System (VIMS) combines vaccine data management, stock management and cold chain equipment inventory management into one tool that facilitates monitoring and improving immunization program performance. The prior system used the district vaccine data management tool (DVDMT), stock management tool (SMT) and cold chain equipment inventory management tool (CCEIT)	Service delivery, Access to medicine, vaccine and Technology
Health Information Mediator Ssanyu Nyinondi, ssanyu_nyinondi@jsi.com	United Republic of Tanzania	09/01/2018	This "middleware" in the Tanzania Health Information Architecture enables data exchange from/between 9 Health information systems from 5 national and specialized hospitals and legacy systems through an interoperability layer (JEEVA, MedPro and Care2x, eLMIS, DHIS2, VIMS, HRHIS, HFR and EPICOR 9)	Health information system
Safer Deliveries Rachel Hofmann, rhofmann@d-tree.org	United Republic of Tanzania	07/05/2015	The Safer Deliveries program equips community health volunteers (CHVs) with digital tools to promote maternal and newborn health. CHVs use a mobile application to guide them in assisting families to plan for facility deliveries and counsel, screen, and refer pregnant women, newborns, and postpartum women to facilities if needed. The program also builds the capacity of the Ministry of Health to utilize program data, generated as a by-product of CHV visits, to improve decision-making.	Service delivery

Mobilizing Maternal Health (MMH) Gloria Kahamba, gkahamba@d-tree.org	United Republic of Tanzania	15/08/2014	A dispatch center with a 24-hour toll-free emergency line and an emergency referral system. The system uses ambulances and community drivers with payments automatically sent through mobile money. A mobile application supports dispatchers to determine maternal and neonatal emergencies and arrange transport to the most appropriate facility. The system reimburses women to travel to a maternity waiting hotel before delivery, and Community Health Workers support women through pregnancy and the postpartum period.	Service delivery
Community Family Planning and Model Household Systems; Population Health Environment (PHE) Rebecca Litner, rlitner@d-tree.org	United Republic of Tanzania	01/10/2017	Community-based family planning and model household systems to support population, health and environment initiatives. The family planning system empowers both community health workers and supervisors in the facility, and was developed based on Balanced Counseling Strategy plus (BCS +) guidelines. Community health workers use a mobile application to evaluate family planning needs of clients and distribute or refer for methods. Supervisors in the facility use a mobile application to follow up with community health workers and also evaluate and communicate about method stock at the facility. The family planning system is integrated with Pathfinder's DHIS to automate reporting activities. The Model Household system is used by Population Health and Environment (PHE) champions, who evaluate households based on 75 criteria. PHE champions then identify weak areas and establish action items with the health workers, and track household improvement over time.	Service delivery, Health information system

Sauti- HIV Prevention Program Gloria Kahamba, gkahamba@d-tree.org	United Republic of Tanzania	29/11/2015	HIV prevention program targeting key and vulnerable populations. Decision support tools guide (i) counselors at mobile clinics to conduct risk assessment; HIV testing and counseling; and STI, TB, and GBV screening and (ii) peer educators to conduct behavioral change communication sessions and financial education in communities. Clients are tracked with a unique QR code, given upon registration and referral. The system provides quality data reporting and program monitoring.	Service delivery, Health information system
Family Planning Digital Health System for Community Health Workers- Mara Region with Shirati KMT Hospital Gloria Kahamba, gkahamba@d-tree.org	United Republic of Tanzania	01/07/2017	Community-based family planning (FP) application with algorithm that supports Community Health Workers (CHWs) providing FP services in the community, following the Balanced Counseling Strategy Plus and Tanzania guidelines. The system includes comprehensive dashboards, summarizing key program indicators and government reports, for program managers to monitor and use real-time data for decision making.	Health information system
MCSP Faclity and Community MCH application Steve Ollis, Steve.ollis@mcsprogram.org	United Republic of Tanzania	01/01/2016	Facility based decision support tool for use by health workers providing MNCH services and CHW decision support tool for community level workers providing MNCH services at the household level	Service delivery
BID INITIATIVE Hassan Mtenga, Laurie Warner, hmtenga@path.org	United Republic of Tanzania	01/05/2014	Patient-level-information-system developed to track children immunization appointment and history, and their well-being from birth through under five as well as track vaccine stock utilization and management. The solution is responding to critical challenges namely, incomplete or untimely reporting, inaccurate target population and lack of unique identifiers of infants to account for continuum of care and complexity of data tools. The solution is deployed in primary health care level.	Service delivery, Health information system

Non-discriminating access for Digital Inclusion (DigI) Christine Holst, christine.holst@medisin.uio.no	United Republic of Tanzania	01/01/2017	Non-Discriminating Access for Digital Inclusion (DigI) is a multi-disciplinary innovation project funded by the Norwegian Research Council and The Norwegian Ministry for Foreign Affairs. This pilot project aims to install small hotspots in rural Tanzania with a Wi-Fi (50 meter radius), providing free access to an "Internet lite"-version and high quality basic health information, via fixed tablets or clients smartphones.	Service delivery
MNCH mobile content with nutrition focus, available in Kiswahili and English in SMS and voice transcript format Mojca Cargo, mcargo@gsma.com	United Republic of Tanzania	02/09/2013	The GSMA, together with its global content consortium lead by CABI, developed open-source nutrition and MNCH mobile ready content for Tanzania available in Kiswahili and English. All content was produced by in-country specialists and is based on recommended MNCH and local nutrition practices. The Tanzania Food and Nutrition Centre (TNFC) played a vital role in the development and content validation. The Content is available in the form of: topic-specific factsheets, SMSs and voice message transcripts.	Service delivery
The Safe Delivery App- Tanzania Helena Lassen, helena@maternity.dk	United Republic of Tanzania	15/06/2015	IFAKARA implemented the App in partnership with and Bluetown in Dodoma. The App was reviewed and approved by the MoH and is available in this aligned version, with plans for a Swahili version in the works. In October 2018 MF held a Safe Delivery App training in Arusha where 19 nurses and midwifes from 8 different hospitals in Arusha participated in a 3-day training.	Service delivery,

USAID BORESHA AFYA -COMMUNITY Health information system ENCOMPASSING OPEN SMART REGISTER PLATFORM, CLOSED USER GROUP & ELEARNING Dunstan Bishanga, Dunstan.Bishanga@jhpiego.org	United Republic of Tanzania	01/10/2017	USAID Boresha Afya Lake and Western Zone is a five-year project implemented in seven regions of Tanzania Mainland and Zanzibar. The goal of the project is to improve the health status of all Tanzanians - with a focus on women, youth and children - by improving the availability of, and access to, quality, respectful and integrated health services. The project is implemented by a consortium of three partners, led by Jhpiego, an affiliate of Johns Hopkins University. The other partners are EngenderHealth and PATH. The project digital health component includes Digital Learning to improve performance of Healthcare providers, Emergency Transport and referral system to improve maternal survival, OpenSRP maintains client data, automates healthcare and provides a foundation for the continuum of care and GIS for Operational Planning	Service delivery, Health information system
SAUTI PROJECT-KVP MOBILE DATA COLLECTION SYSTEM Dr Albert Komba, Albert.Komba@jhpiego.org	United Republic of Tanzania	01/10/2015	Funded by the U.S. President's Emergency Plan for AIDS Relief through the U.S. Agency for International Development, Sauti Project is working in partnership with the Tanzanian Ministry of Health, Community Development, Gender, Elderly and Children to bring community-based HIV and reproductive health services close to key and vulnerable populations (KVPs) in 12 regions. The populations reached by Sauti Project include KVPs as well as other Tanzanians disproportionately affected by HIV. The Sauti project digital health component include mobile data collection for access and efficiency increase	Service delivery, Health information system
Clinical Monitoring System for Preservice Trainees-More and Better Midwives for Rural Tanzania Project Dr Julius Masanika, Julius.Masanika@jhpiego.org	United Republic of Tanzania	01/10/2017	Commcare based Clinical Monitoring System for Preservice Trainees	Service delivery

Family Planning Digital Health System for Community Health Workers - Shinyanga Region Gloria Kahamba, gkahamba@d-tree.org	United Republic of Tanzania	01/01/2014	Community-based family planning program which support CHWs providing FP services in the community, following the Balanced Counseling Strategy Plus and Tanzania guidelines. Comprehensive dashboards, including government reports, discontinuation tracking and other indicators are available. CHW supervisors have a mobile app which allows them to send messages to CHWs about outreach events and method stock levels.	Service delivery, Health information system
Rheumatic Heart Disease and Hypertension Tracking Stefanie Kot, skot@d-tree.org	United Republic of Tanzania	01/03/2019	The hypertension tracking system has three key project objectives: 1) improve the health status of the existing facility-based health workforce as well as quality of patient care for hypertensive disorders through education and health promotion programs; 2) improve access to services for hypertensive disorders (including preeclampsia) across the continuum of care for pregnant and postpartum women as well as other patient populations; and 3) measure the cost effectiveness and health impact of a health workforce approach to hypertension prevention, management, and control. One track of the program focuses on providing services to HCWs. They will be screened at HCW fairs and enrolled into peer-to-peer support groups. The second track of program focuses on pregnant women. They will be screened during ANC and PNC visits in 21 Healthcare Facilities. There are plans to integrate Rheumatic Heart Disease tracking in the system in the future.	Service delivery, Human resources for health
Shang Ring Electronic Data Collection Alice Christensen, alice.christensen@jhpiego.org	United Republic of Tanzania	01/06/2018	Health providers have been given tablets with ODK installed, which they use to collect data electronically. The ODK App has an offline capability which help in centralizing electronic data collection even in places with limited internet connectivity.	Health information system

OpenLMIS Tanzania Rebecca Alban, Info@openlmis.org	United Republic of Tanzania	01/01/2013	In Tanzania, OpenLMIS provides end to end visibility of the supply chain for informed policy decision making – from national procurement planning to replenishing health facilities at the last mile. In 2015 the system was adapted to the needs of Zanzibar's autonomous health system. For both Tanzania mainland and Zanzibar, all products ordered by health facilities from MSD (mainland) or Central Medical Stores (CMS, Zanzibar) except for vaccines are managed with eLMIS. Vaccines are managed through a parallel supply chain.	Access to medicines, vaccines and technology, Service delivery
DYNAMIC Prof. Valérie D'Acremont, head of digital and global health at Unisanté, Lausanne or Dr. Irene Masanja, senior scientist at Ifakara Health Institute, Dar es Salaam, valerie.dacremont@swisstph.ch	United Republic of Tanzania	01/04/2019	We aim to improve quality of care for ~1'200'000 children 0-12 years in 70 health facilities through implementing, validating (impact on clinical outcome and antimalarial/antibiotic use) and further improving ePOCT+, an innovative clinical decision support algorithm (CDSA) combined with point-of-care diagnostic tests. ePOCT+ builds on the validated ePOCT tool, but will contain additional modules for data visualization, e-learning, supervision and self-audit. ePOCT+ will evolve through artificial intelligence, adapting care to environmental changes; machine learning, combined with constant medical evaluation, will tailor algorithms in real time, improving response to outbreaks and generating reporting for informed planning and targeting of interventions by health authorities. We will develop a framework at local, district, and national level for the scale-up of CDSA, investigate facilitating and impeding factors and create specific IT tools to allow health authorities to create their own medical algorithms.	Service delivery

ARTIFICIAL INTELLIGENCE IN SOCIAL MEDIA DURING CORONA VIRUS DISEASE OUTBREAK. DEOGRATIAS MZURIKWAO, deogratias@xsenseai.co.tz	United Republic of Tanzania	24/04/2020	Social media has become the main source of news online with more than 2.4 billion internet users, nearly 64.5 per cent receive breaking news from social media sites like Facebook, Twitter, YouTube, Snapchat and Instagram instead of traditional media. According to a social media analytic company, Sprinklr, it counted a record nearly 20 million mentions of coronavirus-related terms on March 11 with the US alone. This shows how much social media contain potential data about the outbreak. We aim to create an AI algorithm which can extract and analyse social media data related to coronavirus from social media in Tanzania to start with and cover more countries, region, continent and go global.	Health information system
DHIS2 for COVID-19 Surveillance: Zanzibar (in development) Rebecca Potter, covid@dhis2.org	United Republic of Tanzania	08/05/2020	The DHIS2 digital data package for COVID-19 is designed to accelerate case detection, situation reporting, active surveillance and response in countries. The COVID-19 digital data package includes standard metadata aligned with the WHO's technical guidance on COVID-19 surveillance and has been adapted to local country context and language in this implementation.	Health information system

Jamii ni Afya Heiko Hornung, hhornung@d-tree.org	United Republic of Tanzania	01/06/2018	The MOH Zanzibar and D-tree are partnering to implement Jamii ni Afya and the program has been formally adopted by the MOH as part of its 2020-2025 National Community Health Strategy. The MOH Zanzibar is training and deploying digitally enabled CHVs nationwide to provide home-based care and counseling for women and children under five on early child development (ECD), nutrition, and reproductive, maternal, newborn and child health (RMNCH). The digital platform is the foundation of the CHV program and includes a smartphone app, dashboards, and data integration into MoH systems. The app serves as a client tracking and job aid tool for CHVs, with the secondary purpose of collecting data to feed the reporting health management information system (HMIS). Data collected for client visits are automatically synced and aggregated for use in program monitoring and the national information system. Additionally, supervisors at health facilities can monitor CHVs' performance based on indicators.	Service delivery, Health Information system
Afya-Tek: Digital referral and decision support system using biometrics across the continuum of care Rebecca Litner, Rlitner@d-tree.org	United Republic of Tanzania	16/07/2020	The Afya Tek program aims to improve coordination among providers in order to strengthen the primary care system and ensure quality care is accessible to all. Afya Tek is a digital care and referral system connecting ADDOs, Health Facilities, and Community Health Workers around maternal, child and adolescent health. The system enables comprehensive case management with decision support and referral tracking across the three providers to enable appropriate and timely follow up care.	Service delivery

Access to Infant and Maternal (AIM) Health Plus Tanzania Daudi Sosthen Gambo, daudi_gambo@wvi.org	United Republic of Tanzania	01/01/2019	With the support of Irish Aid, the AIM Health Plus project addresses the leading causes of maternal and neonatal mortality and improves young child survival and nutritional status across four countries in Africa. Promoting behaviour change at the household level has been the emphasis. In Tanzania, the AIM Health Plus project has included a digital health component since 2019. The project provides community health workers (CHWs) with smartphones equipped with a tailored version of Dimagi's CommCare software to use during their home visits. The application supports CHWs who are using the Timed and Targeted Counselling (ttC) approach to promote positive health and nutrition behaviour change among pregnant women and mothers or caregivers of children under 2. The application provides reminders to help CHWs to visit homes at the ideal time during pregnancy, infancy and childhood. It also supports CHWs as they conduct counselling sessions, including enabling them to submit community health data in near real time. This data is then used for managing CHW efforts, planning and decision-making. To boost acceptability of this digital health tool, local language audio clips are incorporated into the application. support acceptability of the solution by the CHWs and the target beneficiaries. This CommCare-supported digital health intervention in Tanzania is expected to improve the effectiveness of behaviour change communication delivered to women and caregivers of children under 2 as well as strengthen the health system through better use of community-level data.	Service delivery, Health information system

NIA Juliana Busasi, juliannabusasi@gmail.com	United Republic of Tanzania	16/11/2021		Healthcare finance

Smart Growth Diary Mwombeki Fabian, info@fabstech.com	United Republic of Tanzania	01/01/2020	Diary is a mobile solution that gives autonomy to caregivers and mothers. It follows their child's growth in real time; get instant dietary information and what the child would be required to eat in terms of diversity, frequency, and amount of food; remind important milestones in immunization, and clinics visits.	Service delivery
AfyaIntelligence Harrison Mariki, afyaintelligence@gmail.com	United Republic of Tanzania	01/03/2021	AfyaIntelligence is an intelligent chatbot integrated in common messaging platforms(WhatsApp, Telegram and Text) to support healthcare supply chains. By doing so offers centralized access key of insights and foresights through digital communication platforms that healthcare workers are already familiar with. AfyaIntelligence makes HMIS and LMIS more resilient to pandemics by extending access to data, tracking and analyzing data-use through Chatbot Analytics and Creating a pathway to layer in new interventions like ML Predictions.	Service delivery, Access to medicines, vaccines and technology

Hyperlocal Geospatial Data for Improving Vaccination Campaigns Asha Mustapher, omdtanzania@gmail.com	United Republic of Tanzania	01/02/2022	The target beneficiaries in Dar es Salaam, Tanzania, are vulnerable individuals in local communities such as the elderly and those with underlying medical problems. Currently, Tanzania ranks almost at the bottom worldwide in terms of the percentage of the population to receive a dose of a Covid-19 vaccine. As of November 2021, we stand at 1.74% of the population vaccinated, according to data from the University of Oxford. Tanzania received around one million vaccines through Covid-19 Vaccines Global Access (COVAX), and another 500,000 Sinopharm vaccine doses as a donation from China—but this is for a population of over 60 million people.<br><br>OpenMap Development Tanzania (OMDTZ) has a unique potential to be impactful in improving the efficiency and reach of the national vaccination campaign. This is because for the last five years we have built a strong team of geospatial experts and community mappers, who have conducted projects across Tanzania, and in particular in Dar es Salaam.<br><br>We have worked with over 6500 community members, mapped over 11 million buildings, 10,000 kilometers of roads, and a host of other critical data within Dar es Salaam and Tanzania including the drainage system, community assets, and boundaries to name a few.	Service delivery, Health information system

mSpray` Anne Martin, acmartin@akros.com	Zambia	01/07/2014	mSpray is a field support and management tool for household-level interventions, used in indoor residual spraying. Spray operator teams use the geospatial, tablet based tool, to guide their teams in finding and capturing data on houses as they are sprayed. Data is available daily on dashboards for managers to review progress and adjust team planning as necessary; this data informed approach has been shown to reduce malaria cases and is in pilot phases for other malaria interventions.	Service delivery
eLMIS Facility Edition (FE) Wendy Bomett, wendy_bomett@zm.jsi.com	Zambia	01/06/2016	eLMIS FE has recently been upgraded from a Java Swing based client server application to a web-based two tier application with a PostgreSQL database back-end. At each health facility implementation, it works on their local area network (LAN) to enable multiple user access to the same application. It supports off-line access to it from within the LAN, without having to use internet, since it is a local installation within the facility. It can produce a monthly report and requisition (R&R).	Access to medicines, vaccines and technology, Service delivery
MNCH mobile content with nutrition focus, available in Bemba, Nyanja and English in SMS and voice transcript format Kim Viljoen, kviljoen@gsma.com	Zambia	01/09/2013	The GSMA, together with its global content consortium lead by CABI, developed open-source nutrition and MNCH mobile ready content for Zambia available in Bemba, Nyanja and English. All content was produced by in-country specialists and is based on recommended MNCH and local nutrition practices. The National Food and Nutrition Commission (NFNC) played a vital role in the development and content validation. The Content is available in the form of: topic-specific factsheets, SMSs and voice message transcripts.	Service delivery

ZEIR - Zambia Electronic Immunization Registry Laurie Werner, lwerner@path.org	Zambia	31/12/2017	As part of the BMFG Better Immunization Data (BID) Initiative. The MoH and PATH worked to implement OpenSRP for the Zambian Electronic Immunization Platform in Southern Province. This provides an easy to use app for nurses at facilities to register children, capture their immunization records and track growth monitoring. The application provides in app report, tally generation with the ability to feed aggregate reports to DHIS2. The platform was developed and supported by Ona and local in-country partner Blue Code who has taken over support for the platform for the MoH.	Health information system, Service delivery
Zambia health Analytics Platform (ZHAP) Kaluba Mataka, kaluba@zenysis.com	Zambia	14/01/2019	Zenysis has been working with the Ministry of Health (MOH), the National Malaria Elimination Centre (NMEC) and Medical Stores Limited (MSL) to implement the Zambia health Analytics Platform (ZHAP). The project is centered around three main pillars: Monitoring closely the evolution and trends of malaria burden; making better logistics decision to reduce risks of over-stock, waste and stockout; and, monitoring the impact and effectiveness of malaria intervention packages.	Service delivery, Health information system
OpenLMIS Zambia Rebecca Alban, Info@openlmis.org	Zambia	01/01/2014	OpenLMIS Zambia manages over 2,000 Service delivery points for ARV, HIV Testing, Essential Medicines and Labs with an additional stand alone offline facility edition in 319 high-volume Service delivery points.	Service delivery

DelphiCare: Clinical decision support for comprehensive, longitudinal care of HIV-exposed and infected children and adolescents Douglas Watson, MD, douglaswatsonmd@gmail.com	Zambia	03/02/2020	The University of Maryland, Baltimore; D-tree International; and Logiak, collaborating with the CDC and Zambian Ministry of Health, developed DelphiCare, an Android app enabling HCWs to provide longitudinal, expert-level management of HIV-exposed infants and -infected children. DelphiCare monitors the mother; screens for illness; recommends tests and interprets results; screens for TB and guides TB treatment, recommends drugs and dosages; guides counseling on adherence, disclosure, and nutrition; and recommends follow-up. The app is a unique advance in several respects: (a) the level of complexity and long-term dimension of the clinical condition; (b) its comprehensiveness, constituting both a clinical decision support system and an electronic client record; (c) the use of the Logiak platform, which allows for transparent logic to be written and validated by clinicians; and (d) changes in content and logic can be implemented across a healthcare system in near-real time with central access to all data.	Service delivery
Zambia National Data Warehouse Andrew Kashoka, andrew.kashoka@moh.gov.zm	Zambia	31/10/2018	The Data Warehouse is for Policy and Program Decision making in the Zambia Health Sector. It is the source of data interoperability from multiple systems which include but are not limited to the integrated human resource information system (iHRIS), electronic logistics management information systems (eLMIS), Lab information systems and the DIstrict Health information system (DHIS).<br>It is the link between data and decision support by forming a robust Extraction Transformation Staging Quality assurance & Loading (ETSQL) logical framework that ensures loading and presentation of high quality data in the Data Warehouse (DW) and Business Intelligence (BI) platforms	Health information system

Zambia immunization supply chain Arun Ramanujapuram, arun@logistimo.com	Zambia	11/11/2015	Logistimo SCM is a fully mobile-phone based supply chain management system that help manage the vaccine supply chain network in India, starting from the National warehouses, all the way down to the last-mile primary healthcare centers. The platform enables inventory management (including batch and expiry management), order management, demand forecasting, inventory optimization, remote temperature monitoring, asset/equipment management and delivery management. The system is being used in all 116 National, Provincial and District level warehouses. 37 health centers in Lusaka are covered. On a pilot basis, temperature in 17 refrigerators are being monitored remotely using Berlinger. The entire supply chain can be easily monitored using rich, interactive dashboards on web and mobile applications, supported by big data analytics.	Access to medicines, vaccines and technology, Service delivery
DHIS2 for COVID-19 Surveillance: Zambia (in development) Rebecca Potter, covid@dhis2.org	Zambia	27/08/2020	The DHIS2 digital data package for COVID-19 is designed to accelerate case detection, situation reporting, active surveillance and response in countries. The COVID-19 digital data package includes standard metadata aligned with the WHO's technical guidance on COVID-19 surveillance and has been adapted to local country context and language in this implementation.	Health information system
IDeAS. Integrated Decisions and Analytics Support. Mandy Dube, MDube@brhc.com	Zambia	28/07/2020	Implementing smart technology in healthcare facilities to improve the coordination of patient care. A collaboration between the CDC, the Zambian Ministry of Health and a consortium led by BroadReach to develop and deploy integrated patient management systems, delivering better treatment services, including for people living with HIV and AIDS, boosting overall clinical and public health outcomes in Zambia.The cornerstone of the program is enabling facilities across the country with innovative digital capabilities at the heart of	Health information system, Service delivery

			patient care. The program supports the integration of data from multiple healthcare systems with the ultimate vision of creating a platform for high-quality and secure electronic medical records for every Zambian.	
SmartCare Electronic Health Record System Andrew Kashoka, andrew.kashoka@moh.gov.zm	Zambia	01/09/2020	The SmartCare system ensures each person in Zambia has a complete electronic health record that is used to ensure the continuity of high-quality, confidential care by providing timely information to caregivers at the point of service	Service delivery, Health information system
Zambia Anternatal Care (ANC) Digital Adapation Project I Implementation Research Caren Chizuni, carenchizuni@gmail.com	Zambia	16/09/2020	This is an implementation research study which will be conducted in Eastern Province of Zambia.The WHO Antenatal care(ANC) digital module is a digital tool powered by OpenSRP. It will enable digital tracking and a decision support system to accelerate the adoption of the new ANC policies in Zambia.This study has two phases: a formative phase, which aims to prepare for the second, demonstration phase, by refining tools to the local context. The objective of the formative research phase is to adapt the WHO digital ANC module to the local ANC package and health delivery context. This phase will gather system requirements for local adaption. This will be facilitated by the ANC Digital Accelerator kit, a systems requirements tool that reflects the module.The demonstration phase will use implementation science to systematically introduce and test the applicability of the adapted WHO ANC package for a positive pregnancy experience and a new digital tool: Zambia digital ANC module at selected Service delivery levels.	Service delivery, Health information system

eNgoma Public Wi-Fi Chilufya Musosha, chilufya@e-ngoma.com	Zambia	24/07/2019	The eNgoma Captive Portal Wi-Fi Solution is a communication and engagement platform aimed at communicating with audiences through automated, engaging campaigns (animated digital Ads, infographics, short video clips and surveys - FAQs and Q&As, etc.) that provide a cost-effective proximity engagement solution to augment other traditional communication initiatives being utilized to deliver content to audiences. The eNgoma Captive Portal Wi-Fi Solution reaches audiences on their most viewed devices (their mobile devices) in an innovative, direct, impactful, and cost-effective manner. This has been used in collaboration with partners in the health sector to promote COVID awareness, PrEP, family planning, etc. It gives partners a real-time platform to track IEC materials that would ordinarily be in the form of paper, by converting these materials into byte size digital content, users of the system are provided access to life-changing health information while also having access to free internet access for a limited time. The system promotes a green content sharing platform for IEC materials while tracking views, demographics, and locations of viewers all in real time.	Service delivery
Implementation of a Performance-Based Financing Data System for Zimbabwe - using the open source tool Hesabu Benson Muzenda, Benson.Muzenda@cordaid.org	Zimbabwe	29/04/2020	Data System to Manage the performance based financing program including offline mobile data collection of quality of care and client satisfaction, the computation of program indicators, the calculation of incentive bonuses, the management of the PBF primary and subcontractor relationships and distribution of incentives.	Service delivery, Health informaiton system, Healthcare finance

Interactive SMS for VMMC Post-surgical follow up Caryl Feldacker, cfeld@uw.edu	Zimbabwe	16/04/2018	This project is a RCT study Medic Mobile is conducting in partnership with I-TECH, The University of Washington and ZAZIC, a local organization in Zimbabwe. Around 1-5% of VMMC clients have an adverse event like an infection that needs to be addressed, so currently everyone does multiple in-person follow up visits to be safe. The aim of this study is to test whether these follow up check ins can be performed via SMS. We are also doing a cost and scale/sustainability analysis of the intervention.	Service delivery
Impilo EHR Dr. Robert Gongora, rgongora536@gmail.com	Zimbabwe	01/02/2016	The MoHCC introduced a comprehensive, patient-centric Electronic Health Record system (EHR) in 2016, called "Impilo" (meaning "Health" in the local language of Ndebele) and designed and developed by a 100% Zimbabwean technical team, to support health workers to follow clinical protocols for priority health services such as malaria and HIV testing, maternal health services, and other outpatient care services and ensure a high standard of care, while capturing data along the way that was destined for numerous different health programmes.	Service delivery, Health information system
COVID-19 Hub for Zimbabwe Kumbirai Matingo, matingonk@gmail.com	Zimbabwe	21/03/2020	The COVID-19 Hub for Zimbabwe house's location based, informative, raw data and analytical resources that help the citizens, decision makers and persons in the technical fields to fight against this deadly virus which has claimed more lives over the past year. Citizens can also easily find resources using location intelligent applications contained within the hub (i.e., Vaccine Finders and COVID-19 Testing Centers). The solutions include dashboards, raw-datasets, and visual informative tools for the republic of Zimbabwe. Moreover, through the ArcGIS API we have managed to share external integration which leverage our data and resources into other innovative solutions by the	Service delivery

			Youth in Zimbabwe to promote and use ICT in the fight against COVID-19.	
Telehealth	Benin	Country e-Health Profile,2015	Teleradiology and Telepathology	Service delivery
Free emergency numbers	Benin	Country e-Health Profile,2015	Toll-free emergency numbers	Service delivery
Health call centres	Benin	Country e-Health Profile,2015	Health call centres	Service delivery
Mobile Health	Benin	Country e-Health Profile,2015	mobile health	Service delivery
Community Mobilization	Benin	Country e-Health Profile,2015	Community Mobilization	Service delivery
Access to information databases and tools	Benin	Country e-Health Profile,2015	Access to information databases and tools	Service delivery, Human resources for health
Decision support system	Benin	Country e-Health Profile,2015	Decision support system	Service delivery
Health Surveys	Benin	Country e-Health Profile,2015	Health survey	Healh information system
Supply chain information system	Benin	Country e-Health Profile,2015	Supply chain system	Service delivery, Access to medicines, vacccines and technology
Telehealth	Botswana	Country e-Health Profile,2015	Teleradiology,Teledermatology and Telepsychiatry	Service delivery
Telehealth	Botswana	Country e-Health Profile,2015	Telepathology and remote patient monitoring	Service delivery
Laboratory information system	Botswana	Country e-Health Profile,2015	Laboratory system	Service delivery

Phamarcy information system	Botswana	Country e-Health Profile,2015	Pharmarcy system	Service delivery
Electronic medical billing system	Botswana	Country e-Health Profile,2015	Billing system	Service delivery, Healthcare finance
Patient records	Burkina Faso	Country e-Health Profile,2015	Electronic medical record	Service delivery, Health information system
mLearning	Burkina Faso	Country e-Health Profile,2015	Learning and training system	Service delivery, Human resources for health
Decision support system	Burkina Faso	Country e-Health Profile,2015	Job aids	Service delivery
Diseaser Surveillance	Burkina Faso	Country e-Health Profile,2015	Public health surveilance	Health information system
Free emergency numbers	Burundi	Country e-Health Profile,2015	Toll-free emergency numbers	Service delivery
Health call centres	Burundi	Country e-Health Profile,2015	Health call centres	Service delivery
Appointment reminders	Burundi	Country e-Health Profile,2015	Appointment reminders	Service delivery
Mobile telehealth	Burundi	Country e-Health Profile,2015	Mobile telehealth	Service delivery
Disaster management	Burundi	Country e-Health Profile,2015	Disaster management	Service delivery
Treatment adherence	Burundi	Country e-Health Profile,2015	Treatment adherence	Service delivery
Community mobilization	Burundi	Country e-Health Profile,2015	Community mobilization	Service delivery

Access to information databases and tools	Burundi	Country e-Health Profile,2015	Access to information databases and tools	Service delivery, Human resources for health
Patient monitoring	Burundi	Country e-Health Profile,2015	Patient monitoring	Service delivery
Health Surveys	Burundi	Country e-Health Profile,2015	Health Surveys	Health information system
Diseaser Surveillance	Burundi	Country e-Health Profile,2015	Diseaser Surveillance	Health information system
Laboratory information system/PACS	Cabo Verde	Country e-Health Profile,2015	Laboratory information system/PACS	Service delivery
Pharmarcy information system	Cabo Verde	Country e-Health Profile,2015	Pharmarcy information system	Service delivery
Automatic vaccine alert system	Cabo Verde	Country e-Health Profile,2015	Automatic vaccine alert system	Servie delivery
Supply chain information system	Cabo Verde	Country e-Health Profile,2015	Supply chain information system	Service delivery, Access to medicines, vacccines and technology
e-Learning platform	Cabo Verde	Country e-Health Profile,2015	e-Learning platform	Service delivery, Human resources for health
Free emergency numbers	Cabo Verde	Country e-Health Profile,2015	Toll-free emergency numbers	Service delivery
Health call centres	Cabo Verde	Country e-Health Profile,2015	Health call centres	Service delivery
Appointment reminders	Cabo Verde	Country e-Health Profile,2015	Appointment reminders	Service delivery
mHealth	Cabo Verde	Country e-Health Profile,2015	mHealth	Service delivery

Community Mobilization	Cabo Verde	Country e-Health Profile,2015	Community Mobilization	Service delivery
Access to information databases and tools	Cabo Verde	Country e-Health Profile,2015	Access to information databases and tools	Service delivery, Human resources for health
Patient records	Cabo Verde	Country e-Health Profile,2015	Patient records	Service delivery, Health information system
m-Learning	Cabo Verde	Country e-Health Profile,2015	m-Learning	Service delivery, Human resources for health
Decision support system	Cabo Verde	Country e-Health Profile,2015	Decision support system	Service delivery
Health Surveys	Cabo Verde	Country e-Health Profile,2015	Health Surveys	Health information system
Disease Surveillace	Cabo Verde	Country e-Health Profile,2015	Disease Surveillace	Health information system
Disaster management	Central African Republic	Country e-Health Profile,2015	Disaster management	Service delivery
Telehealth	Côte d'Ivoire	Country e-Health Profile,2015	Teleradiology	Service delivery
Electronic billing system	Côte d'Ivoire	Country e-Health Profile,2015	Electronic billing system	Service delivery, Healthcare finance
Supply chain information system	Côte d'Ivoire	Country e-Health Profile,2015	Supply chain information system	Service delivery, Access to medicines, vacccines and technology
Toll free emergency numbers	Côte d'Ivoire	Country e-Health Profile,2015	Toll free emergency numbers	Service delivery
Health call centres	Côte d'Ivoire	Country e-Health Profile,2015	Health call centres	Service delivery

Appointment reminders	Côte d'Ivoire	Country e-Health Profile,2015	Appointment reminders	Service delivery
Disaster management	Côte d'Ivoire	Country e-Health Profile,2015	Disaster management	Service delivery
Treatment adherence	Côte d'Ivoire	Country e-Health Profile,2015	Treatment adherence	Service delivery
Access to information databases and tools	Côte d'Ivoire	Country e-Health Profile,2015	Access to information databases and tools	Service delivery, Human resources for health
mLearning	Côte d'Ivoire	Country e-Health Profile,2015	mLearning	Service delivery, Human resources for health
Patient monitoring	Côte d'Ivoire	Country e-Health Profile,2015	Patient monitoring	Service delivery
Disease Surveillance	Côte d'Ivoire	Country e-Health Profile,2015	Disease Surveillance	Health informaiton system
Telehealth	Ethiopia	Country e-Health Profile,2015	Teleradiology, teledermatology	Service delivery
Telehealth	Ethiopia	Country e-Health Profile,2015	Telepathology	Service delivery
Electronic Health record	Ethiopia	Country e-Health Profile,2015	Electronic Health record	Service delivey, Health information system
Laboratory information system	Ethiopia	Country e-Health Profile,2015	Laboratory information system	Service delivery
Pharmarcy information system	Ethiopia	Country e-Health Profile,2015	Pharmarcy information system	Service delivery
Electronic medical billing system	Ethiopia	Country e-Health Profile,2015	Electronic medical billing system	Service delivery, Healthcare finance

Supply chain information system	Ethiopia	Country e-Health Profile,2015	Supply chain information system	Service delivery, Access to medicines, vacccines and technology
e-Learning platform	Ethiopia	Country e-Health Profile,2015	e-Learning platform	Service delivery, Human resources for health
Toll free emergency numbers	Ethiopia	Country e-Health Profile,2015	Toll free emergency numbers	Service delivery
Health call centres	Ethiopia	Country e-Health Profile,2015	Health call centres	Service delivery
Appointment reminders	Ethiopia	Country e-Health Profile,2015	Appointment reminders	Service delivery
Mobile telehealth	Ethiopia	Country e-Health Profile,2015	Mobile telehealth	Service delivery
Disaster management	Ethiopia	Country e-Health Profile,2015	Disaster management	Service delivery
Treatment adherence	Ethiopia	Country e-Health Profile,2015	Treatment adherence	Service delivery
Access to information databases and tools	Ethiopia	Country e-Health Profile,2015	Access to information databases and tools	Service delivery, Human resources for health
m-Learning	Ethiopia	Country e-Health Profile,2015	m-Learning	Service delivery, Human resources for health
Health Surveys	Ethiopia	Country e-Health Profile,2015	Health Surveys	Health information system
Disease surveillance	Ethiopia	Country e-Health Profile,2015	Disease surveillance	Health information system
Telehealth	Gambia	Country e-Health Profile,2015	Teleradiology, teledermatology	Service delivery

Telehealth	Gambia	Country e-Health Profile,2015	Telepathology	Service delivery
Supply chain information system	Gambia	Country e-Health Profile,2015	Supply chain information system	Service delivery, Access to medicines, vacccines and technology
e-Learning platform	Gambia	Country e-Health Profile,2015	e-Learning platform	Service delivery, Human resources for health
Toll free emergency	Gambia	Country e-Health Profile,2015	Toll free emergency	Service delivery
Appointment reminders	Gambia	Country e-Health Profile,2015	Appointment reminders	Service delivery
Disaster management	Gambia	Country e-Health Profile,2015	Disaster management	Service delivery
Community mobilization	Gambia	Country e-Health Profile,2015	Community mobilization	Service delivery
Health survey	Gambia	Country e-Health Profile,2015	Health survey	Health information system
Electronic billing system	Ghana	Country e-Health Profile,2015	Electronic billing system	Service delivery,Healthcare finance
Supply chain information system	Ghana	Country e-Health Profile,2015	Supply chain information system	Service delivery, Access to medicines, vacccines and technology
e-Learning platform	Ghana	Country e-Health Profile,2015	e-Learning platform	Service delivery, Human resources for health
Toll free Emergency	Ghana	Country e-Health Profile,2015	Toll free Emergency numbers	Service delivery
Call centres and numbers	Ghana	Country e-Health Profile,2015	Health Call centres	Service delivery

Disaster management	Ghana	Country e-Health Profile,2015	Disaster management	Service delivery
Community mobilization	Ghana	Country e-Health Profile,2015	Community mobilization	Service delivery
Access to information databases and tools	Ghana	Country e-Health Profile,2015	Access to information databases and tools	Service delivery, Human resources for health
Patient records	Ghana	Country e-Health Profile,2015	Patient records	Service delivery, Health information system
m-Learning	Ghana	Country e-Health Profile,2015	m-Learning	Service delivery, Human resources for health
Patient monitoring	Ghana	Country e-Health Profile,2015	Patient monitoring	Service delivery
Health Survey	Ghana	Country e-Health Profile,2015	Health Survey	Health information system
Disease Surveillance	Ghana	Country e-Health Profile,2015	Disease Surveillance	Health information system
Telehealth	Kenya	Country e-Health Profile,2015	Teleradiology	Service delivery
Supply chain information system	Kenya	Country e-Health Profile,2015	Supply chain management information system	Service delivery, Access to medicines, vacccines and technology
Laboratory information system	Lesotho	Country e-Health Profile,2015	Laboratory information system	Service delivery
Pharmarcy information system	Lesotho	Country e-Health Profile,2015	Pharmarcy information system	Service delivery
Electronic billing system	Lesotho	Country e-Health Profile,2015	Electronic billing system	Service delivery, Healthcare finance

Supply chain information system	Lesotho	Country e-Health Profile,2015	Supply chain information system	Service delivery,Access to medicines, vacines and technology
Telehealth	Madagascar	Country e-Health Profile,2015	Teleradiology, Teledermatology	Service delivery
e-Learning platform	Madagascar	Country e-Health Profile,2015	e-Learning platform	Service delivery, Human resources for health
Appointment reminders	Madagascar	Country e-Health Profile,2015	Appointment reminders	Service delivery
Mobile telehealth	Madagascar	Country e-Health Profile,2015	Mobile telehealth	Service delivery
Disaster management	Madagascar	Country e-Health Profile,2015	Disaster management	Service delivery
Treatment adherence	Madagascar	Country e-Health Profile,2015	Treatment adherence	Service delivery
Community mobilization	Madagascar	Country e-Health Profile,2015	Community mobilization	Service delviery
Patient records	Madagascar	Country e-Health Profile,2015	Patient records	Service delivery, Health information system
m-Learning	Madagascar	Country e-Health Profile,2015	m-Learning	Service delviery, Human resources for health
Decision support system	Madagascar	Country e-Health Profile,2015	Decision support system	Service delivery, Human resources for health
Health survey	Madagascar	Country e-Health Profile,2015	Health survey	Health information system
Telehealth	Malawi	Country e-Health Profile,2015	Teleradiology	Service delivery

Electronic billing system	Malawi	Country e-Health Profile,2015	Electronic billing system	Service delivery, Healthcare finance
Supply chain information system	Malawi	Country e-Health Profile,2015	Supply chain information system	Service delivery, Access to medicines, vaccines and technology
e-Learning platform	Malawi	Country e-Health Profile,2015	e-Learning platform	Service delivery, Human resources and health
Toll free emergency numbers	Malawi	Country e-Health Profile,2015	Toll free emergency numbers	Service delivery
Health call centres	Malawi	Country e-Health Profile,2015	Health call centres	Service delivery
Appointment reminders	Malawi	Country e-Health Profile,2015	Appointment reminders	Service delivery
Mobile telehealth	Malawi	Country e-Health Profile,2015	Mobile telehealth	Service delivery
Disaster management	Malawi	Country e-Health Profile,2015	Disaster management	Service delivery
Community mobilization	Malawi	Country e-Health Profile,2015	Community mobilization	Service delivery
Access to information databases and tools	Malawi	Country e-Health Profile,2015	Access to information databases and tools	Service delivery, Human resources and health
Patient records	Malawi	Country e-Health Profile,2015	Patient records	Service delivery, Health information system
m-Learning	Malawi	Country e-Health Profile,2015	m-Learning	Service delivery, Human resources for health
Decision support system	Malawi	Country e-Health Profile,2015	Decision support system	Service delivery

Health survey	Malawi	Country e-Health Profile,2015	Health survey	Health information system
Disease Surveillance	Malawi	Country e-Health Profile,2015	Disease Surveillance	Health information system
Telehealth	Mali	Country e-Health Profile,2015	Teleradiology, Telepathology	Service delivery
Electronic billing system	Mali	Country e-Health Profile,2015	Electronic billing system	Service delivery, Healthcare finance
Supply chain information system	Mali	Country e-Health Profile,2015	Supply chain information system	Service delivery, Access to medicines, vaccines and technology
e-Learning platform	Mali	Country e-Health Profile,2015	e-Learning platform	Service delivery, Human resources for health
Toll free emergency	Mali	Country e-Health Profile,2015	Toll free emergency numbers	Service delivery
Call centres and numbers	Mali	Country e-Health Profile,2015	Health Call centres	Service delivery
Appointment reminders	Mali	Country e-Health Profile,2015	Appointment reminders	Service delivery
Mobile telehealth	Mali	Country e-Health Profile,2015	Mobile telehealth	Service delivery
Disaster management	Mali	Country e-Health Profile,2015	Disaster management	Service delivery
Treatment adherence	Mali	Country e-Health Profile,2015	Treatment adherence	Service delivery
Community mobilization	Mali	Country e-Health Profile,2015	Community mobilization	Service delivery

Access to information databases and tools	Mali	Country e-Health Profile,2015	Access to information databases and tools	Service delivery, Human resources for health
Patient records	Mali	Country e-Health Profile,2015	Patient records	Service delivery, Health information system
m-Learning	Mali	Country e-Health Profile,2015	m-Learning	Service delivery, Human resources for health
Decision support system	Mali	Country e-Health Profile,2015	Decision support system	Service delivery
Health survey	Mali	Country e-Health Profile,2015	Health survey	Health information system
Disease surveillance	Mali	Country e-Health Profile,2015	Disease surveillance	Health information system
Telehealth	Mauritania	Country e-Health Profile,2015	Teleradiology, Telepathology	Service delivery
Electronic billing system	Mauritania	Country e-Health Profile,2015	Electronic billing system	Service delivery, Healthcare finance
Supply chain information system	Mauritania	Country e-Health Profile,2015	Supply chain information system	Service delivery, Access to medicines, vaccines and technology
e-Learning platform	Mauritania	Country e-Health Profile,2015	e-Learning platform	Service delivery, Human resources for health
Toll free emergency	Mauritania	Country e-Health Profile,2015	Toll free emergency	Service delivery
Mobile telehealth	Mauritania	Country e-Health Profile,2015	Mobile telehealth	Service delivery
Disaster management	Mauritania	Country e-Health Profile,2015	Disaster management	Service delivery

Community mobilization	Mauritania	Country e-Health Profile,2015	Community mobilization	Service delivery
Access to information databases and tools	Mauritania	Country e-Health Profile,2015	Access to information databases and tools	Service delivery, Human resources for health
Patient monitoring	Mauritania	Country e-Health Profile,2015	Patient monitoring	Service delivery
Health survey	Mauritania	Country e-Health Profile,2015	Health survey	Health information system
Disease Surveillance	Mauritania	Country e-Health Profile,2015	Disease Surveillance	Health information system
Telehealth	Niger	Country e-Health Profile,2015	Teleradiology, teledermatology, telepathology,telepsychiatry, remote patient monitoring	Service delivery
Electronic billing system	Niger	Country e-Health Profile,2015	Electronic billing system	Service delivery, Healthcare finance
Supply chain information system	Niger	Country e-Health Profile,2015	Supply chain information system	Service delivery, Access to medicines, vaccines and technology
e-Learning platform	Niger	Country e-Health Profile,2015	e-Learning platform	Service delivery, Human resources for health
Toll-free emergency number	Niger	Country e-Health Profile,2015	Toll-free emergency number	Service delivery
Call centres and numbers	Niger	Country e-Health Profile,2015	Health call centres	Service delivery
Appointment reminders	Niger	Country e-Health Profile,2015	Appointment reminders	Service delivery
Mobile telehealth	Niger	Country e-Health Profile,2015	Mobile telehealth	Service delivery

Disaster management	Niger	Country e-Health Profile,2015	Disaster management	Service delivery
Treatment adherence	Niger	Country e-Health Profile,2015	Treatment adherence	Service delivery
Community mobilization	Niger	Country e-Health Profile,2015	Community mobilization	Service delivery
Access to information databases and tools	Niger	Country e-Health Profile,2015	Access to information databases and tools	Service delivery, Human resoures for health
m-Learning	Niger	Country e-Health Profile,2015	m-Learning	Service delivery, Human resources for health
Decision support system	Niger	Country e-Health Profile,2015	Decision support system	Service delivery
Patient monitoring	Niger	Country e-Health Profile,2015	Patient monitoring	Service delivery
Health survey	Niger	Country e-Health Profile,2015	Health survey	Health information system
Disease Surveillance	Niger	Country e-Health Profile,2015	Disease Surveillance	Health information system
Telehealth	Rwanda	Country e-Health Profile,2015	Teleradiology	Service delivery
Telehealth	Rwanda	Country e-Health Profile,2015	Remote patient monitoring	Service delivery
e-Learning platform	Rwanda	Country e-Health Profile,2015	e-Learning platform	Service delivery, Human resources for health
Toll-free emergency number	Rwanda	Country e-Health Profile,2015	Toll-free emergency number	Service delivery

Health call centres	Rwanda	Country e-Health Profile,2015	Health call centres	Service delivery
Appointment reminders	Rwanda	Country e-Health Profile,2015	Appointment reminders	Service delivery
Mobile telehealth	Rwanda	Country e-Health Profile,2015	Mobile telehealth	Service delivery
Disaster management	Rwanda	Country e-Health Profile,2015	Disaster management	Service delivery
Community Mobilization	Rwanda	Country e-Health Profile,2015	Community Mobilization	Service delivery
Access to information databases and tools	Rwanda	Country e-Health Profile,2015	Access to information databases and tools	Service delivery, Human resources for health
Patient records	Rwanda	Country e-Health Profile,2015	Patient records	Service delivery, Health information system
Decision support system	Rwanda	Country e-Health Profile,2015	Decision support system	Service delivery
Patient monitoring	Rwanda	Country e-Health Profile,2015	Patient monitoring	Service delivery
Disease Surveillance	Rwanda	Country e-Health Profile,2015	Disease Surveillance	Health information system
Telehealth	Senegal	Country e-Health Profile,2015	Teleradiology, Teledermatology, telepathology	Service delivery
Telehealth	Senegal	Country e-Health Profile,2015	Remote patient monitoring	Service delivery
Electronic billing system	Senegal	Country e-Health Profile,2015	Electronic billing system	Service delivery, Healthcare finance

Supply chain information system	Senegal	Country e-Health Profile,2015	Supply chain information system	Service delivery, Access to medicines, vaccines and technology
e-Learning platform	Senegal	Country e-Health Profile,2015	e-Learning platform	Service delivery, Human resources for health
Toll free emegency	Senegal	Country e-Health Profile,2015	Toll free emegency number	Service delivery
Call centres and numbers	Senegal	Country e-Health Profile,2015	Health Call centres	Service delivery
Appointment reminders	Senegal	Country e-Health Profile,2015	Appointment reminders	Service delivery
Mobile telehealth	Senegal	Country e-Health Profile,2015	Mobile telehealth	Service delivery
Disaster management	Senegal	Country e-Health Profile,2015	Disaster management	Service delivery
Treatment adherence	Senegal	Country e-Health Profile,2015	Treatment adherence	Service delivery
Community mobilzation	Senegal	Country e-Health Profile,2015	Community mobilzation	Service delivery
Access to information databases and tools	Senegal	Country e-Health Profile,2015	Access to information databases and tools	Service delivery, Human resources for health
Patient records	Senegal	Country e-Health Profile,2015	Patient records	Service delivery, Health information system
m-Learning	Senegal	Country e-Health Profile,2015	m-Learning	Service delivery, Human resoures for health
Decision support system	Senegal	Country e-Health Profile,2015	Decision support system	Service delivery

Patient monitoring	Senegal	Country e-Health Profile,2015	Patient monitoring	Service delivery
Health survey	Senegal	Country e-Health Profile,2015	Health survey	Health information system
Disease Surveillance	Senegal	Country e-Health Profile,2015	Disease Surveillance	Health information system
Health call centres	South Africa	Country e-Health Profile,2015	Health call centres	Service delivery
Appointment reminders	South Africa	Country e-Health Profile,2015	Appointment reminders	Service delivery
Mobile telehealth	South Africa	Country e-Health Profile,2015	Mobile telehealth	Service delivery
Treatment adherence	South Africa	Country e-Health Profile,2015	Treatment adherence	Service delivery
Community mobilization	South Africa	Country e-Health Profile,2015	Community mobilization	Service delivery
Access to information databases and tools	South Africa	Country e-Health Profile,2015	Access to information databases and tools	Service delivery, Human resources for health
Patient records	South Africa	Country e-Health Profile,2015	Patient records	Service delivery, Health information ssytem
Patient monitoring	South Africa	Country e-Health Profile,2015	Patient monitoring	Service delivery
Health survey	South Africa	Country e-Health Profile,2015	Health survey	Health information system
Disease Surveillance	South Africa	Country e-Health Profile,2015	Disease Surveillance	Health information system

Health call centres	South Sudan	Country e-Health Profile,2015	Health call centres	Service delivery
Community mobilization	South Sudan	Country e-Health Profile,2015	Community mobilization	Service delivery
Telehealth	Sudan	Country e-Health Profile,2015	Teleradiology	Service delivery
Electronic billing system	Sudan	Country e-Health Profile,2015	Electronic billing system	Service delivery, Healthcare finance
Supply chain information system	Sudan	Country e-Health Profile,2015	Supply chain information system	Service delivery, Access to medicines, vaccines and technology
e-Learning platform	Sudan	Country e-Health Profile,2015	e-Learning platform	Service delivery, Human resources for health
Toll-free emergency number	Sudan	Country e-Health Profile,2015	Toll-free emergency number	Service delivery
Health call centres	Sudan	Country e-Health Profile,2015	Health call centres	Service delivery
Mobile telehealth	Sudan	Country e-Health Profile,2015	Mobile telehealth	Service delivery
Disaster management	Sudan	Country e-Health Profile,2015	Disaster management	Service delivery
Community mobilization	Sudan	Country e-Health Profile,2015	Community mobilization	Service delivery
Decision support system	Sudan	Country e-Health Profile,2015	Decision support system	Service delivery
Health survey	Sudan	Country e-Health Profile,2015	Health survey	Health information system

Telehealth	Uganda	Country e-Health Profile,2015	Teleradiology, teledermatology, telepathology	Service delivery
Telehealth	Uganda	Country e-Health Profile,2015	Telepsychiatry, remote patient monitoring	Service delivery
Electronic billing system	Uganda	Country e-Health Profile,2015	Electronic billing system	Service delivery, Healthcare finance
Supply chain information system	Uganda	Country e-Health Profile,2015	Supply chain information system	Service delivery, Access to medicine, vaccines and technology
e-Learning platform	Uganda	Country e-Health Profile,2015	e-Learning platform	Service delivery, Human resources for health
Toll free emergency numbers	Uganda	Country e-Health Profile,2015	Toll free emergency numbers	Service delivery
Health call centres	Uganda	Country e-Health Profile,2015	Health call centres	Service delivery
Appointment reminders	Uganda	Country e-Health Profile,2015	Appointment reminders	Service delivery
Mobile telehealth	Uganda	Country e-Health Profile,2015	Mobile telehealth	Service delivery
Disaster management	Uganda	Country e-Health Profile,2015	Disaster management	Service delivery
Treatment adherence	Uganda	Country e-Health Profile,2015	Treatment adherence	Service delivery
Community mobilization	Uganda	Country e-Health Profile,2015	Community mobilization	Service delivery
Access to information databases and tools	Uganda	Country e-Health Profile,2015	Access to information databases and tools	Service delivery, Human resources for health

Patient records	Uganda	Country e-Health Profile,2015	Patient records	Service delivery, Health information system
mLearning	Uganda	Country e-Health Profile,2015	mLearning	Service delivery
Decision support system	Uganda	Country e-Health Profile,2015	Decision support system	Service delivery
Patient monitoring	Uganda	Country e-Health Profile,2015	Patient monitoring	Service delivery
Health survey	Uganda	Country e-Health Profile,2015	Health survey	Health information system
Disease Surveillance	Uganda	Country e-Health Profile,2015	Disease Surveillance	Health information system
Telehealth	Zambia	Country e-Health Profile,2015	Teleradiology	Service delivery
Supply chain information system	Zambia	Country e-Health Profile,2015	Supply chain information system	Service delivery
e-Learning platform	Zambia	Country e-Health Profile,2015	e-Learning platform	Service delivery, Human resources for health
Toll free emergency numbers	Zambia	Country e-Health Profile,2015	Toll free emergency numbers	Service delivery
Health call centres	Zambia	Country e-Health Profile,2015	Health call centres	Service delivery
Appointment reminders	Zambia	Country e-Health Profile,2015	Appointment reminders	Service delivery
Treatment adherence	Zambia	Country e-Health Profile,2015	Treatment adherence	Service delivery

Community mobilization	Zambia	Country e-Health Profile,2015	Community mobilization	Service delivery
Patient monitoring	Zambia	Country e-Health Profile,2015	Patient monitoring	Service delivery
m-Learning	Zambia	Country e-Health Profile,2015	m-Learning	Service delivery, Human resources for health
Decision support system	Zambia	Country e-Health Profile,2015	Decision support system	Service delivery
Health survey	Zambia	Country e-Health Profile,2015	Health survey	Health information system
Disease Surveillance	Zambia	Country e-Health Profile,2015	Disease Surveillance	Health information system
Telehealth	Zimbabwe	Country e-Health Profile,2015	Teleradiology	Service delivery
Telehealth	Zimbabwe	Country e-Health Profile,2015	Teledermatology	Service delivery
Telehealth	Zimbabwe	Country e-Health Profile,2015	Telepathology	Service delivery
Telehealth	Zimbabwe	Country e-Health Profile,2015	Remote patient monitoring	Service delivery
e-Learning platform	Zimbabwe	Country e-Health Profile,2015	e-Learning platform	Service delivery, Human resources for health
Toll free emergency	Zimbabwe	Country e-Health Profile,2015	Toll free emergency numbers	Service delivery
Health call centres	Zimbabwe	Country e-Health Profile,2015	Health call centres	Service delivery

Appointment reminders	Zimbabwe	Country e-Health Profile,2015	Appointment reminders	Service delivery
Mobile telehealth	Zimbabwe	Country e-Health Profile,2015	Mobile telehealth	Service delivery
Disaster management	Zimbabwe	Country e-Health Profile,2015	Disaster management	Service delivery
Treatment adherence	Zimbabwe	Country e-Health Profile,2015	Treatment adherence	Service delivery
Community Mobilization	Zimbabwe	Country e-Health Profile,2015	Community Mobilization	Service delivery
Access to information databases and tools	Zimbabwe	Country e-Health Profile,2015	Access to information databases and tools	Service delivery, Human resources for health
Patient records	Zimbabwe	Country e-Health Profile,2015	Patient records	Service delivery
mLearning	Zimbabwe	Country e-Health Profile,2015	mLearning	Service delivery, Human resources for health
Decision support system	Zimbabwe	Country e-Health Profile,2015	Decision support system	Service delivery
Patient monitoring	Zimbabwe	Country e-Health Profile,2015	Patient monitoring	Service delivery
Health survey	Zimbabwe	Country e-Health Profile,2015	Health survey	Health information system
Disease Surveillance	Zimbabwe	Country e-Health Profile,2015	Disease Surveillance	Health information system

m-health applications in Botswana: telemedicine and education, Littman-Quinn et al; 2013	Botswana	2013	The Botswana-University of Pennsylvania Partnership (BUP) has piloted four mobile telemedicine projects in the specialties of women's health (cervical cancer screening utilizing visual inspection with acetic acid), radiology, oral medicine and dermatology. BUP has initiated an m-learning programme with the University of Botswana School of Medicine	Service delivery, Human resources for health.
e-MANIC, Institut de Recherche en Sciences de la Sante, Burkina Faso,2020	Burkina Faso	2020	Artificial intelligence integrating multiple layers of clinical information such as clinical examination, signs/symptoms and medical history, and laboratory information such as outcomes of biomarkers (CRP and WBC) and pathogen specific POCT (malaria and bacterial infections) and oximetry	Service delivery
Smart glasses for telemedicine, Diaka et al;2021	Democratic Republic of Congo	2021	To improve primary healthcare services, especially referrals to the district hospital, for the population in three health centres in the rural district Kingandu in the Democratic Republic of the Congo (DRC) by introducing Smart Glasses, and leveraging them for telemedicine.	Service delivery
Cloud-based peri-opertive registry,Network of perioperative critical care, 2020	Ethiopia	2020	Context specific, clinician-led, multicentre real-time peri-operative registry in Ethiopia	Service delivery
mHealth Intervention on Delivery and Postnatal Care Utilization, Spigt et al; 2016	Ethiopia	2016	The Effects of a Locally Developed mHealth Intervention on Delivery and Postnatal Care Utilization; A Prospective Controlled Evaluation among Health Centres in Ethiopia	Service delivery
USAID Digital Health Activity (DHA), https://www.jsi.com/project/digital-health-activity/	Ethiopia	2020	The USAID-funded Digital Health Activity (DHA) is a five-year project that supports Ethiopia in improving the quality and availability of healthcare services through the deployment of data-driven and patient-centered digital tools.	Service delivery, Health information system

Cloud-based electronic data system, Jede et al;2020	Ethiopia	2020	A cervical cancer screening campaign was conducted in a rural community in Ethiopia. All women aged 25-65 years were offered genital self-sampling using the Evalyn Brush®. Samples were HPV-DNA-tested at a central laboratory. Key indicators were captured on tablet computers and linked by a cloud-based information system.	Service delivery, Health information system
'virtual ward' (VW) system, Wariri et al; 2021	Gambia	2021	Establishing and operating a 'virtual ward' system to provide care for patients with COVID-19 at home: experience from The Gambia	Service delivery
Empower Health software, Otieno et al;2020	Ghana	2020	On the mobile application, clinicians viewed patient data, provided direct patient feedback, and wrote electronic prescriptions accessible through participating pharmacies	Service delivery
COVID-19 Tracker App, https://www.itu.int/hub/2020/05/ghana-launches-covid-19-tracker-app/	Ghana	2020	The COVID-19 Tracker App is able to trace contacts of persons infected by the virus and show where they have been in recent times through various telephone-related data, and link such people to health professionals for urgent action to be taken.	Service delivery, Health informatin system
mHealth to Train Community Health Nurses, Asgary et al; 2016	Ghana	2016	We evaluated the feasibility and limited efficacy of a smartphone-based training of community health nurses in visual inspection of the cervix under acetic acid (VIA)	Service delivery, Human resources for healths
Telecytology in East Africa, Kumar et al;2012	Kenya	2012	A pilot study to assess the feasibility of telecytology as a diagnostic tool in difficult cases originating from a hospital in East Africa	Service delivery
Real-Time Monitoring and Evaluation of a Visual-Based Cervical Cancer Screening Program Using a Decision Support Job Aid, Peterson et al 2016	Kenya	2016	We tested the implementation of the novel job aid feature on the mobile colposcope app during an EVA deployment in a cervical cancer screening camp in Nairobi, Kenya. We show that the integration of this feature allows for improved M&E of VIA programs by providing key statistics in real time.	Service delivery, Health information system
The Millennium Villages Project (MVP), mobile Health tool, Mushamiri et al; 2015	Kenya	2015	Evaluation of the impact of a mobile health system on adherence to antenatal and postnatal care and prevention of mother-to-child transmission of HIV programs in Kenya	Service delivery

Mobile research, Leidich et al; 2018	Kenya	2018	Evaluating the feasibility and acceptability of sending pregnancy and abortion history surveys through SMS text messaging to help reach sustainable development goal 3	Health information system
Afya data, Karumuribo et al; 2017	Kenya	2017	A mobile based disease sureillance system	Service delivery, Health information system
Connected Diagnostics, van Duijn; 2021	Kenya	2021	Digitalized rapid diagnostic tests (RDTs) through photography and interprets and results stored in cloud-based databases through dedicated RDT readers.	Service delivery
The Health and Demographic Surveillance System (HDSS), Kaneko et al 2012	Kenya	2012	The Health and Demographic Surveillance System (HDSS) is a longitudinal data collection process that systematically and continuously monitors population dynamics for a specified population in a geographically defined area that lacks an effective system for registering demographic information and vital events. It utilizes personal digital assistants and global positioning system devices	Health information system
Empower Health software, Otieno et al;2020	Kenya	2020	On the mobile application, clinicians viewed patient data, provided direct patient feedback, and wrote electronic prescriptions accessible through participating pharmacies	Service delivery
i-PUSH, Abajobir et al; 2021	Kenya	2021	The Innovative Partnership for Universal Sustainable Healthcare (*i*-PUSH) program, developed by AMREF Health Africa and PharmAccess Foundation (PAF), aims to empower low-income women of reproductive age and their families through innovative digital tools. This study aims to evaluate the impact of *i*-PUSH on maternal and child health care utilization, women's health including their knowledge, behavior, and uptake of respective services, as well as women's empowerment and financial protection. It also aims to evaluate the impact of the LEAP training tool on empowering and enhancing community health volunteers' health literacy and to evaluate the impact of the M-TIBA health wallet on savings for health and health insurance uptake.	Service delivery, Healthcare finance

Inuka Coaching, Doukani et al 2021	Kenya	2021	A community health volunteer delivered problem-solving therapy mobile application based on the Friendship Bench 'Inuka Coaching' in Kenya: A pilot cohort study	Service delivery
PANDA (pregnancy and newborn diagnosis assessment) system, Garcia Vilaplana et al;2020	Madagascar	2020	An mHealth device that incorporates the WHO recommendations for antenatal care (ANC). Data, including personal and medical information, but also clinical data such as hypertension, anemia or HIV	Service delivery, Health information system
The Mobile Maternal Health Wallet (MMHW), Lacroze et al; 2021	Madagascar	2021	Determine the impact on health outcomes, cost-effectiveness, feasibility, acceptability, and usefulness of mobile phone-based savings and payment service, the Mobile Maternal Health Wallet (MMHW), for skilled healthcare during pregnancy and delivery among women in Madagascar	Service delivery, Healthcare finance
Smartphone Use for Cervical Cancer Screening in Low-Resource Countries, Catarino et al; 2015	Madagascar	2015	The aim of this study was to evaluate the use of a smartphone for on- and off-site D-VIA diagnosis.	Service delivery
A Smartphone-Based Approach for Triage of Human Papillomavirus, Urner et al; 2017	Madagascar	2017	The aim of the study was to evaluate the performances of digital smartphone-based visual inspection with acetic acid (D-VIA) and Lugol's iodine (D-VILI) for diagnosing cervical precancer and cancer.	Service delivery
Smartphone digital images for the detection of cervical intraepithelial neoplasia of grade 2 or worse (CIN2+), Ricard-Gauthier et al; 2015	Madagascar	2015	Our aim was to evaluate the feasibility and performance of smartphone digital images for the detection of cervical intraepithelial neoplasia of grade 2 or worse (CIN2+) as an adjunct to a conventional visual inspection approach with acetic acid (VIA) and Lugol's iodine (VILI), in comparison with detection by histopathologic examination.	Service delivery
A Mobile Health Data Collection System, Quercia et al; 2018	Madagascar	2018	The aim of this study was to assess the feasibility of a mobile health (m-Health) data collection system to facilitate monitoring of women participating to cervical cancer screening campaign.	Health information system

NeoTree digital capture and quality improvement system Mgusha et al 2021	Malawi	2021	Mobile Perinatal Admissions App to describe patterns of admissions and outcomes in babies admitted to a Malawian neonatal	Service delivery, Health information system
StAR2D, Farmer et al 2021	Malawi	2021	a 12-month two-arm randomised trial of SMS-text messaging and usual care in Cape Town, South Africa and Lilongwe, Malawi.	Service delivery
Computer-assisted digital chest X-ray technology, Twabi et al;2021	Malawi	2021	Computer-assisted digital chest X-ray technology	Service delivery
Mobile Phone Text Message Reminders, Steinhardt et al; 2018	Malawi	2018	The Effect of Mobile Phone Text Message Reminders on Health Workers' Adherence to Malaria and Other Disease Case Management Guidelines in Malawi	Service delivery
Accurate Real-Time Diagnosis of Lymphoproliferative Disorders in Malawi Through Clinicopathologic Teleconferences, Montgomery et al; 2016	Malawi	2016	We have established a laboratory in Malawi to support clinical and research efforts at a national teaching hospital. Consensus real-time diagnoses are rendered by local pathologists after weekly clinicopathologic teleconferences involving clinicians and pathologists from the United States and Malawi. Additional ancillary studies are then performed in the United States prior to final diagnosis.	Service delivery
Tablet-Based Cervical Cancer Educational Intervention, Caster et al; 2017	Malawi	2017	We developed and implemented an interactive, tablet-based educational intervention to improve cervical cancer knowledge among women in rural Malawi	Service delivery
Wellvis COVID triage tool, Owoyemi et al;2021	Nigeria	2021	We developed an eight-question triage tool using the Nigerian Centre for Disease Control surveillance case definitions for new coronavirus disease. Based on the assessed risk level we offered advice and guidance on the next steps. A user-administered tool such as this is vital to COVID19 control. It is also significant in relieving the burden on health systems, providing information on national health guidelines for prevention and control, fostering the participation of citizens, and giving them the next steps, pandemic control efforts become more effective.	Service delivery, Health information system

EXTEND evaluation, Ebenso et al 2018	Nigeria	2018	The eHealth interventions were implemented by 'InStrat' from March 2017 to March 2019, in collaboration with the State Ministries of Health in Ondo and Kano States and the DOH in the FCT. A successful pilot testing of VTR and CliniPAK apps in Ondo State in 2016 led to scaling up of eHealth interventions to Kano state and the FCT in 2017	Service delivery, Human resources for health
Remote Teaching of Histopathology, Rotimi et al; 2017	Nigeria	2017	To explore Web-based applications for pathology teaching in resource-limited sub-Saharan Africa	Service delivery, Human resources for health
Mobile phone short message service, Omole et al; 2018	Nigeria	2018	To determine the impact of SMS (text messages) on maternal health behaviour in Ife-Ijesa zone of Osun State, Nigeria.	Service delivery
Neural Transfer Learning for Cry-based Diagnosis of Perinatal Asphyxia, Onu et al;2019	Nigeria	2019	This study explores a neural transfer learning approach to developing accurate and robust models for identifying infants that have suffered from perinatal asphyxia	Service delivery
PEERNaija, Ahonkhai et al; 2021	Nigeria	2021	PEERNaija. Grounded in Social Cognitive Theory, and principles of contingency management and supportive accountability, PEERNaija delivers a multi-faceted behavioral intervention within a smartphone application to address important obstacles to adherence	Service delivery
Mobile digital quiz game for the promotion of malaria awareness,Chikwuocha et al; 2020	Nigeria	2020	The development and testing of a mobile quiz game to promote malaria awareness, perceptions and appropriate practice	Service delivery
Clinical decision support algorithm, Center for Primary Care and Public Health (Unisante), University of Lausanne, Switzerland, 2022	Rwanda	2022	Dynamic Clinical Decision Support Algorithms to Manage Sick Children in Primary Health Care Settings in Rwanda	Service delivery
RapidSMS-MCH, Ngabo et al; 2012	Rwanda	2012	Innovative SMS-based alert system (RapidSMS-MCH) to monitor pregnancy and reduce maternal and child deaths in Rwanda	Service delivery
Telepathology Triage, Mpunga et al; 2016	Rwanda	2016	In this study, we present the first phase of an anatomic telepathology triage system, which was implemented and validated at the Butaro District Hospital in northern rural Rwanda.	Service delivery

Smartphone Application for Detecting Skin Cancers, Rubagumya et al; 2020	Rwanda	2020	We assessed the feasibility of using a mobile application (app) for detection of skin cancers among people with albinism.	Service delivery
Uber for Blood, McVeigh, K. 2018. "'Uber for Blood': How rwandan Delivery robots Are Saving lives." The Guardian	Rwanda	2018	A Silicon Valley robotics company has teamed up with the Rwandan health ministry to hasten the delivery of vital medicines to hospitals in remote areas	Service delivery, Access to medicines, vaccines and technology
Aviro Health App, https://borgenproject.org/aviro-health-in-south-africa/	South Africa	2012	Aviro Health in South Africa began in 2012. It is a technology company based in Cape Town that develops digital innovations that assist both health care practitioners and patients	Service delivery
Tele-reading, Griggs et al ;2014	South Africa	2014	World Federation of Pediatric Imaging (WFPI) volunteer outreach through tele-reading: the pilot project in South Africa	Service delivery
TBDx, Applied Visual Systems, Inc, Swati et al;2016	South Africa	2016	Diagnostic algorithms using automated digital microscopy	Service delivery
Mobile Health (mHealth) technologies for epilepsy, Davies et al 2021	South Africa	2021	Technology-based home monitoring was investigated in an observational study of 40 children with refractory epilepsy or epilepsy associated with intellectual disability and/or behavior difficulties in South Africa	Service delivery
d-CXR, Moodley et al 2021	South Africa	2021	digital chest X-ray with computer-aided detection (CAD) software (d-CXR) for identifying undiagnosed TB in three primary health clinics in South Africa.	Service delivery
momConnect, Pillay and Motsoaledi,2018	South Africa	2018	MomConnect was established by the National Department of Health in 2014 to register pregnancies and provide pregnant and postpartum women with twice-weekly health information text messages as well as access to a help desk for queries and feedback	Service delivery
StAR2D, Farmer et al 2021	South Africa	2021	a 12-month two-arm randomised trial of SMS-text messaging and usual care in Cape Town, South Africa and Lilongwe, Malawi.	Service delivery
The Philani MOVIE, Adam et al 2021	South Africa	2021	We measured the effects of an animated, mobile video series, the Philani MObile Video Intervention for Exclusive breastfeeding (MOVIE), delivered by a cadre of CHWs ("mentor mothers").	Service delivery

Home-based intervention to test and start (HITS), Mathenjwa et al 2019	South Africa	2019	a tablet-based male-targeted HIV decision support application	Service delivery
ProLife Moriarty et al; 2019	South Africa	2019	The ProLife programme consists of an MI counselling strategy, delivered by lay health workers, augmented with subsequent SMS. Each MI session will be followed by twice-weekly SMS messages targeting treatment adherence, alcohol use and tobacco smoking, as appropriate	Service delivery
Mobile tracking of those with coronavirus, https://www.reuters.com/article/health-coronavirus-telkom-sa/s-africa-launches-mobile-tracking-of-those-with-coronavirus-idUSL8N2BQ61L	South Africa	2020	South Africa's Telkom , Samsung and the government have teamed up to develop a track and trace database to identify the whereabouts of people who may have contracted coronavirus.	Service delivery, Health information system
Electronic booking system, Booyse et al; 2020	South Africa	2020	To evaluate the ability of the booking system to appropriately prioritise and accommodate clinically appropriate patients for an urgent gastroscopy within 2 weeks at Worcester Provincial Hospital (WPH)	Service delivery, Health information system
The Kilimanjaro Method, Yeates et al; 2016	Tanzania	2016	We hypothesized that a smartphone camera and use of cervical image transfer for real-time mentorship by experts located distantly across a closed user group through a commercially available smartphone application would be both feasible and effective in enhancing VIA skills among CCS providers in Tanzania	Service delivery, Human resources for health
The CAD4TB software is a computer-aided detection system Breuninger et al;2014	Tanzania	2014	The CAD4TB software is a computer-aided detection system for Automatic interpretation of chest x-Rays in TB diagnosis	Service delivery
Mobile phone Communication, Lund et al; 2012	Tanzania	2012	Mobile phones as a health communication tool to improve skilled attendance at delivery in Zanzibar: a cluster-randomised controlled trial	Service delivery
InfoSpots, Holst et al; 2021	Tanzania	2021	Installing community information spots (InfoSpots) with access to the internet and a locally stored digital health education platform (the platform) in Migoli and Izazi, Tanzania. The objective of this case study was to explore the perspectives and experiences of InfoSpot users and non-users in these communities.	Service delivery

Clinical decision support algorithm, Center for Primary Care and Public Health (Unisante), University of Lausanne, Switzerland, 2022	Tanzania	2022	Dynamic Clinical Decision Support Algorithms to Manage Sick Children in Primary Health Care Settings in Tanzania	Service delivery
SMS text messaging app, Siedner et al; 2015	Uganda	2015	The trial evaluated the efficacy of a SMS text messaging app to notify PLWH of their laboratory results and request return to care for those with abnormal test results.	Service delivery
Low-cost ruggedized Android tablets to augment in-service training of community health worker Stiles et al;2021	Uganda	2021	A mobile health (mHealth) assisted Integrated Community Case Management (iCCM) refresher training programme for CHWs in Mukono, Uganda	Service delivery, Human resources for health
Mobile Phone Consultations, Ayiasi et al 2016	Uganda	2016	Effect of Village Health Team Home Visits and Mobile Phone Consultations on Maternal and Newborn Care Practices in Masindi and Kiryandongo, Uganda: A Community-Intervention Trial	Service delivery
mTrac UNICEF Uganda, 2011	Uganda	2011	mTrac is used by health facility workers to submit routine, weekly health surveillance data by SMS using their own basic mobile phones. mTrac indicators include notifiable diseases, stock levels for eight tracer medicines, and maternal and neonatal deaths. This data is automatically integrated into DHIS2 for further analysis.	Service delivery , Access to medicines, vaccines and technology
mHealth application to strengthening pharmaceutical systems, Namisango et al; 2016	Uganda	2016	n electronic application was implemented as part of palliative care services at two settings in Uganda; a rural hospital and an urban hospice. Measures of the completeness of data capture, time efficiency of activities and medicines stock and waste management were taken pre- and post-implementation to identify changes to practice arising from the introduction of the application.	Service delivery, Access to medicines, vaccines and technology
Novel Mobile Phone Application to Improve Palliative Home-Care, Harding et al; 2021	Uganda	2021	This study aimed to design a mobile phone application (app) to enable or improve communication between family caregivers, community caregivers, and palliative care teams; to evaluate its acceptability, processes,	Service delivery

			and mechanisms of action; and to propose refinements.	
Smartphone strategies for cervical cancer screening, Asgary et al;2020	Swaziland	2020	We implemented smartphone-based VIA that included standard VIA training, adapted refresher, and 6-month mHealth mentorship, sequentially, in the rural Shiselweni region of Eswatini. A remote expert reviewer provided diagnostic and management feedback on patients' cervical images, which were reviewed weekly by nurses	Service delivery, Human resources for health
Retinopathy AI screening, Bellemo et.al 2019	Zambia	2019	An artificial intelligence (AI) model using deep learning in a population-based diabetic retinopathy screening programme in Zambia	Service delivery
HealthTrax, Hong et al; 2015	Zambia	2015	A new tool to identify and navigate dirt roads for health outreach work in Southern Zambia through GPS tracking	Service delivery
Automatic software (CAD4TB 5) in chest X-ray (CXR), Melendez et al; 2017	Zambia	2017	Compare the performance of automatic software (CAD4TB 5) in chest X-ray (CXR) reading with that of field (general practitioners) and central (radiologists) readers.	Service delivery
Interactive digital tablet-based counselling, Thomas et al;2019	Zimbabwe	2019	This pilot research trial aims to provide an initial evaluation of the impact of an interactive digital tabletbased counselling session, correcting risk perception, and addressing ambiguity around availability, usability, and effectiveness of PrEP.	Service delivery
Novel Mobile Phone Application to Improve Palliative Home-Care, Harding et al; 2021	Zimbabwe	2021	This study aimed to design a mobile phone application (app) to enable or improve communication between family caregivers, community caregivers, and palliative care teams; to evaluate its acceptability, processes, and mechanisms of action; and to propose refinements.	Service delivery

Table S2: Supplementary material : Country Distribution of digital health interventions according to the health systems strengthening building blocks

Country	Service delivery	Health Information System	Human Resources for Health	Access to Medicines	Leadership and Governance	Health care finance	Total
Angola	4 (50)	1(12.5)	1(12.5)	2 (50)	0 (0.0)	0 (0.0)	8
Benin	11 (61.1)	3 (16.7)	2 (11.1)	2 (11.1)	0 (0.0)	0 (0.0)	18
Botswana	6 (66.7)	1 (11.1)	1 (11.1)	0 (0.0)	0 (0.0)	1 (11.1)	9
Burkina Faso	8 (57.1)	5 (35.7)	1 (7.1)	0 (0.0)	0 (0.0)	0 (0.0)	14
Burundi	10 (71.4)	3 (21.4)	1 (7.1)	0 (0.0)	0 (0.0)	0 (0.0)	14
Cabo Verde	14 (63.6)	4 (18.2)	3 (13.6)	1 (4.5)	0 (0.0)	0 (0.0)	22
Cameroon	4 (57.1)	1 (14.3)	0 (0.0)	0 (0.0)	1 (14.3)	1 (14.3)	7
Central African Republic	2 (100)	0 (0.0)	0 (0.0)	0 (0.0)	0 (0.0)	0 (0.0)	2
Chad	0 (0.0)	1 (100)	0 (0.0)	0 (0.0)	0 (0.0)	0 (0.0)	1
Congo	0 (0.0)	1 (100)	0 (0.0)	0 (0.0)	0 (0.0)	0 (0.0)	1
Congo, the Democratic Republic of the	5 (71.4)	1 (14.3)	0 (0.0)	1 (14.3)	0 (0.0)	0 (0.0)	7
Côte d'Ivoire	25 (69.4)	2 (5.6)	4 (11.1)	3 (8.3)	0 (0.0)	2 (5.6)	36
Djibouti	0 (0.0)	1 (100)	0 (0.0)	0 (0.0)	0 (0.0)	0 (0.0)	1
Ethiopia	68 (56.7)	28 (23.3)	10 (8.3)	8 (6.7)	0 (0.0)	6 (5.0)	120
Gabon	2 (100)	0 (0.0)	0 (0.0)	0 (0.0)	0 (0.0)	0 (0.0)	2
Gambia	9 (69.2)	2 (15.4)	1 (7.7)	1 (7.7)	0 (0.0)	0 (0.0)	13
Ghana	21 (60)	6 (17.1)	6 (17.1)	1 (2.9)	0 (0.0)	1 (2.9)	35
Guinea	2 (28.6)	4 (57.1)	0 (0.0)	1 (14.3)	0 (0.0)	0 (0.0)	7
Guinea-Bissau	0 (0.0)	1 (100)	0 (0.0)	0 (0.0)	0 (0.0)	0 (0.0)	1
Kenya	43 (64.2)	16 (23.9)	0 (0.0)	4 (6.0)	0 (0.0)	4 (6.0)	67
Lesotho	11 (52.4)	7 (33.3)	0 (0.0)	2 (9.5)	0 (0.0)	1 (4.8)	21
Liberia	1 (33.3)	2 (66.7)	0 (0.0)	0 (0.0)	0 (0.0)	0 (0.0)	3
Madagascar	17 (60.7)	6 (21.4)	3 (10.7)	0 (0.0)	0 (0.0)	2 (7.1)	28
Malawi	37 (59.7)	17 (27.4)	3 (4.8)	4 (6.5)	0 (0.0)	1 (1.6)	62
Mali	17 (63.0)	5 (18.5)	3 (11.1)	1 (3.7)	0 (0.0)	1 (3.7)	27

Mauritania	11 (61.1)	3 (16.7)	2 (11.1)	1 (5.6)	0 (0.0)	1 (5.6)	18
Mozambique	5 (33.3)	4 (26.7)	2 (13.3)	3 (20.0)	1 (6.7)	0 (0.0)	15
Namibia	2 (66.7)	1 (33.3)	0 (0.0)	0 (0.0)	0 (0.0)	0 (0.0)	3
Niger	16(64.0)	4 (16.0)	3 (12.0)	1 (4.0)	0 (0.0)	1 (4.0)	25
Nigeria	24 (61.5)	5 (12.8)	5 (12.8)	2 (5.1)	0 (0.0)	3 (7.7)	39
Rwanda	22 (71.0)	5 (16.1)	2 (6.5)	1 (3.2)	1 (3.2)	0 (0.0)	31
Sao Tome and Principe	1 (50.0)	1 (50.0)	0 (0.0)	0 (0.0)	0 (0.0)	0 (0.0)	2
Senegal	22 (64.7)	5 (14.7)	3 (8.8)	3 (8.8)	0 (0.0)	1 (2.9)	34
Sierra Leone	8 (36.4)	11 (50.0)	3 (13.6)	0 (0.0)	0 (0.0)	0 (0.0)	22
South Africa	24 (64.9)	12 (32.4)	1 (2.7)	0 (0.0)	0 (0.0)	0 (0.0)	37
South Sudan	5 (83.3)	1 (16.7)	0 (0.0)	0 (0.0)	0 (0.0)	0 (0.0)	6
Sudan	11 (61.1)	4 (22.2)	1 (5.6)	1 (5.6)	0 (0.0)	1 (5.6)	18
Eswatini	2 (50.0)	0 (0.0)	1 (25.0)	1 (25.0)	0 (0.0)	0 (0.0)	4
Tanzania	3 (75.0)	0 (0.0)	1 (25.0)	0 (0.0)	0 (0.0)	0 (0.0)	4
Tanzania	2 (100.0)	0 (0.0)	0 (0.0)	0 (0.0)	0 (0.0)	0 (0.0)	2
Togo	3 (75.0)	1 (25.0)	0 (0.0)	0 (0.0)	0 (0.0)	0 (0.0)	4
Uganda	53 (59.6)	22 (24.7)	6 (6.7)	7 (7.9)	0 (0.0)	1 (1.1)	89
United Republic of Tanzania	23 (54.8)	14 (33.3)	1 (2.4)	3 (7.1)	0 (0.0)	1 (2.4)	42
Zambia	26 (66.7)	9 (23.1)	2 (5.1)	2 (5.1)	0 (0.0)	0 (0.0)	39
Zimbabwe	23 (76.7)	4 (13.3)	3 (10.0)	0 (0.0)	0 (0.0)	0 (0.0)	30
Total	603	224	75	56	3	29	990

Table S3: Supplementary material: Digital Health Interventions used to strengthen service delivery in sub-Saharan Africa

Name/Reference/ Point of contact	Target -User				Stage of development				Addressed Health System Challenge	Aligned Systems Category
	Clients	Providers	Managers	Data Services	Informal	Pilot	Established	Unknown		
Projecto Exemplar Elon Musk, elon@tesla.com	Yes					Yes			5.Utilization	D. Client communications system
IRS - Cunene Sergio Lopes, sergio@mentor-initiative.net			Yes			Yes			1. Information, 3. Quality, 5. Utilization	F. Community-based information system, V. Public health and disease surveillance, W. Research information system
OpenLMIS Angola (SIGLOFA) Rebecca Alban, info@openlmis.org			Yes				Yes		1. Information, 2. Availability, 6. Efficiency, 8. Accountability	T. Logistics management information system
Angola immunization supply chain Neeraj Thakare, neeraj@logistimo.com			Yes				Yes		1. Information, 2.Availability, 6. Efficiency, 8. Accountability	T. Logistics management information system
The Safe Delivery App in Benin Lauren Bellhouse, lauren@maternity.dk		Yes					Yes		2. Availability, 3. Quality	S. Learning and training system, Q. Knowledge management system
Apprendre a Vivre Benin Uju Ofomata, uju.ofomata@oneworld.org	Yes	Yes						Yes	1. Information, 3. Quality, 5. utilization, 8. Accountability	C. Client applications, S. Learning and training system
OpenLMIS Benin (SIIL) Rebecca Alban, info@openlmis.org		Yes	Yes				Yes		1. Information, 2. Availability, 6. Efficiency, 8. Accountability	T. Logistics management and information system

Integrated e-Diagnostic Approach Thierry Agagliate, tag@tdh.ch		Yes					Yes		1. Information, 3.Quality	N. Health management information system, Q. Knowledge management system,
suivi des diabétiques Sanou Mohamed, sanou45@gmail.com		Yes						Yes	2. Availability, 5. Utilization	D. Client communications ystem
Suivi des malades diabitiques au Burkina Sanou Abdoulaye, sanou45@gmail.com	Yes							Yes	2. Availability,7.Cost	E. Clinical terminology and classifications, Y. Telemedicine
Projjet Télé cardiologie Ouedraogo, sanou60@gmail.com	Yes	Yes						Yes	2. Availability, 7. Cost	Y. Telemedicine
Bio-fortified Value Chains for Improved Maternal and Child Nutrition (B4MCN) Bertin Nduwayo, bertin_nduwayo@wvi.org	Yes					Yes			2. Availability, 3. Quality, 6. Efficiency, 7. Cost	F. Community-based Information System. V. Public health and disease surveillance, Y. Telemedicine
Implementation of a Performance-Based Financing Data System for Cameroon - using the open source tool Hesabu Mfouapon N Henock, National Data Manager of PBF, henockmfouapon@yahoo.fr			Yes				Yes		1. Information, 3. Quality	C. Client applications, N. Health management information system
Bafia District Hospital Armand Mpassy-Nzoumba, armandmpassy@yahoo.com		Yes	Yes			Yes			1. Information, 2. Availability,3. Quality, 6. Efficiency	K. Facility management information system
Bikop Health Center Armand MPASSY, GNU Solidario, armandmpassy@yahoo.com			Yes			Yes			1. Information, 2. Availability, 3. Quality, 6. Efficiency	K. Facility management information System
Klarah Ginyu Innocentia Kwalar, hello@klarah.com		Yes				Yes			2. Availability, 5.Utilization, 6. Efficiency,	K. Facility management system

The Prevention Pack Program: ensuring availability of post-rape medicines in rural clinics Jean Armas, jean.armas@globalstrategies.org		Yes	Yes			Yes			2.Availability, 5. Utilization,	I. Emergency response system
Connecting persons seeking information about HIV/AIDS to HIV medical and social service organizations Jean Armas, jean.armas@globalstrategies.org	Yes					Yes			1. Information	C. Client applications L. Geographic information system, P. Identification registries and directories
Meilleur santé accessible à tous EPENGE DJONGA EMMANUEL, Falbalardc@gmail.com	Yes							Yes	2. Availability, 5. Utilization ,7. Cost	C. Client applications. Y. Telemedicine
OpenELIS Global in Cote d'Ivoire Casey Iiams-Hauser, caseyi@uw.edu		Yes	Yes					Yes	1. Information, 2. Availability, 6.Efficiency	R. Laboratory an diagnostics information system
OpenLMIS Cote d'Ivoire (eSIGL) Rebecca Alban, info@openlmis.org		Yes	Yes				Yes		1. Information, 2. Availability, 6. Efficiency, 8. Accountability	T. Logistics management information system
COVID-19 Vaccine Allocation App Anubhuti Mishra, anubhuti.mishra@thepalladiumgroup.com			Yes				Yes		1. Information, 2. Availability, 6. Efficiency,	F. Community-based Information System
Hiwot: Addressing Maternal & Neonatal Health Stefanie Kot, skot@d-tree.org		Yes				Yes			1. information, 3. Quality, 6. Efficiency	F. Community-based Information System,.Q. Knowledge management system,S. Learning and training system
OppiaMobile Alex Little, consult@alexlittle.net		Yes				Yes			3. Quality	S. Learning and training system

Medical Incident Reporting System Innovation (MIRSI) Fiseha Tesfaw, fiseha.tesfaw@jhpiego.org		Yes				Yes		Yes	1. Information, 4 Acceptability	K. Facility management information system
Blood Safety Strengthening Programme: Blood Safety Information System (BSIS) Rhonwyn Cornell, rhonwyn.cornell@jembi.org			Yes					Yes	1. Information, 2. Availability, 3 Quality, 6. Efficiency	H. Electronic medical record,X. Shared health record and health information repositories
Tenaye Federal Ministry of Health, dha@moh.gov.et	Yes						Yes		1 Information,	C. Client applications,Q. Knowledge management system
Tena PIMS Federal Ministry of Health, dha@moh.gov.et		Yes					Yes		1. Information, 6. Efficiency,	H. Electronic medical record
SuperHIM (Super HIMS health information management system) Federal Ministry of Health, dha@moh.gov.et		Yes	Yes				Yes		1. Information, 6. Efficiency	K. Facility management information system
SmartEMS (Samrt Electronic Medical System) Federal Ministry of Health, dha@moh.gov.et		Yes	Yes				Yes		1. Information, 6. Efficiency	H. Electronic medical record
Quntimed and Quentitb, Federal Ministry of Health, dha@moh.gov.et			Yes				Yes		1. Information, 2. Availability, 6. Efficiency, 8. Accountability	T. Logistics management information system
PRS (Patient Registration System) Federal Ministry of Health, dha@moh.gov.et		Yes					Yes		1. Information	H. Electronic medical record
PrimeCare electronic health record Federal Ministry of Health, dha@moh.gov.et		Yes					Yes		1. Information, 6. Efficiency	H. Electronic medical record

Pipeline Federal Ministry of Health, dha@moh.gov.et			Yes				Yes		1. Information, 2. Availability, 6. Efficiency,8. Accountability	T. Logistics management information system
PaxscanL06 Federal Ministry of Health, dha@moh.gov.et		Yes					Yes		1. Information, 3. Quality	R. Laboratory and diagnostics information system
Patient Registration Software Federal Ministry of Health, dha@moh.gov.et		Yes					Yes		1. Information	H. Electronic medical record
PACS (Picture Archiving and communication System) Federal Ministry of Health, dha@moh.gov.et		Yes					Yes		1. Information, 6. Efficiency	R. Laboratory and diagnostics information system
OTRS (Online Ticket Registration System) Federal Ministry of Health, dha@moh.gov.et			Yes				Yes		6. Efficiency	K. Facility management information System
Orbit Health Federal Ministry of Health, dha@moh.gov.et		Yes					Yes		1. Information, 6. Efficiency	H. Electronic medical record, X. Shared health record and health information repositories
OR management system Federal Ministry of Health, dha@moh.gov.et		Yes					Yes		1. Information	K. Facility management information System
Open Clinic Federal Ministry of Health, dha@moh.gov.et		Yes					Yes		1. Information, 6. Efficiency	H. Electronic medical record, K. Facility management system
mHealth Federal Ministry of Health, dha@moh.gov.et		Yes					Yes		1. Information, 3. Quality	S. Learning and training system
MFR Federal Ministry of Health, dha@moh.gov.et	Yes						Yes		1. Information	P. Identification registries and directories
MEMS (Medical equipment management system) Federal Ministry of Health, dha@moh.gov.et			Yes				Yes		6. Efficiency, 8. Accountability	T. Logistics management information system

Medicine Registration System Federal Ministry of Health, dha@moh.gov.et			Yes				Yes		1. Information, 3. Quality	P. Identification registries and directories, U. Pharmacy Information System
KPI Survey Database Federal Ministry of Health, dha@moh.gov.et			Yes				Yes		1. Information	W. Research information system
IVR (Interactive voice response) Federal Ministry of Health, dha@moh.gov.et		Yes					Yes		1. Information, 2. Availability, 6. Efficiency,8. Accountability	B. Civil registration and vital statistics, T. Logistics management information system
Intellicare Federal Ministry of Health, dha@moh.gov.et		Yes	Yes				Yes		1. Information, 3.Quality	K. Facility management information system
HMIS Brisk Federal Ministry of Health, dha@moh.gov.et		Yes	Yes				Yes		1. Information, 6. Efficiency	H. Electronic medical record
Gx alert Federal Ministry of Health, dha@moh.gov.et		Yes					Yes		1. Information, 6. Efficiency	R. Laboratory and diagnostics information system
Green Stone Digital library Federal Ministry of Health, dha@moh.gov.et		Yes					Yes		1. Information	Q. Knowledge management system
Fleet management system Federal Ministry of Health, dha@moh.gov.et			Yes				Yes		6. Efficiency, 8. Accountability	T. Logistics management information system
EPS pharmaceutical information systems (ARSMBDHS) Federal Ministry of Health, dha@moh.gov.et			Yes				Yes		1. Information, 2. Availability,6. Efficiency, 8. Accountability	T. Logistics management information system
EMR - ART Module Federal Ministry of Health, dha@moh.gov.et		Yes					Yes		1. Information, 6. Efficiency	H. Electronic medical record, X. Shared health record and health information repositories
EGBLS Federal Ministry of Health, dha@moh.gov.et			Yes				Yes		1. Information	K. Facility management information system, T. Logistics management information system

ECHMIS (Electronic Communication HMIS) Federal Ministry of Health, dha@moh.gov.et		Yes					Yes		1. Information, 2. Availability,6. Efficiency, 8. Accountability	T. Logistics management information system
eCHIS - New Federal Ministry of Health, dha@moh.gov.et		Yes					Yes		1.Information, 3. Quality, 6. Efficiency	F. Community based information system, Q. Knowledge management system,
E&RIS (Emergency and referral information system) Federal Ministry of Health, dha@moh.gov.et			Yes				Yes		1. Information, 6.Efficiency	P. Identification registries and directories, T. Logistics management information system
DHMS (Digital Hospital Management System) Federal Ministry of Health, dha@moh.gov.et		Yes	Yes				Yes		1. Information, 6. Efficicency	K. Facility management information system
Dagu Federal Ministry of Health, dha@moh.gov.et		Yes					Yes		1. Information, 2. Availability, 6. Efficiency, 8. Accountability	T. Logistics management information system
Cnet Federal Ministry of Health, dha@moh.gov.et			Yes				Yes		1. Information, 2. Availability, 6. Efficiency, 8. Accountability	T. Logistics management information system
CMMS (Computerized Maintenance Management System) Federal Ministry of Health, dha@moh.gov.et			Yes				Yes		1. Information, 2. Availability, 8. Accountability	T. Logistics management information system
CCEIT Federal Ministry of Health, dha@moh.gov.et		Yes					Yes		1. Information, 2. Availability, 8. Accountability	T. Logistics management information system
CBHI (community based health insurance system) Federal Ministry of Health, dha@moh.gov.et		Yes					Yes		1. Information, 7. Cost	M. Health finance and insurance information system
APTS (auditable pharmaceuticals transactions system) Federal Ministry of Health, dha@moh.gov.et			Yes				Yes		1. Information, 2. Availability, 6. Efficiency, 8. Accountability	K. Facility management information system, U. Pharmacy information system

Antroplus Federal Ministry of Health, dha@moh.gov.et		Yes					Yes		1. Information	R. Laboratory and diagnostics information system
AGHMC EMR Federal Ministry of Health, dha@moh.gov.et		Yes					Yes		1. Information, 6. Efficiency	H. Electronic medical record
ADR (report online) Federal Ministry of Health, dha@moh.gov.et		Yes					Yes		1. Information,	U. Pharmacy Information System
Admission Referral Service Federal Ministry of Health, dha@moh.gov.et		Yes					Yes		1. Information, 6. Efficiency	K. Facility management information system
Life-saving Mobile Health Kits (LMHK) for Obstetric Care in Remote Areas Belay Haffa, belay_haffa@wvi.org		Yes				Yes			2. Availability, 5. Utilization, 6. Efficiency, 7. Cost	F. Community-based Information System, Q. Knowledge Management system, Y. Telemedicine
Implementation of a LIMS based on GNU Health at the International Center for Medical Research of Franceville Armand Mpassy-Nzoumba, armandmpassy@yahoo.com		Yes				Yes			1. Information, 6. Efficiency,	R. Laboratory management information system
Suivi des malades diabétiques Mr Dupont, dupont58@gmail.com	Yes	Yes						Yes	1. Information, 6. Efficiency, 7. Cost	C. Client applications, Y. Telemedicine
Hello Nurse Darlene Irby, Darlene.Irby@jhpiego.org		Yes				Yes			3. Quality	S. Learning and training system
Blood Safety Strengthening Programme: Blood Safety Information System (BSIS) Implementation Rhonwyn Cornell, rhonwyn.cornell@jembi.org		Yes	Yes					Yes	1. Information, 3. Quality	H. Electronic medical record, X. Shared health record and health information repositories

The Safe Delivery App in Ghana Lauren Bellhouse, lauren@maternity.dk		Yes					Yes		2. Availability, 3. Quality	Q. Knowledge Management System, S. Learning and training system
MNCH mobile content with nutrition focus, available in Twi, Ewe, Hausa and English in SMS and voice transcript format Kim Viljoen, kviljoen@gsma.com		Yes				Yes			1. Information	C. Client application, D. Client communications system
LBG Covid-19 Response Tang Simon, simon@literacybridge.org		Yes				Yes			1. Information	C. Client application, D. Client communications system, Q. Knowledge management system
MEDTRACK Omar Seidu Farouk, omar@medtrack.com.gh		Yes				Yes			1. Information, 6. Efficiency	H. Electronic health record, X. Shared health record and health information repositories
Carely Digital Health Platform Kelvin Ashie, healthdirectglobal@gmail.com		Yes				Yes			1. Information, 2. Availability, 6. Efficiency, 7. Cost	C. Client applications, H. Electronic health record, M. health finance and insurance information system
Suivi de la vaccination de routine des enfants de moins 5 ans Dr Souma, souma58@gmail.com		Yes						Yes	1. Information	D. Client communications ystem,
OpenLMIS Guinea Rebecca Alban, info@openlmis.org			Yes				Yes		1. Information, 2. Availability, 6. Efficiency	T. Logistics management information system
cStock/DHIS2 Zoya Mohammed, zoya_mohamed@insupplyhealth.com			Yes			Yes			1. Information, 2. Availability ,6. Efficiency, 8. Accountability	F. Community-based Information System, T. Logistics management information system
personal women's health advice via mobile chat MacGregor, Mac@lily.health	Yes							Yes	1. Information, 2. Availability, 4. Acceptability, 5. Utilization	C. Client application

Scaling of BLIS(Basic Laboratory Information System) 3.0 Implementation Across Africa Emmanuel Kweyu, ekweyu@strathmore.edu	Yes						Yes		1. Information	R. Laboratory and diagnostic information system
Medic App in Siaya County in partnership with Ministry of Health Michael Korir, korir@medicmobile.org	Yes					Yes			1. Information, 2. Availability	F. Community-based Information System, Q. Knowledge Management
Regional Action Through Data (RAD) Laurie Markle, LMarkle@brhc.com	Yes					Yes			1. Information, 5. Utilization	H. Electronic health record, X. Shared health record and health information repositories
Malaria Test Tim Nichols, tim.nichols@vitalwave.com	Yes					Yes			2. Availability, 6. Efficiency	C. Client application
SmartHealth App in Kenya Nii Amon Dsane, ndsane@livinggoods.org		Yes	Yes					Yes	1. Information, 2. Availability, 5. Utilization, 6. Efficiency	F. Community-based Information System,H. Electronic medical record, Q. Knowledge management system
MNCH mobile content with nutrition focus, available in Swahili and English in SMS and voice transcript format Kim Viljoen, kviljoen@gsma.com	Yes					Yes			1. Information	C. Client application, D. Client communications system, Q. Knowledge management system
Keheala - Digital adherence technology that uses behavioral 'nudges' and basic feature phones to iA139:J139mprove health outcomes. Jon Rathauser, jonr@keheala.com	Yes					Yes			1. Information	C. Client applications,D. Client communications system,Q. Knowledge Management system

Medic App in Elgeyo Marakwet Susan Maigua, susan@medicmobile.org		Yes				Yes			5. Utilization,	Q. Knowledge management system
Digital African Health Library Dr Bruce Dahlman, director@digitalhealthlibrary .net		Yes						Yes	3. Quality	Q. Knowledge management system
Improving Healthcare Capacity and Access Using THINKMD's Clinical Decision Support Tool Used by Private Healthcare Providers Meg McLauhlin, mmclaughlin@thinkmd.org		Yes				Yes			1. Information, 3. Quality	Q. Knowledge Management system, U. Pharmacy information system
Safe Abortion App Sarah Shannon, Executive Director, SJHOLCOMBE@gmail.CO M	Yes							Yes	1. Information,5. Utilization, 8. Accountability	C. Client application, Q. Knowledge management system
Empower Health Molly Guy, molly.guy@medtronic.com	Yes	Yes							2. Availability, 5. Utilization, 6. Efficiency, 7. Cost	C. Client applications, D. Client communications system, Y. Telemedicine
M-Sawazisha: Integrating Mobile Technology in MNCH in Afya Jijini Michael Korir, korir@medicmobile.org		Yes				Yes			1. Information, 2. Availability, 5. Utilization,	B. Civil Registration and Vital Statistics. Community-based Information System,
PROMPTS - Promoting Mothers during Pregnancy through SMS Rachel M. Jones, Rjones@jacarandahealth.org	Yes					Yes			5. Utilization,	D. Client communication system

Medkit.Network Dr. Louis Somoni Machogu, dr.somoni@gmail.com	Yes							Yes	1. Information, 3. Quality	C. Client applications, P. Identification registries and directories, U. Pharmacy information system
SASAdoctor Telemedicine Android App Francis Osiemo, francis.osiemo@sasadoctor.com	Yes	Yes				Yes			2. Availability, 3. Quality,6. Efficiency, 7. Cost	Y. Telemedicine
Kenya Quality Model For Health e-tool Steven Wanyee, swanyee@intellisoftkenya.com			Yes				Yes		3. Quality	K. Facility management information system
Bethany Kids Kijabe Hospital Steven Wanyee, swanyee@intellisoftkenya.com		Yes	Yes				Yes		1. Information , 6. Efficiency	H. Electronic medical record
Kenya Health & Empowerment Wade Munday, wmunday@addisclinic.org		Yes				Yes			2. Availability, 3. Quality , 6. Efficiency, 7. Cost	Y. Telemedicine
e-Hospital Steven Wanyee, swanyee@intellisoftkenya.com			Yes				Yes		1.Information, 6. Efficiency, 8. Accountability	K. Facility management information system
Mobile Solutions for Women and Children's Health (Mobile WACh) Keshet Ronen, keshet@uw.edu	Yes	Yes				Yes			1. Information, 2. Availability, 5. Utilization, 8. Accountability	C. Client applications, D. Client communications system
DawaSure francis munyeki, munyekif@cadcreations.co.ke	Yes					Yes			2. Availability, 5.Utilization	C. Client applications, U. Pharmacy information system
Living Goods- Community Health Innovation Network (CHIN) Fransicah Nzanga, fnzanga@livinggoods.org	Yes	Yes				Yes			1. Information, 6. Efficiency	F. Community-based information system,

Lwala Community Alliance x OpenFn: Building a real-time decision support tool to strengthen healthcare in western Kenya Aleksa Krolls, aleksa@openfn.org	Yes	Yes					Yes		3. Quality	Q. Knowledge management system
COVID-19 Service Delivery Tracker Liz McNeil, lmcneil@jacarandahealth.org			Yes			Yes			1. Information	W. Research Information System
KenyaEMR Pascal Mwele, Pascal.Mwele@thepalladium group.com			Yes				Yes		1. Information, 6.Efficiency	H. Electronic medical record
Afya Mobile Pascal Mwele, pascal.mwele@thepalladium group.com		Yes					Yes		1. Information	F. Community-based information system
ENDCORONAVIRUS.CO. KE Katherine Piets, kat@every1mobile.com	Yes					Yes			1. Information	C. Client application
Case Management Mobile App for Children with Disabilities Jessica Charles, jessica@kupenda.org		Yes				Yes			1. Information, 2. Availability, 5. Utilization	F. Community-based Information System,
Ohospital Daniel Kimani, suhadetechkenya@gmail.co m	Yes	Yes				Yes			2. Availability, 7. Cost	Y. Telemedicine
PaperEMR Pratap Kumar, pratap@health-e-net.org	Yes					Yes		Yes	1. Information ,6. Efficiency	H. Electronic medical record
Lesotho eRegister Monaheng Maoeng, monaheng.maoeng@gov.ls	Yes							Yes	1. Information, 6. Efficiency	H. Electronic medical record, X. Shared Record and health information repositories

Blood Safety Strengthening Programme: Blood Safety Information System (BSIS) Implementation Rhonwyn Cornell, rhonwyn.cornell@jembi.org	Yes								1. Information, 6. Efficiency	H. Electronic Medical Record, X. Shared Health Record and health information repositories
Lab Information System Malebanye Lerotholi, 4malbe@gmail.com	Yes						Yes		1. Information	R. Laboratory and diagnostics information system
SMS Notification System Tsele Moloeli, moloet@yahoo.com	Yes							Yes	1. Information,5. Utilization	D. Client communications system
Electronic Logistics Management and Inventory Management System Monaheng Maoeng, monaheng.maoeng@gov.ls	Yes						Yes		1. Information 2. Availability , 6. Efficiency, 8. Accountability	T. Logistics and diagnostics information system
CITIZEN APP Pule Phafane, pule@smartmycompany.com			Yes					Yes	1. Information,	I. Emergency response system , V. Public health and disease surveillance system , W. Research information system
Client Tracking Application Sele Mthimkhulu, smthimmkhulu@pedaids.org	Yes					Yes			5. Utilization,	D. Client communications system
mHero Liberia IntraHealth International, mHero@intrahealth.org		Yes	Yes				Yes		1. Information,	I. Emergency response system , V. Public health and disease surveillance system
mTOMADY Jennifer Bencivenga, jb@mtomady.com	Yes	Yes				Yes			7.Cost	M. Health finance an insurance information system
USAID ACCESS m-health initiative Stephanie Ranaivo, sranaivo@usaidaccess.org		Yes					Yes		1. Information, 3. Quality	F. Community-based Information System, N. Health management information system

The Malawi Digital Village Clinic (DVC) Christopher Kulanga, chriskulanga@gmail.com		Yes				Yes			1. Information, 2. Availability, 3. Quality,	F. Community-based Information system, Q. Knowledge Management system
cStock - EPI Erick Mwale, Erick.Mwale@savethechildren.org		Yes					Yes		1. Information, 2. Availability, 6. Efficiency , 8. Accountability	T. Logistics management information system
Medic at St Gabriel's Hospital Alex Ngalande, unknown@yahoo.com		Yes				Yes			1, Information	B. Civil Registration and Vital Statistics
ONSE - Mobile Village Toolkit Rudi Thetard - ONSE Chief of Party, rthetard@msh.org			Yes			Yes			1. Information, 2. Availability, 3. Quality	F. Community-based Information System, Q. Knowledge management system
ONSE - Integrated Supportive Supervision Toolkit Rudi Thetard - ONSE Chief of Party, rthetard@msh.org			Yes			Yes			1.Information, 3.Quality	N. Health management information system
Supporting LIFE Joseph WU, wcsg@lukeinternational.no			Yes			Yes			1. Information,	W. Research Information System
Malawi electronic integrated disease surveillance and response system (Malawi eIDSR) Joseph Wu, wcsg@lukeinternational.no		Yes	Yes				Yes		1. Information, 6. Efficiency	H. Electronic medical records, V. Public health and disease surveillance system
One Community Steve Choko, stevec@jhuccpmw.org		Yes				Yes			1. Information	F. Community based information system
Malawi EID ART Initiation Tracking Sean Blaschke, sblaschke@unicef.org		Yes					Yes		1. Information,	R. Laboratory and diagnostic information system

Chipatala Cha Pa Foni (CCPF) Health Center by Phone Malawi Carla Blauvelt, carla.blauvelt@villagereach.org	Yes						Yes		2. Availability, 6. Efficiency	C. Client application
Barr Foundation iCCM mHealth project Erica Layer, elayer@d-tree.org			Yes			Yes			1. Information	F. Community based health information system, Q. Knowledge management system
CMAM Stock Monitoring System Malawi Sylvester Kathuma, kathumbasylvester@gmail.com			Yes					Yes	1. Information	T. Logistics management information system
Medic Mobile DIY Malawi Jacqueline Edwards, jacqueline@medicmobile.org		Yes						Yes	1. Information, 6.Efficiency	H. Electronic medical record
MNCH mobile content with nutrition focus, available in Chichewa, Tumbuka and English in SMS and voice transcript format Kim Viljoen, kviljoen@gsma.com	Yes					Yes			1. Information	C. Client applications, D. Client communications system, Q. Knowledge management system
OpenLMIS Malawi Rebecca Alban, info@openlmis.org		Yes	Yes				Yes		1. Information, 2. Availability, 6. Efficiency, 8. Accountability	T. Logistics management information system
Fetal Monitoring device and mobile decision support Chris Kulanga, Ckulanga@D-tree.org		Yes						Yes	5. Utilization	Q. Knowledge management system
Electronic Participant Locator (ePAL) Luke Banda, lsbanda@mlw.mw			Yes			Yes			1. Information	I. Emergency response system, V.Public health and disease surveillance system

Advanced mobile toolkit alongside Muso's ProCCM model in Mali Fatou Fall, fatou@medicmobile.org		Yes	Yes					Yes	3. Quality, 6. Efficiency	F. Community based information system
BIOWINPHARMA Mamadou Dakouo, contact@biowinpharma.com		Yes					Yes		1. Information, 6. Efficiency	H. Electronic Medical Record, X. Shared health record and health information repositories
Access to Infant and Maternal (AIM) Health Plus Mauritania Zeine Abidine Ba, zeine_abidine@wvi.org		Yes				Yes			1. Information 2. Availability, 3. Quality, 5. Utilization	F. Community - based information system, Q. Knowledge Management system
upSCALE Joaquim Rebelo, j.rebelo@malariaconsortium.org		Yes					Yes		1. Information, 2. Availability, 3. Quality, 5. Utilization	F. Community -based information system, Q. Knowledge management system
MNCH mobile content with nutrition focus, available in 4 languages in SMS and voice transcript format Kim Viljoen, kviljoen@gsma.com	Yes					Yes			1. Information	C. Client application, D. Client communications system
OpenLMIS Mozambique (SELV) Rebecca Alban, Info@openlmis.org		Yes	Yes				Yes		1. Information, 2. Availability, 6. Efficiency , 8. Accountability	T. Logistics management information system
OpenLMIS Mozambique (SIGLUS) Rebecca Alban, Info@openlmis.org		Yes	Yes				Yes		1. Information, 2. Availability, 6. Efficiency,8. Accountability	T. Logistics management information system
PENSA Valter Cumbi, vcumbi@sourcecode.solutions	Yes						Yes		1. Information,2. Availability, 5. Utilization	C. Client applications, Q. Knowledge management system
Namibia Master Facility List Maria Helao, Maria.Helao@mhss.gov.na			Yes				Yes		1. Information, 5. Utilization	L. Geographic information system (GIS), X. Shared health record and health information repositories

Namibia Planned Parenthood Association (NAPPA) Erin Sullivan, erin.sullivan@jhpiego.org		Yes						Yes	1. Information, 6. Efficiency	H. Electronic medical record
Act to Save (A2S) Ezéchiel Mahamane, ezechiel_mahamane@wvi.org		Yes				Yes			1. Information, 2. Availability	F.Community-based information system,Q. Knowledge management system
HelloMAMA Steve Ollis, steve.ollis@mcsprogram.org		Yes				Yes			1. Information, 5. Utilization	D. Client communications system,
USAID funded - CaTSS OpenMRS Electronic Medical Record System for 19 ART Clinics Benjamin Akinmoyeje, bakinmoyeje@msh.org		Yes	Yes					Yes	1. Information, 6. Efficiency	H. Electronic medical record
WeMUNIZE Steve Ollis, steve.ollis@mcsprogram.org		Yes							1. Information, 5. Utilization	D. Client communications system
MNCH mobile content with nutrition focus, available in 5 local languages in SMS and voice transcript format Kim Viljoen, kviljoen@gsma.com	Yes	Yes				Yes			1. Information	C. Client application, D. Client communications system
NaijaCare Dr Saidat Akanbi, saidat@every1mobile.com			Yes			Yes			2. Availability, 5. Utilization	P. Identification registries and directories
NaijaCare Project Moshood Abdullateef, moshood@every1mobile.com		Yes				Yes			2. Availability,3. Quality	S. Learning and training system
Complete Health Nneka Mobisson, nneka.mobisson@mymdoc.com	Yes							Yes	1. Information	C. Client applications
Wellvis Wale Adeosun, wale@wellvis.org	Yes	Yes							2. Availability, 3. Quality ,5.	Y. Telemedicine

									Utilization, 7. Cost	
SwissTPH x OpenFn: Automated, real-time data integration & disease monitoring Aleksa Krolls, aleksa@openfn.org		Yes	Yes				Yes		1. Information, 6. Efficiency	H. Electronic medical record, N. Health management information system,
Audio Job Aids for Family Planning Providers Emily Mangone, emily_mangone@abtassoc.com		Yes					Yes		2. Availability, 3. Quality	Q. Knowledge management system
HelpMum Dr Abiodun Adereni, abbey2real@gmail.com		Yes					Yes		1. Information, 2. Availability,3. Quality	Q. Knowledge management system S. Learning and training system
Real-Time Monitoring of Moderna Vaccines in collaboration with National Primary Health Care Development Agency. Oghenetega Iortim, tega@gricd.com		Yes	Yes				Yes		1. Information, 3. Quality	T. Logistics management information system
ulerawa luke shors, lshors@icloud.com			Yes			Yes			7. Cost	M. Health finance and insurance information system
Quick Health Chris Agape Ajah, hello@getquickhealth.com	Yes					Yes			1. Information, 2. Availability	P. Identification registries and directories
PHARMARUN IBITAYO TAIWO, tayotaiwo83@yahoo.com	Yes							Yes	2. Availability	C. Client applications
EmHealth-Paediatric Digital Health Initiative Dr Agbarakwe Chukwuemeka, brammy@emhealthglobal.org		Yes						Yes	1. Information, 6. Efficiency	H. Electronic medical record

OneCare Hafeez Ayanda, hayanda@innovastra.com	Yes	Yes						Yes	1. Information, 6. Efficiency, 7. Cost	H. Electronic medical record, K. Facility management information system, Y. Telemedicine
E-Heza Data Solutions Wendy Leonard, MD, AAHIVS, wendy@theihanganeproject.com		Yes					Yes		1. Information, 3. Quality	Q. Knowledge management system
Rwandan digital ANC module Mr Felix Sayinzoga, felix.sayinzoga@rbc.gov.rw		Yes	Yes			Yes			1. Information, 3. Quality	Q. Knowledge management system
JoCare Jerome Nshimiyimana, njerome05@gmail.com	Yes					Yes			1. Information	C. Client application
Automation and AI for Health Campaign Planning Sangeeta Jobanputra, sraja@connecti3.com			Yes			Yes			1. Information, 6. Efficiency	T. Logistics management information system
Suivi nutritionnel des enfants de moins de 5 ans dans la région de Lobata Eduardo, ferreirae@who.int	Yes	Yes						Yes	2. Availability, 5. Utilization, 7.Cost	Y. Telemedicine
Implementation of a Performance-Based Financing Data System for Senegal - using the open source tool Hesabu Dr Malick Cissé Ndiaye, illeyya@yahoo.fr			Yes				Yes		1. Information, 3. Quality	K. Facility management information system
Suivi des grossesses dans la région de Kaolack Dr Dia, nourougaloya@gmail.com	Yes	Yes						Yes	2. Availability, 5. Utilization, 7.Cost	Y. Telemedicine
Informed Push Model - Yeksi-Naa Melanie Joiner, mjoiner@intrahealth.org			Yes					Yes	1. Information, 2. Availability, 6. Efficiency, 8. Accountability	T. Logistics management information system

Senegal immunization supply chain Neeraj Thakare, neeraj@logistimo.com			Yes			Yes			1. Information, 2. Availability, 6. Efficiency, 8. Accountability	T. Logistics management information system
Aarleen mHealth System Gorgui Diallo, gdiallo@africare.org		Yes					Yes		1. Information, 8. Accountability	Q. Knowledge management system
VaxTrac Dayo Spencer-Walters, dayo.spencer-walters@sl.ehealthafrica.org		Yes						Yes	1. Information, 6. Efficiency	H. Electronic medical records
Electronic Vaccination Record and Tracking System Implementation Project (VaxTrac) Musa Bernard Komeh, musa.komeh@sl.ehealthafrica.org		Yes						Yes	1. Information, 6. Efficiency	H. Electronic medical records, Q. Knowledge management system
Saving Lives Mohammad B. Jalloh, mbjalloh@focus1000.org		Yes							1. Information, 5. Utilization, 6. Efficiency	H. Electronic medical records
RapidPro Kazutaka Sekine, ksekine@unicef.org		Yes	Yes				Yes		1. Information,	N. Health management information system
Mobile Training and Support (MOTS) Frederick F. Kamara (World Vision-SL), Fredrick_kamara@wvi.org		Yes						Yes	2. Availability, 3. Quality,	S. Learning and training system
The Safe Delivery App in Sierra Leone Lauren Bellhouse, lauren@maternity.dk		Yes					Yes		2. Availability, 3. Quality	S. Learning and training system
Access to Infant and Maternal (AIM) Health Plus Sierra Leone Joseph Musa, joseph_musa@wvi.org		Yes				Yes			1. Information, 2. Availability,	F. Community-based information System, Q. Knowledge management system

MOTS COVID-19 Monica Amponsah, mamponsah@grameenfoundation.org		Yes						Yes	2. Availability, 3. Quality	S. Learning and training system
Public Health Incentives Gugulethu Nyathi, gugu@ribbonblockchain.com	Yes							Yes	2. Availability	C. Client application
NACOSA Health Analytics Platform (NHAP) Safa Naraghi, safa@zenysis.com			Yes				Yes		1. Information,	N. Health management information system
BroadReach COVID-19 Pandemic Management Fiona de Korte, fdekorte@brhc.com		Yes	Yes				Yes		1. Information, 2. Availability,5. Utilization , 6. Efficiency	N. Health management information system, V. Public health and disease surveillance system
Vula Mobile William Mapham, william@vulamobile.com		Yes						Yes	2. Availability, 3. Quality, 5. Utilization, 7. Cost	Y. Telemedicine
eLQAS: Collecting real-time polio vaccination data Yaw Anokwa, yanokwa@nafundi.com		Yes							1. Information	N. Health management information system
e-Hospital - South Sudan Steven Wanyee, swanyee@intellisoftkenya.com		Yes					Yes		1. Information, 6. Efficiency,	K. Facility management information system
Integrated Humanitarian Data Package for public health data needs Jonathan Hanson, jhanson@mapaction.org			Yes					Yes	1. Information	T. Logistics management information system

Improving Healthcare Capacity and Access Using THINKMD's Clinical Decision Support Tool in a Humanitarian Setting Meg McLaughlin, mmclaughlin@thinkmd.org		Yes				Yes			1. Information, 6. Efficiency	H. Electronic medical record
eLMIS Facility Edition (FE) Ashraf Islam, ashraf_islam@jsi.com			Yes					Yes	1. Information, 2. Availability,6. Efficiency, 8. Accountability	T. Logistics management information system
The Safe Delivery App in Togo (part of DI 2018 Project) Lauren Bellhouse, lauren@maternity.dk		Yes					Yes		2. Availability, 3. Quality	Q. Knowledge management system
Digital Tools for CHWs with Integrate Health and Medic Mobile Fatou Fall, fatou@medicmobile.org		Yes				Yes			1. Information, 2. Availability ,3. Quality	Q. Knowledge management system
Télédermatologie TOGO Palokinam PITCHE, vincent.pitche@gmail.com		Yes				Yes			2. Availability, 3. Quality, 7. Cost	Y. Telemedicine
UgandaEMR (OpenMRS) Jonathan Mpango, jmpango@musph.ac.ug		Yes					Yes		1. Information , 6. Efficiency	H. Electronic medical record
: Improving outcomes in HIV patients usingmobile phone based interactive software support Rosalind Parkes Ratanshi, rp549@medschl.cam.ac.uk		Yes						Yes	1. Information, 5. Utilization	D. Client communications system,
ART ACCESS Dr Rosalind Parkes-Ratanshi, rp549@medschl.cam.ac.uk		Yes				Yes			1. Information, 5. Utilization, 6. Efficiency	H. Electronic medical record, R. Laboratory and diagnostics information system

Connect for Life - Infectious Disease Institute (IDI) Theresa Pattery, tpattery@its.jnj.com	Yes					Yes			1. Information	C. Client applications
STRE@MLINE Mr Samuel Mugisha, CEO Innovation Streams Ltd, samuel@innovationstreams.tech		Yes				Yes			1. Information, 6. Efficiency,	K. Facility management information system
REFERENCE LABORATORY INFORMATION SYSTEMS (EID/VL LIMS) PROSCOVIA NAMBUYA MBABAZI, pronam2000@gmail.com		Yes					Yes		1. Information, 6. Efficiency	R. Laboratory and diagnostics information systems
Access to Infant and Maternal (AIM) Health Plus Uganda Racheal Auma, racheal_auma@wvi.org		Yes					Yes		2. Availability, 3 Quality,	Q. Knowledge management system
SmartHealth App in Uganda Nii Amon Dsane, ndsane@livinggoods.org		Yes						Yes	2. Availability, 3. Quality	F. Community -based information system, Q. Knowledge management system, T. Logistics management information system
MNCH mobile content with nutrition focus, available in 5 languages in SMS and voice transcript format Kim Viljoen, kviljoen@gsma.com	Yes	Yes				Yes			1. Information	C. Client applications, D. Client communications system

NoviGuide: Tablet-based clinical decision support bringing neonatal expertise to the bedside while uncovering barriers to care Joshua Bress, josh.bress@globalstrategies.org		Yes				Yes			3. Quality	Q. Knowledge and management system
The Zone Raquel Palomino Gonzalez, lund@unfpa.org	Yes					Yes			1. Information	C. Client applications , Q. Knowledge management system
Bringing Back Mothers and Babies Kisaakye Linda, lkisaakye@gmail.com		Yes						Yes	1. Information, 5. Utilization, ,6. Efficiency	H. Electronic medical record
DREAMS/OVC Juliet Cheptoris, julietcheptoris@gmail.com		Yes	Yes					Yes	1. Information, 6. Efficiency	H. Electronic medical record, N. Health management information system,
Early Infant Diagnosis(EID) Dashboard Dr. Victor Bigira, vbigira@musph.ac.ug		Yes	Yes					Yes	1. Information, 6. Efficiency	H. Electronic medical record
HiMap Mobile Application Jonathan Mpango, jmpango@musph.ac.ug	Yes	Yes						Yes	1. Information	P. Identification registries and directories
RxSolution Stephanie Xueref, smxueref@msh.org		Yes	Yes			Yes			1. Information, 2. Availability,	T. Logistics management information system
Pharmaceutical Information Portal Stephanie Xueref, smxueref@msh.org				Yes			Yes		1. Information,	N. Health management information system
Option B+ Kisaakye Linda, lkisaakye@gmail.com		Yes						Yes	1. Information, 5. Utilization	N. Health management information system

Smart Hospital Information System (SmartHIS) Dr Simon Ndira, simon.ndira@compelling.works		Yes				Yes			1. Information, 6. Efficiency	K. Facility management information system
Performance Tracking App for CHW Supervisors Simon Mutama, smutama@livinggoods.org		Yes	Yes					Yes	1. Information, 3. Quality,	F. Community-based information system, O. Human resources information system, Q. Knowledge management system
mTrac Uganda Carol Kyozira, ckyozira@gmail.com		Yes	Yes					Yes	1. Information, 2. Availability, 6. Efficiency, 8. Accountability	T. Logistics management information system
FamilyConnect Dr. Jesca Nsungwa Sabiiti, jnsabiiti@gmail.com		Yes						Yes	1. Information	B. Civil Registration and Vital Statistics, F. Community-based information system,
Stre@mline Samuel Mugisha, samuel@streamlinehealth.org		Yes						Yes	1. Information, 5. Utilization, 6. Efficiency	H. Electronic medical record, D. Client communications system
Open Client Registry Wayan Vota, wvota@intrahealth.org		Yes						Yes	1. Information , 6. Efficiency	H. Electrronic medical record, X. Shared health record and health information repositories
USAID RHITES-N, Lango Med Makumbi, med_makumbi@ug.jsi.com		Yes						Yes	1. Information, 5. Utilization	D. Client communications system
Pfizer Project Bugiri Elisha Nangosha, elisha_nangosha@wvi.org		Yes				Yes			3. Quality,	Q. Knowledge and management system ,S. Learning and training system
Buikwe Maternal Newborn and Child Health (B-MNCH) Project Angella Akolla, angella_akolla@wvi.org		Yes				Yes			1. Information, 8. Accountability	Q. Knowledge management system

Kampala Emergency Digital Emergency System Douglas Akii Bua, dbua@psiug.org		Yes				Yes			1. Information, 5. Utilization	F. Community based information system
Electronic Inventory Management Services System (e-IMSS) Cliton Chikwata, clinton.chikwata@tmcg.co.ug		Yes					Yes		1. Information, 2. Availability	T. Logistics management information system
Public Health Open Community Mapping Kiggudde Deogratius, deogratias@mapuganda.org		Yes				Yes			1. Information	F. Community-based Information System, L. Geographic information system
OpenONCIMS Dr Wasswa William, williamwasswa036@gmail.com		Yes							1. information, 3. Quality, 6. Efficiency	R. Laboratory and diagnostics information system
Vaccine Information Management System (VIMS) Ssanyu Nyinondi, snyinondi@tz.jsi.com			Yes	Yes			Yes		2. Availability	T. Logistics management information system, G. Data interchange interoperability and accessibility
Safer Deliveries Rachel Hofmann, rhofmann@d-tree.org		Yes					Yes		1. Information, 2. Availability	Q. Knowledge management system
Mobilizing Maternal Health (MMH) Gloria Kahamba, gkahamba@d-tree.org	Yes							Yes	2. Availability, 5. Utilization, 6. Efficiency	I. Emergency response system
Community Family Planning and Model Household Systems; Population Health Environment (PHE) Rebecca Litner, rlitner@d-tree.org		Yes						Yes	1. Information, 5. Utilization, 8. Accountability	F. Community based information system, Q. Knowledge management system

Sauti- HIV Prevention Program Gloria Kahamba, gkahamba@d-tree.org		Yes						Yes	1. Information, 5. Utilization, 6. Efficiency	H. Electronic medical record
MCSP Faclity and Community MCH application Steve Ollis, Steve.ollis@mcsprogram.org		Yes						Yes	, 2. Availability, 3. Quality	Q. Knowledge management system
BID INITIATIVE Hassan Mtenga, Laurie Warner, hmtenga@path.org		Yes						Yes	1. Information, 5. Utilization, 6. Efficiency	H. Electronic medical record
Non-discriminating access for Digital Inclusion (DigI) Christine Holst, christine.holst@medisin.uio.no	Yes					Yes			1. Information,	C. Client application
MNCH mobile content with nutrition focus, available in Kiswahili and English in SMS and voice transcript format Mojca Cargo, mcargo@gsma.com	Yes	Yes				Yes			1. Information	C. Client applications, D. Client communications system
The Safe Delivery App-Tanzania Helena Lassen, helena@maternity.dk		Yes				Yes			2. Availability, 3. Quality	Q. Knowledge management system
USAID BORESHA AFYA - COMMUNITY HEALTH INFORMATION SYSTEM ENCOMPASSING OPEN SMART REGISTER PLATFORM, CLOSED USER GROUP & ELEARNING Dunstan Bishanga, Dunstan.Bishanga@jhpiego.org		Yes				Yes			1. Information, 2. Availability, 5. Utilization, 6. Efficiency	H. Electronic medical record, I. Emergency response system, S. Learning and training system

SAUTI PROJECT-KVP MOBILE DATA COLLECTION SYSTEM Dr Albert Komba, Albert.Komba@jhpiego.org		Yes						Yes	1. Information,	F. Community-based health information system
Clinical Monitoring System for Preservice Trainees-More and Better Midwives for Rural Tanzania Project Dr Julius Masanika, Julius.Masanika@jhpiego.org		Yes						Yes	1. Information, 3. Quality,	O. Human resources information system
Family Planning Digital Health System for Community Health Workers - Shinyanga Region Gloria Kahamba, gkahamba@d-tree.org		Yes						Yes	1. Information,	F. Community-based Information System,
Rheumatic Heart Disease and Hypertension Tracking Stefanie Kot, skot@d-tree.org		Yes						Yes	1. Information 2. Availability, 5. Utilization ,6. Efficiency	H. Electronic medical record, O. Human resources information system
OpenLMIS Tanzania Rebecca Alban, Info@openlmis.org			Yes				Yes		1. Information, 2. Availability ,6. Efficiency, 8. Accountability	T. Logistics management information system
DYNAMIC Prof. Valérie D'Acremont, head of digital and global health at Unisanté, Lausanne or Dr. Irene Masanja, senior scientist at Ifakara Health Institute, Dar es Salaam, valerie.dacremont@swisstph.ch		Yes						Yes	1. Information, 3. Quality, 5. Utilization, 6. Efficiency	N. Health management information system,Q. Knowledge Management,R. Laboratory and Diagnostic System, S. Learning and training systems
Jamii ni Afya Heiko Hornung, hhornung@d-tree.org		Yes					Yes		1. Information	F. Community-based information system, Q. Knowledge management system

Afya-Tek: Digital referral and decision support system using biometrics across the continuum of care Rebecca Litner, Rlitner@d-tree.org		Yes						Yes	5. Utilization,	I. Emergency response system
Access to Infant and Maternal (AIM) Health Plus Tanzania Daudi Sosthen Gambo, daudi_gambo@wvi.org		Yes				Yes			1. Information, 2. Availability, 5. Utilization	F. Community-based information system, Q. Knowledge management system
Smart Growth Diary Mwombeki Fabian, info@fabstech.com	Yes							Yes	2. Availability, 3. Quality, 4. Acceptability	C. Client application,D. Client communications system
AfyaIntelligence Harrison Mariki, afyaintelligence@gmail.com		Yes	Yes					Yes	1. Information, 2. Availability, 8. Accountability	T. Logistics management information system , N. Health management information system
Hyperlocal Geospatial Data for Improving Vaccination Campaigns Asha Mustapher, omdtanzania@gmail.com			Yes					Yes	1. Information	F. Community-based information system, L. Geographic information system,
mSpray` Anne Martin, acmartin@akros.com			Yes					Yes	1. Information,	F. Community-based information system
eLMIS Facility Edition (FE) Wendy Bomett, wendy_bomett@zm.jsi.com		Yes	Yes					Yes	1. Information, 2. Availability ,6. Efficiency, 8. Accountability	T. Logistics management information system
MNCH mobile content with nutrition focus, available in Bemba, Nyanja and English in SMS and voice transcript format Kim Viljoen, kviljoen@gsma.com	Yes	Yes				Yes			1. Information	C. Clients applications, D. Client communications ystem

ZEIR - Zambia Electronic Immunization Registry Laurie Werner, lwerner@path.org		Yes					Yes		1. Information, 5. Utilization , 6. Efficiency	H. Electronic medical record
Zambia health Analytics Platform (ZHAP) Kaluba Mataka, kaluba@zenysis.com			Yes				Yes		1. Information,	N. Health management information system, T. Logistics management information system
OpenLMIS Zambia Rebecca Alban, Info@openlmis.org		Yes	Yes				Yes		1. Information, 2. Availability, 6. Efficiency ,8. Accountability	T. Logistics management information system
DelphiCare: Clinical decision support for comprehensive, longitudinal care of HIV-exposed and infected children and adolescents Douglas Watson, MD, douglaswatsonmd@gmail.com		Yes						Yes	3. Quality ,7.Cost	Q. Knowledge management system,Y. Telemedicine
Zambia immunization supply chain Arun Ramanujapuram, arun@logistimo.com		Yes	Yes				Yes		1. Information, 2. Availability, 6. Efficiency, 8. Accountability	T. Logistics management information system
IDeAS. Integrated Decisions and Analytics Support. Mandy Dube, MDube@brhc.com		Yes					Yes		1. Information, 6. Efficiency	H. Electronic medical records
SmartCare Electronic Health Record System Andrew Kashoka, andrew.kashoka@moh.gov.zm		Yes					Yes		1. Information, Efficiency	H. Electronic medical record
Zambia Anternatal Care (ANC) Digital Adapation Project I Implementation Research Caren Chizuni, carenchizuni@gmail.com		Yes				Yes			1. Information , 3. Quality	F. Community-based information system,Q. Knowledge management system

eNgoma Public Wi-Fi Chilufya Musosha, chilufya@e-ngoma.com			Yes				Yes		1. Information, 8. Accountability	D. Client communications system
Implementation of a Performance-Based Financing Data System for Zimbabwe - using the open source tool Hesabu Benson Muzenda, Benson.Muzenda@cordaid.org			Yes				Yes		1. Information, 3. Quality	M. Health finance and insurance information system
Interactive SMS for VMMC Post-surgical follow up Caryl Feldacker, cfeld@uw.edu			Yes			Yes			5. Utilization	D. Client communication system
Impilo EHR Dr. Robert Gongora, rgongora536@gmail.com		Yes					Yes		1. Information, 6. Efficiency,	H. Electronic medical record
COVID-19 Hub for Zimbabwe Kumbirai Matingo, matingonk@gmail.com	Yes								1. Information	C. Client applications ,P. Identification registries and directories,
Telehealth		Yes					Yes		2. Availability	Y. Telemedicine
Free emergency numbers	Yes	Yes					Yes		2.Availability, 5.Utilization, 7. Cost	I. Emergency response system
Health call centres	Yes	Yes					Yes		2.Availability, 5.Utilization, 7. Cost	I. Emergency response system, Y. Telemedicine
Mobile Health	Yes	Yes				Yes			2.Availability, 5.Utilization, 7. Cost	Y. Telemedicine
Community Mobilization		Yes	Yes				Yes		1.Information	D. Client communications system
Access to information databases and tools		Yes			Yes				1.Information, 3.Quality	S. Learning and training system
Decision support system		Yes				Yes			3. Quality	Q. Knowledge management system

Supply chain information system			Yes					Yes	1. Information, 2. Availability, 6. Efficiency 8. Accountability	T. Logistics management information system
Telehealth		Yes					Yes		2. Availability , 7.Cost	Y. Telemedicine
Telehealth		Yes				Yes			2. Availability 7. Cost	Y. Telemedicine
Laboratory information system		Yes						Yes	6. Efficiency	R. Laboratory and diagnostic information system
Phamarcy information system		Yes						Yes	6. Efficiency	U. Pharmacy information system
Electronic medical billing system		Yes						Yes	6. Efficiency, 8. Accountability	M. Health finance and insurance information system
Patient records		Yes						Yes	1. Information,6. Efficiency	H. Electronic medical record
mLearning		Yes						Yes	2. Availability, 3. Quality	S. Learning and training system
Decision support system		Yes						Yes	3. Quality	Q. Knowledge management system
Free emergency numbers	Yes	Yes					Yes		2.Availability, 5.Utilization	I. Emergency response system
Health call centres	Yes	Yes					Yes		2.Availability, 5.Utilization	I. Emergency response system, Y. Telemedicine
Appointment reminders		Yes				Yes			5. Utilization	D. Client communications system
Mobile telehealth	Yes	Yes			Yes				2. Availability, 7. Cost	Y. Telemedicine
Disaster management			Yes			Yes			2. Availability, 6. Efficiency	I. Emergency response system
Treatment adherence	Yes	Yes				Yes			5. Utilization	C. Client application
Community mobilization			Yes		Yes				1. Information	D. Client communications system
Access to information databases and tools		Yes					Yes		1. Information, 3. Quality	Q. Knowledge management system, S. Learning and training system

Patient monitoring		Yes				Yes			2. Availability, 5. Utilization ,7. Cost	Y. Telemedicine
Laboratory information system/PACS		Yes						Yes	6. Efficiency	R. Laboratory and diagnostic information system
Pharmarcy information system		Yes						Yes	6. Efficiency	U. Pharmacy information system
Automatic vaccine alert system		Yes						Yes	5. Utilization	D. Client communications system
Supply chain information system		Yes	Yes					Yes	1. Information, 2. Availability, 6. Efficiency, 8. Accountability	T. Logistics management information system
e-Learning platform		Yes						Yes	2. Availability, 3. Quality	S. Learning and training system
Free emergency numbers	Yes	Yes					Yes		2.Availability, 5.Utilization	I. Emergency response system
Health call centres	Yes	Yes					Yes		2.Availability, 5.Utilization	I. Emergency response system, Y. Telemedicine
Appointment reminders		Yes					Yes		5. Utilization	D. Client communications system
mHealth		Yes					Yes		2. Availability, 7. Cost	Y. Telemedicine
Community Mobilization			Yes				Yes		1. Information	D. Client communications system
Access to information databases and tools		Yes					Yes		1. Information, 3. Quality	Q. Knowledge management system, S. Learning and training system
Patient records		Yes					Yes		1. Information, 6. Efficiency	H. Electronic medical record
m-Learning		Yes					Yes		2. Availability, 3. Quality	S. Learning and training system
Decision support system		Yes					Yes		3. Quality	Q. Knowledge management system
Disaster management			Yes			Yes			2. Availability	I. Emergency response system
Telehealth		Yes				Yes			2. Availability, 7. Cost	Y. Telemedicine

Electronic billing system		Yes						Yes	6. Efficiency, 8. Accountability	M. Health finance and insurance information system
Supply chain information system		Yes	Yes					Yes	1. Information,2. Availability, 6. Efficiency, 8. Accountability	T. Logistics management information system
Toll free emergency numbers	Yes	Yes					Yes		2. Availability,5. Utilization	I. Emergency response system
Health call centres	Yes	Yes					Yes		2. Availability, 5. Utilization, 7.Cost	I. Emergency response system, Y. Telemedicine
Appointment reminders		Yes					Yes		5. Utilization	D. Client communications system
Disaster management			Yes				Yes		6. Efficiency	I. Emergency response system
Treatment adherence	Yes	Yes				Yes			5. Utilization	C. Client application
Access to information databases and tools		Yes					Yes		1. Information,2. Availability, 3. Quality	Q. Knowledge management system, S. Learning and training system
mLearning		Yes			Yes				2. Availability, 3. Quality	S. Learning and training system
Patient monitoring		Yes			Yes				5. Utilization, 7. Cost	Y. Telemedicine
Disaster management			Yes			Yes			2. Availability, 7. Cost	I. Emergency response system
Telehealth		Yes				Yes			2. Availability, 7. Cost	Y. Telemedicine
Electronic billing system		Yes						Yes	6. Efficiency, 8. Accountability	M. Health finance and insurance information system
Supply chain information system		Yes	Yes					Yes	1. Information,2. Availability, 6. Efficiency, 8. Accountability	T. Logistics management information system
Toll free emergency numbers	Yes	Yes					Yes		2. Availability,5. Utilization	I. Emergency response system
Health call centres	Yes	Yes					Yes		2. Availability, 5. Utilization, 7.Cost	I. Emergency response system, Y. Telemedicine
Appointment reminders		Yes					Yes		5. Utilization	D. Client communications system

Disaster management			Yes				Yes		6. Efficiency	I. Emergency response system
Treatment adherence	Yes	Yes				Yes			5. Utilization	C. Client application
Access to information databases and tools		Yes					Yes		1. Information,2. Availability, 3. Quality	Q. Knowledge management system, S. Learning and training system
mLearning		Yes			Yes				2. Availability, 3. Quality	S. Learning and training system
Patient monitoring		Yes			Yes				2. Availability, 5. Utilization, 7.Cost	Y. Telemedicine
Telehealth		Yes					Yes		2. Availability, 7. Cost	Y. Telemedicine
Telehealth		Yes			Yes				2. Availability, 7. Cost	Y. Telemedicine
Electronic Health record		Yes						Yes	1. Information, 6. Efficiency	H. Electronic medical record
Laboratory information system		Yes						Yes	6. Efficiency	R. Laboratory and diagnostic information system
Pharmarcy information system		Yes						Yes	6. Efficiency	U. Pharmacy information system
Electronic medical billing system		Yes						Yes	6. Efficiency, 8. Accountability	M. Health finance and insurance information system
Supply chain information system		Yes	Yes					Yes	1. Information, 2. Availability, 6. Efficiency,8. Accountability	T. Logistics management information system
e-Learning platform		Yes						Yes	2 Availability, 3. Quality	S. Learning and training system
Toll free emergency numbers	Yes	Yes					Yes		2. Availability	I. Emergency response system
Health call centres	Yes	Yes					Yes		2. Availability	I. Emergency response system, Y. Telemedicine
Appointment reminders		Yes				Yes			5. Utilization	D. Client communications system
Mobile telehealth	Yes	Yes				Yes			2. Availability, 7. Cost	Y. Telemedicine
Disaster management			Yes				Yes		6. Efficiency	I. Emergency response system

Treatment adherence	Yes	Yes				Yes			5. Utilization	C. Client application
Access to information databases and tools		Yes				Yes			1. Information, 2. Availability, 3. Quality	Q. Knowledge management system, S. Learning and training system
m-Learning		Yes					Yes		2. Availability, 3. Quality	S. Learning and training system
Telehealth		Yes					Yes		2.Availability, 7. Cost	Y. Telemedicine
Telehealth		Yes						Yes	2. Availability, 7. Cost	Y. Telemedicine
Supply chain information system		Yes	Yes					Yes	1. Information, 2, Availability,6. Efficiency, 8. Accountability	T. Logistics management information system
e-Learning platform		Yes						Yes	2. Availability, 3. Quality	S. Learning and training system
Toll free emergency	Yes	Yes					Yes		2. Availability	I. Emergency response system
Appointment reminders		Yes						Yes	5. Utilization	D. Client communications system
Disaster management			Yes		Yes				6. Efficiency	I. Emergency response system
Community mobilization			Yes					Yes	1. Information	D. Client communications system
Electronic billing system		Yes						Yes	6. Efficiency, 8. Accountability	M. Health finance and insurance information system
Supply chain information system		Yes	Yes					Yes	1. Information, 2. Availability, 6. Efficiency, 8. Accountability	T. Logistics management information system
e-Learning platform		Yes						Yes	2. Availability, 3. Quality	S. Learning and training system
Toll free Emergency	Yes	Yes					Yes		2. Availability	I. Emergency response system
Call centres and numbers	Yes	Yes					Yes		2.Availability	I. Emergency response system
Disaster management			Yes			Yes			6. Efficiency	I. Emergency response system, Y. Telemedicine
Community mobilization			Yes				Yes		1. Information	D. Client communications system

Access to information databases and tools		Yes					Yes		1. Information, 2. Availability, 3. Quality	Q. Knowledge management system, S. Learning and training system
Patient records		Yes			Yes	Yes			1. Information, 6. Efficiency	H. Electronic medical record
m-Learning		Yes				Yes	Yes		2. Availability, 3. Quality	S. Learning and training system
Patient monitoring	Yes	Yes					Yes		6. Utilization	Y. Telemedicine
Telehealth		Yes			Yes			Yes	2. Availability, 7. Cost	Y. Telemedicine
Supply chain information system		Yes	Yes					Yes	1. Information,2. Availability, 6. Efficiency, 8. Accountability	T. Logistics management information system
Laboratory information system		Yes						Yes	6. Efficiency	R. Laboratory and diagnostic information system
Pharmarcy information system		Yes						Yes	6. Efficiency	U. Pharmacy information system
Electronic billing system		Yes						Yes	6. Efficiency, 8. Accountability	M. Health finance and insurance information system
Supply chain information system		Yes	Yes					Yes	1. Information,2. Availability, 6. Efficiency, 8. Accountability	T. Logistics management information system
Telehealth		Yes				Yes			2. Availability, 7. Cost	Y. Telemedicine
e-Learning platform		Yes				Yes			2. Availability, 3. Quality	S. Learning and training system
Appointment reminders		Yes				Yes			5. Utilization	D. Client communications system
Mobile telehealth		Yes				Yes			2. Availability, 7. Cost	Y. Telemedicine
Disaster management			Yes			Yes			6. Efficiency	I. Emergency response system
Treatment adherence	Yes	Yes				Yes			5. Utilization	C. Client application
Community mobilization		Yes				Yes			1. Information	D. Client communications system
Patient records		Yes				Yes			1. Information, 6. Efficiency	H. Electronic medical record

m-Learning		Yes				Yes			2. Availability, 3. Quality	S. Learning and training system
Decision support system		Yes				Yes			3. Quality	Q. Knowledge management system
Telehealth		Yes					Yes		2. Availability, 7. Cost	Y. Telemedicine
Electronic billing system		Yes						Yes	6. Efficiency, 9. Accountability	M. Health finance and insurance information system
Supply chain information system		Yes	Yes					Yes	1. Information, 2. Availability, 6. Efficiency, 8. Accountability	T. Logistics management information system
e-Learning platform		Yes						Yes	2 Availability, 3. Quality	S. Learning and training system
Toll free emergency numbers	Yes	Yes					Yes		2. Availability	I. Emergency response system
Health call centres	Yes	Yes					Yes		2. Availability	I. Emergency response system, Y. Telemedicine
Appointment reminders		Yes				Yes			5. Utilization	D. Client communications system
Mobile telehealth	Yes	Yes					Yes		2. Availability, 7. Cost	Y. Telemedicine
Disaster management			Yes			Yes			6. Efficiency	I. Emergency response system
Community mobilization			Yes		Yes				1. Information	D. Client communications system
Access to information databases and tools		Yes			Yes				1. Information, 3. Quality	Q. Knowledge management system, S. learning and training system
Patient records		Yes					Yes		1. Information, 6. Efficiency	H. Electronic medical records
m-Learning		Yes			Yes				3. Quality	S. Learning and training system
Decision support system			Yes				Yes		1. Information	Q. Knowledge management system
Telehealth		Yes				Yes			2. Availability, 7. Cost	Y. Telemedicine
Electronic billing system		Yes						Yes	6. Efficiency, 8. Accountability	M. Health finance and insurance information system

Supply chain information system		Yes	Yes					Yes	1. Information, 2. Availability, 6. Efficiency, 8. Accountability	T. Logistics management information system
e-Learning platform		Yes						Yes	2. Availability, 3. Quality	S. Learning and training system
Toll free emergency	Yes	Yes			Yes				2. Availability	I. Emergency response system
Call centres and numbers	Yes	Yes				Yes			2. Availability	I. Emergency response system, Y. Telemedicine
Appointment reminders		Yes			Yes				5. Utilization	D. Client communications system
Mobile telehealth		Yes				Yes			2. Availability, 7. Cost	Y. Telemedicine
Disaster management			Yes			Yes			6. Efficiency	I. Emergency response system
Treatment adherence	Yes	Yes				Yes			5. Utilization	C. Client application
Community mobilization			Yes		Yes				1. Information	D. Client communications system
Access to information databases and tools		Yes				Yes			1. Information,2. Availability, 3. Quality	Q. Knowledge management system, S. learning and training system
Patient records		Yes				Yes			1. Information, 6. Efficiency	H. Electronic medical records
m-Learning		Yes				Yes			2. Availability, 3. Quality	S. Learning and training system
Decision support system		Yes				Yes			3. Quality	Q. Knowledge management system
Telehealth		Yes				Yes			2. Availability, 7. Cost	Y. Telemedicine
Electronic billing system		Yes						Yes	6. Efficiency, 8. Accountability	M. Health finance and insurance information system
Supply chain information system		Yes	Yes					Yes	1. Information,2. Availability, 6. Efficiency, 8. Accountability	T. Logistics management information system
e-Learning platform		Yes						Yes	2. Availability, 3. Quality	S. Learning and training system
Toll free emergency	Yes	Yes				Yes			2. Availability	I. Emergency response system

Mobile telehealth	Yes	Yes				Yes			2. Availability, 7. Cost	Y. Telemedicine
Disaster management			Yes		Yes				6. Efficiency	I. Emergency response system
Community mobilization			Yes		Yes				1. Information	D. Client communications system
Access to information databases and tools		Yes				Yes			1. Information,3. Quality	Q. Knowledge management system, S. learning and training system
Patient monitoring		Yes			Yes				5. Utilization, 7. Cost	Y. Telemedicine
Telehealth		Yes			Yes				2. Availability, 7. Cost	Y. Telemedicine
Electronic billing system		Yes						Yes	6. Efficiency, 8. Accountability	M. Health finance and insurance information system
Supply chain information system		Yes	Yes					Yes	1. Information, 2 Availability, 6. Efficiency, 8. Accountability	T. Logistics management information system
e-Learning platform		Yes						Yes	2. Availability, 3. Quality	S. Learning and training system
Toll-free emergency number	Yes	Yes					Yes		2. Availability	I. Emergency response system
Call centres and numbers	Yes	Yes					Yes		2. Availability	I. Emergency response system, Y. Telemedicine
Appointment reminders		Yes			Yes				5. Utilization	D. Client communications system
Mobile telehealth	Yes	Yes			Yes				2. Availability, 7. Cost	Y. Telemedicine
Disaster management			Yes				Yes		6. Efficiency	I. Emergency response system
Treatment adherence	Yes	Yes			Yes				5. Utilization	C. Client application
Community mobilization			Yes		Yes				1. Information	D. Client communications system
Access to information databases and tools	Yes	Yes			Yes				1. Information, 2. Availability, 3. Quality	Q. Knowledge management system, S. learning and training system
m-Learning		Yes					Yes		2. Availability, 3. Quality	S. Learning and training system
Decision support system		Yes			Yes				3. Quality	Q. Knowledge management system

Patient monitoring		Yes			Yes				1. Information, 3. Quality, 7. Cost	Y. Telemedicine
Telehealth		Yes					Yes		2. Availability, 7. Cost	Y. Telemedicine
Telehealth		Yes				Yes			2. Availability, 7. Cost	Y. Telemedicine
e-Learning platform		Yes						Yes	3. Quality	S. Learning and training system
Toll-free emergency number	Yes	Yes					Yes		2. Availability	I. Emergency response system
Health call centres		Yes					Yes		2. Availability	I. Emergency response system, Y. Telemedicine
Appointment reminders		Yes					Yes		5. Utilization	D. Client communications system
Mobile telehealth		Yes					Yes		2. Availability, 7. Cost	Y. Telemedicine
Disaster management			Yes				Yes		6. Efficiency	I. Emergency response system
Community Mobilization			Yes				Yes		1. Information	D. Client communications system
Access to information databases and tools		Yes					Yes		1. Information,2. Availability, 3. Quality	Q. Knowledge management system, S. learning and training system
Patient records		Yes					Yes		1. Information, 6. Efficiency	H. Electronic medical records
Decision support system		Yes					Yes		3. Quality	Q. Knowledge management system
Patient monitoring		Yes					Yes		5. Utilization, 7. Cost	Y. Telemedicine
Telehealth		Yes					Yes		2. Availability, 7. Cost	Y. Telemedicine
Telehealth		Yes				Yes			2. Availability, 7. Cost	Y. Telemedicine
Electronic billing system		Yes						Yes	6. Efficiency, 8. Accountability	M. Health finance and insurance information system
Supply chain information system		Yes	Yes					Yes	1. Information, 2. Availability, 6. Efficiency, 8. Accountability	T. Logistics management information system
e-Learning platform		Yes						Yes	2. Availability, 3. Quality	S. Learning and training system

Toll free emegency	Yes	Yes				Yes			2. Availability	I. Emergency response system
Call centres and numbers	Yes	Yes					Yes		2. Availability	I. Emergency response system, Y. Telemedicine
Appointment reminders		Yes				Yes			5. Utilization	D. Client communications system
Mobile telehealth	Yes	Yes			Yes	Yes			2. Availability, 7. Cost	Y. Telemedicine
Disaster management			Yes				Yes		6. Efficiency	I. Emergency response system
Treatment adherence	Yes	Yes				Yes			5. Utilization	D. Client communications system
Community mobilzation			Yes				Yes		1. Information	D. Client communications system
Access to information databases and tools		Yes				Yes	Yes		1. Information, 2. Availability,3. Quality	Q. Knowledge management system, S. learning and training system
Patient records		Yes				Yes	Yes		1. Information, 6. Efficiency	H. Electronic medical records
m-Learning		Yes					Yes		2. Availability,	S. Learning and training system
Decision support system		Yes				Yes	Yes		3. Quality	Q. Knowledge management system
Patient monitoring		Yes				Yes			5. Utilization, 7. Cost	Y. Telemedicine
Health call centres		Yes					Yes		2. Availability, 7. Cost	Y. Telemedicine
Appointment reminders		Yes				Yes			5. Utilization	D. Client communications system
Mobile telehealth	Yes	Yes				Yes			2. Availability, 7. Cost	Y. Telemedicine
Treatment adherence		Yes				Yes	Yes		5. Utilization	D. Client communications system
Community mobilization			Yes						1. Information	D. Client communications system
Access to information databases and tools		Yes				Yes			1. Information, 2. Availability, 3. Quality	Q. Knowledge management system, S. learning and training system
Patient records		Yes				Yes			1. Information, 6. Efficiency	H. Electronic medical records
Patient monitoring		Yes					Yes		5. Utilization, 7. Cost	Y. Telemedicine
Health call centres	Yes	Yes				Yes			2. Availability, 7. Cost	Y. Telemedicine

Community mobilization			Yes				Yes		1. Information	D. Client communications system
Telehealth		Yes				Yes			2. Availability, 7. Cost	Y. Telemedicine
Electronic billing system		Yes						Yes	6. Efficiency, 8. Accountability	M. Health finance and insurance information system
Supply chain information system		Yes	Yes					Yes	1. Information, 2. Availability, 6. Efficiency, 8. Accountability	T. Logistics management information system
e-Learning platform		Yes						Yes	2. Availability, 3. Quality	S. Learning and training system
Toll-free emergency number	Yes	Yes					Yes		2. Availability	I. Emergency response system
Health call centres	Yes	Yes					Yes		2. Availability	I. Emergency response system, Y. Telemedicine
Mobile telehealth	Yes	Yes					Yes		2. Availability, 7. Cost	Y. Telemedicine
Disaster management			Yes				Yes		6. Efficiency	I. Emergency response system
Community mobilization			Yes				Yes		1. Information	D. Client communications system
Decision support system		Yes					Yes		3. Quality	Q. Knowledge management system
Telehealth		Yes					Yes		2. Availability, 7. Cost	Y. Telemedicine
Telehealth		Yes				Yes			2. Availability, 7. Cost	Y. Telemedicine
Electronic billing system		Yes						Yes	6. Efficiency, 8. Accountability	M. Health finance and insurance information system
Supply chain information system		Yes	Yes					Yes	1. Information, 2. Availability, 6. Efficiency, 8. Accountability	T. Logistics management information system
e-Learning platform		Yes						Yes	2. Availability, 3. Quality	S. Learning and training system
Toll free emergency numbers	Yes	Yes				Yes			2. Availability	I. Emergency response system
Health call centres	Yes	Yes				Yes			2. Availability	I. Emergency response system, Y. Telemedicine

Appointment reminders		Yes				Yes			5. Utilization	D. Client communications system
Mobile telehealth	Yes	Yes			Yes				2. Availability, 7. Cost	Y. Telemedicine
Disaster management			Yes				Yes		6. Efficiency	I. Emergency response system
Treatment adherence	Yes	Yes				Yes			5. Utilization	D. Client communications system
Community mobilization			Yes			Yes			1. Information	D. Client communications system
Access to information databases and tools		Yes					Yes		1. Information, 2. Availability,3. Quality	Q. Knowledge management system, S. learning and training system
Patient records		Yes					Yes		1. Information, 6. Efficiency	H. Electronic medical records
mLearning		Yes					Yes		2.Availability, 3. Quality	S. Learning and training system
Decision support system		Yes					Yes		3. Quality	Q. Knowledge management system
Patient monitoring		Yes					Yes		5. Utilization, 7. Cost	Y. Telemedicine
Telehealth		Yes						Yes	2. Availability, 7. Cost	Y. Telemedicine
Supply chain information system		Yes	Yes					Yes	1. Information, 2. Availability,6. Efficiency,8. Accountability	T. Logistics management information system
e-Learning platform		Yes						Yes	2. Availability, 3. Quality	S. Learning and training system
Toll free emergency numbers	Yes	Yes						Yes	2. Availability	I. Emergency response system
Health call centres	Yes	Yes						Yes	2. Availability, 7. Cost	I. Emergency response system, Y. Telemedicine
Appointment reminders	Yes	Yes						Yes	5. Utilization	D. Client communications system
Treatment adherence	Yes	Yes						Yes	5. Utilization	D. Client communications system
Community mobilization			Yes					Yes	1. Information	D. Client communications system
Patient monitoring		Yes				Yes			5. Utilization, 7. Cost	Y. Telemedicine
m-Learning		Yes						Yes	2. Availability, 3. Quality	S. Learning and training system

Decision support system		Yes						Yes	3. Quality	Q. Knowledge management system
Telehealth		Yes						Yes	2. Availability, 7. Cost	Y. Telemedicine
Telehealth		Yes			Yes				2. Availability, 7. Cost	Y. Telemedicine
Telehealth		Yes					Yes		2. Availability, 7. Cost	Y. Telemedicine
Telehealth		Yes				Yes			2. Availability, 7.Cost	Y. Telemedicine
e-Learning platform		Yes						Yes	2. Availability, 3. Quality	S. Learning and training system
Toll free emergency	Yes	Yes						Yes	2. Availability	I. Emergency response system
Health call centres	Yes	Yes				Yes			2. Availability, 7. Cost	I. Emergency response system, Y. Telemedicine
Appointment reminders		Yes				Yes			5. Utilization	D. Client communications system
Mobile telehealth	Yes	Yes			Yes		Yes		2. Availability, 7. Cost	Y. Telemedicine
Disaster management			Yes		Yes		Yes		6. Efficiency	I. Emergency response system
Treatment adherence		Yes				Yes			5. Utilization	D. Client communications system
Community Mobilization			Yes			Yes			1. Information	D. Client communications system
Access to information databases and tools	Yes	Yes					Yes		1. Information, 2. Availability,3. Quality	Q. Knowledge management system, S. Learning and training system
Patient records		Yes			Yes				1. Information, 6. Efficiency	H. Electronic medical records
mLearning		Yes			Yes				2. Availability, 3. Quality	S. Learning and training system
Decision support system		Yes			Yes				3. Quality	Q. Knowledge management system
Patient monitoring			Yes				Yes		1. Information, 6. Efficiency, 7. Cost	Y. Telemedicine
m-health applications in Botswana: telemedicine and education, Littman-Quinn et al; 2013		Yes				Yes			2. Availability, 3. Quality, 7. Cost	S. Learning and training system, Y. Telemedicine

e-MANIC, Institut de Recherche en Sciences de la Sante, Burkina Faso,2020		Yes				Yes			3. Quality	R. Laboratory and diagnostics information system
Smart glasses for telemedicine, Diaka et al;2021		Yes				Yes			2. Availability, 5. Utilization, 7.Cost	Y. Telemedicine
Cloud-based peri-opertive registry,Network of perioperative critical care, 2020		Yes					Yes		2. Availability, 3. Quality, 5. Utilization, 6. Efficiency	K. Facility management information system,
mHealth Intervention on Delivery and Postnatal Care Utilization, Spigt et al; 2016		Yes				Yes			1. Information, 5. Utilization	D. Client communications system
USAID Digital Health Activity (DHA), https://www.jsi.com/project/digital-health-activity/			Yes				Yes		1. Information, 2. Availability, 3. Quality, 6. Efficiency	N. Health management information system, V. Public health and disease surveillance system
Cloud-based electronic data system, Jede et al;2020		Yes				Yes			1. Information , 6. Efficiency	H. Electronic medical record
‘virtual ward’ (VW) system, Wariri et al; 2021		Yes				Yes			2. Availability, 7. Cost	Y. Telemedicine
Empower Health software, Otieno et al;2020		Yes				Yes			2. Availability, 3. Quality, 6. Efficiency	Y. Telemedicine D. Client communications system
COVID-19 Tracker App, https://www.itu.int/hub/2020/05/ghana-launches-covid-19-tracker-app/			Yes				Yes		1. Information	N. Health management information system, V. Public health and disease surveillance system
mHealth to Train Community Health Nurses, Asgary et al; 2016		Yes				Yes			2. Availability, 3. Quality	S. Learning and training system
Telecytology in East Africa, Kumar et al;2012		Yes				Yes			2. Availability, 3. Quality	R. Laboratory and diagnostics information system

Real-Time Monitoring and Evaluation of a Visual-Based Cervical Cancer Screening Program Using a Decision Support Job Aid, Peterson et al 2016		Yes				Yes			1. Information, 3. Quality	H. Electronic medical record , Q. Knowledge management system
The Millennium Villages Project (MVP), mobile Health tool, Mushamiri et al; 2015		Yes				Yes			1. Information, 5. Utilization	D. Client communications system
Afya data, Karumuribo et al; 2017		Yes				Yes			1. Information, 6. Efficiency	V. Public Health and disease surveillance system
Connected Diagnostics, van Duijn; 2021		Yes				Yes			2. Availability,3. Quality 6. Efficiency	R. Laboratory and diagnostics information system
Empower Health software, Otieno et al;2020		Yes				Yes			2. Availability, 3. Quality, 6. Efficiency	Y. Telemedicine D. Client communications system
i-PUSH, Abajobir et al; 2021	Yes					Yes			7.Cost	C. Client application, M. Health finance and insurance information system
Inuka Coaching, Doukani et al 2021		Yes				Yes			2. Availability	Q. Knowledge management system
PANDA (pregnancy and newborn diagnosis assessment) system, Garcia Vilaplana et al;2020		Yes				Yes			1. Information, 2. Availability, 5. Utilization, 6. Efficiency, 7. Cost	H. Electronic medical record
The Mobile Maternal Health Wallet (MMHW), Lacroze et al; 2021	Yes					Yes			7. Cost	M. Health finance and insurance information system
Smartphone Use for Cervical Cancer Screening in Low-Resource Countries, Catarino et al; 2015		Yes				Yes			3. Quality	R. Laboratory and diagnostics information system

A Smartphone-Based Approach for Triage of Human Papillomavirus, Urner et al; 2017		Yes				Yes			2. Availability	R. Laboratory and diagnostics information system
Smartphone digital images for the detection of cervical intraepithelial neoplasia of grade 2 or worse (CIN2+), Ricard-Gauthier et al; 2015		Yes				Yes			2. Availability	R. Laboratory and diagnostics information system
NeoTree digital capture and quality improvement system Mgusha et al 2021		Yes				Yes			1. Information, 3. Quality , 6. Efficiency	H. Electronic medical record
StAR2D, Farmer et al 2021		Yes				Yes			1. Information, 5. Utilization	D. Client communications system
Computer-assisted digital chest X-ray technology, Twabi et al;2021		Yes					Yes		2. Availability, 3. Quality, 7. Cost	R. Laboratory and diagnostics information system
Mobile Phone Text Message Reminders, Steinhardt et al; 2018		Yes				Yes			1.Information	Q. Knowledge management system
Accurate Real-Time Diagnosis of Lymphoproliferative Disorders in Malawi Through Clinicopathologic Teleconferences, Montgomery et al; 2016		Yes				Yes			2. Availability	R. Laboratory and diagnostics information system, Y. Telemedicine
Tablet-Based Cervical Cancer Educational Intervention, Caster et al; 2017	Yes					Yes			1. Information	C. Client application
Wellvis COVID triage tool, Owoyemi et al;2021	Yes						Yes		1. Information, 2. Availability,6. Efficiency, 7.Cost , 8. Accountability	C. Client application, V. Public health and disease surveillance system

EXTEND evaluation, Ebenso et al 2018		Yes					Yes		1. Information,2. Availability	N. Health management information system, S. Learning and training system
Remote Teaching of Histopathology, Rotimi et al; 2017		Yes				Yes			2. Availability, 3. Quality	S. Learning and training system
Mobile phone short message service, Omole et al; 2018		Yes				Yes			1. Information, 5.Utilization	D. Client communications system
Neural Transfer Learning for Cry-based Diagnosis of Perinatal Asphyxia, Onu et al;2019		Yes				Yes			3. Quality	R. Laboratory and diagnostics information system
PEERNaija, Ahonkhai et al; 2021	Yes					Yes			1. Information, 5. Utilization	C. Client application
Mobile digital quiz game for the promotion of malaria awareness,Chikwuocha et al; 2020		Yes	Yes			Yes			1. Information, 2. Availability	C. Client application
Clinical decision support algorithm, Center for Primary Care and Public Health (Unisante), University of Lausanne, Switzerland, 2022		Yes				Yes			3. Quality	Q. Knowledge management system
RapidSMS-MCH, Ngabo et al; 2012		Yes				Yes			1. Information, 2. Availability, 5. Utilization, 6. Efficiency	D. Client communications system, I. Emergency response system
Telepathology Triage, Mpunga et al; 2016		Yes				Yes			2. Availability	R. Laboratory and diagnostics information system
Smartphone Application for Detecting Skin Cancers, Rubagumya et al; 2020		Yes				Yes			2. Availability	R. Laboratory and diagnostics information system

Uber for Blood, McVeigh, K. 2018. “'Uber for Blood': How rwandan Delivery robots Are Saving lives.” The Guardian		Yes				Yes			2. Availability, 6. Efficiency	T. Logistics management information system
Aviro Health App, https://borgenproject.org/aviro-health-in-south-africa/	Yes	Yes					Yes		1. Information, 2. Availability, 3. Quality	C. Client application, S.Learning and training system, Y. Telemedicine
Tele-reading, Griggs et al ;2014		Yes				Yes			2. Availability , 7.Cost	Y. Telemedicine
TBDx, Applied Visual Systems, Inc, Swati et al;2016		Yes				Yes			2. Availability, 3. Quality, 7. Cost	R. Laboratory and diagnostics information system
Mobile Health (mHealth) technologies for epilepsy, Davies et al 2021		Yes				Yes			2. Availability, 7. Cost	Y. Telemedicine
d-CXR, Moodley et al 2021		Yes				Yes			2. Availability, 6. Efficiency, 7. Cost	S. Learning and training system
momConnect, Pillay and Motsoaledi,2018		Yes					Yes		1. Information, 5. Utilization	S. Learning and training system
StAR2D, Farmer et al 2021		Yes				Yes			1. Information, 5. Utilization	S. Learning and training system
The Philani MOVIE, Adam et al 2021		Yes				Yes			1. Information	S. Learning and training system
Home-based intervention to test and start (HITS), Mathenjwa et al 2019	Yes					Yes			5. Utilization	C. Client application , Q. Knowledge management system
ProLife Moriarty et al; 2019		Yes				Yes			1. Information, 5. Utilization	D. Client communications system
Mobile tracking of those with coronavirus, https://www.reuters.com/article/health-coronavirus-telkom-sa/s-africa-launches-mobile-tracking-of-those-with-coronavirus-idUSL8N2BQ61L			Yes				Yes		1. Information	N. Health management information system, V. Public health and disease surveillance system

Electronic booking system, Booyse et al; 2020		Yes				Yes			1. Information, 6. Efficiency	H. Electronic medical records
The Kilimanjaro Method, Yeates et al; 2016		Yes				Yes			3. Quality	S. Learning and training system
The CAD4TB software is a computer-aided detection system		Yes				Yes			2. Availability, 3. Quality, 6. Efficiency	R. Laboratory and diagnostics information system
Mobile phone Communication, Lund et al; 2012		Yes				Yes			1. Information , 5. Utilization	D. Client communications system
InfoSpots, Holst et al; 2021	Yes					Yes			1. Information	C. Client application
Clinical decision support algorithm, Center for Primary Care and Public Health (Unisante), University of Lausanne, Switzerland, 2022		Yes				Yes			3. Quality	Q. Knowledge management system
SMS text messaging app, Siedner et al; 2015		Yes				Yes			1. Information, 5. Utilization	R. Laboratory and diagnostics information system
Low-cost ruggedized Android tablets to augment in-service training of community health worker Stiles et al;2021		Yes				Yes			2. Availability, 3. Quality	Q. Knowledge management system
Mobile Phone Consultations, Ayiasi et al 2016		Yes				Yes			1. Information, 5. Utilization , 7. Cost	Y. Telemedicine
mTrac UNICEF Uganda, 2011		Yes					Yes		1. Information, 2. Availability,	T. Logistics management information system
mHealth application to strengthening pharmaceutical systems, Namisango et al; 2016		Yes				Yes			1. Information, 6. Efficiency	U. Pharmacy information system

Novel Mobile Phone Application to Improve Palliative Home-Care, Harding et al; 2021	Yes	Yes				Yes			2. Availability, 5. Utilization	C. Client application
Smartphone strategies for cervical cancer screening, Asgary et al;2020		Yes				Yes			2. Availability, 3. Quality	S. Learning and training system, Y. Telemedicine
Retinopathy AI screening, Bellemo et.al 2019		Yes				Yes			2. Availability, 3. Quality,	R. Laboratory and diagnostics information system
HealthTrax, Hong et al; 2015			Yes			Yes			1. Information, 5. Utilization	L. Geographic information system
Automatic software (CAD4TB 5) in chest X-ray (CXR), Melendez et al; 2017		Yes				Yes			2. Availability, 3. Quality, 6. Efficiency	R. Laboratory and diagnostics information system
Interactive digital tablet-based counselling, Thomas et al;2019	Yes					Yes			1. Information, 2. Availability, 3. Quality, 5. Utilization	C. Client application , Q. Knowledge management system
Novel Mobile Phone Application to Improve Palliative Home-Care, Harding et al; 2021	Yes	Yes				Yes			2. Availability, 5. Utilization	C. Client application

Table S4: Supplementary material: Digital Health Interventions used to strengthen health workforce in sub-Saharan Africa

Name/Reference/ Pointt of contact	Target -User				Stage of development				Addressed Health System Challenge	Aligned Systems Category
	Clients	Providers	Managers	Data Services	Informal	Pilot	Established	Unknown		
Kassai Anya Fedorova, Anya.Fedorova@psiangola.org		Yes					Yes		1. Information, 3. Qality	S. Learning and training system
The Safe Delivery App in Benin Lauren Bellhouse, lauren@maternity.dk		Yes					Yes		2. Avalability, 3. Quality	S. Learning and training system, Q. Knowledge management system,
OppiaMobile Alex Little, consult@alexlittle.net		Yes				Yes			3. Quality	S. Learning and training system
OPAC (Koha software open public access cataloge) Federal Ministry of Health, dha@moh.gov.et		Yes					Yes		1. Information	P. Identification registries and directories
National residency matching Federal Ministry of Health, dha@moh.gov.et		Yes					Yes		2. Availability, 3. Quality	S. Leaning and training systems
mHealth Federal Ministry of Health, dha@moh.gov.et		Yes					Yes		1. Information, 3. Quality	S. Learning and training system
HRIS Licensing Federal Ministry of Health, dha@moh.gov.et			Yes				Yes		2. Availability	O. Human resources information system
Green Stone Digital library Federal Ministry of Health, dha@moh.gov.et		Yes					Yes		1. Information	Q. Knowledge management system
DSS - Self Assement Tool Federal Ministry of Health, dha@moh.gov.et		Yes					Yes		1. Information	W. Research information system

Hello Nurse Darlene Irby, Darlene.Irby@jhpiego.org		Yes				Yes			3. Quality	S. Learning and training system
The Safe Delivery App in Ghana Lauren Bellhouse, lauren@maternity.dk		Yes					Yes		2. Availability, 3. Quality	Q. Knowledge management system, S. Learning and training system
eSIP-Saude Devan Manharlal, devan.manharlal@jhpiego.org			Yes			Yes			1. Information,6. Efficiency	O. Human resource information system
SIFIn Júlio Tualufo, julio.tualufo@jhpiego.org			Yes				Yes		1. Information,3. Quality, 6. Efficiency	O. Human resource information system, S. Learning and training system
Shugabanci Agbons Oaiya, bonkhi@gmail.com			Yes			Yes			2. Availability, 3. Quality	O. Human resource information system
NaijaCare Project Moshood Abdullateef, moshood@every1mobile.com		Yes				Yes			2. Availability,3. Quality	S. Learning and training system
HelpMum Dr Abiodun Adereni, abbey2real@gmail.com		Yes					Yes		1. Information, 2. Availability, 3. Quality	Q. Knowledge management system S. Learning and training system
Mobile Training and Support (MOTS) Frederick F. Kamara (World Vision-SL), Fredrick_kamara@wvi.org		Yes						Yes	2. Availability, 3. Quality,	S. Learning and training system
The Safe Delivery App in Sierra Leone Lauren Bellhouse, lauren@maternity.dk		Yes					Yes		2. Availability, 3. Quality	S. Learning and training system
MOTS COVID-19 Monica Amponsah, mamponsah@grameenfoundation.org		Yes						Yes	2. Availability, 3. Quality	S. Learning and training system

Improving health worker adherence to IPTp provision guidelines through sending text messages Christian Rassi, c.rassi@malariaconsortium.org		Yes				Yes			3. Quality	S. Learning and training system
Duty Roster and Attendance Tracking Mobile Application Dennis Kibiye, kibsden@gmail.com			Yes				Yes		1. Information, 2. Availability	O. Human resources information system
integrated Human Resource Information System (iHRIS) Ismail Wadembere, iwadembere@intrahealth.org			Yes					Yes	3. Quality	O. Human resource information system
Rheumatic Heart Disease and Hypertension Tracking Stefanie Kot, skot@d-tree.org		Yes						Yes	2. Availability, 5. Utilization	H. Electronic medical record, O. Human resources information system
Access to information databases and tools		Yes			Yes				1.Information, 3.Quality	S. Learning and training system
mLearning		Yes						Yes	2. Availability, 3. Quality	S. Learning and training system
Access to information databases and tools		Yes					Yes		1. Information, 3. Quality	Q. Knowledge management system, S. Learning and training system
e-Learning platform		Yes						Yes	2. Availability, 3. Quality	S. Learning and training system
Access to information databases and tools		Yes					Yes		1. Information, 3. Quality	Q. Knowledge management system, S. Learning and training system
m-Learning		Yes					Yes		2. Availability, 3. Quality	S. Learning and training system
Access to information databases and tools		Yes					Yes		1. Information,2. Availability, 3. Quality	Q. Knowledge management system, S. Learning and training system
mLearning		Yes			Yes				2. Availability, 3. Quality	S. Learning and training system

Access to information databases and tools		Yes					Yes		1. Information,2. Availability, 3. Quality	Q. Knowledge management system, S. Learning and training system
mLearning		Yes			Yes				2. Availability, 3. Quality	S. Learning and training system
e-Learning platform		Yes							2 Availability, 3. Quality	S. Learning and training system
Access to information databases and tools		Yes				Yes			1. Information, 2. Availability, 3. Quality	Q. Knowledge management system, S. Learning and training system
m-Learning		Yes					Yes		2. Availability, 3. Quality	S. Learning and training system
e-Learning platform		Yes						Yes	2. Availabilty, 3. Quality	S. Learning and training system
e-Learning platform		Yes						Yes	2. Availability, 3. Quality	S. Learning and training system
Access to information databases and tools		Yes					Yes		1. Information, 2. Availability, 3. Quality	Q. Knowledge management system, S. Learning and training system
m-Learning		Yes				Yes	Yes		2. Availability, 3. Quality	S. Learning and training system
e-Learning platform		Yes				Yes			2. Availability, 3. Quality	S. Learning and training system
m-Learning		Yes				Yes			2. Availability, 3. Quality	S. Learning and training system
Decision support system		Yes				Yes			3. Quality	Q. Knowledge management system
e-Learning platform		Yes						Yes	2 Availability, 3. Quality	S. Learning and training system
Access to information databases and tools		Yes			Yes				1. Information, 3. Quality	Q. Knowledge management system, S. learning and training system
m-Learning		Yes			Yes				3. Quality	S. Learning and training system
e-Learning platform		Yes						Yes	2. Availability, 3. Quality	S. Learning and training system
Access to information databases and tools		Yes				Yes	Yes		1. Information,2. Availability, 3. Quality	Q. Knowledge management system, S. learning and training system

m-Learning		Yes				Yes	Yes		2. Availability, 3. Quality	S. Learning and training system
e-Learning platform		Yes						Yes	2. Availability, 3. Quality	S. Learning and training system
Access to information databases and tools		Yes				Yes	Yes		1. Information,3. Quality	Q. Knowledge management system, S. learning and training system
e-Learning platform		Yes						Yes	2. Availability, 3. Quality	S. Learning and training system
Access to information databases and tools	Yes	Yes			Yes				1. Information, 2. Availability, 3. Quality	Q. Knowledge management system, S. learning and training system
m-Learning		Yes					Yes		2. Availability, 3. Quality	S. Learning and training system
e-Learning platform		Yes					Yes		3. Quality	S. Learning and training system
Access to information databases and tools		Yes					Yes		1. Information,2. Availability, 3. Quality	Q. Knowledge management system, S. learning and training system
e-Learning platform		Yes						Yes	2. Availability, 3. Quality	S. Learning and training system
Access to information databases and tools		Yes				Yes	Yes		1. Information, 2. Availability,3. Quality	Q. Knowledge management system, S.Learning and training system
m-Learning		Yes					Yes		2. Availability	S. Learning and training system
Access to information databases and tools		Yes				Yes			1. Information, 2. Availability, 3. Quality	Q. Knowledge management system, S.Learning and training system
e-Learning platform		Yes						Yes	2. Availability, 3. Quality	S. Learning and training system
e-Learning platform		Yes						Yes	2. Availability, 3. Quality	S. Learning and training system
Access to information databases and tools		Yes					Yes		1. Information, 2. Availability,3. Quality	Q. Knowledge management system, S. Learning and training system
e-Learning platform		Yes						Yes	2. Availability, 3. Quality	S. Learning and training system
m-Learning		Yes						Yes	2. Availability, 3. Quality	S. Learning and training system

e-Learning platform		Yes						Yes	2. Availability, 3. Quality	S. Learning and training system
Access to information databases and tools	Yes	Yes					Yes		1. Information, 2. Availability,3. Quality	Q. Knowledge management system, S. Learning and training system
mLearning		Yes			Yes				2. Availability, 3. Quality	S. Learning and training system
m-health applications in Botswana: telemedicine and education, Littman-Quinn et al; 2013		Yes				Yes	Yes		2. Availability, 3. Quality	S. Learning and training system, Y. Telemedicine
mHealth to Train Community Health Nurses, Asgary et al; 2016		Yes				Yes			2. Availability, 3. Quality	S. Learning and training system
EXTEND evaluation, Ebenso et al 2018		Yes					Yes		1. Information,2. Availability	N. Health management information system, S. Learning and training system
Remote Teaching of Histopathology, Rotimi et al; 2017		Yes				Yes	Yes		2. Availability, 3. Quality	S. Learning and training system
The Kilimanjaro Method, Yeates et al; 2016		Yes				Yes	Yes		3. Quality	S. Learning and training system
Low-cost ruggedized Android tablets to augment in-service training of community health worker Stiles et al;2021		Yes				Yes	Yes		2. Availability, 3. Quality	Q. Knowledge management system
Smartphone strategies for cervical cancer screening, Asgary et al;2020	Yes					Yes	Yes		2. Availability, 3. Quality	S. Learning and training system, Y. Telemedicine

Table S5: Supplementary material: Digital Health Interventions used to strengthen Health Information System in sub-Saharan Africa

**Name/Reference/Point of contact**	**Target - User**				**Stage of development**				**Addressed Health System Challenge**	**Aligned Systems Category**
	**Clients**	**Providers**	**Manages**	**Data Services**	**Informal**	**Pilot**	**Established**	**Unknown**		
IRS - Cunene Sergio Lopes, sergio@mentor-initiative.net			Yes			Yes			1. information, 3. Quality, 5. Utilization	F. Community -based information system, V. Public health and disease surveillance, W. Research information system
NTD LF TAS and Coverage Surveys Clara Burgert, cburgert@rti.org			Yes					Yes	1. Information	W. Research Information System
OpenLMIS Benin (SIIL) Rebecca Alban, info@openlmis.org		Yes	Yes				Yes		1. Information, 2. Availability, 6. Efficiency, 8. Accountability	T. Logistics management and information system (LMIS)
DHIS2 for COVID-19 Surveillance: Botswana Rebecca Potter, covid@dhis2.org			Yes				Yes		1. Information	V. Public Health and disease Sureillance System
Integrated e-Diagnostic Approach Thierry Agagliate, tag@tdh.ch		Yes					Yes		1. Information, 3.Quality	N. Health management information system, Q. Knowledge Management System,
One Health Platform Romain Tohouri, romain_tohouri@jsi.com				Yes			Yes		1. Information	N. Health management information system
DHIS2 for COVID-19 Surveillance: Burkina Faso Boukary, ouedbouks@gmail.com			Yes				Yes		1. Information	V. Public health and disease surveillance

Bio-fortified Value Chains for Improved Maternal and Child Nutrition (B4MCN) Bertin Nduwayo, bertin_nduwayo@wvi.org		Yes				Yes			1. Information, 2. Availability, 3. Quality, 6. Efficiency,	,F. Community-based Information System,V. Public health and disease surveillance, Y. Telemedicine
DHIS2 for COVID-19 Surveillance: Cabo Verde Rebecca Potter, covid@dhis2.org			Yes				Yes		1. Information	V. Public health and disease surveillance system
DHIS2 for COVID-19 Surveillance: Cameroun (in development) Rebecca Potter, covid@dhis2.org			Yes				Yes		1. Information	V. Public health and disease surveillance
Suivi de de la vaccination des enfants de moisn d'un an Sessouma Abdoulaye, sesabdnaz@yahoo.fr			Yes					Yes	1. Information	N. Health Managemen Information System
mHealth with ESPEN Collect REBOLLO POLO Maria, rebollopolom@who.int			Yes					Yes	1. Information	A.Census, population information & data warehouse, W. Research Information System
DHIS2 for COVID-19 Surveillance: Congo DRC (in development) Rebecca Potter, covid@dhis2.org			Yes				Yes		1. Information	V. Public health and disease surveillance
COVID-19 Vaccine Allocation App Anubhuti Mishra, anubhuti.mishra@thepalladiumgroup.com			Yes				Yes		1. Information, 2. Availability, 6. Efficiency,	F. Community-based Information System
DHIS2 for COVID-19 Surveillance: Djibouti Rebecca Potter, covid@dhis2.org			Yes				Yes		1. Information	V. Public health and disease surveillance

Hiwot: Addressing Maternal & Neonatal Health Stefanie Kot, skot@d-tree.org		Yes				Yes			1. information, 3. Quality, 6. Efficiency	F. Community-based Information System,Q. Knowledge Management,S. Learning and training system
VERS Federal Ministry of Health, dha@moh.gov.et			Yes				Yes		1. Information,	B. Civil Registration and Vital Statistics
Tena PIMS Federal Ministry of Health, dha@moh.gov.et		Yes					Yes		1. Information, 6. Efficiency,	H. Electronic medical record
SmartEMS (Samrt Electronic Medical System) Federal Ministry of Health, dha@moh.gov.et		Yes	Yes				Yes		1. Information, 6. Efficiency	H. Electronic medical record
RI (routine immunization dashboard) Federal Ministry of Health, dha@moh.gov.et			Yes				Yes		1. Information	N. Health management information system
PRS (Patient Registration System) Federal Ministry of Health, dha@moh.gov.et		Yes					Yes		1. Information, 6. Efficiency	H. Electronic medical record
PrimeCare electronic health record Federal Ministry of Health, dha@moh.gov.et		Yes					Yes		1. Information, 6. Efficiency	H. Electronic medical record
Patient Registration Software Federal Ministry of Health, dha@moh.gov.et		Yes					Yes		1. Information, 6. Efficiency	H. Electronic medical record
Open Clinic Federal Ministry of Health, dha@moh.gov.et		Yes					Yes		1. Information, 6. Efficiency	H. Electronic medical record, K. Facility management system
NHDD Federal Ministry of Health, dha@moh.gov.et			Yes				Yes		1. Information	N. Health Management information system
MRIS Federal Ministry of Health, dha@moh.gov.et			Yes				Yes		1. Information	F. Community-based Information System

HMIS Brisk Federal Ministry of Health, dha@moh.gov.et		Yes	Yes				Yes		1. Information, 6. Efficiency	H. Electronic medical record
eMRIS (Electronic Multi Sectoral Response Information System) Federal Ministry of Health, dha@moh.gov.et			Yes				Yes		1. Information	N. Health management information system
eHMIS - Regions except SNNP Region Federal Ministry of Health, dha@moh.gov.et			Yes				Yes		1. Information,	B. Civil Registration and Vital Statistics, N. Health management information system
eCHIS - Old Federal Ministry of Health, dha@moh.gov.et		Yes					Yes		1. Information	F. Community-based Information System
eCHIS - New Federal Ministry of Health, dha@moh.gov.et		Yes					Yes		1. information, 3. Quality, 6. Efficiency	F. Community based information system, Q. Knowledge management system,
DSS (demographic survellance system) Federal Ministry of Health, dha@moh.gov.et			Yes				Yes		1. Information	B. Civil registration and vital statistics
DHMS (Digital Hospital Management System) Federal Ministry of Health, dha@moh.gov.et		Yes	Yes				Yes		1. Information, 6. Efficicency	K. Facility management information system
District Health Information System (DHIS2) Federal Ministry of Health, dha@moh.gov.et			Yes				Yes		1. Information	N. Health management information system
DATIM (Data for Accountability Transparency & Impact Monitoring) Federal Ministry of Health, dha@moh.gov.et			Yes				Yes		1. Information	N. Health management information system
AGHMC EMR Federal Ministry of Health, dha@moh.gov.et		Yes					Yes		1. Information, 6. Efficiency	H. Electronic Medical Record

Ethiopia Health Data Analytics Platform (EHDAP) Dawit Kassa, dawit@zenysis.com				Yes			Yes		1. Information, 6. Efficiency,	G. Data interchange inteoperability and accessibiity
DHIS2 for COVID-19 Surveillance: Ethiopia Rebecca Potter, covid@dhis2.org			Yes				Yes		1. Information	V. Public health and disease surveillance
Life-saving Mobile Health Kits (LMHK) for Obstetric Care in Remote Areas Belay Haffa, belay_haffa@wvi.org		Yes				Yes			1. Information, 2. Availability, 5. Utilization, 6. Efficiency	F. Community-based Information System, Q. Knowledge Management Y. Telemedicine
Real-time COVID-19 case monitoring for the MoH Gambia Aleksa Krolls, aleksa@openfn.org			Yes			Yes			1. Information, 6. Efficiency	V. Public health and disease surveillance
DHIS2 for COVID-19 Surveillance: Gambia Rebecca Potter, covid@dhis2.org			Yes				Yes		1. Information	V. Public health and disease surveillance
MEDTRACK Omar Seidu Farouk, omar@medtrack.com.gh		Yes				Yes			1. Information, 6. Efficiency	H. Electronic health record, X. Shared Health record and health information repositories
Global Health Security Agenda Pia MacDonald, pmacdonald@rti.org			Yes					Yes	1. Information,	V. Public health and disease surveillance
Global Open Facility Registry (GOFR) Eileen Reynolds, ereynolds@rti.org				Yes			Yes		1. Information, 6. Efficiency	G. Data interchange, interoperability, and accessibility
Guinea EVD contact Tracing Anne Lui, Aboubacar DIALLO, Mamady Camara, adiallo84x@gmail.com			Yes					Yes	1. Information	F. Community-based Information System,,V. Public health and disease surveillance

DHIS2 for COVID-19 Surveillance: Guinea Rebecca Potter, covid@dhis2.org			Yes				Yes		1. Information	V. Public health and disease surveillance
DHIS2 for COVID-19 Surveillance: Guinea Bissau Rebecca Potter, covid@dhis2.org			Yes				Yes		1. Information	V. Public health and disease surveillance
Hoji Mobile Data Collection and Analysis Platform Gitahi Ng'ang'a, gitahi@hoji.co.ke			Yes				Yes		1. Information	W. Research Information System
Regional Action Through Data (RAD) Laurie Markle, LMarkle@brhc.com		Yes				Yes			1. Information, 5. Utilization, Efficiency	H. Electronic health record, X. Shared Health record and health information repositories
M-Sawazisha: Integrating Mobile Technology in MNCH in Afya Jijini Michael Korir, korir@medicmobile.org		Yes				Yes			1. Information, 2. Availability, 5. Utilization,	B. Civil Registration and Vital Statistics,F. Community-based Information System,
Bethany Kids Kijabe Hospital Steven Wanyee, swanyee@intellisoftkenya.com		Yes	Yes				Yes		1. Information, 6. Efficiency	H. Electronic medical record
COVID-19 SMS Data Collaboration Initiative Boris Maguire, boris@echomobile.org			Yes				Yes		1. Information	F. Community-based information system
DHIS2 for COVID-19 Surveillance: Kenya Rebecca Potter, covid@dhis2.org			Yes				Yes		1. Information	V. Public health and disease surveillance
KenyaEMR Pascal Mwele, Pascal.Mwele@thepalladiumgroup.com			Yes				Yes		1. Information, 6. Efficiency	H. Electronic medical record

National Data Warehouse (NDWH) Pascal Mwele, pascal.mwele@thepalladiumgroup.com				Yes			Yes		1. Information, 6. Efficiency	G. Data interchange, interoperability, and accessibility
Interoperability Layer Pascal Mwele, pascal.mwele@thepalladiumgroup.com				Yes			Yes		1. Information, 6. Efficiency	G. Data interchange inteoperability and accessibiity
DWAPI Pascal Mwele, pascal.mwele@thepalladiumgroup.com				Yes			Yes		1. Information, 6. Efficiency	G. Data interchange inteoperability and accessibiity
Afya Mobile Pascal Mwele, pascal.mwele@thepalladiumgroup.com		Yes					Yes		1. Information	F. Community-based information system
m-Jali Caroline Mbindyo, caroline.mbindyo@mref.org			Yes				Yes		1. Information	F. Community-based Information System, N. Health management information system
PaperEMR Pratap Kumar, pratap@health-e-net.org		Yes				Yes			1. Information, 6. Efficiency	H. Electronic Medical Record
DHIS2 MoH Data Warehouse Tsele Moloeli, moloelit@yahoo.com			Yes				Yes		1. Information	N. Health information management information system
Lesotho eRegister Monaheng Maoeng, monaheng.maoeng@gov.ls		Yes						Yes	1. Information, 6. Efficiency	H. Electrinic medical record, X. Shared Record and health information repositories
HIV and Health Situation Rooms Masebeo Koto, kotomasebeo@gmail.com			Yes					Yes	1. Information	N. Health management information system

CITIZEN APP Pule Phafane, pule@smartmycompany.com			Yes					Yes	1. Information,	I. Emergency response system , V. Public health and disease surveillance system , W. Research information system
DHIS2-Covid -19 surveillance -Lesotho Monaheng Maoeng, monaheng.maoeng@gov.ls			Yes				Yes		1. Information,	V. Public health and disease surveillance system
Bophelo-Ka-Mosebeletsi (Village Healthcare Workers) Application Teboho Khoali, teboho.khoali@undp.org		Yes				Yes			1. Information,	F. Community-based information system
EADEL - EGPAF Analytics Data Engine Lesotho Sele Mthimkhulu, smthimkhulu@pedaids.org		Yes	Yes				Yes		1. Information,	N. Health management information system
mHero in Liberia Intrahealth International, digitalhealth@intrahealth.org		Yes	Yes				Yes		1. Information	I. Emergencey response system, V. Public health and disease surveillance system
Liberia Health Analytics Platform (LHAP) Claire Cravero, claire@zenysis.com				Yes			Yes		1. Information, 6. Efficiency	G, Data interchange interoperability and accessibility
USAID ACCESS m-health initiative Stephanie Ranaivo, sranaivo@usaidaccess.org		Yes					Yes		1. Information, 3. Quality	F. Community-based Information System,N. Health Management Information System
Covid-19 DHIS 2 Rebecca Potter, covid@dhis2.org			Yes				Yes		1. Information	V. Public health and disease surveillance
UBALE (MIRA STUDY) Dane Fredenburg, dane.fredenburg@crs.org			Yes			Yes			1. Information, 8. Accountability	W. Research Information System

Medic at St Gabriel's Hospital Alex Ngalande, unknown@yahoo.com		Yes				Yes			1, Information	B. Civil Registration and Vital Statistics
ONSE - Integrated Supportive Supervision Toolkit Rudi Thetard - ONSE Chief of Party, rthetard@msh.org			Yes			Yes			1. Information, 3.Quality	N. Health management information system
ARGUS Joseph Wu, wcsg@lukeinternational.no			Yes			Yes			1. Information,	V. Public health and disease surveillance
Malawi electronic integrated disease surveillance and response system (Malawi eIDSR) Joseph Wu, wcsg@lukeinternational.no		Yes	Yes				Yes		1. Information, 6. Efficiency	H. Electronic medical records, V. Public health and disease surveillance system
One Community Steve Choko, stevec@jhuccpmw.org		Yes				Yes			1. Information	F. Community based information system
eIDSR Case Based Surveillance App Malawi Soyapi Mumba, soyapim@gmail.com		Yes	Yes				Yes		1. Information	V. Public health and disease surveillance system
Barr Foundation iCCM mHealth project Erica Layer, elayer@d-tree.org			Yes			Yes			1. Information	F. Community based health informatin system, Q. Knowledge management system
Medic Mobile DIY Malawi Jacqueline Edwards, jacqueline@medicmobile.org		Yes						Yes	1. Information, 6. Efficiency	H. Electronic medical record
Malawi German Health Programme (MGHP) Simon Ndira, simon.ndira@giz.de		Yes	Yes			Yes			1. Information	N. health management information system
DHIS2 for COVID-19 Surveillance: Malawi Rebecca Potter, covid@dhis2.org			Yes				Yes		1. Information	V. Public health and disease surveillance

Post disaster IDSR Strengthening in 14 targeted districts of Malawi Joseph Wu, wcsg@lukeinternational.no			Yes				Yes		1. Information	V.Public health and disease surveillance system
Electronic Participant Locator (ePAL) Luke Banda, lsbanda@mlw.mw			Yes			Yes			1. Information	I. Emergency response system, V.Public health and disease surveillance system
BIOWINPHARMA Mamadou Dakouo, contact@biowinpharma.com		Yes					Yes		1. Information, 6. Efficiency	H. Electronic Medical Record, X. Shared Health Record and health information repositories
DHIS2 for COVID-19 Surveillance: Mali Rebecca Potter, covid@dhis2.org			Yes				Yes		1. Information	V. Public health and disease surveillance
Access to Infant and Maternal (AIM) Health Plus Mauritania Zeine Abidine Ba, zeine_abidine@wvi.org		Yes				Yes			1. Information 2. Availability, 3. Quality, 5. Utilization	F. Community - based information system, Q. Knowledge Management system
upSCALE Joaquim Rebelo, j.rebelo@malariaconsortium.org		Yes					Yes		1. Information, 2. Availability, 3. Quality, 5. Utilization	F. Community -based information system, Q. Knowledge management sytem
mAlert Safa Naraghi, safa@zenysis.com			Yes				Yes		1. Information, 6. Efficiency	I. Emergency response system, V.Public health and disease surveillance system
DHIS2 for COVID-19 Surveillance: Mozambique Rebecca Potter, covid@dhis2.org			Yes				Yes		1. Information	V. Public health and disease surveillance
Community Scorecard App_Mozambique Robert Worthington, rob@kwantu.net		Yes				Yes			1. Information 8. Accountability	F. Community-based Information System

Namibia Planned Parenthood Association (NAPPA) Erin Sullivan, erin.sullivan@jhpiego.org		Yes						Yes	1. Information, 6. Efficiency	H. Electronic medical record
DHIS2 for COVID-19 Surveillance: Niger (in development) Rebecca Potter, covid@dhis2.org			Yes					Yes	1. Information	V. Public health and disease surveillance
Act to Save (A2S) Ezéchiel Mahamane, ezechiel_mahamane@wvi.org		Yes				Yes			1. Information, 2. Availability	F.Community-based Information System,Q. Knowledge management system
USAID funded - CaTSS OpenMRS Electronic Medical Record System for 19 ART Clinics Benjamin Akinmoyeje, bakinmoyeje@msh.org		Yes	Yes					Yes	1. Information, 6. Efficiency	H. Electronic medical record
DHIS2 for COVID-19 Surveillance: Nigeria (in development) Rebecca Potter, covid@dhis2.org			Yes				Yes		1. Information	V. Public health and disease surveillance
SwissTPH x OpenFn: Automated, real-time data integration & disease monitoring Aleksa Krolls, aleksa@openfn.org		Yes	Yes				Yes		1. Information, 6. Efficiency	H. Electronic medical record, N. health management information system,
EmHealth-Paediatric Digital Health Initiative Dr Agbarakwe Chukwuemeka, brammy@emhealthglobal.org		Yes						Yes	1. Information, 6. Efficiency	H. Electronic medical record
Rwanda Health Analytics Platform (RHAP) Eliachim Ishimwe, eliachim@zenysis.com				Yes				Yes	1. Information, 6. Efficiency,	G. Data interchange interoperability and accessibility

DHIS2 for COVID-19 Surveillance: Rwanda Rebecca Potter, covid@dhis2.org			Yes				Yes		1. Information	V. Public health and disease surveillance
Rwanda Health Information Exchange System (RHIES) Julie Vanvolsem, julie@savics.org				Yes			Yes		1. Information, 6. Efficiency	G. Data interchange interoperability and accessibility
DHIS2 for COVID-19 Surveillance: Sao Tome and Principe (in development) Rebecca Potter, covid@dhis2.org			Yes					Yes	1. Information	V. Public health and disease surveillance
NTD SCH-STH prevalance survey Clara Burgert, cburgert@rti.org			Yes					Yes	1. Information	W. Research information
DHIS2 for COVID-19 Surveillance: Senegal Rebecca Potter, covid@dhis2.org			Yes				Yes		1. Information	V. Public health and disease surveillance
electronic Integrated Disease Surveillance and Response (eIDSR) Nelson Clemens, nelson.clemens@sl.ehealthafrica.org			Yes				Yes		1. Information	V. Public health and disease surveillance
CHAMPS DAYO SPENCER-WALTERS, dayo.spencer-walters@sl.ehealthafrica.org		Yes						Yes	1. Information	B. Civil Registration and Vital Statistics
VaxTrac Dayo Spencer-Walters, dayo.spencer-walters@sl.ehealthafrica.org		Yes						Yes	1. Information, 6. Efficiency	H. Electronic medical record
AVADAR: Auto-Visual AFP Detection and Reporting Faye Simmonds, faye.simmonds@sl.ehealthafrica.org			Yes					Yes	1. Information	V. Public health and disease surveillance

117 Alerts System Dayo Spencer-Walters, dayo.spencer-walters@sl.ehealthafrica.org		Yes	Yes					Yes	1. Information	V. Public health and disease surveillance
Electronic Vaccination Record and Tracking System Implementation Project (VaxTrac) Musa Bernard Komeh, musa.komeh@sl.ehealthafrica.org		Yes						Yes	1. Information, 6. Efficiency	H. Electronic medical record, Q. Knowledge management system
Saving Lives Mohammad B. Jalloh, mbjalloh@focus1000.org		Yes						Yes	1. Information, 5. Utilization, 6. Efficiency	H. Electronic medical records
RapidPro Kazutaka Sekine, ksekine@unicef.org		Yes	Yes				Yes		1. Information,	N. Health management information system
Access to Infant and Maternal (AIM) Health Plus Sierra Leone Joseph Musa, joseph_musa@wvi.org		Yes				Yes			1. Information, 2. Availability,	F. Community-based Information System, Q. Knowledge Management
Global Open Facility Registry (GOFR) Reconciliation Tool Telli Koroma, tellikoroma@gmail.com				Yes				Yes	1. Information	G. Data interchange interoperability and accessibility
DHIS2 for COVID-19 Surveillance: Sierra Leone Rebecca Potter, covid@dhis2.org			Yes				Yes		1. Informaiton	V. Public health and disease surveillance
Instant OpenHIE Daniel Futerman, daniel.futerman@jembi.org			Yes					Yes	1. Information,	N. Health information management information system
OpenHIM (Open Health Information Mediator) Daniel Futerman, daniel.futerman@jembi.org				Yes				Yes	1. Information,	G. Data interchange interoperability and accessibility
NACOSA Health Analytics Platform (NHAP) Safa Naraghi, safa@zenysis.com			Yes				Yes		1. Information,	N. Health management information system

BroadReach COVID-19 Pandemic Management Fiona de Korte, fdekorte@brhc.com		Yes	Yes				Yes		1. Information, 2. Availability,5. Utilization , 6. Efficiency	N. Health management information system, V. Public health and disease surveillance system
DHIS2 for COVID-19 Surveillance: South Africa Rebecca Potter, covid@dhis2.org			Yes				Yes		1. Information	V. Public health and disease surveillance
HIV Solution powered by Vantage Todd Malone, Todd.Malone@brhc.com			Yes				Yes		1. Information	N. Health information management information system, V. Public health and disease surveillance system
COVID 19 - Safe Entry Management Kent Perils, kent@bcpi.co.za			Yes					Yes	1. Information	V. Public health and disease surveillance
eLQAS: Collecting real-time polio vaccination data Yaw Anokwa, yanokwa@nafundi.com		Yes						Yes	1. Information	N. Health management information system
Improving Healthcare Capacity and Access Using THINKMD's Clinical Decision Support Tool in a Humanitarian Setting Meg McLaughlin, mmclaughlin@thinkmd.org		Yes				Yes			1. Information, 6. Efficiency	H. Electronic medical record
NEMO Andrew Nute, andrew.nute@cartercenter.org			Yes					Yes	1. Information	W. Research Information System
DHIS2 for COVID-19 Surveillance: Sudan (in development) Rebecca Potter, covid@dhis2.org			Yes					Yes	1. Information	V. Public health and disease surveillance
Togo Health Analytics Platform (THAP) Quentin Perrot, quentin@zenysis.com				Yes			Yes		1. Information, 6. Efficiency,	G. Data interchange interoperability and accessibility

mTrac Sean Blaschke, sblaschke@unicef.org			Yes				Yes		1. Information,	N. Health information management system, V. Public health and disease surveillance system
UgandaEMR (OpenMRS) Jonathan Mpango, jmpango@musph.ac.ug		Yes					Yes		1. Information , 6. Efficiency	H. Electronic medical record
STRE@MLINE Mr Samuel Mugisha, CEO Innovation Streams Ltd, samuel@innovationstreams.tech		Yes				Yes			1. Information, 6. Efficiency,	K. Facility management information system
SmartHealth App in Uganda Nii Amon Dsane, ndsane@livinggoods.org		Yes						Yes	1. Information, 2. Availability, 3. Quality	F. Community -based information system,Q. Knowledge Management,T. Logistics management information system
Bringing Back Mothers and Babies Kisaakye Linda, lkisaakye@gmail.com		Yes						Yes	1. Information, 5. utilization, 6. Efficiency	H. Electronic medical record
DREAMS/OVC Juliet Cheptoris, julietcheptoris@gmail.com		Yes	Yes					Yes	1. Information, 6. Efficiency,	H. Electronic medical record, N. Health management information system,
Early Infant Diagnosis(EID) Dashboard Dr. Victor Bigira, vbigira@musph.ac.ug		Yes	Yes					Yes	1. Information, 6. Efficiency,	H. Electronic medical record
Option B+ Kisaakye Linda, lkisaakye@gmail.com		Yes						Yes	1. Information, 5. Utilization	N. Health management information system
Smart Hospital Information System (SmartHIS) Dr Simon Ndira, simon.ndira@compelling.works		Yes				Yes			1. Information, 6. Efficiency	K. Facility management information system

Performance Tracking App for CHW Supervisors Simon Mutama, smutama@livinggoods.org		Yes	Yes					Yes	1. Information, 3. Quality,	F. Community-based informatin system, O. Human resources information system, Q. Knowledge management system
Strategic Information Mobile Apllication Jonathan Mpango, vbigira@musph.ac.ug		Yes	Yes					Yes	1. Information	N. Health information management system,
Viral Load Dashboard Dr. Victor Bigira, vbigira@musph.ac.ug			Yes				Yes		1. Information	N. Health management information system
FamilyConnect Dr. Jesca Nsungwa Sabiiti, jnsabiiti@gmail.com		Yes						Yes	1. Information	B. Civil Registration and Vital Statistics, F. Community-based information system,
NTD LF pre-TAS and TAS survey Clara Burgert, cburgert@rti.org			Yes					Yes	1. Information	W. Research Information System
Stre@mline Samuel Mugisha, samuel@streamlinehealth.org		Yes						Yes	1. Information, 5. Utilization, 6. Efficiency	H. Electronic medical record, D. Client communication system
Open Client Registry Wayan Vota, wvota@intrahealth.org		Yes						Yes	1. Information, 6. Efficiency	H. Electrronic medical record, X. SHR and health information repositories
DHIS2 for COVID-19 Surveillance: Uganda Rebecca Potter, covid@dhis2.org		Yes	Yes				Yes		1. Information	V. Public health and disease surveillance
Buikwe Maternal Newborn and Child Health (B-MNCH) Project Angella Akolla, angella_akolla@wvi.org		Yes				Yes			1. Information, 8. Accountability	Q. Knowledge management system
Public Health Open Community Mapping Kiggudde Deogratius, deogratias@mapuganda.org		Yes				Yes			1. Information	F. Community-based Information System, L. Geographic Information system

Health Information Mediator Ssanyu Nyinondi, ssanyu_nyinondi@jsi.com				Yes			Yes		1. Information, 6. Efficiency	G. Data interchange interoperability and accessibility
Community Family Planning and Model Household Systems; Population Health Environment (PHE) Rebecca Litner, rlitner@d-tree.org		Yes						Yes	1. Information, 5. Utilization, 8. Accountability	F. Community based information system, Q. Knowledge management system
Sauti- HIV Prevention Program Gloria Kahamba, gkahamba@d-tree.org		Yes						Yes	1. Information, 5. Utilization, 6. Efficiency	H. Electronic medical record
Family Planning Digital Health System for Community Health Workers- Mara Region with Shirati KMT Hospital Gloria Kahamba, gkahamba@d-tree.org		Yes						Yes	1. Information	F. Community-based health system
BID INITIATIVE Hassan Mtenga, Laurie Warner, hmtenga@path.org		Yes						Yes	1. Information, 5. Utilization, 6. Efficiency	H. Electronic medical record
USAID BORESHA AFYA - COMMUNITY HEALTH INFORMATION SYSTEM ENCOMPASSING OPEN SMART REGISTER PLATFORM, CLOSED USER GROUP & ELEARNING Dunstan Bishanga, Dunstan.Bishanga@jhpiego.org		Yes				Yes			1. Information, 2. Availability, 5. Utilization, 6. Efficiency	H. Electronic medical record, I. Emergency response system, S. Learning and training system
SAUTI PROJECT-KVP MOBILE DATA COLLECTION SYSTEM Dr Albert Komba, Albert.Komba@jhpiego.org		Yes						Yes	1. Information,	F. Community-based health information system

Family Planning Digital Health System for Community Health Workers - Shinyanga Region Gloria Kahamba, gkahamba@d-tree.org		Yes						Yes	1. Information,	F. Community-based Information System,
Shang Ring Electronic Data Collection Alice Christensen, alice.christensen@jhpiego.org		Yes						Yes	1. Information	W. Research Information System
ARTIFICIAL INTELLIGENCE IN SOCIAL MEDIA DURING CORONA VIRUS DISEASE OUTBREAK. DEOGRATIAS MZURIKWAO, deogratias@xsenseai.co.tz			Yes					Yes	1. Information	W. Research Information System
DHIS2 for COVID-19 Surveillance: Zanzibar (in development) Rebecca Potter, covid@dhis2.org			Yes					Yes	1. Information	V. Public health and disease surveillance
Jamii ni Afya Heiko Hornung, hhornung@d-tree.org		Yes					Yes		1. Information	F. Community-based information system, Q. Knowledge management system
Access to Infant and Maternal (AIM) Health Plus Tanzania Daudi Sosthen Gambo, daudi_gambo@wvi.org		Yes				Yes			1. Information, 2. Availability, 5. Utilization	F. Community-based information system, Q. Knowledge management system
Hyperlocal Geospatial Data for Improving Vaccination Campaigns Asha Mustapher, omdtanzania@gmail.com			Yes					Yes	1. Information	F. Community-based information system, L. Geographic information system,
ZEIR - Zambia Electronic Immunization Registry Laurie Werner, lwerner@path.org		Yes					Yes		1. Information, 5. Utilization , 6. Efficiency	H. Electronic medical record

Zambia health Analytics Platform (ZHAP) Kaluba Mataka, kaluba@zenysis.com			Yes				Yes		1. Information,	N. Health management information system, T. Logistics management information system
Zambia National Data Warehouse Andrew Kashoka, andrew.kashoka@moh.gov.zm				Yes			Yes		1. Information, 6. Efficiency	G. Data interchange interoperability and accessibility
DHIS2 for COVID-19 Surveillance: Zambia (in development) Rebecca Potter, covid@dhis2.org			Yes					Yes	1. Information	V. Public health and disease surveillance
IDeAS. Integrated Decisions and Analytics Support. Mandy Dube, MDube@brhc.com		Yes					Yes		1. Information, 6. Efficiency	H. Electronic medical records
SmartCare Electronic Health Record System Andrew Kashoka, andrew.kashoka@moh.gov.zm		Yes					Yes		1. Information, 6. Efficiency	H. Electronic medical record
Zambia Anternatal Care (ANC) Digital Adapation Project I Implementation Research Caren Chizuni, carenchizuni@gmail.com		Yes				Yes			1. Information , 3. Quality	F. Community-based information system,Q. Knowledge Management
Implementation of a Performance-Based Financing Data System for Zimbabwe - using the open source tool Hesabu Benson Muzenda, Benson.Muzenda@cordaid.org			Yes				Yes		1. Information, 3. Quality	M. Health finance and insurance information system
Impilo EHR Dr. Robert Gongora, rgongora536@gmail.com		Yes					Yes		1. Information, 6. Efficiency,	H. Electronic medical record
Health Surveys			Yes			Yes			1. Information	W. Research information system
Patient records		Yes						Yes	1. Information, 6. Efficiency,	H. Electronic medical record

Diseaser Surveillance			Yes					Yes	1. Information	V. Public Health and disease suveillance system
Health Surveys			Yes				Yes		1. Information	W. Research information system
Diseaser Surveillance			Yes				Yes		1. Information	V. Public Health and disease suveillance system
Patient records			Yes				Yes		1. Information, 6. Efficiency	H. Electronic medical record
Health Surveys			Yes				Yes		1. Information	W. Research information system
Disease Surveillace			Yes				Yes		1. Information	V. Public Health and disease suveillance system
Disease Surveillance			Yes				Yes		1. Information	V. Public Health and disease suveillance system
Health Surveys			Yes			Yes			1. Information	W. Research information system
Disease surveillance			Yes			Yes			1. Information	V. Public Health and disease suveillance system
Health survey			Yes		Yes	Yes			1. Information	W. Research information system
Patient records			Yes		Yes	Yes			1. Information, 6. Efficiency	H. Electronic medical record
Health Survey			Yes				Yes		1. Information	W. Research information system
Disease Surveillance			Yes				Yes		1. Information	V. Public Health and disease suveillance system
Patient records		Yes				Yes			1. Information, 6. Efficiency	H. Electronic medical record
Health survey			Yes			Yes			1. Information	W. Research information system
Patient records		Yes					Yes		1. Information, 6. Efficiency	H. Electronic medical records
Health survey			Yes				Yes		1. Information	W. Research information system
Disease Surveillance			Yes		Yes				1. Information	V. Public health and disease surveillance system
Patient records		Yes				Yes			1. Information, 6. Efficiency	H. Electronic medical records

Health survey			Yes				Yes		1. Information	W. Research information system
Disease surveillance			Yes			Yes			1. Information	V. Public health and disease surveillance system
Health survey			Yes			Yes			1. Information	W. Research information system
Disease Surveillance			Yes			Yes			1. Information	V. Public health and disease surveillance system
Health survey			Yes				Yes		1. Information	W. Research information system
Disease Surveillance			Yes				Yes		1. Information	V. Public health and disease surveillance system
Patient records		Yes					Yes		1. Information, 6. Efficiency	H. Electronic medical records
Disease Surveillance			Yes				Yes		1. Information	V. Public health and disease surveillance system
Patient records		Yes				Yes	Yes		1. Information, 6. Efficiency	H. Electronic medical records
Health survey			Yes				Yes		1. Information	W. Research information system
Disease Surveillance			Yes			Yes	Yes		1. Information	V. Public health and disease surveillance system
Patient records		Yes				Yes			1. Information, 6. Efficiency	H. Electronic medical records
Health survey			Yes				Yes		1. Information	W. Research information system
Disease Surveillance			Yes				Yes		1. Information	V. Public health and disease surveillance system
Health survey			Yes				Yes		1. Information	W. Research information system
Patient records		Yes					Yes		1. Information, 6. Efficiency	H. Electronic medical records
Health survey			Yes				Yes		1. Information	W. Research information system
Disease Surveillance			Yes				Yes		1. Information	V. Public health and disease surveillance system
Health survey			Yes					Yes	1. Information	W. Research information system

Disease Surveillance			Yes					Yes	1. Information	V. Public health and disease surveillance system
Health survey			Yes				Yes		1. Information	W. Research information system
Disease Surveillance			Yes				Yes		1. Information	V. Public health and disease surveillance system
USAID Digital Health Activity (DHA), https://www.jsi.com/project/digital-health-activity/			Yes				Yes		1. Information, 2. Availability, 3. Quality, 6. Efficiency	N. Health management information system, V. Public health and disease surveillance system
Cloud-based electronic data system, Jede et al;2020		Yes				Yes			1. Information, 6. Efficiency	H. Electronic medical record
COVID-19 Tracker App, https://www.itu.int/hub/2020/05/ghana-launches-covid-19-tracker-app/			Yes				Yes		1. Information	N. Health management information system, V. Public health and disease surveillance system
Real-Time Monitoring and Evaluation of a Visual-Based Cervical Cancer Screening Program Using a Decision Support Job Aid, Peterson et al 2016		Yes				Yes			1. Information, 3. Quality ,6. Efficiency	H. Electronic medical record , Q. Knowledge management system
Mobile research, Leidich et al; 2018			Yes			Yes			1. Information	W. Research information system
The Health and Demographic Surveillance System (HDSS), Kaneko et al 2012			Yes			Yes			1. Information	F. Community based information system
PANDA (pregnancy and newborn diagnosis assessment) system, Garcia Vilaplana et al;2020		Yes				Yes			1. Information, 2. Availability, 5. Utilization, 7. Cost	H. Electronic medical record
A Mobile Health Data Collection System, Quercia et al; 2018		Yes				Yes			1. Information	F. Community based information system

NeoTree digital capture and quality improvement system Mgusha et al 2021		Yes				Yes			1. Information, 3. Quality, 6. Efficiency	H. Electronic medical record
Wellvis COVID triage tool, Owoyemi et al;2021	Yes						Yes		1. Information, 2. Availability,6. Efficiency, 7.Cost , 8. Accountability	C. Client application, V. Public health and disease surveillance system
Mobile tracking of those with coronavirus, https://www.reuters.com/article/health-coronavirus-telkom-sa/s-africa-launches-mobile-tracking-of-those-with-coronavirus-idUSL8N2BQ61L			Yes				Yes		1. Information	N. Health management information system, V. Public health and disease surveillance system
Electronic booking system, Booyse et al; 2020		Yes				Yes			1. Information	H. Electronic medical records

Table S6: Supplementary material: Digital Health Interventions used to strengthen Access to Essential Medicines, Vaccines, and Technology in sub-Saharan Africa

**Name/Reference/ Point of contact**	**Target-User**				**Stage of development**				**Addressed Health System Challenge**	**Aligned Systems Category**
	**Clients**	**Providers**	**Managers**	**Data Services**	**Informal**	**Pilot**	**Established**	**Unknown**		
OpenLMIS Angola (SIGLOFA) Rebecca Alban, info@openlmis.org			Yes				Yes		1. Information, 2. Availability, 6. Efficiency, 8. Accountability	T. Logistics management information system (LMIS)
Angola immunization supply chain Neeraj Thakare, neeraj@logistimo.com			Yes				Yes		1. Information, 2.Availability, 6. Efficiency, 8. Accountability	T. Logistics management information system (LMIS)
OpenLMIS Benin (SIIL) Rebecca Alban, info@openlmis.org		Yes	Yes				Yes		1. Information, 2. Availability, 6. Efficiency, 8. Accountability	T. Logistics management and information system (LMIS)
The Prevention Pack Program: ensuring availability of post-rape medicines in rural clinics Jean Armas, jean.armas@globalstrategies.org		Yes	Yes			Yes			2.Availability, 5. Utilization,	I. Emergency response system
OpenLMIS Cote d'Ivoire (eSIGL) Rebecca Alban, info@openlmis.org		Yes	Yes				Yes		1. Information, 2. Availability, 6. Efficiency ,8. Accountability	T. Logistics management information system (LMIS)
Quntimed and Quentitb, Federal Ministry of Health, dha@moh.gov.et			Yes				Yes		1. Information, 2. Availability, 6. Efficiency, 8. Accountability	T. Logistics management information system (LMIS)

Pipeline Federal Ministry of Health, dha@moh.gov.et			Yes				Yes		1. Information, 2. Availability, 6. Efficiency,8. Accountability	T. Logistics management information system (LMIS)
IVR (Interactive voice response) Federal Ministry of Health, dha@moh.gov.et		Yes					Yes		1. Information, 2. Availability, 6. Efficiency,8. Accountability	B. Civil registration and vital statistics, T. Logistics management information system
EPS pharmaceutical information systems (ARSMBDHS) Federal Ministry of Health, dha@moh.gov.et			Yes				Yes		1. Information, 2. Availability,6. Efficiency, 8. Accountability	T. Logistics management information system (LMIS)
ECHMIS (Electronic Communication HMIS) Federal Ministry of Health, dha@moh.gov.et		Yes					Yes		1. Information, 2. Availability,6. Efficiency, 8. Accountability	T. Logistics management information system (LMIS)
Dagu Federal Ministry of Health, dha@moh.gov.et		Yes					Yes		1. Information, 2. Availability, 6. Efficiency, 8. Accountability	T. Logistics management information system (LMIS)
CCEIT Federal Ministry of Health, dha@moh.gov.et			Yes				Yes		1. Information, 2. Availability, 8. Accountability	T. Logistics management information system (LMIS)
OpenLMIS Guinea Rebecca Alban, info@openlmis.org			Yes				Yes		1. Information, 2. Availability, 6. Efficiency	T. Logistics management information system (LMIS)
cStock/DHIS2 Zoya Mohammed, zoya_mohamed@insupplyhealth.com			Yes			Yes			1. Information, 2. Availability ,6. Efficiency, 8. Accountability	F. Community-based Information System, T. Logistics management information system
Medkit.Network Dr. Louis Somoni Machogu, dr.somoni@gmail.com	Yes							Yes	1. Information, 3. Quality	C. Client applications,P. Identification registries and directories, U. Pharmacy information system
DawaSure francis munyeki, munyekif@cadcreations.co.ke	Yes					Yes			2. Availability, 5.Utilization	C. Client applications, U. Pharmacy information system

Electronic Logistics Management and Inventory Management System Monaheng Maoeng, monaheng.maoeng@gov.ls		Yes					Yes		1. Information 2. Availability , 6. Efficiency, 8. Accountability	T. Logistics and diagnostics information system
cStock - EPI Erick Mwale, Erick.Mwale@savethechildren.org		Yes					Yes		1. Information, 2. Availability, 6. Efficiency , 8. Accountability	T. Logistics management information system (LMIS)
CMAM Stock Monitoring System Malawi Sylvester Kathuma, kathumbasylvester@gmail.com			Yes					Yes	1. Information, 2. Availability, 6. Efficiency , 8. Accountability	T. Logistics management information system (LMIS)
OpenLMIS Malawi Rebecca Alban, info@openlmis.org		Yes	Yes				Yes		1. Information, 2. Availability, 6. Efficiency, 8. Accountability	T. Logistics management information system (LMIS)
OpenLMIS Mozambique (SELV) Rebecca Alban, Info@openlmis.org		Yes	Yes				Yes		1. Information, 2. Availability, 6. Efficiency , 8. Accountability	T. Logistics management information system (LMIS)
OpenLMIS Mozambique (SIGLUS) Rebecca Alban, Info@openlmis.org		Yes	Yes				Yes		1. Information, 2. Availability, 6. Efficiency,8. Accountability	T. Logistics management information system (LMIS)
PharmaDexMZ Kim Hoppenworth, khoppenworth@mtapsprogram.org			Yes					Yes	1. Information	U. Pharmacy Information System
Real-Time Monitoring of Moderna Vaccines in collaboration with National Primary Health Care Development Agency. Oghenetega Iortim, tega@gricd.com		Yes	Yes				Yes		1. Information, 3. Quality	T. Logistics management information system (LMIS)

PHARMARUN IBITAYO TAIWO, tayotaiwo83@yahoo.com	Yes							Yes	2. Availability	C. Client applications
Informed Push Model - Yeksi-Naa Melanie Joiner, mjoiner@intrahealth.org			Yes					Yes	1. Information, 2. Availability, 6. Efficiency, 8. Accountability	T. Logistics management information system (LMIS)
Senegal immunization supply chain Neeraj Thakare, neeraj@logistimo.com			Yes			Yes			1. Information, 2. Availability, 6. Efficiency, 8. Accountability	T. Logistics management information system (LMIS)
eLMIS Facility Edition (FE) Ashraf Islam, ashraf_islam@jsi.com			Yes						1. Information, 2. Availability,6. Efficiency, 8. Accountability	T. Logistics management information system (LMIS)
RxSolution Stephanie Xueref, smxueref@msh.org		Yes	Yes			Yes			1. Information, 2. Availability,	T. Logistics management information system (LMIS)
Pharmaceutical Information Portal Stephanie Xueref, smxueref@msh.org				Yes			Yes		1. Information	N. Health management information system
mTrac Uganda Carol Kyozira, ckyozira@gmail.com		Yes	Yes						1. Information, 2. Availability, 6. Efficiency, 8. Accountability	T. Logistics management information system (LMIS)
Electronic Inventory Management Services System (e-IMSS) Cliton Chikwata, clinton.chikwata@tmcg.co.ug		Yes					Yes		1. Information, 2. Availability	T. Logistics management information system (LMIS)
Vaccine Information Management System (VIMS) Ssanyu Nyinondi, snyinondi@tz.jsi.com			Yes	Yes			Yes		2. Availability	T. Logistics management information system, G. Data interchange interoperability and accessibility
OpenLMIS Tanzania Rebecca Alban, Info@openlmis.org			Yes				Yes		1. Information, 2. Availability ,6.	T. Logistics management information system (LMIS)

									Efficiency, 8. Accountability	
AfyaIntelligence Harrison Mariki, afyaintelligence@gmail.com		Yes	Yes					Yes	1. Information, 2. Availability, 6. Efficiency 8. Accountability	T. Logistics management information system (LMIS), N. Health management information system
eLMIS Facility Edition (FE) Wendy Bomett, wendy_bomett@zm.jsi.com		Yes	Yes					Yes	1. Information, 2. Availability ,6. Efficiency, 8. Accountability	T. Logistics management information system
Zambia immunization supply chain Arun Ramanujapuram, arun@logistimo.com		Yes	Yes			Yes			1. Information, 2. Availability, 6. Efficiency, 8. Accountability	T. Logistics management information system
Supply chain information system			Yes					Yes	1. Information, 2. Availability, 6. Effiiciency 8. Accountability	T. Logistics management information system
Supply chain information system		Yes	Yes					Yes	1. Information, 2. Aailability, 6. Efficiency, 8. Accountability	T. Logistics management information system
Supply chain information system		Yes	Yes					Yes	1. Information,2. Availability, 6. Efficiency, 8. Accountability	T. Logistics management information system
Supply chain information system		Yes	Yes					Yes	1. Information,2. Availability, 6. Efficiency, 8. Accountability	T. Logistics management information system
Supply chain information system		Yes	Yes					Yes	1. Information, 2. Availability, 6. Efficiency,8. Accountability	T. Logistics management information system
Supply chain information system		Yes	Yes					Yes	1. Information, 2, Availability,6. Efficiency, 8. Accountability	T. Logistics management information system

Supply chain information system		Yes	Yes					Yes	1. Information, 2. Availability, 6. Efficiency, 8. Accountability	T. Logistics management information system
Supply chain information system		Yes	Yes					Yes	1. Information,2. Availability, 6. Efficiency, 8. Accountability	T. Logistics management information system
Supply chain information system		Yes	Yes					Yes	1. Information,2. Availability, 6. Efficiency, 8. Accountability	T. Logistics management information system
Supply chain information system		Yes	Yes					Yes	1. Information, 2. Availability, 6. Efficiency, 8. Accountability	T. Logistics management information system
Supply chain information system		Yes	Yes					Yes	1. Information, 2. Availability, 6. Efficiency, 8. Accountability	T. Logistics management information system
Supply chain information system		Yes	Yes					Yes	1. Information,2. Availability, 6. Efficiency, 8. Accountability	T. Logistics management information system
Supply chain information system		Yes	Yes					Yes	1. Information, 2 Availability, 6. Efficiency, 8. Accountability	T. Logistics management information system
Supply chain information system		Yes	Yes					Yes	1. Information, 2. Availability, 6. Efficiency, 8. Accountability	T. Logistics management information system
Supply chain information system		Yes	Yes					Yes	1. Information, 2. Availability, 6. Efficiency, 8. Accountability	T. Logistics management information system
Supply chain information system		Yes	Yes					Yes	1. Information, 2. Availability, 6. Efficiency, 8. Accountability	T. Logistics management information system

Uber for Blood, McVeigh, K. 2018. "'Uber for Blood': How rwandan Delivery robots Are Saving lives." The Guardian		Yes				Yes			2. Availability, 6. Efficiency	T. Logistics management information system
mTrac UNICEF Uganda, 2011		Yes					Yes		1. Information, 2. Availability,	T. Logistics management information system (LMIS)
mHealth application to strengthening pharmaceutical systems, Namisango et al; 2016		Yes				Yes			1. Information, 6. Efficiency	U. Pharmacy information system

Table S7: Supplementary material: Digital Health Interventions used to strengthen Healthcare Finance in sub-Saharan Africa

**Name/Reference/ Point of contact**	**Target-User**				**Stage of development**				**Addressed Health System Challenge**	**Aligned Systems Category**
	**Clients**	**Providers**	**Managers**	**Data Services**	**Informal**	**Pilot**	**Established**	**Unknown**		
Implementation of a Performance-Based Financing Data System for Cameroon - using the open source tool Hesabu Mfouapon N Henock, National Data Manager of PBF, henockmfouapon@yahoo.fr			Yes				Yes		1. Information, 3. Quality	C. Client applications, N. Health Management Information System
Providers System Federal Ministry of Health, dha@moh.gov.et			Yes				Yes		1. Information	M. Health finance and insurance system

Members system Federal Ministry of Health, dha@moh.gov.et			Yes				Yes		1. information	M. Health finance and insurance system
IFMIS - Integrated Financial Management Information System Federal Ministry of Health, dha@moh.gov.et			Yes				Yes	Yes	7.Cost	M Health finance and Insurance information system
IBEX (Integrated Budget Expenditure System IBEX) Federal Ministry of Health, dha@moh.gov.et			Yes				Yes		7. Cost	M. Health finance and insurance information system
CBHI (community based health insurance system) Federal Ministry of Health, dha@moh.gov.et			Yes				Yes		1. Information, 7. Cost	M. Health finance and insurance information system
M-TIBA mobile health wallet Maarten Ras, m.ras@carepay.co.ke	Yes						Yes		7.Cost	C. Client application
Connected Diagnostics for malaria Tobias Rinke de Wit, t.rinkedewit@pharmaccess.org		Yes				Yes			1. Information, 7. Cost	C. Client applications, R. Laboratory and Diagnostic System
Digital Cash Advance for the health sector Tom Bouma, t.bouma@medicalcreditfund.org			Yes				Yes		2. Availability	M. Health finance and insurance information systen
mTOMADY Jennifer Bencivenga, jb@mtomady.com	Yes	Yes				Yes			7.Cost	M. Health finance an insurane information system
CarePay mobile health wallet Steve Maina, s.maina@carepay.co.ke	Yes					Yes			7. Cost	M. Health finance and insurance information system
Lafya HealthShare Nanlir Kapnan, kapnan@lafya.co	Yes					Yes			1. Information, 3. Quality,7. Cost	C. Client applications, M. Health finance and insurance system
ulerawa luke shors, lshors@icloud.com			Yes			Yes			7. Cost	M. Health finance and insurance information system

NIA Juliana Busasi, juliannabusasi@gmail.com	Yes							Yes	7. Cost	M. Health finance and insurance information system
Electronic medical billing system		Yes						Yes	6. Efficiency, 8. Accountability	M. Health finance and insurance information system
Electronic billing system		Yes						Yes	6. Efficiency, 8. Accountability	M. Health finance and insurance information system
Electronic billing system		Yes						Yes	6. Efficiency, 8. Accountability	M. Health finance and insurance information system
Electronic medical billing system		Yes						Yes	6. Efficiency, 8. Accountability	M. Health finance and insurance information system
Electronic billing system		Yes						Yes	6. Efficiency, 8. Accountability	M. Health finance and insurance information system
Electronic billing system		Yes						Yes	6. Efficiency, 8. Accountability	M. Health finance and insurance information system
Electronic billing system		Yes						Yes	6. Efficiency, 8. Accountability	M. Health finance and insurane information system
Electronic billing system		Yes						Yes	6. Efficiency, 8. Accountability	M. Health finance and insurane information system
Electronic billing system		Yes						Yes	6. Efficiency, 8. Accountability	M. Health finance and insurance information system
Electronic billing system		Yes						Yes	6. Efficiency, 8. Accountability	M. Health finance and insurance information system
Electronic billing system		Yes						Yes	6. Efficiency, 8. Accountability	M. Health finance and insurance information system
Electronic billing system		Yes						Yes	6. Efficiency, 8. Accountability	M. Health finance and insurance information system
Electronic billing system		Yes						Yes	6. Efficiency, 8. Accountability	M. Health finance and insurance information system

i-PUSH, Abajobir et al; 2021	Yes					Yes			7.Cost	C. Client application, M. Health finance and insurance information system
The Mobile Maternal Health Wallet (MMHW), Lacroze et al; 2021	Yes					Yes			7. Cost	M. Health finance and insurance information system

Table S8: Supplementary material: Digital Health Interventions used to Leadership and Governance strengthen in sub-Saharan Africa

**Name/Reference/ Point of contact**	**Target-User**				**Stage of development**				**Addressed Health System Challenge**	**Aligned Systems Category**
	**Clients**	**Providers**	**Managers**	**Data Services**	**Informal**	**Pilot**	**Established**	**Unknown**		
Building a national health map in a week Yaw Anokwa, yanokwa@nafundi.com			Yes				Yes		1. Information	P. Identification registries and directories, W. Research information system
Community Scorecard App_Mozambique Robert Worthington, rob@kwantu.net		Yes				Yes			1. Information 8. Accountability	F. Community-based Information System
Automation and AI for Health Campaign Planning Sangeeta Jobanputra, sraja@connecti3.com			Yes			Yes			1. Information, 6. Efficiency	T. Logistics management information system (LMIS)
